# Adaptive variation in butyrylcholinesterase-influenced lipid metabolism during horse domestication

**DOI:** 10.1016/j.celrep.2026.117879

**Published:** 2026-08-20

**Authors:** Yanli Zhang, Xuexue Liu, Xintong Wang, Yanhui Yu, Yaozhen Jia, Yabin Pu, Yuehui Ma, Ludovic Orlando, Lin Jiang

**Affiliations:** 1 State Key Laboratory of Animal Biotech Breeding, Institute of Animal Sciences, Chinese Academy of Agricultural Sciences (CAAS), Beijing 100081, China; 2 National Germplasm Center of Domestic Animal Resources, Ministry of Technology, Institute of Animal Sciences, Chinese Academy of Agricultural Sciences (CAAS), Beijing 100081, China; 3 Centre for Anthropobiology and Genomics of Toulouse (CNRS/Université de Toulouse), Faculté de Santé, Bâtiment A, 37 Allées Jules Guesde, 31000 Toulouse, France; 4 College of Animal Science and Technology, Qingdao Agricultural University, Qingdao, Shandong 266109, China; 5 Gongzhuling City Animal Disease Prevention and Control Center, Gongzhuling, Jilin 136100, China

**Keywords:** serum biochemistry parameters, BCHE, butyrylcholinesterase, BChE, lipid metabolism, lipolysis, horse domestication, ancient DNA, positive selection

## Abstract

Lipid metabolism influences horse athletic performance, yet its genetic basis remains unclear. Here, we investigate the genetic basis of lipid metabolic traits by quantifying key metabolic parameters and sequencing the genomes of 298 horses. We identify a missense mutation (ECA19:9,198,989, G>C) in the *BCHE* gene that is strongly associated with lipid metabolism, especially butyrylcholinesterase (BChE) activity. The “C” allele is associated with increased BChE activity and reduced intracellular triglyceride levels, hepatic adiposity, and overall fat mass, highlighting a central role for *BCHE* in lipid hydrolysis and fat storage in horses. This variant evolved neutrally across most of the horse domestication history outside Asia, but underwent strong positive selection in Asia until the 13th century CE. This spatiotemporal divergence suggests that Asian and non-Asian horses experienced distinct selective pressures on lipid metabolism, possibly reflecting differences in management practices, environmental conditions, or energetic demands before the rise of the Great Mongolian Empire.

## Highlights

• One missense SNP in *BCHE* affects lipid metabolism in horses
• The C variant increases BChE activity and reduces lipid accumulation
• Cellular and mouse models support a role for *BCHE* in fat storage
• The C variant was positively selected in Asian horses until the mid-13th century CE

## Graphical abstract

**Figure:**
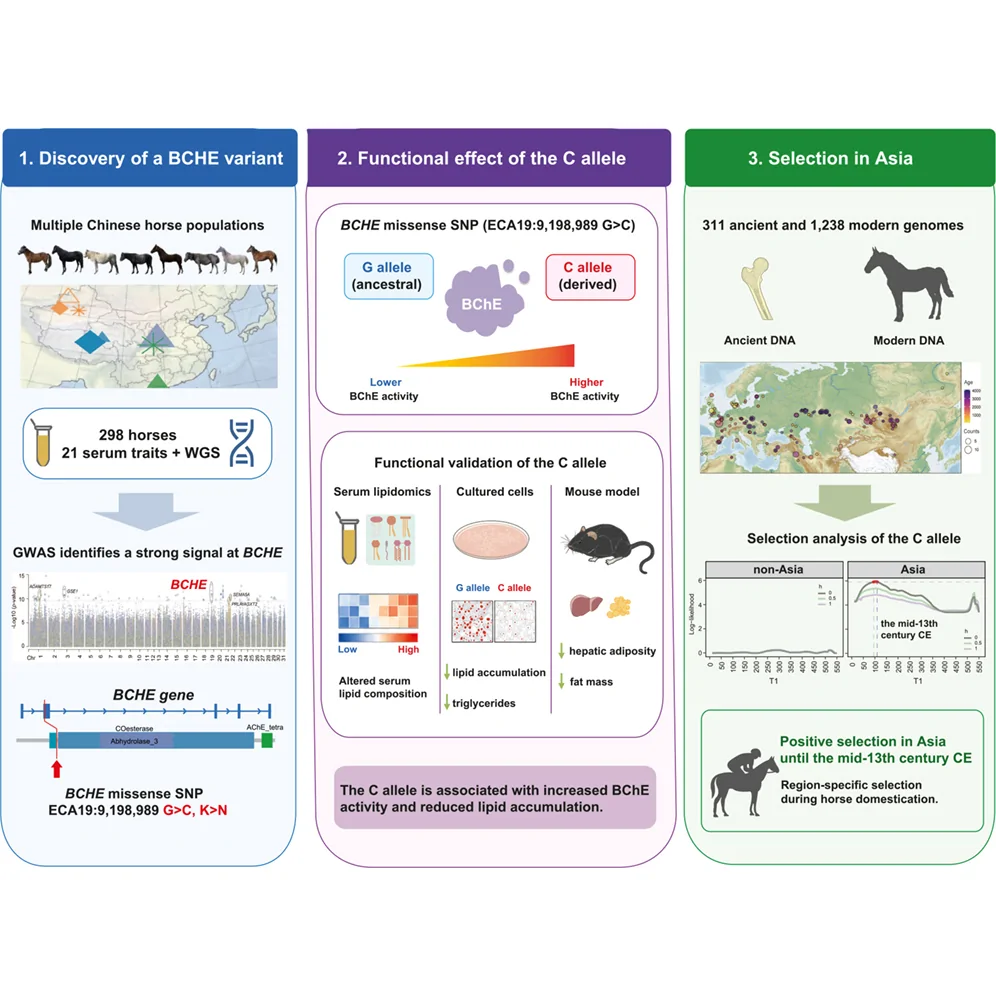

## Teaser

Zhang et al. identify a missense mutation in *BCHE* that affects lipid metabolism in horses. Cellular and mouse models show that the C variant increases BChE activity and reduces lipid accumulation. Ancient DNA analyses reveal positive selection in Asian horses until the mid-13th century CE.

## Introduction

Serum biochemistry parameters are tightly linked to metabolic processes as well as to the physiological and health status in both humans and animals. In humans, they not only provide key indicators for routine disease diagnosis^1^^,^^2^ and treatment monitoring but also for risk^3^^,^^4^ and prognosis assessment.^5^^,^^6^ Furthermore, serum biochemistry is key for monitoring energy metabolism in animals, and provides important predictors of performance capacity,^7^ production efficiency,^8^ and immune function.^9^

Previous work, including genome-wide association studies (GWASs) targeting blood biochemical traits, has identified numerous genetic loci significantly associated with human diseases.^6^^,^^10^^,^^11^^,^^12^ In comparison, similar research in non-human animals remains limited, although recent work has started to reveal important serum-related traits and their genetic basis. For example, multi-omic studies in racing horses such as Throughbreds^13^^,^^14^ and Yili horses^15^^,^^16^ identified plasma metabolites strongly associated with performance traits, with training and prolonged endurance exercise profoundly reshaping lipid composition, energy, and protein metabolic pathways.^17^^,^^18^^,^^19^ Shifts in metabolic profiles have also been associated with adiposity and metabolic syndromes, including bile acids segregating with horses showing higher metabolic risk,^20^ and genome-wide analyses incorporating metabolite phenotypes from equine metabolic syndrome cohorts have provided evidence for a heritable component of metabolic traits.^21^ Despite such advances, and the growing list of loci associated with serum biochemistry traits in species such as duck^22^^,^^23^^,^^24^ and chicken,^25^ precise characterization of the biological mechanisms linking genetic variation and metabolic traits is largely lacking, leaving their broader relevance and evolutionary history unclear.

In this study, we show that butyrylcholinesterase (BChE) plays a pivotal role in horse lipid metabolism, and that a high-activity variant of the *BCHE* gene has experienced shifting selection regimes in Asia over the last ∼4,000 years. We measured 21 serum biochemistry parameters and sequenced the genomes of 298 Chinese horses to assess genotype-phenotype association. GWAS identified *BCHE*, which encodes the BChE enzyme, as the most significant locus associated with BChE activity, as one of the 21 serum parameters measured. We also identified a strong correlation between BChE activity and body conformation traits. Lipidomic profiling and cellular assays confirmed that higher BChE activity improves the efficiency of lipid catabolism, thereby establishing a mechanistic link between the *BCHE* gene, BChE enzymatic function, and lipid metabolism.

Beyond advancing understanding of the central role that *BCHE* plays in lipid hydrolysis and fat storage in horses, our findings add important insights to the history of horse management and breeding, following their initial domestication in the Pontic-Caspian steppes in the second half of the third millennium BCE,^26^^,^^27^ and their further differentiation into local breeds or landraces adapted to specific activities and environments.^28^ In particular, the contrasted evolutionary trajectories of the low- and high-activity *BCHE* variants within and outside Asia suggest different responses to distinct environmental pressures. The signature of positive selection detected in Asia for the “C” allele, which limits hepatic adiposity and overall fat storage, may reflect local adaptation to the energetic constraints of steppe ecosystems, where water limitation and strong seasonality not only impose relatively low and fluctuating energy availability but also favor metabolic efficiency under highly mobile pastoral lifeways.^29^

## Results

### Genome sequencing and population structure

**Figure 1:**
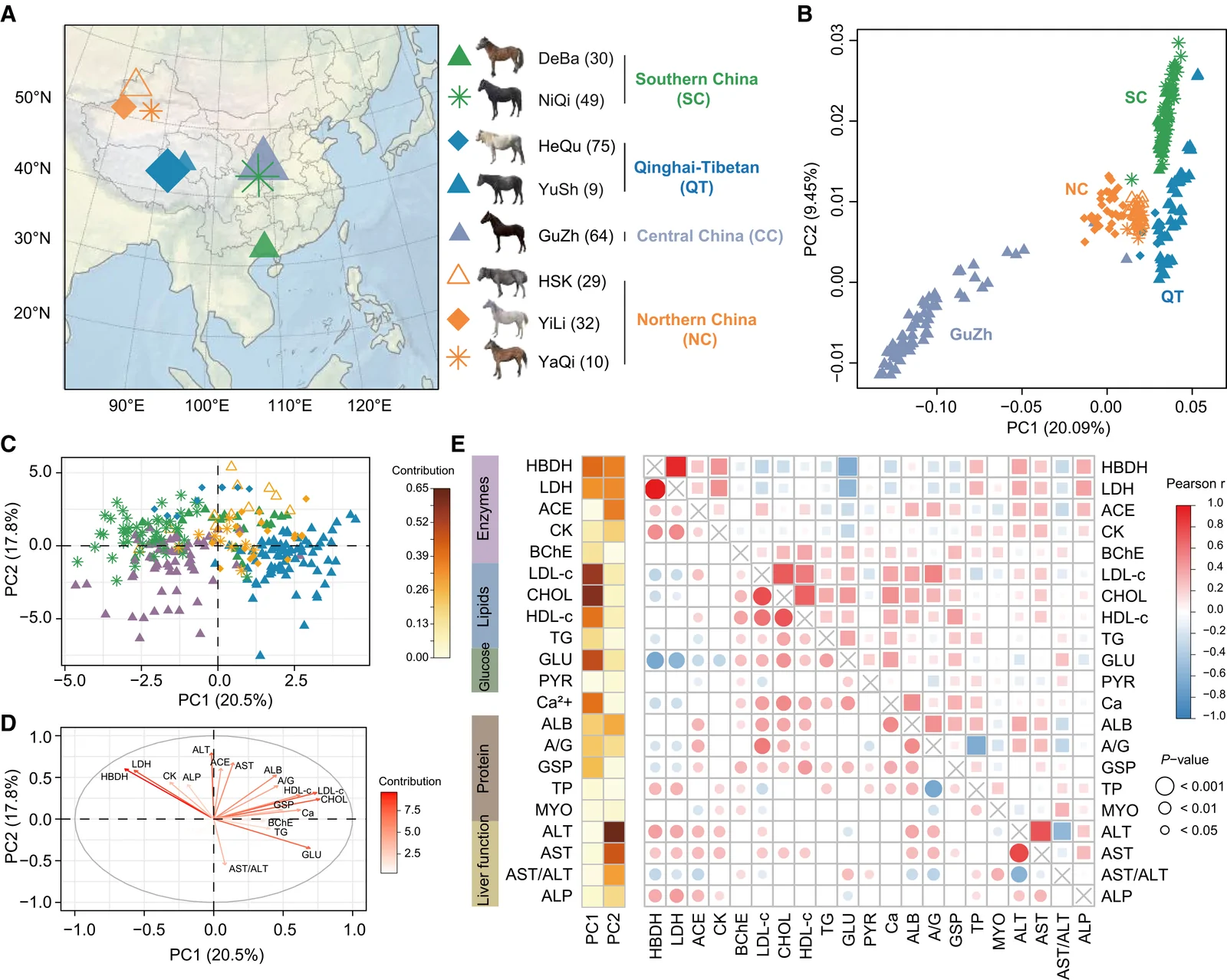
Integrated overview of the genetic background and molecular phenotype associations in Chinese horse breeds (A) Sample map, with symbols and colors reflecting horse breeds from across China. SC, Southern China; QT, Qinghai-Tibetan plateau; CC, Central China; NC, Northern China; DaTo, DaTong; DeBa, Debao; GuZh, Guanzhong; HeQu, Hequ; HSK, Kazakh; NiQi, NiQiang; YaQi, YanQi; YiLi, YiLi; YuSh, YuShu. (B) Principal component analysis (PCA) of autosomal SNP variation. The fraction of the total variance explained is reported between parentheses on each principal component. (C) PCA of 21 molecular phenotypes, with vectors indicating the contribution of each phenotype to the first two principal components. A/G, globulin ratio; ACE, angiotensin-converting enzyme activity (U/L); ALB, albumin concentration (g/L); ALP, alkaline phosphatase activity (U/L); ALT, alanine amino transferase activity (U/L); AST, aspartate amino transferase activity (U/L); AST/ALT, ratio of AST and ALT; BChE, butyrylcholinesterase activity (U/mL); Ca, calcium concentration (mmol/L); CHOL, cholesterol concentration (mmol/L); CK, creatine kinase concentration (U/L); GLU, glucose concentration (mmol/L); GSP, glycosylated serum protein concentration (μg/L); HBDH, α-hydroxy-butyrate dehydrogenase activity (U/L); HDL-c, high-density lipoprotein cholesterol concentration (mmol/L); LDH, lactate dehydrogenase activity (U/L); LDL-c, low-density lipoprotein cholesterol concentration (mmol/L); MYO, myoglobin concentration (μg/L); PYR, pyruvic acid concentration (mmol/L); TG, triglyceride concentration (mmol/L); TP, total protein concentration (g/L). (D) Individual contribution of each phenotype to the first two principal components. (E) Matrix of pairwise Pearson correlations between molecular phenotypes. The colors above the main diagonal are proportional to the correlation coefficients, while the size of the circles below the main diagonal indicate statistical significance. See also Figures S1–S5 and Table S1. Sample information for Chinese domestic horse populations, related to Figure 1, Table S2. Whole-genome sequencing statistics, related to Figure 1, Table S3. Serum biochemical parameters, related to Figure 1.

We collected blood samples from 298 horses representing eight Chinese domestic horse breeds, including Debao (DeBa), Ningqiang (NiQi), Hequ (HeQu), Yushu (YuSh), Guanzhong (GuZh), Kazakh (HSK), Yili (YiLi), and Yanqi (YaQi) horses (Figure 1A; Table S1; STAR Methods). Whole-genome sequencing was performed on the BGISEQ platform, yielding an average depth-of-coverage of ∼10.62× per individual (Median = 10.13×, range = 4.55–18.67×; Table S2). Our dataset presents, for the first time, genome-wide data from the YuSh horse, a remote breed, native to extreme high altitude (∼5,000 m above sea level).

After applying stringent quality control to the genome-wide dataset of 298 Chinese horses, we retained 19,878,270 high-quality SNPs for downstream analysis. Principal component analysis (PCA) and population structure analysis revealed four major genetic clusters, including Southern China ponies (SC; DeBa and NiQi), as well as horses from Northern China (NC; HSK, YaQi, and YiLi), the Qinghai-Tibetan (QT) plateau (HeQu and YuSh), and GuZh (Central China [CC]) horses (Figures 1B and S1A–S1C). These clusters are consistent with previous reports and highlight the genetic differentiation of these horse populations and regional adaptation.^30^^,^^31^

YuSh horses clustered closely with HeQu horses, another breed originating from the QT plateau, suggesting a shared evolutionary history shaped by adaptation to high-altitude environments. In contrast, the GuZh horse did not group with the NC cluster, despite its geographic location in NC. It formed instead a separate and genetically distinct lineage (Figure 1B). This divergence aligns with GuZh breeding history, which was derived as mixed ancestries from Budyonny (Russia) and Cleveland Bay (UK) horses (China National Commission of Animal Genetic Resources, 2011). GuZh horses also generally exhibited longer runs of homozygosity (ROHs) compared with other Chinese native breeds (Figures S1D–S1F), indicating lower genetic diversity and greater recent inbreeding levels.

Combined, our results suggest that the genetic structure of modern Chinese horse breeds has been shaped by both natural geographical isolation and human-mediated admixture.

### Serum biochemistry traits

Serum biochemistry indicators are blood-derived parameters closely linked to physiological processes and are often considered direct reflections of metabolic activity. The 21 serum biochemistry parameters measured in 298 Chinese horses (Table S3; STAR Methods) showed substantial variation, with coefficients of variation ranging from 14.93% for calcium concentration (Ca^2+^ to 117.92% for myoglobin concentration [MYO]) (Figures S2 and S3).

PCA revealed distinct patterns of variation among the measured parameters (Figure S4). The first two principal components explained 38.3% of the total variance (Figures 1C and 1D), indicating substantial dimensionality reduction. Pairwise correlation analysis based on variable contributions identified four distinct clusters, suggesting the presence of functional interdependencies amongst subsets of parameters, independent of sex (Figures 1E and S5).

Among these subsets, the two transaminases measured (aspartate aminotransferase [AST] and alanine amino transferase [ALT]) showed a strong positive correlation (*p* value = 2.20e−16), consistent with their shared role in hepatic metabolism (Figure 1E). Similarly, the myocardial enzymes creatine kinase (CK), lactate dehydrogenase (LDH), and α-hydroxybutyrate dehydrogenase (HBDH) were highly correlated (*p* values < 6.44e−16), and showed a negative relationship with blood glucose levels (Figure 1E), reflecting a metabolic trade-off between energy demand and glucose utilization.

Our analyses also revealed a distinct lipid-associated cluster, comprising triglycerides (TG), cholesterol (CHOL), high-density lipoprotein cholesterol (HDL-c), and low-density lipoprotein cholesterol (LDL-c), as well as albumin (ALB) and the albumin/globulin ratio (A/G) (*p* values < 2.56e−7) (Figure 1E). Interestingly, BChE showed a significant positive correlation with HDL-c (*p* value = 6.99e−5), and a negative correlation with LDL-c (*p* value = 0.02). This pattern may reflect the complex influence of BChE on lipid metabolism, potentially through interactions with cholesterol esterase.^32^ Similar associations have been reported in other mammalian species,^23^^,^^25^^,^^33^^,^^34^ suggesting a conserved biochemical mechanism across taxa.

### GWAS top-candidate locus for serum biochemistry traits

**Figure 2:**
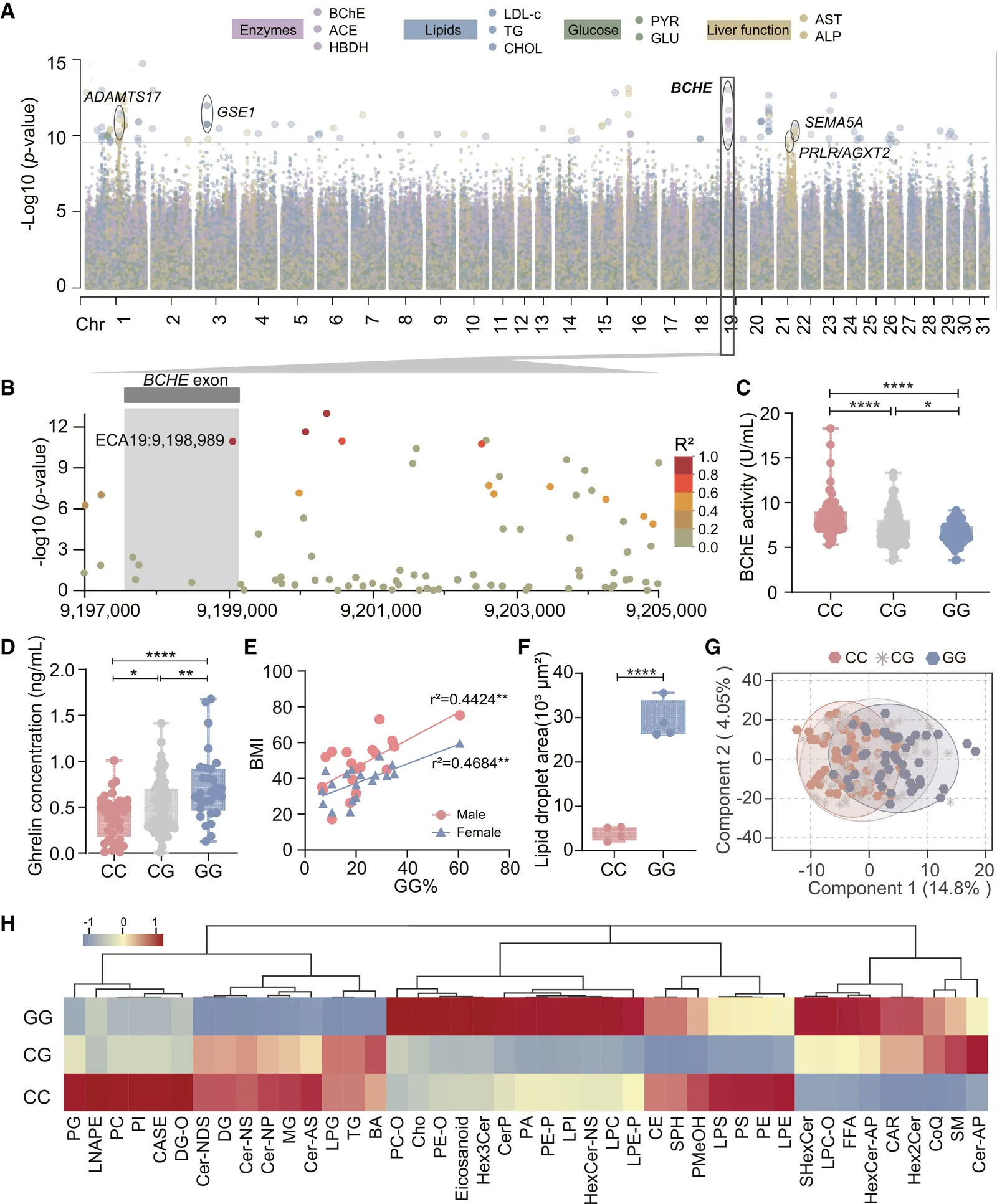
Statistical association between genotypes and phenotypes (A) Manhattan plot of −log_10_(*p* values) reflecting the statistical association between genome-wide genetic variation and seven serum molecular phenotypes. The gray dashed line indicates the genome-wide significance level, with Bonferroni correction for multiple testing (*p* value = 2.82e−9). The names of the genes annotated within the 1 kb region flanking individual significant SNPs are reported. Colors indicate the main phenotype category (liver function, enzymatic activity, and lipids concentration). (B) Zoom-in the region showing the top-significant associated SNPs, with linkage disequilibrium (LD) calculated within 500 kb flanking regions. The first two exons of *BCHE* are represented by gray boxes. The significant SNP located in the second exon (ECA19:9,198,989, G > C) is annotated. (C) BChE activity (U/mL) in horse serum for the three genotypes at ECA19:9,198,989. The *p* values of Kruskal-Wallis tests indicate the level of statistical significance. (D) Same as (C), for ghrelin concentration (mg/mL). (E) Pearson linear regression analysis between horse body mass index (BMI) and the frequency of the GG genotype at ECA19:9,198,989. (F) Lipid droplet area (10^2^ μm^2^) in seven homozygous horses at ECA19:9,198,989. Lipid droplet area is measured following Oil Red O staining of horse liver. (G) PCA of blood relative concentrations for 1,515 lipids. Ellipses indicate the 95% confidence interval of each individual genotype at ECA19:9,198,989. (H) Hierarchical clustering heatmap of blood relative concentrations for 1,515 lipids, grouped within 43 subclasses. Relative concentrations are z scored transformed, with zero values representing average values. Scores equal to (minus) one, thus, indicate values within one standard deviation above (below) the average. Data are represented as mean ± SD unless otherwise indicated. See also Figures S6 and S15; Table S4. QQ-plot lambda values for serum biochemical trait GWAS, related to Figure 2, Table S5. GWAS results for serum biochemical parameters, related to Figure 2, Table S6. Vertebrate species used for BCHE conservation analysis, related to Figure 2, Table S7. Body-size traits and BCHE genotype frequencies, related to Figure 2, Table S8. Serum lipidomic profiles, related to Figure 2, Table S9. Variation and heritability of serum metabolites, related to Figure 2, Table S10. Lipids associated with BCHE genotype variation, related to Figure 2. Because the C allele is the main allele of interest in this study, genotypes are displayed as CC, CG, and GG in genotype-comparison panels.

To investigate the genetic basis of serum biochemical variation, we conducted a *meta*-GWAS on 21 serum biochemistry traits in 298 horses. Ten traits exhibited well-calibrated QQ plots, with no significant inflation, indicating robust association signatures (Table S4). These traits included BChE, AST, alkaline phosphatase (ALP), glucose (GLU), as well as pyruvic acid (PYR), HBDH, angiotensin-converting enzyme (ACE), TG, CHOL, and LDL-c concentrations (Figure 2A).

Overall, a total of 50 genomic loci encompassing 110 SNPs exceeded the genome-wide significance threshold (2.82e−9; Table S5). The genomic windows supported by at least three consecutive significant SNPs included nine annotated genes (Figure 2A). Among these, *PRLR*, *AGXT2*, and *SEMA5A* were associated with AST, *KLHL7* with LDL-c, *ADAMTS17*, *THSD7B*, and GSE1 with TG, while *LHFPL4* and *BCHE* were associated with BChE. The most significant association was identified on chromosome 19, within the *BCHE* gene, for BChE activity (*p* value = 1.02e−13) (Figure 2A). *BCHE* encodes the BChE, a multifunctional enzyme with key protein domains including an acetylcholinesterase tetramerization domain (AChE_tetra), a carboxylesterase domain (COesterase), and an α/β hydrolase fold (Abhydrolase_3) (Figure S6A). This enzyme displays diverse biological functions, such as detoxifying nerve agents,^35^^,^^36^ hydrolyzing cocaine,^37^^,^^38^ and is involved in research on Alzheimer’s disease,^39^^,^^40^ emotional disorders,^41^ and lipid metabolism.^40^ Given its strong association and functional relevance, *BCHE* was prioritized as the most promising candidate gene for further validation.

### Fine-mapping of a functional missense variant in *BCHE*

To pinpoint the causal variant underlying the *BCHE* association signal, we conducted fine-mapping of the candidate region. A strong linkage disequilibrium (LD) block was observed within the *BCHE* gene on chromosome 19 (chr19:9,197,000–9,205,000), with three SNPs (ECA19:9,198,989, ECA19:9,199,96, and ECA19:9,200,240) exhibiting high pairwise LD (R^2^ > 0.8; Figure 2B). Among these, ECA19:9,198,989 (G/C) consists of a missense mutation resulting in an amino acid substitution from asparagine (N) to lysine (K). The “G” allele at this position is associated with a ∼20% reduction in circulating BChE activity (*p* value = 1.55e−11) (Figure 2C). Linear modeling indicated that the effect of the *BCHE* missense variant on BChE activity remained highly significant after adjustment for sex, breed, and population structure (as represented by the first three principal components) (β = 1.04, *p* value = 6.54 × 10^−8^) (Figure S7). No significant variant × sex interaction was detected (*p* value = 0.377) (Figure S7). Overall, the *BCHE* missense variant could explain ∼9.5% of the phenotypic variance in BChE activity. Comparative sequence alignment revealed that the region surrounding the asparagine (N) or lysine (K) residue is highly conserved, with approximately 90% sequence similarity across 60 vertebrate species (Figure S6B; Table S6), underscoring its functional importance.

Interestingly, while the lysine (K) residue is ancestral and widely conserved across mammals, the horse reference genome, which derives from a Thoroughbred individual,^42^ carry the derived “G” allele, encoding an asparagine (N) residue. Structural modeling using TrRosetta^43^^,^^44^^,^^45^ predicted significant conformational differences, with the *BCHE* protein containing the K residue exhibiting greater predicted structural stability (Figures S8 and S9).

Previous studies have shown that the BChE enzyme modulates ghrelin concentration by cleaving its octanoyl group, leading to change in appetite and impacting fat metabolism and deposition.^46^^,^^47^ To explore this functional role in horses, we contrasted circulating ghrelin levels in blood across *BCHE* genotypes. Horses with the GG genotype (low BChE activity) showed significantly elevated ghrelin levels compared with other genotypes (*p* value = 9.67e−9), a trend consistent across multiple breeds (Figures 2D and S10). Combined, these results pointed to ECA19:9,198,989 as a strong candidate causal variant influencing both lipid metabolism and energy regulation in horses.

### BChE activity and lipid-associated traits

Previous studies in humans have shown that BChE activity is associated with lower weight and body mass index (BMI).^48^ To explore the physiological effects of the G > C *BCHE* K > N missense variant (ECA19:9,198,989) in horses, we explored the presence of possible correlations between its frequency and body conformation traits (Table S7). Regardless of sex, the allele “G” was significantly and positively corrected with BMI, body weight, height, length, and chest circumference (r^2^ = 0.2867–0.4824, *p* values < 0.05) (Figures 2E and S11). These findings are consistent with previous observations in mice^49^^,^^50^ and humans,^48^ suggesting a conserved functional role for *BCHE* in regulating body size and energy storage.

Oil red O staining of liver sections further revealed that horses homozygous for the low-activity BChE allele (“GG”-genotype, encoding for the N *BChE* residue) accumulated more lipid droplets than those homozygous for the high-activity allele (“CC”-genotype, encoding for the K *BChE* residue) (Figures 2F and S12). This suggested a role for BChE in hepatic lipid metabolism in horses.

To investigate the impact of BChE on systemic lipid metabolism, we performed lipidomic profiling on serum samples from 214 horses, including 64 CC-genotype, 102 CG-genotype, and 48 GG-genotype individuals. A total of 1,515 lipid species were identified and quantified (Table S8). These lipids were classified into six major categories: glycerophospholipids (GP, 53.5%), glycerolipids (GL, 24.4%), sphingolipids (SP, 14.0%), fatty acyls (FA, 5.7%), sterol lipids (ST, 2.3%), and prenol lipids (PR, 0.1%), and further subdivided into 21 subclasses (Figure S12). We next calculated the coefficients of variation (CVs) and narrow-sense heritability (h^2^) for all 1,515 lipids, leveraging associated whole-genome sequence data (Table S9). More than 65.5% lipids had CVs greater than 50%, while 77.0% showed h^2^ above 0.5, indicating substantial individual variability and a strong genetic component in lipid regulation.

Orthogonal partial least square-discriminant analysis (OPLS-DA) revealed a clear separation between the lipidomic profiles of GG- and CC-genotype carriers (Figure 2G), while hierarchical clustering showed that the lipidomic profiles of CG heterozygous carriers were more similar to those of the CC-homozygous carriers than to GG-homozygous carriers (Figure 2H). These patterns suggest that the *BCHE* missense variant exerts a significant influence on lipid composition in horse serum.

Univariate analysis identified 93 lipid species that differed significantly between BCHE genotypes (*p* value < 0.05, VIP > 1 and |fold change| ≥ 1.2) (Table S10). In GG-genotype horses, triacylglycerol (TG), phosphatidylcholine (PC), phosphatidylethanolamine (PE), phosphatidylserine (PS), and acylcarnitine (Cx:y) levels were significantly elevated, while ceramides (CER) and hexosylceramides (HexCer) were significantly reduced (*p* values < 0.05) (Figure S14). Specific lipid species, such as PC (18:0_24:5), PC (O-16:1_22:6), PE (19:2_22:5), TG (16:0_20:0_22:6), and PE (P-17:0_22: 6), were significantly less abundant in CC-homozygous horses than in the other two genotype groups (Figure S14).

Least absolute shrinkage and selection operator (LASSO) regression analysis of the 93 differential lipids selected 15 candidate lipids with non-zero coefficients (Figure S15A). Subsequent Spearman correlation analysis highlighted six lipids (HexCer (d19:1/23:0), Cer (t17:2/35:1), Cer (t17:2/35:0), PC (18:0_22:5), PG (21:1_18:2), and Carnitine C6:0) that were strongly associated with BChE activity (Figures S15B–S15H), representing robust candidate biomarkers with potential biological relevance.

Collectively, our results suggest that *BCHE* genetic variation is associated with changes in BChE activity, leading to alterations in lipid composition and accumulation, especially marked for the six candidate biomarkers. This highlights the importance of BChE in lipid metabolism in horses.

### Functional validation of the *BCHE* causative variant

To further elucidate the functional role of BChE in lipid metabolism, we carried out a series of experiments using both cell lines and a mouse model. We first constructed Flag-tagged expression plasmids carrying either the “C” (encoding for the N residue) or “G” (encoding for the K residue) allele at the equine BCHE missense variant (ECA19:9,198,989) (Figure S16; STAR Methods), and transfected them individually into HEK293T and HepG2 cells.

**Figure 3:**
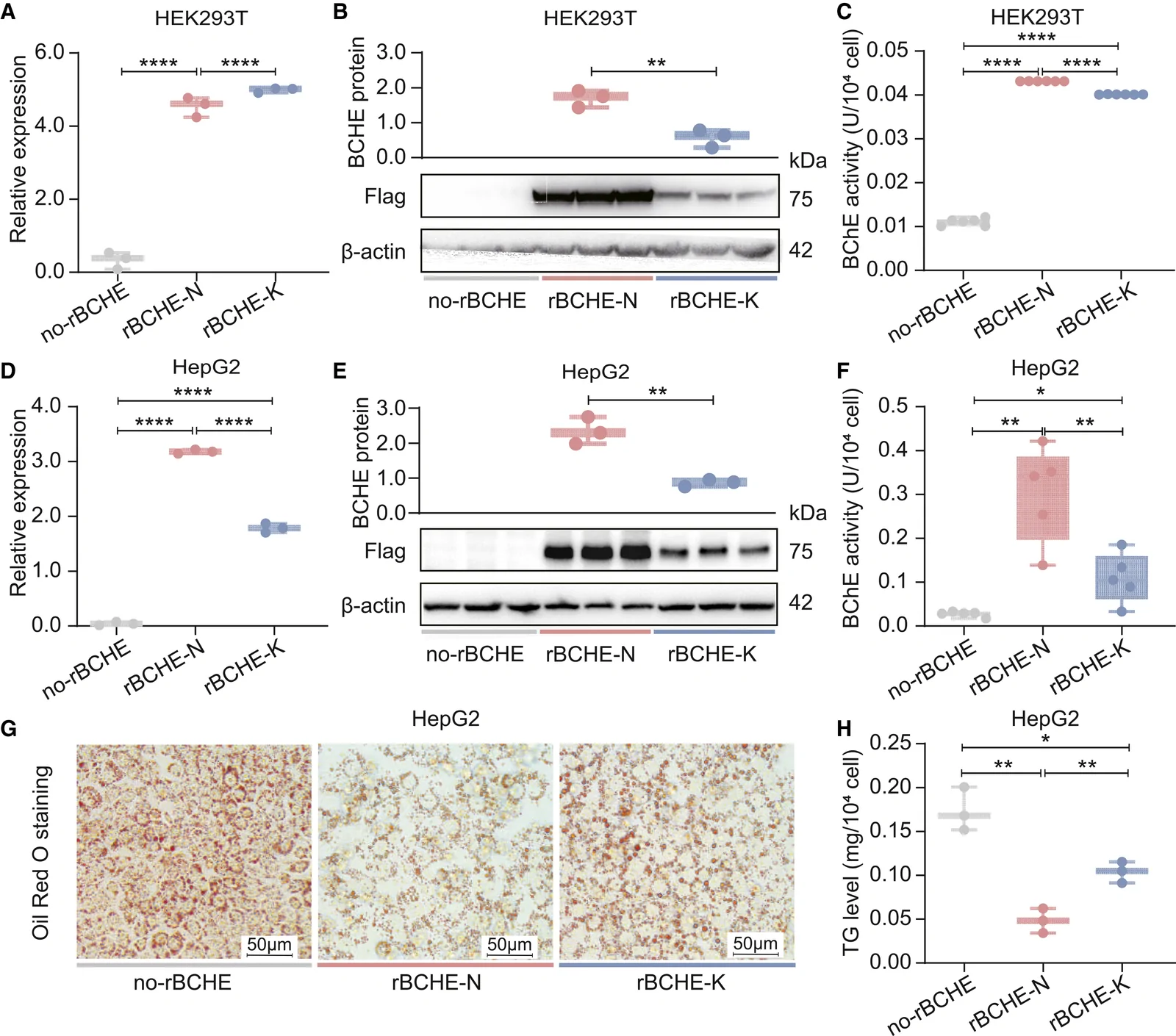
Overexpression of BCHE gene promotes lipolysis in HEK293T and HepG2 cells (A) Over-expression of BCHE mRNA in HEK293T cells. Vector indicates the control expression level relative to GAPDH expression, while C and G reflect the fold expression increase in the presence of vectors carrying the two BCHE gene variants identified at ECA19:9,198,989. (B) Western blot analysis and ImageJ quantification of BCHE protein levels. The levels of recombinant proteins were measured using anti-Flag antibodies, and normalized relative to β-actin levels and BCHE transcription levels. (C) Normalized enzymatic activity of recombinant BChE in HEK293T cells. Activity was normalized by the amount of recombinant BChE. (D) Same as (A) in HepG2 cells. (E) Quantification of recombinant BCHE protein levels in HepG2 cells. (F) Same as (C) in HepG2 cells. (G) Oil red O staining after adipogenic induction of HepG2 cells transfected with recombinant vectors. Scale bars, 50 μm. (H) Triglyceride (TG) levels in HepG2 cells transfected with recombinant vectors. Data are represented as mean ± SD unless otherwise indicated. Statistical significance was assessed using Kruskal-Wallis tests (^∗^*p* value < 0.05; ^∗∗^*p* value < 0.01; ^∗∗∗^*p* value < 0.001; ^∗∗∗∗^*p* value < 0.0001). See also Figures S16–S19. Because the C allele encodes the BChE-N form, recombinant BChE groups are displayed as no-rBChE, rBChE-N, and rBChE-K.

Western blot analyses revealed that the “G” allele resulted in significantly reduced BChE protein levels (66.59% in HEK293T, and 36.70% in HepG2), compared with the “C” allele (*p* values < 0.05) (Figures 3A, 3B, 3D, and 3E). BChE enzymatic activity was also markedly reduced: cells transfected with the Flag-tagged expression plasmids carrying the “G” allele showed an 11.05% (HEK293T) and 78.60% (HepG2) reduction in BChE activity relative to those transfected with plasmids carrying the “C” allele only (*p* values < 0.01, Figures 3C–3F). These results confirm that the “G” allele leads to both decreased protein levels and impaired enzymatic function.

Given the established association between BChE and lipid metabolism, we next induced lipid accumulation in HepG2 cells and performed Oil Red O staining to quantify lipid droplets. Cells expressing the “G” allele (K residue) exhibited a denser accumulation of lipid droplets compared with those expressing the “C” allele (N residue), although still less than the empty vector control (Figure 3G). Consistently, intracellular TG levels, a direct indicator of the cellular lipid content, were significantly higher in HepG2 cells expressing the “G” allele than in those expressing the “C” allele (*p* values = 6.50e−3), but lower than in the control (*p* values = 0.01) Figure 3H). Increased levels of lipid droplet-associated protein *PLIN1* in cells expressing the “G” allele further supported lipid accumulation (Figure S17).

We then assessed the expression of genes involved in lipid anabolism and catabolism. Cells expressing the “C” allele displayed significantly lower expression of the *de novo* lipidogenesis genes *ACLY* and *FASN* (*p* value < 0.05) (Figures S18A and S18B), suggesting that higher BChE activity reduces, or suppresses, lipogenesis. However, expression of key lipolytic enzymes, such as adipose triglyceride lipase (*ATGL*), hormone-sensitive lipase (*HSL*), and monoacylglycerol lipase *(MGL*), was not significantly altered in any group (Figures S18C and S18D). Despite this, co-localization of BChE with these enzymes was observed (Figure S19A), and structural similarity analyses indicated that BChE shares conserved domains with classical lipases (Figure S19B). This suggests that BChE may act in concert with canonical lipolytic enzymes to promote lipid mobilization.

The transfection assays above demonstrate that the *BCHE* missense variant compromises BChE protein levels and enzymatic activity, thereby impairing its lipid-hydrolyzing capacity and promoting lipid accumulation. To validate these findings *in vivo*, we generated recombinant AAV9 vectors designed to express the “C” or “G” allele in the liver, and administered them via tail vein injection to 8-week C57BL/6J mice (1.0 × 10^13^ vg/mouse). Empty vectors were injected as controls. Mice were then monitored weekly for food intake and body weight until 28 weeks of age, and showed no significant differences among the groups (Figure S20).

**Figure 4:**
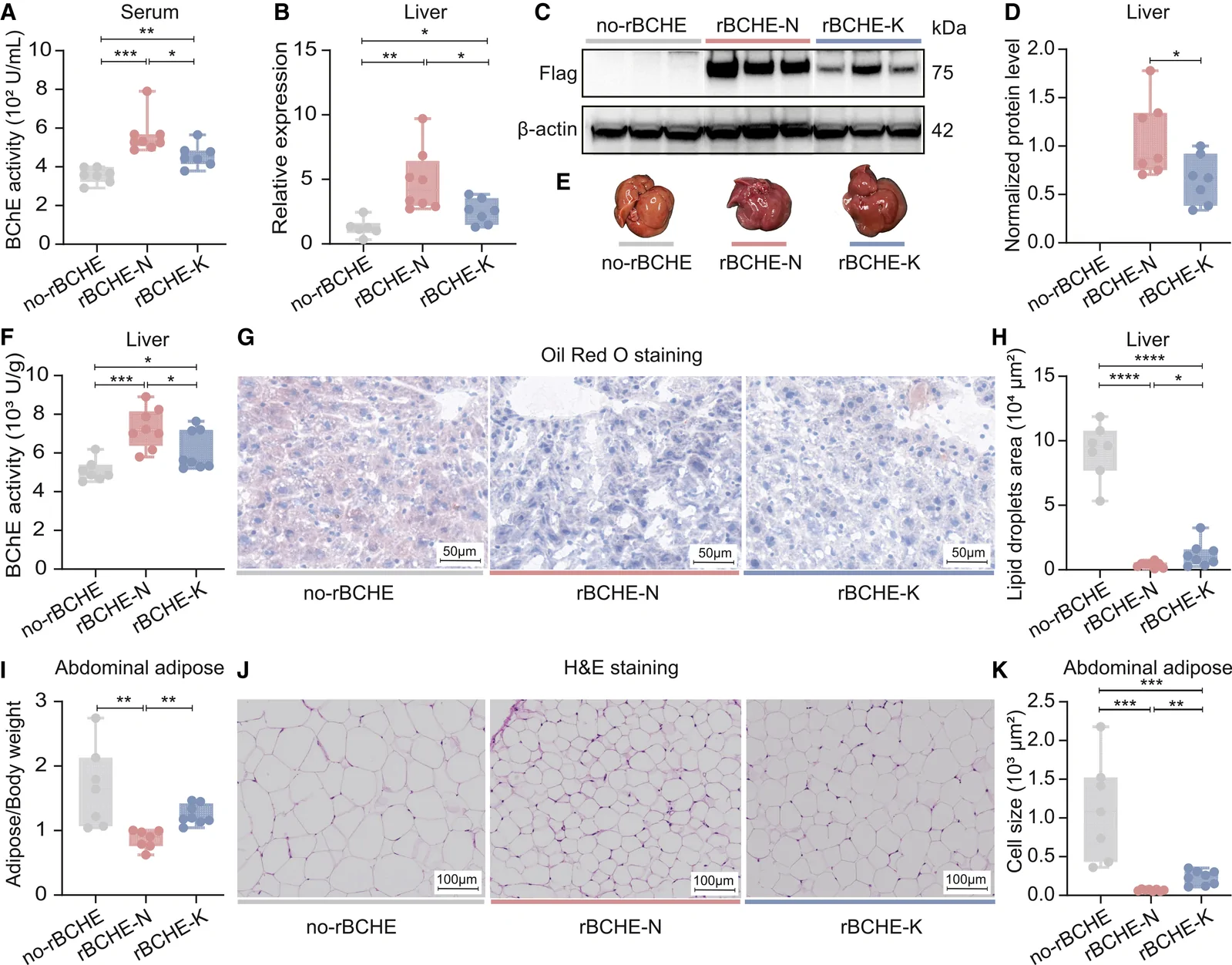
Overexpression of BCHE gene promotes lipolysis in mice (A) Serum BChE activity (10^2^ U/mL) in mice injected by three different recombinant vectors (G and C vectors carry the two BCHE gene variants identified at ECA19:9,198,989, while ‘vector’ indicates empty, control vectors. (B) Over-expression of *BCHE* mRNA in liver. (C) Western blot analysis of BCHE protein levels in three individuals per recombinant vector. (D) Entire livers of three individuals injected by different recombinant vectors. (E) ImageJ quantification of BCHE protein levels, normalized relative to β-actin levels and BCHE transcription levels. (F) Liver BChE activity (10^2^ U/mL). (G) Oil red O staining of liver sections following injections by three different recombinant vectors. Scale bars, 50 μm. (H) Lipid droplet area (10^4^ μm^2^) measured following Oil Red O staining of mice livers. (I) Abdominal adipose content, relative to body mass. (J) H&E staining of abdominal adipose tissue. Scale bars, 100 μm. (K) Cell size (10^4^ μm^2^) of adipose tissue. Data are represented as mean ± SD unless otherwise indicated. Statistical significance was assessed using Kruskal-Wallis tests (^∗^*p* value < 0.05; ^∗∗^*p* value < 0.01; ^∗∗∗^*p* value < 0.001; ^∗∗∗∗^*p* value < 0.0001). See also Figure S20. Because the C allele encodes the BChE-N form, recombinant BChE groups are displayed as no-rBChE, rBChE-N, and rBChE-K.

Mice injected with vectors carrying the “C” allele showed significantly higher *BCHE* transcription in the liver than those injected with vectors carrying the “G” allele (*p* values < 0.05) (Figures 4A–4E; STAR Methods). Concurrently, BChE activity was significantly enhanced in both the liver (*p* value = 0.04) and serum (*p* value = 0.02) (Figures 4A–4F). Oil Red O staining of liver sections revealed the greatest lipid accumulation in the liver of control mice, followed by those injected with vectors carrying the “G” allele, and least in those injected with vectors carrying the “C” allele. These differences were also mirrored in the morphology and color of the liver (Figures 4G and 4H). Moreover, mice injected with vectors carrying the “C” allele had significantly lower fat mass (Figure 4I), and smaller adipocytes (Figures 4J and 4K) compared with those injected with vectors carrying the “G” allele or controls. These *in vivo* results corroborate our *in vitro* findings, confirming that BChE plays a critical role in lipid hydrolysis and fat mobilization. The *BCHE* missense variant (G > C, leading to a K > N amino acid substitution) impairs these functions by reducing both the enzyme levels and activity, leading to increased lipid accumulation and adiposity.

### Selection at the *BCHE* locus

Given the strong association between *BCHE* variation and lipid metabolism, we next examined the selection history of this locus during horse domestication. We first estimated the frequency of the “C” allele at position ECA19:9,198,989 in a global panel of 1,238 modern horses (Table S11). While the “C” allele appears fixed in the Przewalski’s horse lineage, it reaches a worldwide frequency of 52.79% in modern domesticated horses (DOM2), with continental estimates of 54.04% in Asia, and 48.38% outside Asia. These values, obtained from both KASP genotyping (*N* = 541) and genotyping-by-sequencing (*N* = 736), were consistent with frequencies deduced from a previously published panel of horse genomes^28^ (Figure S21; Table S12).

**Figure 5:**
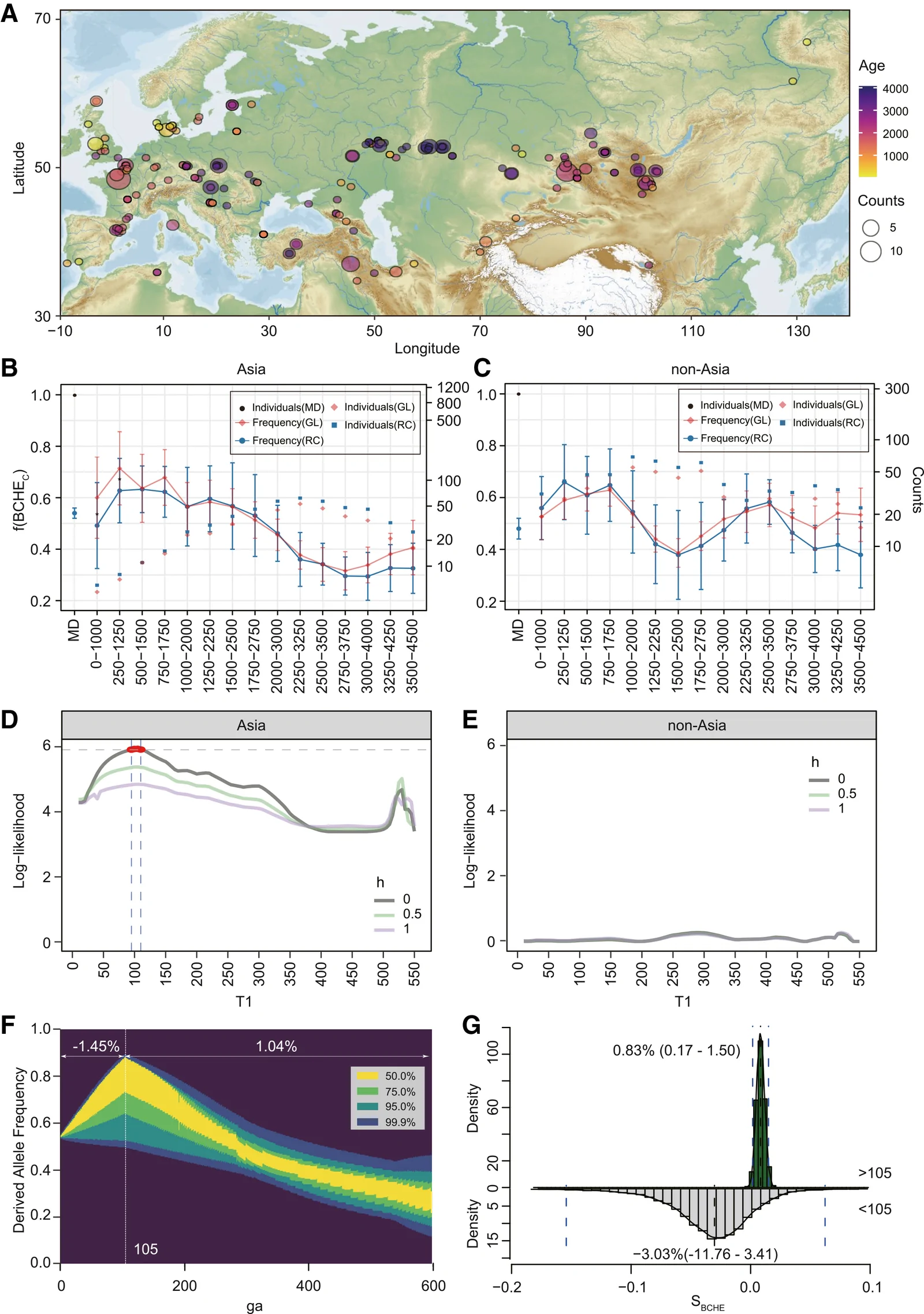
Selection history of the BCHE “C” allele (A) Map of ancient horses analyzed in this study. The color gradient reflects time (radiocarbon dates or archaeological contexts). (B) Temporal allele trajectory within Asia. (C) Temporal allele trajectory outside Asia. Allele frequencies are estimated using read counts (RC), or genotype likelihoods (GL), from the corresponding numbers of individuals showing sufficient sequence coverage. The left vertical axis represents allele frequencies, while the right vertical axis indicates the number of individuals with non-null sequence coverage. (D) CLUES2 Log Likelihood support for the selection model relative to neutrality in Asia. Analyses were performed using a grid for the time of selection shift, considering every 5 generations from 5 to 600 generations ago. (E) Same as (D), conditioning on the horses from outside Asia. (F) CLUES2 best-model fit for the temporal allele trajectory within Asia. (G) PMMH-based posterior distribution for the selection coefficients before and after 105 generations ago. See also Figures S21–S24 and Table S11. Modern horse populations used for validation of ECA19:9,198,989, related to Figure 5, Table S12. Modern frequencies of the BCHE “C” allele, related to Figure 5, Table S13. CLUES2 selection analyses for the BCHE “C” allele, related to Figure 5, Table S14. PMMH estimates for the BCHE “C” allele, related to Figure 5.

To gain understanding of frequency shifts over time, we analyzed an extensive genome time-series including 311 ancient horse specimens for this locus (Figures 5A–5C), and modeled temporal trajectories of the “C” allele within and outside Asia using CLUES2.^51^ Outside Asia, the observed temporal frequency changes were compatible with neutral drift (−log_10_*p* value = 0.12), based on the global demographic model presented by Liu and colleagues. In contrast, neutral evolution could be rejected within Asia (−log_10_*p* value = 2.58) (Table S13). Here, the “C” allele appears to have undergone positive selection from the earliest stages of DOM2 expansion outside their domestication center and until ∼95–105 generations ago (i.e., ∼777–703 years ago or ∼1248–1322 Common Era, assuming an average generation time of 7.4 years^27^), when it shifted to neutrality.

We further validated these findings by repeating our analyses to additional demographic models: the subcontinental models presented by Liu et al.^28^ for Asian and non-Asian horses, and the global model presented by Der Sarkissian et al.^52^ for the DOM2 lineage. The results remained consistent across all models (Figures 5D–5G, S22, and S23; Table S13), providing robust evidence that positive selection was specific to Asia.

We next leveraged a complementary statistical framework based on particle marginal Metropolis-Hastings (PMMH),^53^ to estimate selection coefficients before and after 105 generations ago. We first assumed recessivity for the “C” allele, since CLUES2^51^ Log likelihood was maximized under a recessive model (*h* = 0). This analysis confirmed a shift from positive selection (S^−^ = 0.83%, 95% confidence interval [CI] = 0.17%–1.50%) to neutrality in Asia, with a selection coefficient intercepting zero (95% CI = −11.76%–3.41%) from 105 generations ago to the present day (Figures 5D–5G, S22, and S23). The same pattern held when relaxing the recessivity assumption and allowing for any possible *h* value (S^−^ = 0.89%, 95% CI = 0.16%–1.61% vs. S^+^ 95% CI = −38.87%–2.90%), although these analyses indicated limited power to infer inheritance mode (Figure S24; Table S14).

Combined, our analyses indicate contrasting selection regimes for the *BCHE* “C” allele within and outside Asia, reflecting distinct breeding practices and goals across continents. Moreover, they support a shift in selection pressure within Asia around the mid-13th century CE, broadly coincident with the rise and expansion of the Great Mongol Empire and the Yuan dynasty, the first of China’s Late Imperial dynasties.^54^

## Discussion

Morphological development and athletic performance are closely influenced by metabolic processes, with genetic factors playing a pivotal role. Certain metabolic biomarkers have long been used to predict athletic potential in horses,^55^ and exercise-related traits, including aerobic capacity, are known to show close association with circulating metabolic and lipid profiles.^13^^,^^15^^,^^56^ Mutations in genes such as *MSTN* are already commercially applied to forecast racehorse performance.^57^ Despite this, the genetic underpinnings of metabolic regulation and the influence of lipids on organismal development and phenotypic traits remain fragmentary.

To address this knowledge gap, we collected and analyzed serum biochemistry data from representative domestic horse breeds across China. By integrating whole-genome sequencing with meta-analysis of serum biochemical parameters, we identified a G > C missense mutation (ECA19:9,198,989) in the *BCHE* gene that is significantly associated with BChE activity and body conformation traits in horses. Because body conformation is an important determinant of locomotor and performance-related traits in horses, the observed association between the *BCHE* variant and body conformation provides a basis for future studies integrating *BCHE*-associated metabolic variation with standardized performance or exercise-related phenotyping.

GWAS revealed that the top association with BChE activity was at the *BCHE* locus, with the missense variant emerging as a likely causal mutation. BChE has traditionally been recognized for hydrolyzing substrates such as acetylcholine, organophosphates, and cocaine, and is involved in the treatment of organophosphate poisoning and Alzheimer’s disease.^40^^,^^58^ More recent research in mice has suggested that BChE may regulate body weight as well as insulin secretion and sensitivity by hydrolyzing ghrelin.^41^^,^^49^ Our study expands on these findings by demonstrating the role of BChE in lipid deposition and hydrolysis in horses (Figure 3), in line with previous work in humans.^48^

The “C” allele is significantly associated with increased BChE enzymatic activity and decreased circulating levels of ghrelin and lipid content (Figure 4). Functional assays in cell lines and lipidomic analyses of serum confirmed the role of BChE in promoting lipid hydrolysis (Figure 5), with higher enzymatic activity correlating with more efficient lipid breakdown. While previous research proposed a BChE-ghrelin regulatory pathway, our findings provide evidence that BChE contributes to lipid metabolism, underscoring its broader physiological significance beyond ghrelin modulation. Remarkably, the specific activity and expression yield of equine BChE exceeds those reported in primates, pigs, birds, dogs, and mice,^59^ highlighting the potential of horse-derived BChE for translational research and therapeutic applications. These findings open new avenues for exploring equine BChE in regulation and drug development.

Interestingly, while the “C” allele evolved under neutrality in Europe following the expansion of DOM2 horses beyond their domestication center, it was subject to positive selection in Asia until the mid-13th century CE. When considered alongside its functional consequences, this pattern points to divergent breeding goals and environment contexts in the two continents. In Asia, selection favored allele carriers with increased BChE enzymatic activity and decreased circulating levels of ghrelin and lipids, potentially reflecting distinct management practices and/or dietary regimes, possibly shaped by the predominance of steppe ecosystems. These contrasting selection regimes disappeared during the mid-13th century, after which Asian horse populations evolved under neutrality. Future work, including *BCHE* genotyping from high-resolution time series, will be required to determine whether this shift was triggered by the expansion of the Great Mongolian Empire, which formed a transcontinental horse-based nomad empire across Eurasia,^54^ or by other major historical events, such as the rise of Late Imperial dynasties in China.

### Limitations of the study

This study integrates genomic, biochemical, lipidomic, cellular, mouse-model to demonstrate the regulatory role of *BCHE* in equine lipid metabolism. Several limitations, however, remain to be addressed. First, larger cohorts with standardized metadata regarding athletic performance, feeding management, diet, and living environments are required to validate the associations beyond those Chinese horse breeds investigated. Second, although our functional assays confirm that the *BCHE* missense variant modulates BChE enzymatic activity and cellular lipid accumulation, the downstream mechanisms linking BChE function and lipid metabolism regulation remain further unexplored. Third, while the BCHE locus exhibits signatures of region-specific selection in Asian horse populations, the exact adaptive phenotype under selection remains uncharacterized. Accordingly, the phenotypic divergence between Asian and non-Asian horses cannot be directly attributed to variations in lipid metabolism. Further studies incorporating dense temporal sampling and multi-dimensional data covering phenotypic traits, archaeological records, environmental conditions, and breeding management contexts are needed to disentangle the distinct selective pressures acting on the *BCHE* locus and the exact context driving the shifting selection regimes detected.

## Resource availability

### Lead contact

Further information and requests for resources and reagents should be directed to and will be fulfilled by the lead contact, Lin Jiang (jianglin@caas.cn).

### Materials availability

No new, unique reagents were generated in this study.

### Data and code availability

All whole-genome sequencing reads generated in this study have been deposited in the Sequence Read Archive (BioProject: PRJNA1074421). All other data supporting the findings of this study are available throughout the paper and its supplemental information. This paper does not report original code.

## Acknowledgments

This work was supported by the Fundamental Research Fund of CAAS (grant nos. CAAS-CSAB-202402 and Y2025YC50) and 10.13039/501100001809National Natural Science Foundation of China (grant no. 32222079). L.O. was supported by the 10.13039/501100000781European Research Council (HorsePower-101071707).

## Author contributions

L.J. and Y.Z. conceived and designed the study. Y.Z., Y.P., Y.M., and L.J. contributed horse samples and genomic data. Y.Z. measured and analyzed the serum biochemistry parameters data. Y.Z. and X.L. analyzed modern genome data. X.L. and L.O. analyzed ancient genome data. Y.Z. and X.W. performed cell expression analyses. Y.Z. and X.W. conducted metabolome data analysis. Y.Z., Y.J., and Y.Y. performed mouse feeding and validation experiments. Y.Z., X.L., L.O., and L.J. wrote the manuscript. All authors discussed the results and the manuscript.

## Declaration of interests

The authors declare no competing interests.

## STAR★Methods

### Key resources table

**Table:** 

REAGENT or RESOURCE	SOURCE	IDENTIFIER
**Biological samples**		

Debao pony (CSTR:12156.05.1311C0001000001081)	this study	PRJNA1074421
Ningqiang pony (CSTR:12156.05.1311C0001000001050)	this study	PRJNA1074421
Hequ horse (CSTR:12156.05.1311C0001000001033)	this study	PRJNA1074421
Yushu horse (CSTR:12156.05.1311C0001000001051)	this study	PRJNA1074421
Guanzhong horse (CSTR:12156.05.1311C0001000001059)	this study	PRJNA1074421
Kazakh horse (CSTR:12156.05.1311C0001000001037)	this study	PRJNA1074421
Yili horse (CSTR:12156.05.1311C0001000001053)	this study	PRJNA1074421
Yanqi horse (CSTR:12156.05.1311C0001000001036)	this study	PRJNA1074421

**Chemicals, peptides, and recombinant proteins**		

DME/F-12 medium	HyClone	Cat# SH30023.01
Fetal bovine serum (FBS)	Gibco	Cat# A5669701
Triton X-100 solution	Solarbio	Cat# T8200
TRIzol	Invitrogen	Cat# 15596018
Sodium oleate	Fila	Cat# T36390
Sodium palmitate	Fila	Cat# S161420

**Commercial assays**		

Wizard® Genomic DNA Purification Kit	Promega	Cat# A1125
Alanine Aminotransferase (ALT) Assay Kit	Maccura	Cat# CH0101201
Aspartate Aminotransferase (AST) Assay Kit	Maccura	Cat# CH0101202
Alkaline Phosphatase (ALP) Assay Kit	Maccura	Cat# CH0101203
Total Protein (TP) Assay Kit	Maccura	Cat# CH0101001
Albumin (ALB) Assay Kit	Maccura	Cat# CH0101002
Cholinesterase (ChE) Assay Kit	Maccura	Cat# CH0101217
High-Density Lipoprotein Cholesterol (HDL-C) Assay Kit	Maccura	Cat# CH0101161
Low-Density Lipoprotein Cholesterol (LDL-C) Assay Kit	Maccura	Cat# CH0101162
Triglycerides (TG) Assay Kit	Maccura	Cat# CH0101151
Total Cholesterol (Chol) Assay Kit	Maccura	Cat# CH0101152
Glucose (Glu) Assay Kit	Maccura	Cat# CH0101102
Glycosylatedserumprotein (GSP) Assay Kit	SJodax	Cat# RB40411
Creatine Kinase (CK) Assay Kit	Maccura	Cat# CH0101210
Lactate Dehydrogenase (LDH) Assay Kit	Maccura	Cat# CH0101208
α-Hydroxybutyrate Dehydrogenase (HBDH) Assay Kit	Maccura	Cat# CH0101209
Angiotensin-converting enzyme (ACE) Assay Kit	SJodax	Cat# RB50652
Myoglobin (MYO) Assay Kit	SJodax	Cat# RB50711
Pyruvicacid (PYR) Assay Kit	SJodax	Cat# RC40901
Calcium (Ca^2+^ Assay Kit	Maccura	Cat# CH0101251
Horse ghrelin ELISA assay Kit	Enzyme-linked	N/A
Lipofectamine 3000 Reagent Kit	Invitrogen	Cat# L3000015
Triglycerides (TG) ELISA Assay Kit	Solarbio	Cat# BC0625
Taq Pro Universal SYBR qPCR Master Mix	Vazyme	Cat# Q712-03
HiScript III All-in-one RT SuperMix Perfect for qPCR	Vazyme	Cat# R333-01

**Recombinant DNA**		

pcDNA3.1-3^∗^flag (no-rBCHE)	This study	N/A
pcDNA3.1-3^∗^flag-BCHE-N (rBCHE-N)	This study	N/A
pcDNA3.1-3^∗^flag-BCHE-K (rBCHE-K)	This study	N/A
rAAV9-3^∗^flag (no-rBCHE)	This study	N/A
rAAV9-3^∗^flag-BCHE-N (rBCHE-N)	This study	N/A
rAAV9-3^∗^flag-BCHE-N (rBCHE-K)	This study	N/A

**Antibodies**		

Anti-DDDDK tag (Binds to FLAG® tag sequence) antibody [EPR20018-251]	Abcam	Cat# ab205606; RRID:AB_2916341
anti-β-actin antibody [AC-15]	Abcam	Cat# Ab6276; RRID:AB_2223210
Alexa Fluor 488 Goat anti-Mouse IgG (H + L)	Invitrogen	Cat# A28175; RRID: AB_2536161
Cy3-conjugated antibodies	Proteintech	Cat# SA00009-1; RRID: AB_2814746

**Deposited data**		

Raw and analyzed data	This study	NCBI BioProject: PRJNA1074421

**Experimental models: Cell lines**		

Human: human hepatocellular carcinoma: HepG2	Peking Union Medical College Hospital	RRID: 1101HUM-PUMC000035
Human: human embryonic kidney 293T cell: HEK293T	Peking Union Medical College Hospital	RRID: 3101HUMSCSP5293

**Experimental models: Organisms/strains**		

C57BL/6J mice	Sibeifu	N/A

**Software and algorithms**		

Burrows-Wheeler Algorithm (BWA)	^44^	http://bio-bwa.sourceforge.net/
Genome Analysis Toolkit (GATK)	^45^	https://gatk.broadinstitute.org/hc/en-us
VCFtools	^46^	http://vcftools.sourceforge.net
PLINK	^47^	https://www.cog-genomics.org/plink2
GCTA	^48^	http://cnsgenomics.com/software/gcta/mlmassoc.html
ADMIXTURE	^49^	http://dalexander.github.io/admixture/index.html
Efficient Mixed Model Association eXpedited method (EMMAX)	^51^	https://genome.sph.umich.edu/wiki/EMMAX
CLUES2	^36^	https://github.com/avaughn271/CLUES2
PMMH	^38^	https://github.com/zhangyi-he/WFM-1L-DiffusApprox-PMMH

### Experimental model and study participant details

#### Horse samples

We collected peripheral blood samples from 298 horses representing eight populations, including Debao pony (DeBa, *N* = 30), Ningqiang pony (NiQi, *N* = 49), Hequ horse (HeQu, *N* = 75), Yushu horse (YuSh, *N* = 9), Guanzhong horse (GuZh, *N* = 64), Kazakh horse (HSK, *N* = 29), Yili horse (YiLi, *N* = 32), and Yanqi horse (YaQi, *N* = 10) (Table S1). Samples were collected from eight geographically distinct farms or pastures. Adult horses were preferentially sampled, although exact age records were not consistently available. Sex was considered during sampling, but ratios were not strictly balanced across populations (Tables S2; S3). Under veterinary guidance, only clinically healthy individuals without obvious signs of disease were included, although fitness or training status was not directly quantified. The populations were grouped into four regional categories: southern China (SC; DeBa and NiQi), Qinghai–Tibetan (QT; HeQu and YuSh), central China (CC; GuZh), and northern China (NC; YiLi, YaQi, and HSK). These populations differ in breeding background, body type, adaptive characteristics, management system, and environmental conditions, including altitude and climate (Table S1). Such factors may contribute to inter-population variation, particularly in metabolic analyses, and were therefore considered in the interpretation of the results.

Blood samples were collected from horses under the supervision of a licensed equine veterinarian. The jugular vein was punctured using a sterile blood collection needle, and approximately 10 mL of blood was drawn into EDTA and heparinized tubes. Samples were immediately placed on ice and transported to the laboratory for processing. Serum was separated by centrifugation at 1200× g for 5 min and stored at −80°C until further analysis. All procedures involving horse sampling were conducted in strict accordance with institutional and national ethical guidelines. Ethical approval for this study was granted by the Animal Welfare and Ethics Committee of the Institute of Animal Science, Chinese Academy of Agricultural Sciences, Beijing, China, under protocol number IAS2023-92.

#### Mice

C57BL/6J mice (8 weeks old) were purchased from Sibeifu (Beijing) Biotechnology Co., Ltd., and housed at the Laboratory Animal Center of the Institute of Animal Sciences, Chinese Academy of Agricultural Sciences, Beijing, China. All mice were male and were housed under a 12-h light/dark cycle with free access to food and water. All mouse experiments were conducted in strict accordance with institutional and national ethical guidelines. Ethical approval for the mouse experiments was granted by the Ethics Committee for Animal Experimentation of the Institute of Animal Science, Chinese Academy of Agricultural Sciences, Beijing, China, under protocol number IAS2024-171.

#### Cell lines

Human embryonic kidney 293T (HEK293T) cells and human hepatocellular carcinoma (HepG2) cells were purchased from the Peking Union Medical College Hospital. Cell-line authentication and mycoplasma testing were performed by the provider. The cell lines were not independently authenticated or tested for mycoplasma contamination in this study. Both cell lines were cultured in Dulbecco’s Modified Eagle Medium Nutrient Mixture F-12 (DME/F-12; HyClone, USA) supplemented with 10% certified fetal bovine serum (Gibco, USA) and penicillin (100 U/mL)/streptomycin (100 μg/mL) (Gibco, USA) at 37°C and 5% CO_2_.

### Methods

#### Whole-genome sequencing

Genomic DNA was extracted from the blood using the standard Promega extraction protocol (Promega, A1125, America). DNA quality and integrity were assessed via A260/A280 ratio and agarose gel electrophoresis. Library preparation and whole-genome sequencing were conducted by BGI Genomics (Shenzhen, China). Briefly, qualified genomic DNA was fragmented, end-repaired, A-tailed, and ligated with indexed adapters to construct paired-end sequencing libraries. These libraries were then sequenced on the DNBSEQ-T7 platform to generate 150 bp paired-end reads. The raw sequencing data processing, including base calling and quality control, was performed using BGI’s in-house bioinformatics pipelines. Clean sequence reads were aligned to the EquCab3 horse reference genome^42^ using Burrows-Wheeler Algorithm (BWA-mem algorithm),^60^ with default parameters. PCR duplicates were removed using the MarkDuplicates function in Picard (picard-tools-1.56, http://picard.sourceforge.net), and local realignment around indels was performed by Genome Analysis Toolkit (GATK).^61^ Sequencing achieved an average depth-of-coverage of 10.62-fold per individual, yielding a total of 19,878,270 high-confidence SNPs (Table S2). SNP calling was first performed in each individual using GATK HaplotypeCaller (version 4.0), followed by joint genotyping with GenotypeGVCFs applying default settings. SNPs were filtered by retaining only those with a minor allele frequency (MAF) > 0.01, genotype call rate >90%, missing genotype rate <10%, biallelic status, and Hardy-Weinberg equilibrium (HWE) *p*-value > 1e−5 using VCFTools.^62^

#### Population structure analysis

Linkage disequilibrium (LD) pruning was performed in PLINK v1.9,^63^ using sliding windows of 50 SNPs, a step size of 10, and a pairwise r^2^ threshold of 0.2 (--indep-pairwise 1000 10 0.2), resulting in 3,513,517 independent SNPs for downstream analyses. Principal component analysis (PCA) was carried out using GCTA v1.91,^64^ and visualization of the first three principal components, which combined, explained 24.6% of the total variance, was conducted in R. Individual ancestry was inferred using ADMIXTURE v1.3.0^65^ with 10,000 bootstrap pseudo-replicates, exploring K values ranging from 2 to 5. A Neighbor-Joining (NJ) tree was constructed in MEGA-X v10.1.8 based on the pairwise genetic distance matrix computed by PLINKv1.9^63^ (1-ibs).

#### Runs of homozygosity and LD-decay

Runs of homozygosity (ROH) were identified for each breed or population using the --homozyg function implemented in PLINK v1.9,^63^ based on the unpruned matrix of high-quality SNPs (*N* = 19,878,270). The main analysis was conducted using the following parameters^30^: --homozyg-window-snp 50, --homozyg-window-het 1, --homozyg-snp 10, --homozyg-kb 100, --homozyg-density 10, and --homozyg-gap 100. These settings were selected for the high-density whole-genome SNP dataset to retain sensitivity for relatively short ROH while limiting artificial extension caused by sparse marker density or excessive merging of adjacent homozygous tracts. In general, lower minimum SNP-number or ROH-length thresholds increase the detection of short ROH, whereas larger inter-SNP gap allowances or more permissive heterozygosity settings may inflate ROH length estimates by merging neighboring homozygous segments. To assess parameter sensitivity, we further compared the main parameter set with two previously published ROH parameter settings. Although the absolute estimates of total ROH length, mean ROH length, and f_ROH_ varied among parameter sets, the major population-level patterns remained broadly consistent.

#### Serum biochemistry parameters

A total of 21 baseline serum biochemistry parameters were quantified, including alanine amino transferase (ALT), aspartate amino transferase (AST), AST/ALT, total protein (TP), globulin ratio (A/G), albumin (ALB), butyrylcholinesterase (BChE), alkaline phosphatase (ALP), glucose (GLU), pyruvicacid (PYR), glycosylated serum protein (GSP), creatine kinase (CK), lactate dehydrogenase (LDH), α-hydroxybutyrate dehydrogenase (HBDH), myoglobin (MYO), angiotensin-converting enzyme (ACE), calcium (Ca), triglyceride (TG), cholesterol (CHOL), high-density lipoprotein cholesterol (HDL-c), low-density lipoprotein cholesterol (LDL-c) (Table S3). All measurements were performed using an automatic analyser (Hitachi 7080, Japan) in accordance with the manufacturer’s protocols. Assay kits were sourced from Maccura Biotech Co., Ltd.

#### Principal component analysis (PCA) and correlation analysis of serum biochemical parameters

To account for age-related effects on serum biochemical parameters, we applied a linear regression model defined as: y = β_0_ + β_1_x + ϵ, where β_0_ is the intercept, β_1_ represents the change in the indicator per unit increase in age (x), and ϵ is the residual error term. Principal component analysis (PCA) was carried out in R to identify major trends and sources of variation within the biochemical dataset. Contribution plots and PCA score distributions were generated to visualize the principal parameters contributing to principal components. Correlation analysis was performed using the cor() function in R on a complete dataset (i.e., no missing values), with Pearson’s correlation coefficient (r^2^) quantifying pairwise associations between traits. The ggplot2 package was used for data visualization.

#### Genome-wide association study

We carried out genome-wide association studies (GWASs) for 21 serum biochemistry traits across 298 Chinese horse samples using the Efficient Mixed Model Association eXpedited method (EMMAX).^66^ To control for sex and population structure, sex and the first three principal components derived from whole genome SNP data were included as fixed covariates. To account for cryptic relatedness and polygenic background, a kinship matrix was incorporated as a random effect. This matrix was specifically generated as an Identity-by-State (IBS) matrix using the emmax-kin software. The calculation employed the recommended HEreg method (-h flag) to standardize the IBS values based on expectations under Hardy-Weinberg Equilibrium, followed by scaling to achieve a mean of zero (-s flag). This optimized approach yields a robust IBS-based genetic relationship matrix that most effectively captures the realized genetic similarities among all pairs of individuals for inclusion in the mixed model. Genome-wide significance thresholds were determined using Bonferroni, setting a cutoff at 2.82e−9 (0.05/total SNPs). Linkage disequilibrium (LD) analysis was conducted using PLINK v1.9,^63^ and LD blocks within 6 kb were visualized using LDBlockShow v1.35.^67^

#### Linear modeling of BCHE variant effects

To further evaluate the effect of the *BCHE* variant on BChE activity, we performed additional linear-model analyses in R. Individuals with missing values for BChE activity, genotype, or sex were excluded. The three *BCHE* genotypes at ECA19:9,198,989 (G/C) were coded additively as 0, 1, and 2, according to the number of “C” alleles. Models included sex, breed, and the first three principal components as covariates. The main-effect model was specified as BChE ∼ Genotype + Sex + Breed + PC1 + PC2 + PC3, and the interaction model as BChE ∼ Genotype × Sex + Breed + PC1 + PC2 + PC3. The variant × sex interaction was assessed by comparing models with and without the interaction term. The proportion of phenotypic variance explained by the *BCHE* locus was estimated as the increase in model R^2^ after adding the SNP term to a baseline model including sex, breed, and the first three principal components.

#### Serum ghrelin

To assess basal ghrelin levels, serum was collected from 134 individuals selected from the original cohort of 298 horses across five horse breeds: Hequ horse (HeQu, *N* = 22), Yushu horse (YuSh, *N* = 21), Guanzhong horse (GuZh, *N* = 31), Kazakh horse (HSK, *N* = 23), and Yanqi horse (YaQi, *N* = 37). This subset was not designed to enforce exact proportional sampling across breeds or strict matching of sex composition, but to retain the main ecological and management backgrounds represented in the original cohort, including predominantly grazing populations, one stall-fed population, and populations from different altitude regions. Ghrelin concentration was quantified using a spectrophotometric ELISA assay (Enzyme-linked Biotechnology, China), following the standardized manufacturer’s protocol to ensure consistency and reliability. Absorbance was measured at 450 nm with a Tecan Infinite 200 Pro MultiMode Microplate Reader.

#### Serum lipid profiling

We profiled the lipid composition of horse serum samples using liquid chromatography-tandem mass spectrometry (LC/MS-MS), enabling comprehensive characterization of lipid metabolites. A total of 214 horses representing 10 breeds were selected and stratified by genotypes, with 64 CC homozygotes, 102 heterozygotes, and 48 GG homozygotes at ECA19:9,198,989 (G/C). The breed distribution included Debao (DeBa, *N* = 22), Ningqiang (NiQi, *N* = 32), Hequ horse (HeQu, *N* = 23), Yushu horse (YuSh, *N* = 24), Guanzhong horse (GuZh, *N* = 25), Kazakh horse (HSK, *N* = 28), and Yanqi horse (YaQi, *N* = 30). Lipidomics analysis was conducted by Metware Biotechnology Co., Ltd, using the procedures descripted as Supplementary information.

#### Coefficients of variation and heritability

Coefficients of variation (CV) for serum biochemistry parameters and metabolites were calculated as follows: CV (%) = (sd/mean)^∗^100%, where “sd” and “mean” represent the standard deviation and mean of individual serum biochemistry parameters and metabolites, respectively.

The narrow-sense heritability (h^2^) of each serum biochemical trait was estimated using a genomic-relatedness-based restricted maximum likelihood (GREML) approach in GCTA v1.90.^64^ The model incorporated a genetic relationship matrix (GRM) derived from genome-wide SNPs to estimate the additive genetic variance (σ^2^_a). Sex and the top 100 genetic principal components were included as fixed covariates. The heritability was calculated as h^2^ = σ^2^_a/(σ^2^_a + σ^2^_e), where σ^2^_e is the residual variance.

#### Least absolute shrinkage and selection operator (LASSO) regression analysis

A total of 93 differential lipids identified from pairwise comparisons were used as predictor variables, with BChE activity treated as the response variable. Prior to analysis, lipid abundances were standardized to ensure comparability across variables. LASSO regression analysis was performed using the *glmnet* R package,^68^ which implements L1-regularization to select a subset of predictive variables.^69^ 10-fold cross-validation was applied to determine the optimal regularization parameter (λ), and lipids with non-zero coefficients at the optimal λ were retained for subsequent analysis.

To further assess associations between individual lipids and BChE activity, Spearman correlation analysis was conducted. Lipids supported by both LASSO selection and Spearman correlation analysis were further visualized using correlation scatterplots.

#### Plasmid construction

All expression constructs were synthesized by Tsingke Biotechnology Co., Ltd., except for the 3×Flag-tagged pcDNA3.1 vector provided by Dr. Li.^70^ Site-specific mutations were introduced by PCR-based mutagenesis using high-fidelity DNA polymerase, followed by digestion with NheI and BamHI and subcloning into the pcDNA3.1 vector using the EasyGeno Rapid Recombinant Clone Kit (TIANGEN). To enhance expression efficiency in mammalian cells, the equine BCHE coding sequence was codon-optimized (CAI increased from 0.64 to 0.94) using GeneOptimizer, while maintaining the original amino acid sequence. Detailed procedures are provided in the Supplementary Information.

#### Cell transfection

Recombinant full-length equine BChE proteins, each tagged with a C-terminal 3×FLAG epitope, were transiently expressed in HEK293T and HepG2 cells. Cells were seeded and cultured to ∼70% confluence before transfection with 5 mg of plasmid DNA using a Lipofectamine 3000 Reagent kit (Invitrogen, USA), following the manufacturer’s instructions.

#### Western blot analysis

To confirm BChE overexpression 48-h post-transfection, HEK293T and HepG2 cell lysates were prepared using lysis buffer (Solarbio, China) with phenyl methyl sulfonyl fluoride (PMSF; Solarbio, China). Proteins were separated by SDS-PAGE on 10% sodium dodecyl sulfate-polyacrylamide gels, and transferred to polyvinylidene fluoride (PVDF) membranes (Millipore, USA). Membranes were blocked with Fast Blocking Western solution (Beyotime Biotechnology, China) for 10min, followed by overnight incubation at 4°C with rabbit anti-FLAG (1:5000; Abcam, UK) and mouse anti-β-actin (1:2000; Abcam, UK) primary antibodies. After three washes with TBST, membranes were incubated with appropriate HRP-conjugated secondary antibodies for 1h at room temperature and washed again three times. Detection was performed using enhanced chemiluminescent substrate, and images were captured using a Tanon 5200 Multi Imaging system (Tanon, China).

#### Adipogenic induction and Oil Red O staining

To evaluate the role of *BCHE* on lipid metabolism, adipogenesis was induced in HepG2 cells using a mixture of sodium oleate and sodium palmitate (v: v = 2:1) (Fila Biotechnology, China), following a standard protocol based on an adipogenic cocktail. Forty-eight hours post-transfection, sodium oleate and sodium palmitate were added to the HepG2 culture medium. After 24h of induction, cells were washed three times with PBS, fixed with 4% paraformaldehyde for 30 min at room temperature, and stained with Oil red O staining solution (Beyotime Biotechnology, China) for 30 min. Lipid droplet accumulation was visualized as orange-red inclusions under a Zeiss LSM880 confocal microscope (Zeiss, Germany).

#### Intracellular triglyceride (TG) content

Following adipogenic induction, HepG2 cells were harvested, and intracellular lipids were extracted using a 1:1 (v/v) mixture of n-heptane and isopropanol, followed by ultrasonic disruption with a Bioruptor UCD-200 (Diagenode, Belgium). Intracellular triglyceride (TG) levels were quantified using an ELISA assay kit (Solarbio, China), following the manufacturer’s instructions. Absorbance was measured at 420nm using a Tecan infinite 200 pro MultiMode Microplate Reader (Tecan, Switzerland). TG concentrations were calculated using the following formula:

TG content (mg/10^4^ cell) = $C_{\text{standard}}\times$ (A_test_−A_blank_)/(A_standard_−A_blank_)/Cell (mg/10^4^ cell), where C_standard_ = 1mg/mL.

#### Immunofluorescence (IF) staining

Given the apparent hydrolysis effect of BChE on lipid droplets, we sought to determine the subcellular localization of the enzyme in HepG2 cells, following adipogenic induction. Immunofluorescence staining was carried out to visualize BChE distribution. Treated cells were washed three times with PBS and fixed with 4% paraformaldehyde for ∼15-30min at room temperature. After fixation, cells were permeabilized with 0.25% Triton X-100 (Solarbio, China), and washed again with PBS. Blocking was carried out using 10% goat serum for 1h at room temperature. Cells were then incubated overnight at 4°C with a rabbit anti-FLAG primary antibody (1:5000; Abcam, UK) diluted in blocking buffer, followed by three PBS washes. Secondary antibody incubation was conducted for 1h at room temperature using Alexa Fluor 488 (Invitrogen, USA) and Cy3-conjugated antibodies (Proteintech, USA), diluted in PBS. Nuclei were stained with DAPI (50–100 μg/mL) (Solarbio, China) for 15 min at room temperature. Fluorescence images were captured using a Zeiss LSM880 confocal scanning microscope (Zeiss, Germany).

#### Construction of *BCHE* overexpression mouse model

To investigate BChE effects *in vivo*, we used overexpression vectors carrying the C and “G” alleles, or flag controls, to generate a type 9 recombinant adeno-associated virus (rAAV), under the control of a CMV promoter. The constructs were synthesized and packaged by HanHeng Biotechnology (Shanghai) Co., Ltd., via helper virus-free transient transfection in HEK-293 cells, with viral titers of no less than 1×10^12^ vector genomes (V.g.)/mL.

A total of 24 male C57BL/6J mice (8-weeks old) were purchased from Sibeifu (Beijing) Biotechnology Co., Ltd., and randomly subdivided into three groups. The first group (no-rBCHE) was injected with Flag-rAAV, while the second (rBCHE-N) and third (rBCHE-K) groups were injected with C-rAAV and G-rAAV vectors. Each mouse received a 100μL tail vein injection. Body weight and food intake were monitored weekly until sacrifice at week 28.

#### RT-qPCR experiments

Liver and adipose tissues were collected from rAAV-injected C57BL/6J mice at 28 weeks. Mice were euthanized under anesthesia, and tissues were excised, weighed, and either snap-frozen for RNA extraction or fixed for histological analysis. Total RNA was isolated using TRIzol (Invitrogen) and reverse-transcribed with the HiScript III All-in-one RT SuperMix Kit (Vazyme). Quantitative real-time PCR was performed using SYBR Green chemistry on an ABI Q7 Flex Real-Time PCR System (Applied Biosystems). BCHE mRNA levels were normalized to ACTB and calculated using the 2^∧^–ΔΔCT method. Detailed information on primer sequences and thermocycling parameters is provided in the Supplementary Information. Detailed procedures are provided in the Supplementary Information.

#### Histological staining

Mice were euthanized by cervical dislocation under anesthesia. The abdominal cavity was immediately opened through a midline incision, and liver and fat pads surrounding the gonads or the abdominal wall were carefully dissected, weighed, and either snap-frozen in liquid nitrogen or fixed in 4% paraformaldehyde (Solarbio, China). Fixed samples were subjected to Hematoxylin and Eosin (H&E) staining, while frozen samples were subjected to Oil Red O staining. All embedding, sectioning, and staining processes were carried out by Servicebio Biotechnology Co., Ltd.

#### Validation in the modern horse populations

We genotyped 1,238 modern horses (GP2) from 64 breeds or populations to assess the frequency of the *BCHE* SNP variant (ECA19:9,198,989, G/C) in present-day horse populations worldwide. The validation dataset included 978 Asian and 260 non-Asian individuals, and detailed information on the GP2 dataset is provided in Table S11. Genotyping was performed using two approaches: Kompetitive Allele-Specific PCR (KASP)^71^ for 525 horses and SNP calling from whole-genome sequencing data for 713 horses. KASP genotyping was carried out by China Golden Marker (Beijing) Biotech Co., Ltd. The sequencing data included those data newly generated in this study and publicly available data from previous studies (NCBI BioProject accession nos. PRJEB14779, PRJEB9267, PRJEB9269, PRJEB9799, PRJNA230019, PRJNA641243, PRJNA841639, PRJNA331853, PRJNA357976, PRJNA168142, PRJNA391840, PRJNA515160, and PRJNA780752).

#### Selection analyses

We estimated modern allele frequencies using two independent datasets. The first (GP1) included 990 modern DOM2 horses reported in,^28^ and the second (GP2) included 1,238 modern DOM2 horses sequenced by KASP^71^ and whole-genome sequencing. Horses were classified as Asia (AS) or non-Asia (non-AS) showed different genetic ancestry profiles (Figure S19). Allele frequencies and 95% HPD intervals were then estimated in R. Based on GP1, the “C” allele frequency was 0.51 (95% HPD = 0.48–0.54, *N* = 571) in AS horses and 0.48 (95% HPD = 0.44–0.51, *N* = 419) in non-AS horses. Similarly, GP2 yielded a frequency of 0.54 (95% HPD = 0.52–0.57, *N* = 987) in AS horses and 0.48 (95% HPD = 0.44–0.53, *N* = 269) in non-AS horses.

Demographic modeling was performed as described by Liu et al.^28^ Ancient DOM2 horses were further grouped into AS and non-AS categories based on their geographic distribution. Genotype probabilities were estimated using ANGSD v0.930, following the procedures outlined by Liu et al.^28^ Genotype likelihoods were log-transformed, and the age of each individual, determined from radiocarbon dating, was converted to generations assuming a generation time of 7.4 years.^27^

Both one-epoch and two-epochs selection models were applied to AS and non-AS horses. For the two-epochs model, the time point at which the selection regime shifted (T) was inferred through a grid search, testing values of T between 10 and 550 generations in steps of five, with Tend fixed at 600 generations. Three dominance parameters were considered: *h* = 0 (recessive), *h* = 0.5 (codominant), and *h* = 1 (dominant). The maximum selection coefficient (-sMax) was explored within the range of −0.75 to 0.75. The model yielding the highest log likelihood and corresponding log likelihood was taken as the most likely selection scenario for the dataset, including estimates of both T and h.

To ensure the robustness of our findings against potential demographic mis-specifications, we cross-validated the selection signals using multiple independent demographic scenarios: the subcontinental models from Liu et al.^28^ for DOM2 populations within and outside Asia, as well as the global model from Der Sarkissian et al.^52^ for the global DOM2 lineage. For each demographic scenario, we re-ran the CLUES2 grid search across the same range of selection shift times (T) and dominance parameters (h) (Table S13).

Selection coefficients were further estimated using the PMMH software.^53^ Input files were derived from genotype likelihoods generated by ANGSD, following the same procedure as in CLUES2, with T and h fixed to the best estimates obtained from CLUES2. To improve convergence, we increased the number of iterations from 20,000 to 100,000. Both PMMH v1.5 and v1.4 were run to investigate the potential impact of the inheritance mode assumed for the “C” allele.

### Quantification and statistical analysis

Statistical analyses and data visualization were performed using R v4.2.2, GraphPad Prism v8.0.2, and analysis-specific software described in the corresponding Method details subsections. Group comparisons were assessed using Kruskal-Wallis tests, and P-values <0.05 were considered statistically significant. Exact values of n are provided in the corresponding Results section, figure legends, tables, and Method details subsections. Depending on the analysis, n represents the number of horses, mice, serum samples, tissue samples, cell-culture replicates, lipid species, SNPs, or ancient genomes, as specified in the relevant section. Measures of center and dispersion or precision, including mean, SD, confidence intervals, and HPD intervals, are reported in the corresponding figure legends, figures, Results section, tables, and Method details subsections.

## Supplemental information

Document S1. Figures S1–S5

Table S1. Sample information for Chinese domestic horse populations, related to Figure 1 This table summarizes breed information, cohort characteristics, sampling regions, environmental metadata, and management systems.

Table S2. Whole-genome sequencing statistics, related to Figure 1 This table summarizes sequencing depth, mapping rate, and sample-level sequencing information.

Table S3. Serum biochemical parameters, related to Figure 1 This table provides individual-level measurements of 21 serum biochemical parameters across Chinese horse breeds.

Table S4. QQ-plot lambda values for serum biochemical trait GWAS, related to Figure 2 This table summarizes genomic inflation factors for GWAS analyses.

Table S5. GWAS results for serum biochemical parameters, related to Figure 2 This table lists significant loci and candidate genes associated with serum biochemical traits.

Table S6. Vertebrate species used for BCHE conservation analysis, related to Figure 2 This table lists taxonomic information and protein accession numbers for 60 vertebrate species.

Table S7. Body-size traits and BCHE genotype frequencies, related to Figure 2 This table summarizes body conformation measurements and genotype frequencies in Chinese horse populations.

Table S8. Serum lipidomic profiles, related to Figure 2 This table provides lipid annotations and normalized abundance values across serum samples.

Table S9. Variation and heritability of serum metabolites, related to Figure 2 This table summarizes coefficients of variation, OPLS-DA VIP values, heritability estimates, and associated statistics.

Table S10. Lipids associated with BCHE genotype variation, related to Figure 2 This table lists differential lipid species and associated statistical measures across genotype groups.

Table S11. Modern horse populations used for validation of ECA19:9,198,989, related to Figure 5 This table summarizes populations and genotype counts used for validation analyses.

Table S12. Modern frequencies of the BCHE “C” allele, related to Figure 5 This table reports allele frequencies, sample counts, confidence intervals, regions, and dataset sources.

Table S13. CLUES2 selection analyses for the BCHE “C” allele, related to Figure 5 This table summarizes selection analyses across geographic groups and demographic models.

Table S14. PMMH estimates for the BCHE “C” allele, related to Figure 5 This table reports posterior estimates of selection coefficients and confidence intervals.

Document S2. Article plus supplemental information
