# Supplementary material for: Adaptive variation in butyrylcholinesterase-influenced lipid metabolism during horse domestication
Source: Cell Rep. 2026 Aug 20;45(9):117879. doi: 10.1016/j.celrep.2026.117879 (PMC13597480; doi:10.1016/j.celrep.2026.117879)
Supplement: Supplementary file 1 [file mmc1.pdf]

Cell Reports, Volume *45*

Supplemental information

Adaptive variation in butyrylcholinesterase-influenced lipid metabolism during horse domestication

Yanli Zhang, Xuexue Liu, Xintong Wang, Yanhui Yu, Yaozhen Jia, Yabin Pu, Yuehui Ma, Ludovic Orlando, and Lin Jiang

Supplementary Methods S1: Additional methodological details supporting the STAR Methods

Conservation analysis of BCHE protein

To investigate the structural features of the equine BCHE protein, we retrieved its protein sequence from the Uniprot database (Accession Nb. F7CC84). Domain annotations provided by Uniprot were supplemented using the Simple Modular Architecture Research Tool (SMART)[1], revealing four distinct regions, including the Acetylcholinesterase tetramerization domain (AChE_tetra), the Carboxylesterase family domain (COesterase), the Abhydrolase_3 domain, and a low-complexity region. To assess the evolutionary conservation of BCHE across taxa, we downloaded BCHE protein sequences from 60 vertebrate via NCBI, comprising 34 mammals, 15 birds, four lepidosaurs, four turtles, two amphibians, and one lungfish (accession numbers are listed in Table S6. Multiple sequence alignment was performed using the MUSCLE algorithm with default parameters, followed by manual curation. A Neighbor-Joining (NJ) tree was constructed using MEGA X[2]. The evolutionary distances were computed using the Poisson correction method. All positions containing gaps and missing data were eliminated (complete deletion option). The robustness of the inferred phylogeny was evaluated by bootstrap resampling with 1000 replicates.

Prediction of BCHE protein structure and stability

To evaluate structural changes in the BChE protein resulting from the amino acid substitution identified, we used the transform-restrained Rosetta (trRosetta) server for accurate *de novo* prediction of K ("G" allele) and N ("C" allele) protein structures[3-5]. The inclusion of homologous templates improved model accuracy, with TM-scores of 0.936 (K, "G" allele), and 0.948 (N, "C" allele), indicating structural reliability. The trRosetta server is freely accessible at https://yanglab.nankai.edu.cn/trRosetta/. The resulting structural models of the two variant BCHE proteins were further evaluated for stability using the DUET web server, which integrates three computational approaches, i.e., DUET[6], mCSM[7], and SDM[8]. All predictions consistently supported differential stability between the two variants (Figures S8 and S9). The DUET web server is freely accessible at http://structure.bioc.cam.ac.uk/duet.

Lipid profiling

We profiled the lipid composition of horse serum samples using liquid chromatography-tandem mass spectrometry (LC/MS-MS), enabling comprehensive characterization of lipid metabolites.

For lipid extraction, 50 µL of serum was mixed with 1mL of extraction solvent (MTBE: MeOH =3:1, v/v) containing internal standards. The mixture was vortexed for 15 min, followed by the addition of 200 µL of water, vortexing for 1 min, and centrifugation at 12,000 rpm for 10 min. The upper organic phase (200 µL) was collected, dried using a vacuum concentrator, and resuspended in 200 µL mobile phase B prior to LC-MS/MS analysis on the LC-ESI-MS/MS platform (UPLC, ExionLC AD; MS, QTRAP® System, https://sciex.com/). Chromatic separation was achieved on a Thermo Accucore™ C30 column (2.6 µm, 2.1 mm*100 mm i.d.). The solvent system consisted of mobile phase A: acetonitrile/water (60/40, V/V, 0.1% formic acid, 10 mmol/L ammonium formate), and; mobile phase B: acetonitrile/isopropanol (10/90 V/V, with the same additives. The gradient program was as follows: A/B（80:20, V/V）at 0 min, 70:30 V/V at 2.0 min, 40:60 V/V at 4 min, 15:85 V/V at 9 min, 10:90 V/V at 14 min, 5:95 V/V at 15.5 min, 5:95 V/V at 17.3 min, 80:20 V/V at 17.3 min, 80:20 V/V at 20 min. Flow rate was set at 0.35 ml/min, column temperature at 45°C, and injection volume at 2µl.

Mass spectrometry was performed in both positive and negative ion modes using a ESI-triple quadrupole-linear ion trap (QTRAP) equipped with an ESI Turbo Ion-Spray interface controlled by Analyst v1.6.3 software (Sciex). ESI parameters were included: source temperature 500°C, ion spray voltage (IS) 5500 (positive) / -4500 V (negative), ion source gas 1 (GS1=45 psi), ion source gas 2 (GS2=55 psi), curtain gas (CUR=35 psi), and collision gas (CAD) set to medium. Instrument tuning and mass calibration were performed using 10 and 100 µmol/L polypropylene glycol solutions in QQQ and LIT modes, respectively. Multiple reaction monitoring (MRM) was used for metabolite detection, with nitrogen as the collision gas (5 psi). Declustering potential (DP) and collision energy (CE) were optimized for each MRM transition, and metabolite-specific MRM transitions were assigned on the basis of retention times.

Metabolite identification was carried out using the targeted standard Metare Database (MWDB), which integrates compound retention time (RT), parent ion pair information, and MS/MS fragmentation spectra for qualitative analysis. After raw mass spectrometry data acquisition across all samples, peak areas were extracted for each metabolite, and signal intensities were normalized

across samples to correct for inter-run variability. Multivariate statistical analyses were conducted using the Metware Cloud (https://cloud.metware.cn/). Principal component analysis (PCA) and orthogonal partial least squares-discriminant analysis (OPLS-DA) were performed to capture global variation and discriminate between groups. Model performance was assessed using the R^2X, R^2Y, and Q^2 metrics to ensure robustness and predictive reliability. Differential metabolites were defined as those with a variable importance in projection (VIP) score >1.0, an absolute|fold change (FC)| ≥1.5, and a statistical significance threshold of $P<0.05$.

Plasmid construction

Vector construction followed previously described protocols[9]. Briefly, site-specific mutations were introduced by designing complementary oligonucleotide primers spanning the target locus. PCR amplification was then performed using a high-fidelity DNA polymerase (Vazyme, China) under the following thermocycling conditions: an initial denaturation at 98°C for 30 s; followed by 18 cycles of 98°C for 10 s, 60°C for 30 s, and 72°C for 6 min; with a final extension at 72°C for 10 min. This process generated both the mutant (C) and wild-type (G) variant fragments, and the methylated parental DNA template was subsequently digested with *NheI* and *BamHI* restriction enzyme. The EasyGeno Rapid Recombinant Clone Kit (VI201, TIANGEN) was used to subclone the wild-type and the mutant sequences of the equine BCHE gene into the pcDNA3.1 vector for subsequent overexpression analysis. The native equine BCHE (horse-BCHE) sequence exhibited a suboptimal codon adaptation index (CAI) of 0.64 for expression in mammalian systems. To enhance its translational efficiency in human and murine host cells, we performed codon optimization using GeneOptimizer® software (Tsingke Biotechnology Co., Ltd.), while ensuring the encoded amino acid sequence remained unchanged (Figure S16). The optimization process incorporated preferred codon usage patterns from both Homo sapiens and Mus musculus. The optimized construct achieved a significantly improved CAI of 0.94, representing a 46.9% increase in codon adaptation relative to the wild-type sequence. This substantial enhancement in codon optimization is predicted to markedly improve BCHE protein expression levels in the target expression systems.

Detection of the recombinant BChEs activity

BChE enzymatic activity was measured using a spectrophotometric assay (Enzyme-linked

Biotechnology, China), as previously described[10; 11]. Culture media containing recombinant BChEs, collected post-transfection and adipogenic induction, were subjected to ultrasonic disruption and centrifuged at 1000 g for 10min after to remove cellular debris. For each reaction, 0.1mL of sample, standard, or ddH_2O (blank) was mixed with 0.5mL of substrate and 0.5mL of indicator, and incubated at 37°C for 6 min. Subsequently, 0.03mL inhibitor and 0.1mL clarifying agent were added to each mixture and incubated at room temperature for 15min. Absorbance was measured at 412 nm using a Tecan infinite 200 pro MultiMode Microplate Reader. Enzymatic activity (U/mL) was calculated using the formula:

$$\text{Active enzyme units (U/mL)} = \frac{A_{test} - A_{control}}{A_{standard} - A_{blank}} \times C_{standard} \times N$$

where $C_{standard}$ is the concentration of the standard, and N is the sample dilution factor.

RT-qPCR experiments

We sacrificed rAAV-injected C57BL/6J mice at 28 weeks and collected tissues including liver and adipose tissues. Mice were euthanized by cervical dislocation under anesthesia. The abdominal cavity was immediately opened through a midline incision, and abdominal adipose tissue was carefully dissected. Fat pads surrounding the gonads or the abdominal wall were excised, weighed, and either snap-frozen in liquid nitrogen for subsequent analysis or fixed in 4% paraformaldehyde (Solarbio, China) for histology. Total RNA was extracted using TRIzol (Invitrogen, USA) and the TIANGEN Reagent kit (TIANGEN, China). First-strand cDNA was synthesized by reverse-transcription using the HiScript III All-in-one RT SuperMix Perfect for qPCR Kit (Vazyme, China). Quantitative real-time PCR (qPCR) was performed on an ABI Q7 Flex Real-Time PCR System (Applied Biosystems, USA) using the Taq Pro Universal SYBR qPCR Master Mix kit (Vazyme, China). BCHE expression levels were measured through technical replicates and normalized to ACTB expression levels. qPCR conditions were as follows: Each 20 μL qPCR reaction contained 10 μL of 2X Taq Pro Universal SYBR qPCR Master Mix, 0.4 μL of each forward and reverse primer (10μM), 2 μL of cDNA template, and 7.2 μL of ddH2O. The thermal cycling conditions were as follows: initial denaturation at 95°C for 30s; followed by 40 cycles of 95°C for 10s (denaturation) and 60°C for 30s (annealing/extension). Forward and reverse PCR primers were 5'-TGCAGGTTTTGGACCCTGTTCTT and 5'-ACTACAAGGATGACGATGA, and 5'-TCTGGCACCACCTTCTACAATG and 5'-AGCACAGCCTGGATAGCAACG for BCHE

and ACTB, respectively). These PCR primers targeted PCR fragments of 188 bp and 260 bp, respectively. Gene expression fold-change was calculated via the $2^{(-\Delta\Delta CT)}$ method. For each sample, ΔCT was computed as CT_target - CT_ACTB. The ΔΔCT value was then determined using the formula ΔΔCT = ΔCT_sample -ΔCT_control, where the control group average ΔCT served as the calibrator. Results are expressed as $2^{(-\Delta\Delta CT)}$.

Validation in the modern horse populations

We genotyped 1,277 modern horses from 69 breed or populations to assess the frequency of the BCHE SNP variant (ECA19:9,198,989, G/C) across the world (Table S11). This data set included: 21 Przewalski's horses; 1097 Chinese landrace horses from Southwest China (SC=243; DeBa=57, NiQi=57, YuNa=17, WeSh=27, LiCh=25, LuPi=25, and JiCh=35), Qinghai-Tibet (QT=352; YuSh=50, HeQu=83, JiZi=16, MZH=24, NiMu=37, LKZ=21, DaTo=57, and CDM=64), Central China (CC=104, GuZh=104), North China (NC=370; JiLi=24, HSK=38, HeHe=29, YiLi=44, MoGo=60, ELC=57, YaQi=69, WuSh=27 and SeSe=22); Mongolia horse (WMG=28); Pony mix (Pony mix=59); Quarter horse (Quarter=37); Thoroughbred (ThB=72); Middle East horse (MA=20); Europe horse (EU=78); and American horse (AM=12). Breed classification followed the China National Commission of Animal Genetic Resources 2011 and Horse and Ass Breeds in China (1986). SNP calling was carried out as described follows (section Whole-genome sequencing). Genotyping of the “C” allele was performed with Graphtyper v2.7.4 using default parameters, and variants were subsequently filtered with vcffilter(Garrison et al. 2022), using the following criteria: -f 'ABHet < 0.0 | ABHet > 0.33' -f 'ABHom < 0.0 | ABHom > 0.97' -f 'MaxAASR > 0.4' -f 'MQ > 30' -t 'pass' -F 'graphtyper_filter'. The frequencies of the BCHE “C” allele estimated within and outside Asia were compared with the frequencies present in a previously published global panel of horse genomes[12], applying the same genotyping filters as above. To assess the consistency of the genotype frequencies between this global panel (GP1) and this study data (GP2), we performed a bootstrap resampling analysis. We used the breed proportions from GP1 as sampling weights to generate 10,000 bootstrap samples from GP2. The empirical distribution of allele “C” frequencies from these resamples was then compared to the observed frequency in GP2, with significance determined using 95% confidence intervals and two-sided *P*-values.

Supplementary Figures

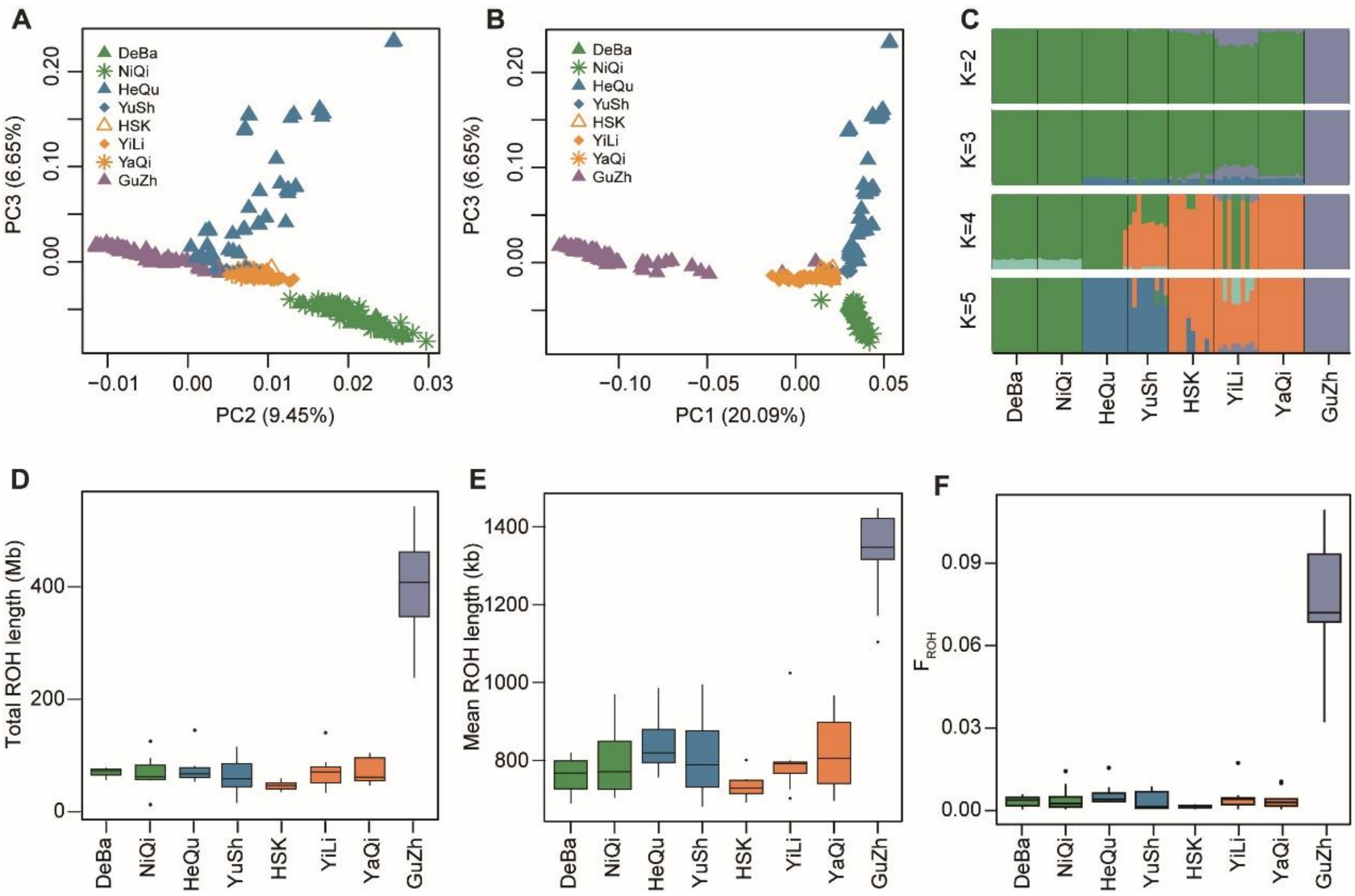

Figure S1. Population structure and length of Runs of Homozygosity (ROH), related to Figure 1.

(A) Principal Component Analysis (PCA) of autosomal SNP variation. The fractions of the total variance explained by PC2 and PC3 are reported between parentheses on each axis. DeBa=Debao, GuZh=Guanzhong, HeQu=Hequ, HSK=Kazakh, NiQi=NiQiang, YaQi=YanQi, YiLi=YiLi, YuSh=YuShu.

(B) same as **(A)**, for PC1 and PC3.

(C) ADMIXTURE profiles for 10 randomly selected individuals per breed. Analyses assumed K=2 to K=5 genetic ancestry components, with K=4 representing the optimal value according to cross-validation.

(D) Total ROH length (Mb).

(E) Mean ROH length (kb).

(F) Inbreeding Coefficient based on ROH (F_{ROH}).

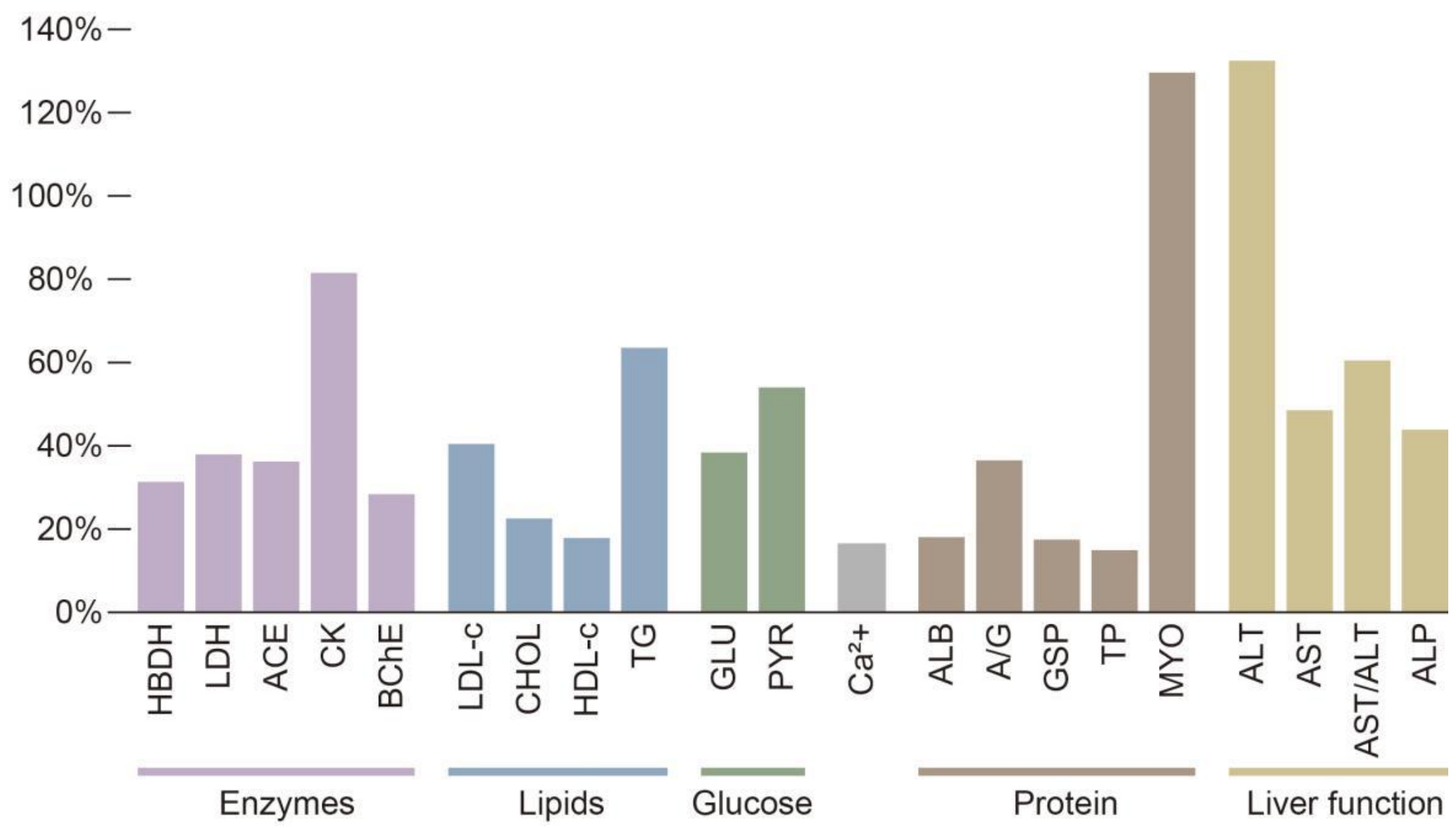

Figure S2. Coefficient of variation for 21 serum biochemical parameters, related to Figure 1. Serum parameters include: A/G=globulin ratio, ACE=angiotensin-converting enzyme activity (U/L), ALB=albumin concentration (g/L), ALP=alkaline phosphatase activity (U/L), ALT=alanine amino transferase activity (U/L), AST=aspartate amino transferase activity (U/L), AST/ALT=ratio of AST and ALT, BChE=butyrylcholinesterase activity (U/mL), Ca^{2+}=calcium concentration (mmol/L), CHOL=cholesterol concentration (mmol/L), CK=creatine kinase activity (U/L), GLU=glucose concentration (mmol/L), GSP=glycosylated serum protein concentration (μg/L), HBDH=α-hydroxy-butyrate dehydrogenase activity (U/L), HDL-c=high density lipoprotein cholesterol concentration (mmol/L), LDH=lactate dehydrogenase activity (U/L), LDL-c=low density lipoprotein cholesterol concentration (mmol/L), MYO=myoglobin concentration (μg/L), PYR=pyruvic acid concentration (mmol/L), TG=triglyceride concentration (mmol/L), and; TP=total protein concentration (g/L). Coefficients of variation were calculated by dividing the standard deviation by the average and are expressed in percentage. Colors indicate the main class to which each parameter belongs to.

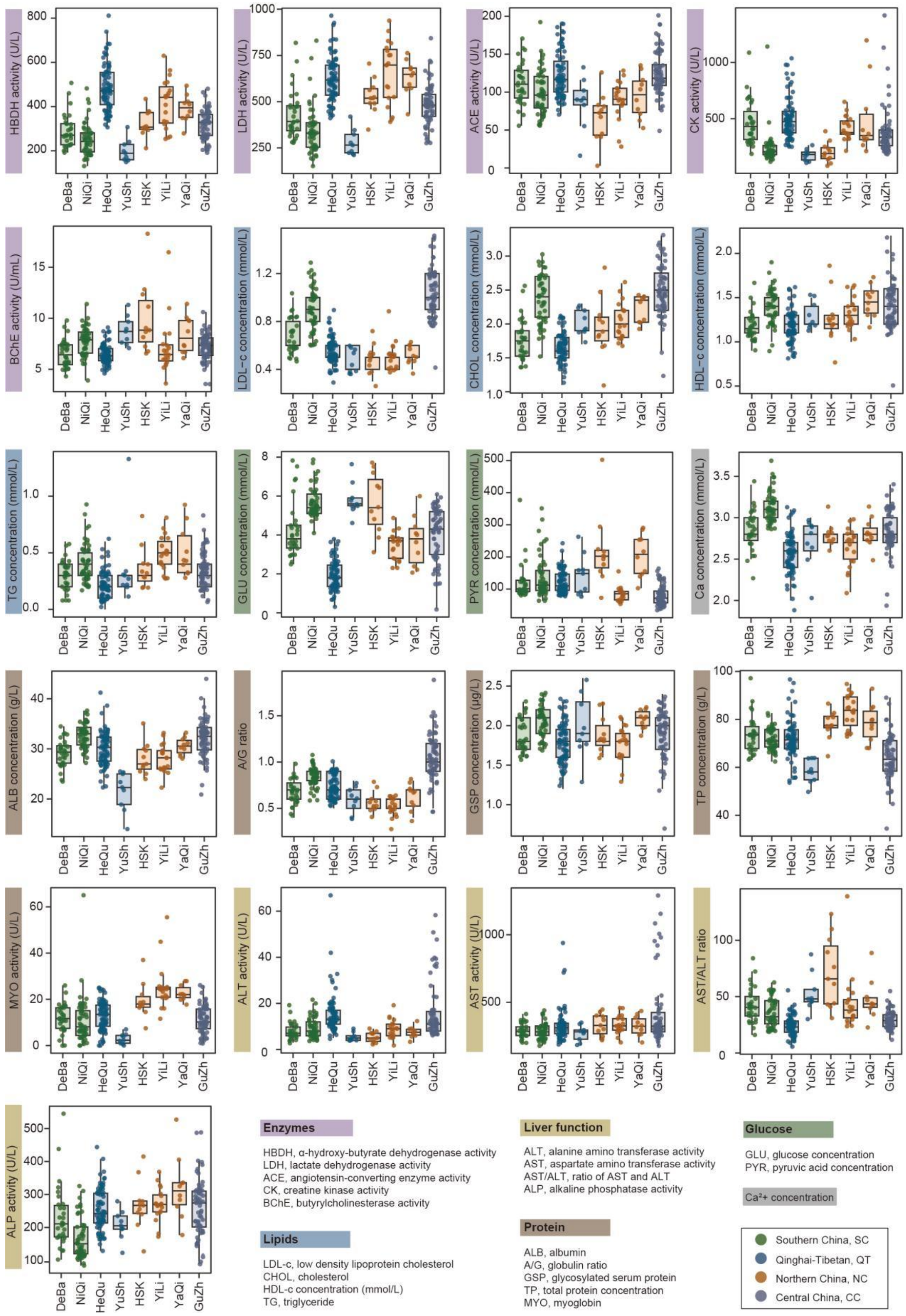

Figure S3. Individual measurements of 21 serum biochemical parameters in eight breeds of Chinese horses, related to Figure 1. DeBa=Debao, NiQi=Niqiang, HeQu=Hequ, YuSh=Yushu, GuZh=Guanzhong, HSK=Kazakh, YaQi=Yanqi, and YiLi=Yili.

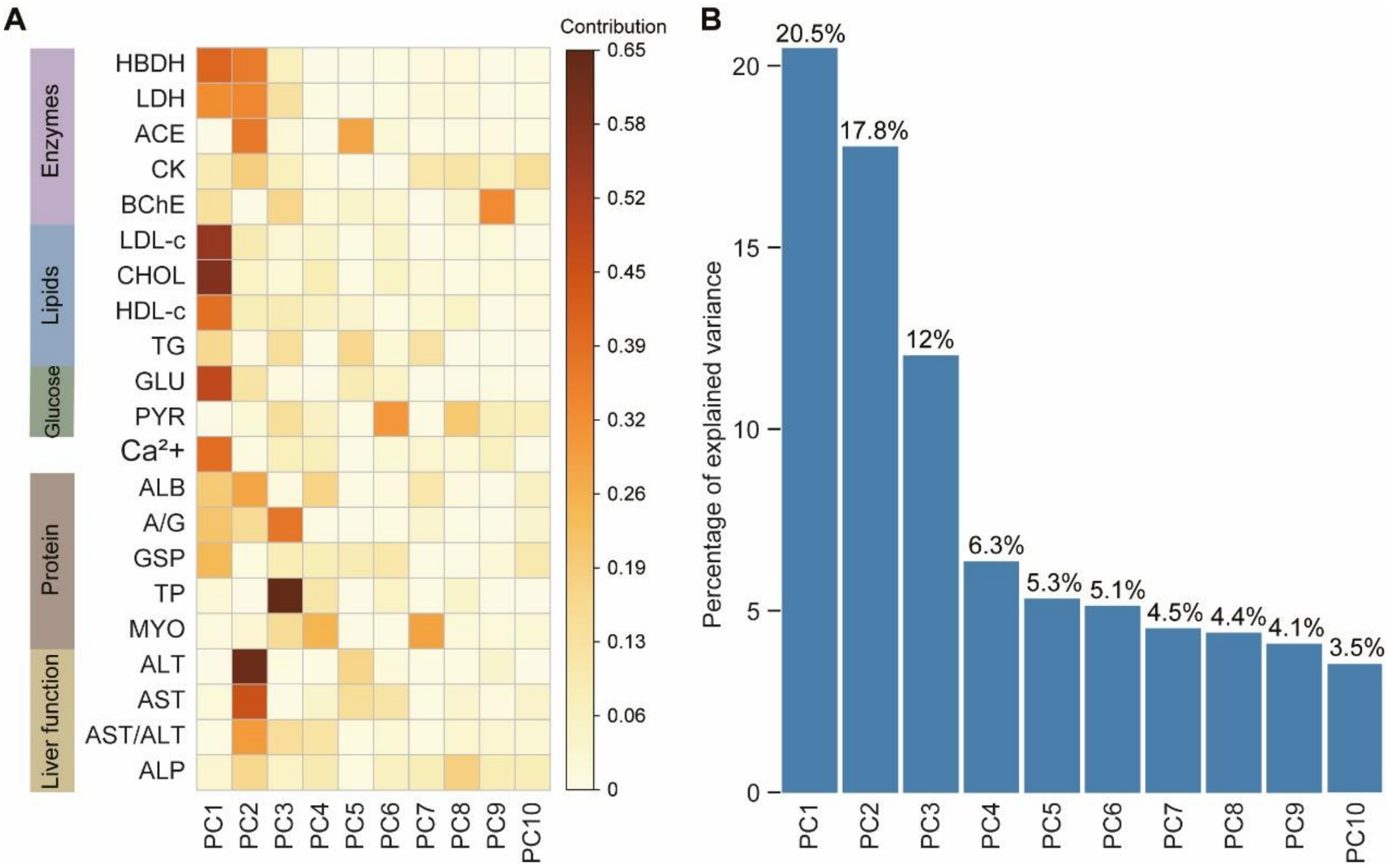

Figure S4. Principal Component Analysis of the variation measured in 21 serum biochemical parameters, related to Figure 1.

(A) Weight contribution of each serum biochemical parameter to the first 10 principal components.

(B) Fraction of the total phenotypic variance explained by the first 10 principal components. Combined, the first ten components explain 84.5% of the total variance. The location of each horse on the PCA space represented by the first two components is shown on Fig. 1c, and color-coded according to the breeds they belong to.

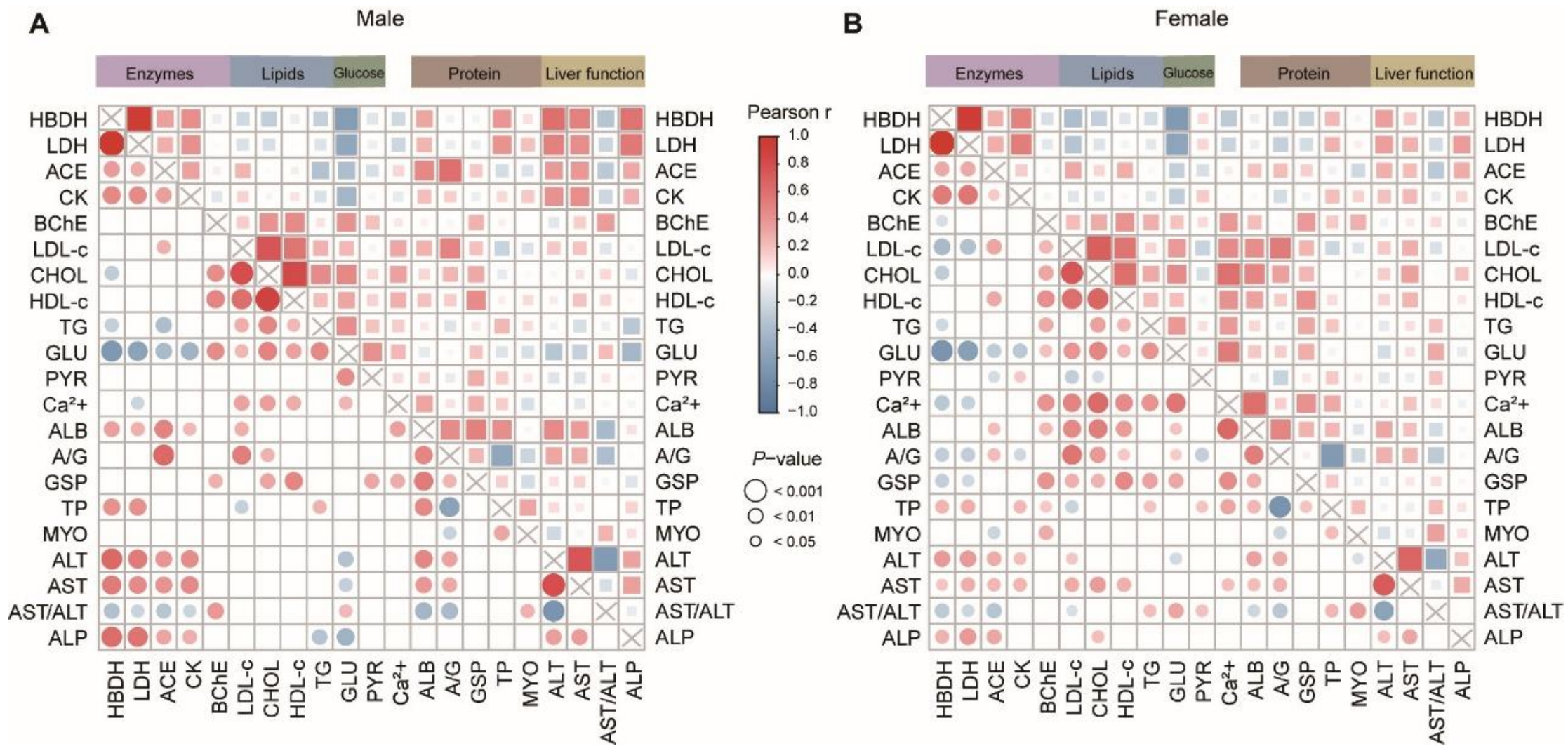

Figure S5. Matrices of pairwise Pearson correlations between biochemical parameters in males (A) and females (B) , related to Figure 1. The colors above the main diagonal are proportional to the correlation coefficients, while the size of the circles below the main diagonal indicate statistical significance.

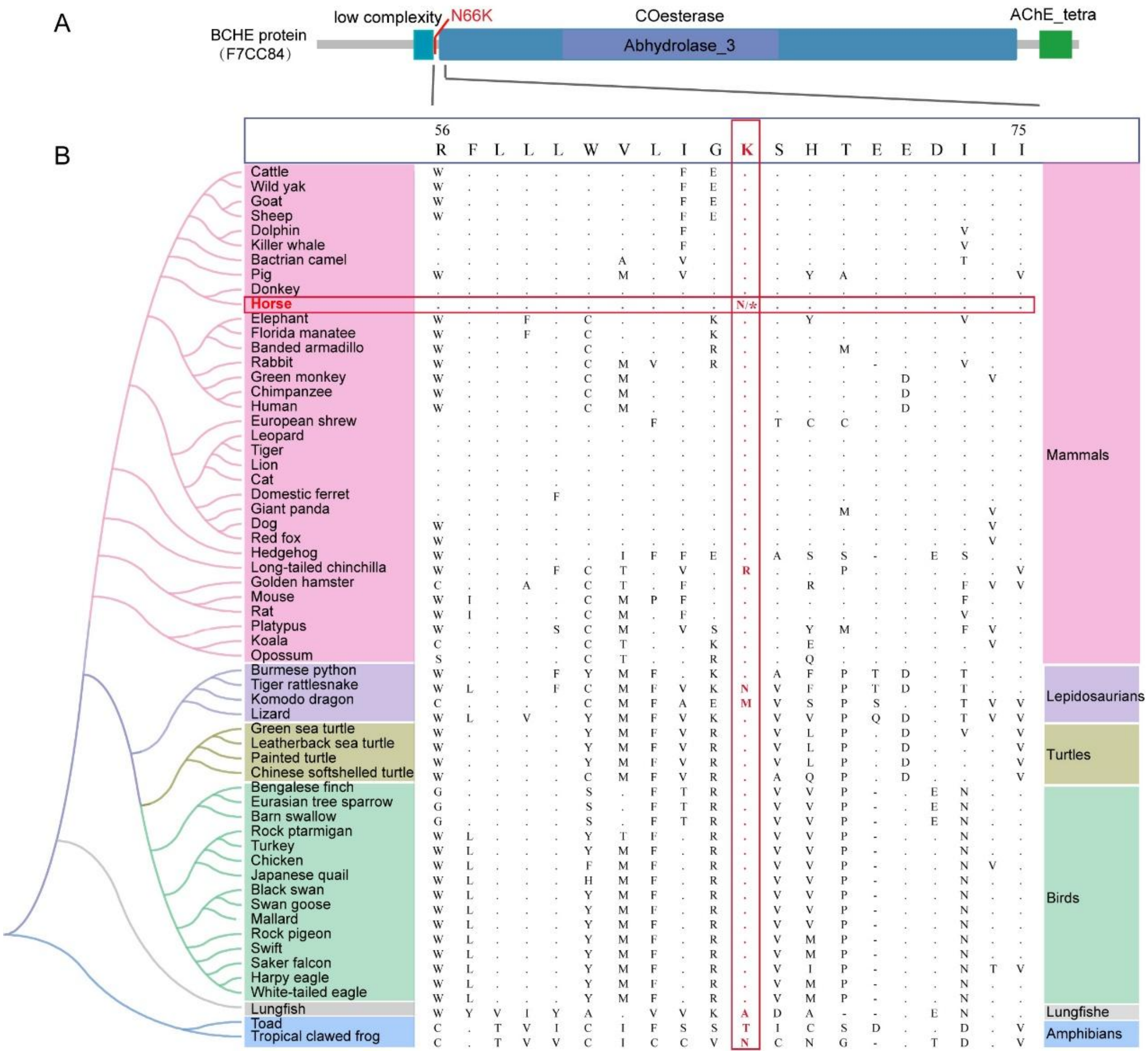

Figure S6. Conservation analysis of BCHE protein sequence, related to Figure 2.

(A) BCHE horse protein domains (AChE_tetra=Acetylcholinesterase tetramerization domain, COesterase= Carboxylesterase family, Abhydrolase_3, and; low-complexity domain).

(B) Sequence alignment between amino-acids 56 and 75 of 60 Vertebrate BCHE proteins (Table S6). The Neighbor-Joining tree represents the phylogenetic relationships between sequences along the entire BCHE protein, as obtained from MEGA X.

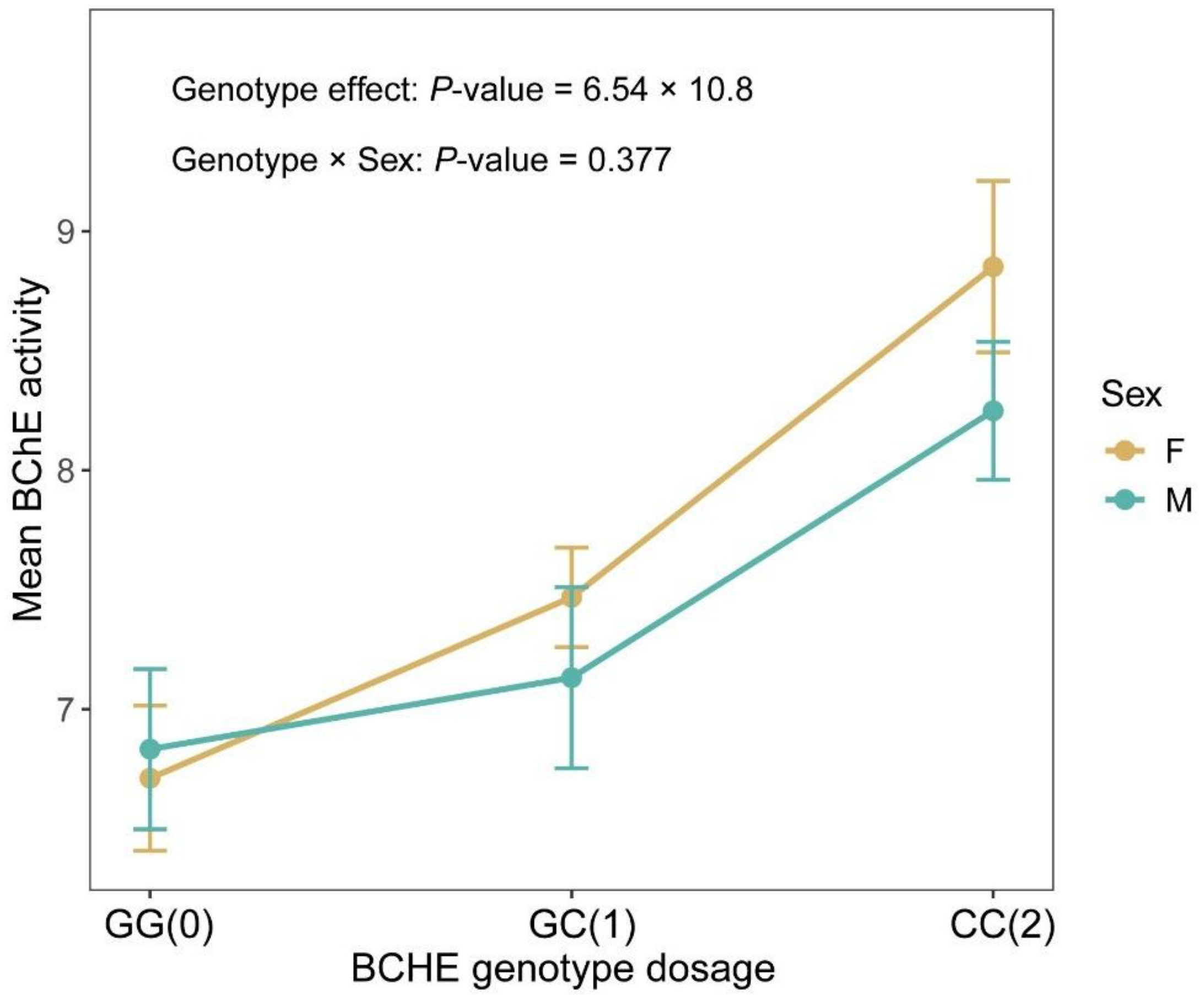

Figure S7. Sex-stratified association between the BCHE missense variant (ECA19:9,198,989, G/C) and BChE activity, related to Figure 2. Mean BChE activity is shown by genotype dosage of the BCHE missense variant (ECA19:9,198,989, G/C) separately for females and males, with error bars indicating standard errors. BChE activity increased with the "C"-allele dosage in both sexes. The variant effect remained significant after adjustment for sex and other covariates (β = 1.04, *P*-value = 6.54×10^{-8}), whereas the variant × sex interaction was not significant (*P*-value = 0.377).

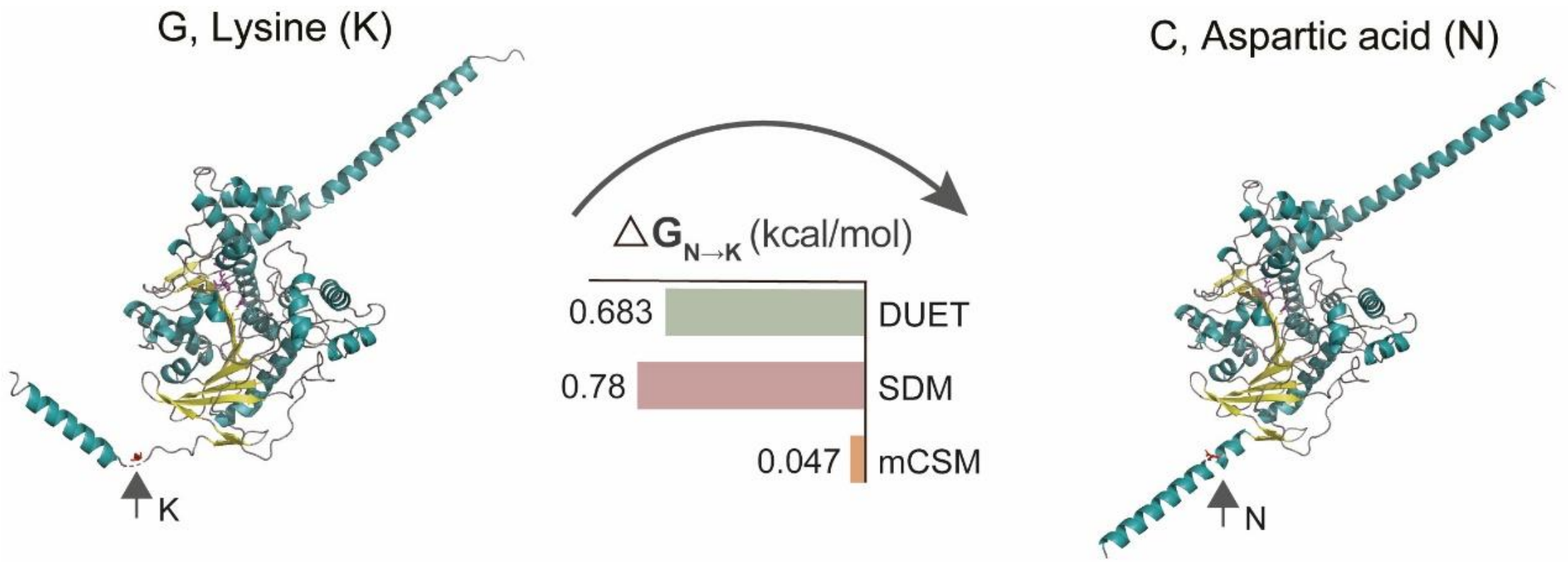

Figure S8. Predicted secondary structures for two genetic variants of the horse BCHE protein, related to Figure 2. The two variants modeled correspond to the "C" and "G" alleles identified at position ECA19:9198989. Colors represent various secondary structures, including alpha-helices (blue), beta-sheets (yellow), and random coils (grey). The arrows indicate the position of the mutated amino acids, with the "C" allele translating into Aspartic acid (N), and the "G" allele into Lysine (K). The middle panel displays the Gibbs free energy variation for the two protein models, as predicted using the trRosetta online server (http://struct.bioc.cam.ac.uk/duet), and assuming DUET, mCSM, and SDM methods.

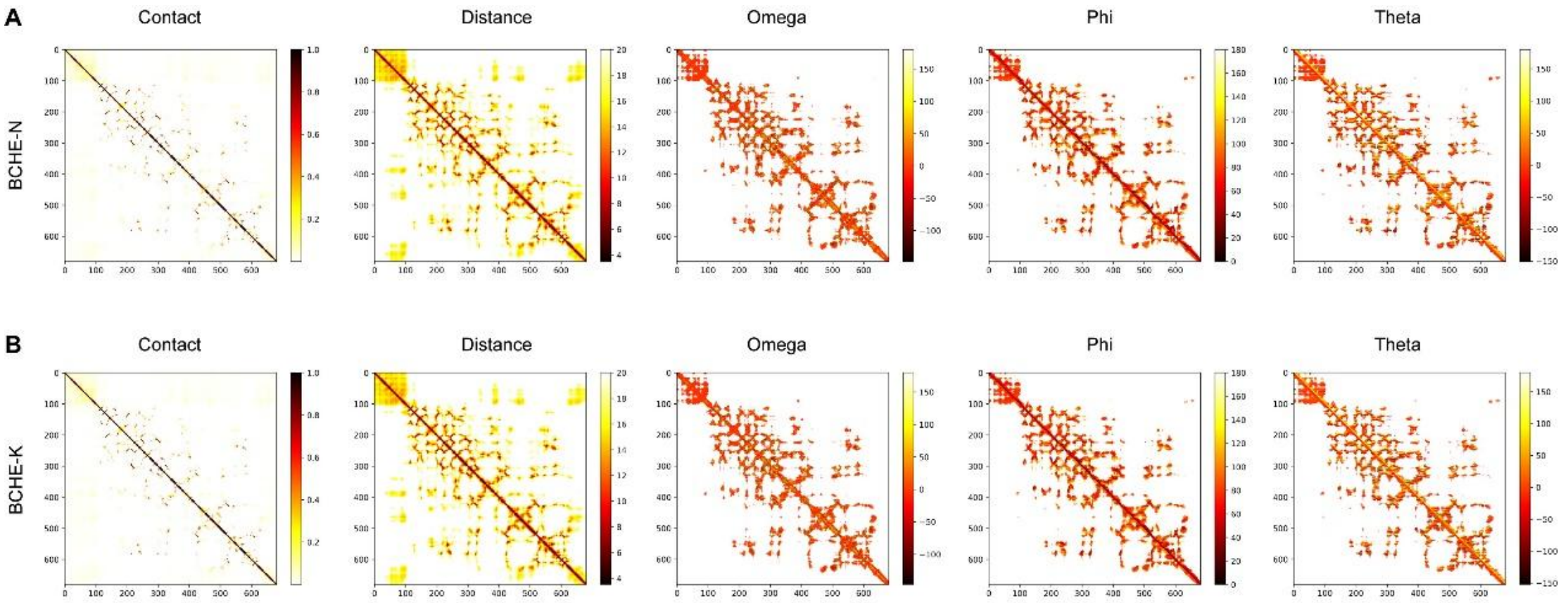

Figure S9. Predicted inter-residue distance and orientations distributions for BCHE variants, related to Figure 2. Panels display the output matrices from the trRosetta structure prediction network for the **(A)** horse-C and **(B)** horse-G protein sequences. For each variant, the five probabilistic distributions are shown: Contact (probability of residue pair contact), Distance (Å, between Cβ atoms), Omega (dihedral angle around the Cβ-Cβ virtual bond, °), Phi (rotation angle around the virtual Cβ-Cβ bond, °), and Theta (angle between Cα-Cβ vectors, °). These matrices represent the network's confidence and are used as constraints to generate the final 3D structural models.

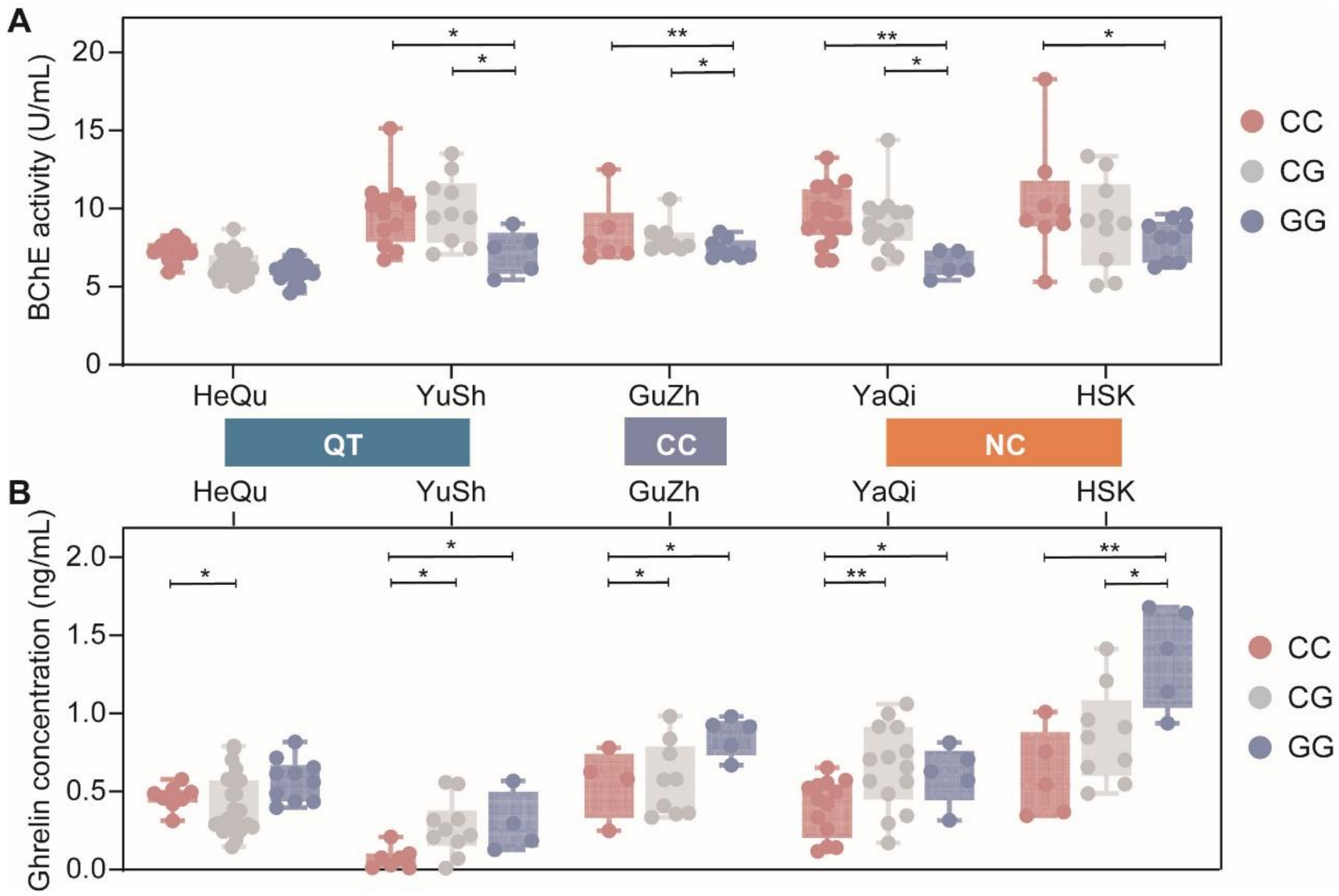

Figure S10. Serum BChE activity (U/mL) (A) and ghrelin concentration (ng/mL) (B) in five horse breeds for the CC, CG and GG genotypes at ECA19:9198989, related to Figure 2. Statistical significance was assessed using Kruskal-Wallis tests (*, *P*-value<0.05; *, *P*-value<0.01; **). Because the C allele is the main allele of interest in this study, genotypes are displayed as CC, CG, and GG in genotype-comparison panels.

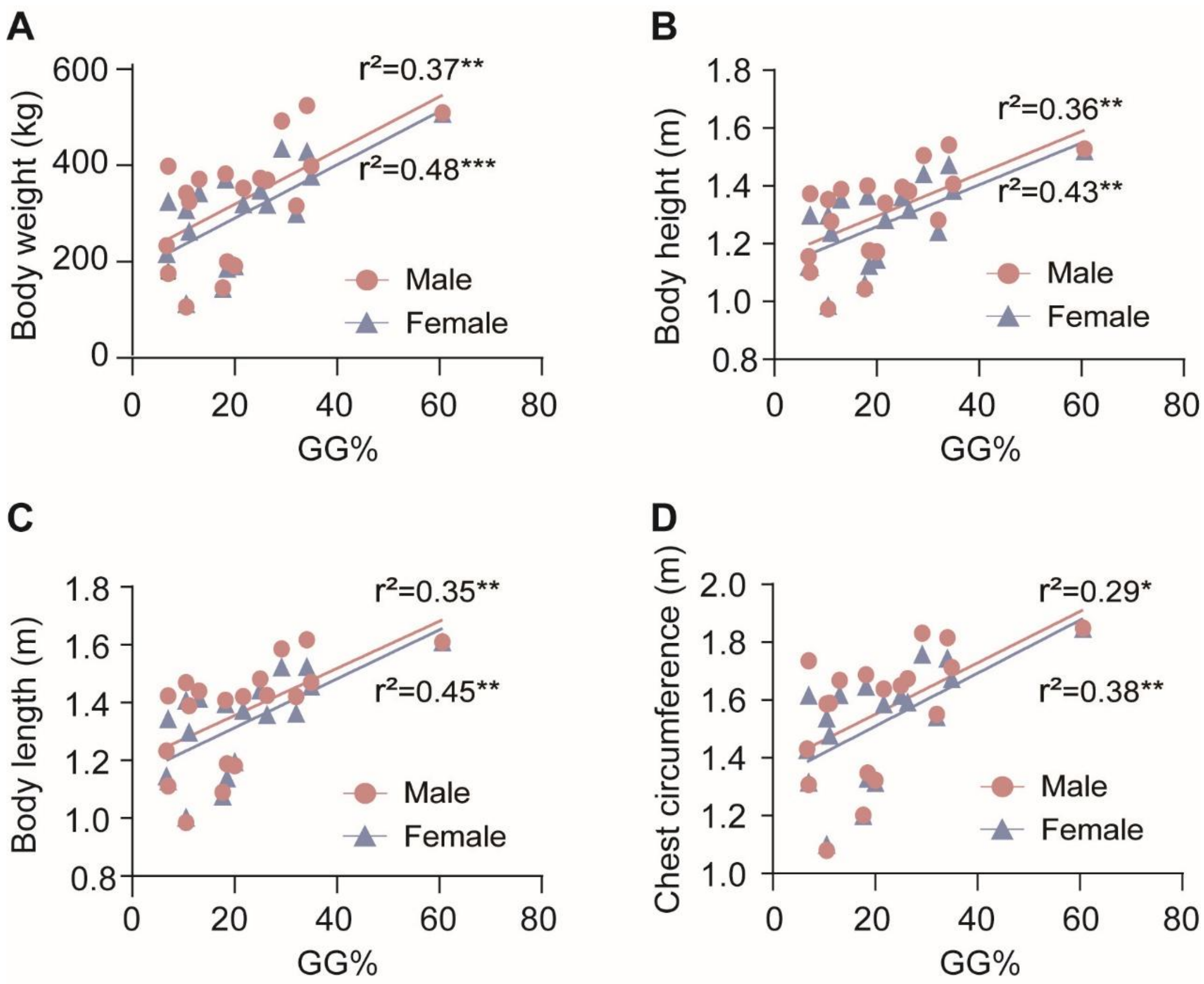

Figure S11. Sex-specific linear regression analysis between horse the frequency of the GG genotype at ECA19:9198989, and weight (A), body height (B), body length (C), chest circumference (D) , related to Figure 2. Red circles and blue triangles represent males and females, respectively. All body size data were from China National Commission of Animal Genetic Resources. R-squared Pearson correlation coefficients are indicated next to their respective models (*, P-value<0.05; **, P-value<0.01; ***, P-value<0.001).

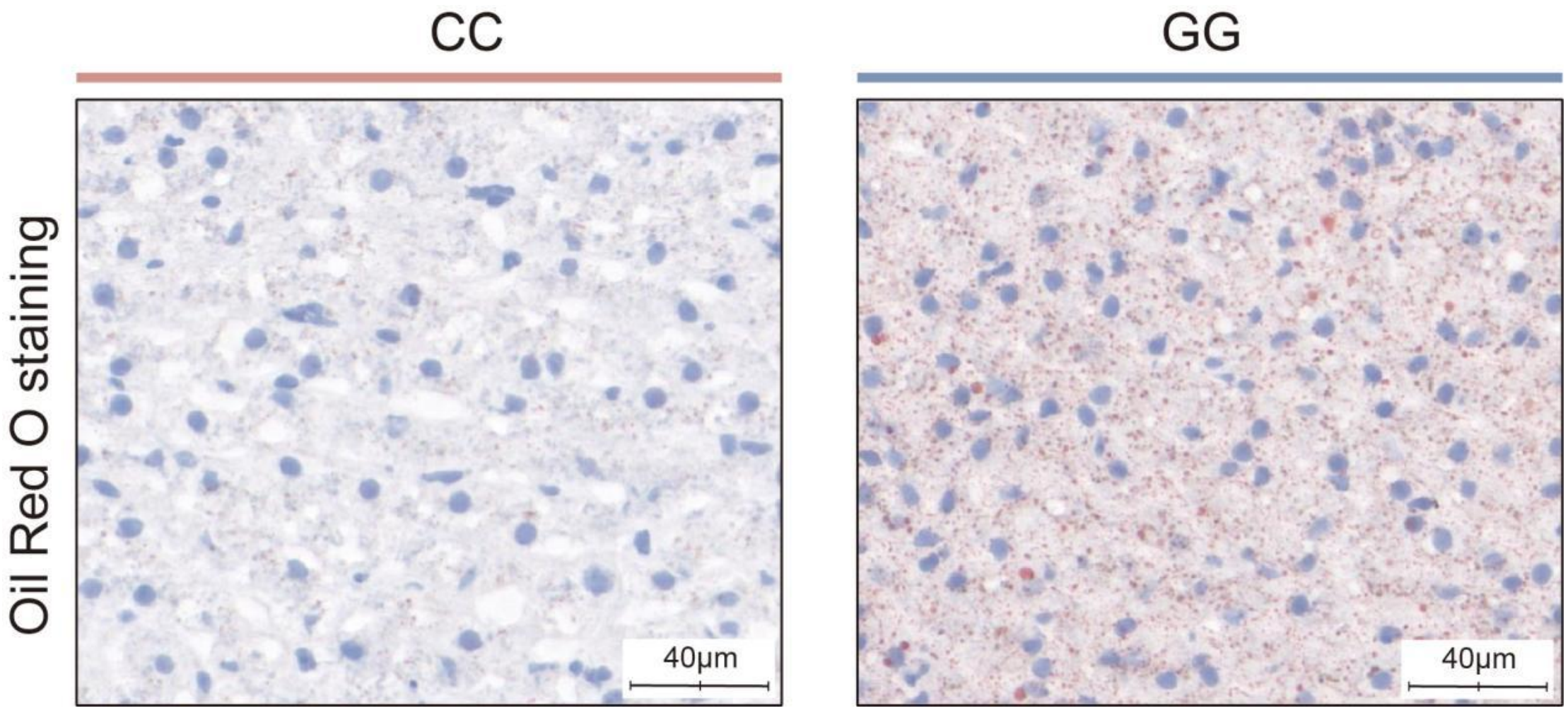

Figure S12. Oil red O staining of horse liver sections for two homozygous individuals at ECA19:9198989, related to Figure 2. The genotype of each individual is reported on the picture. Scale bar=40 μm.

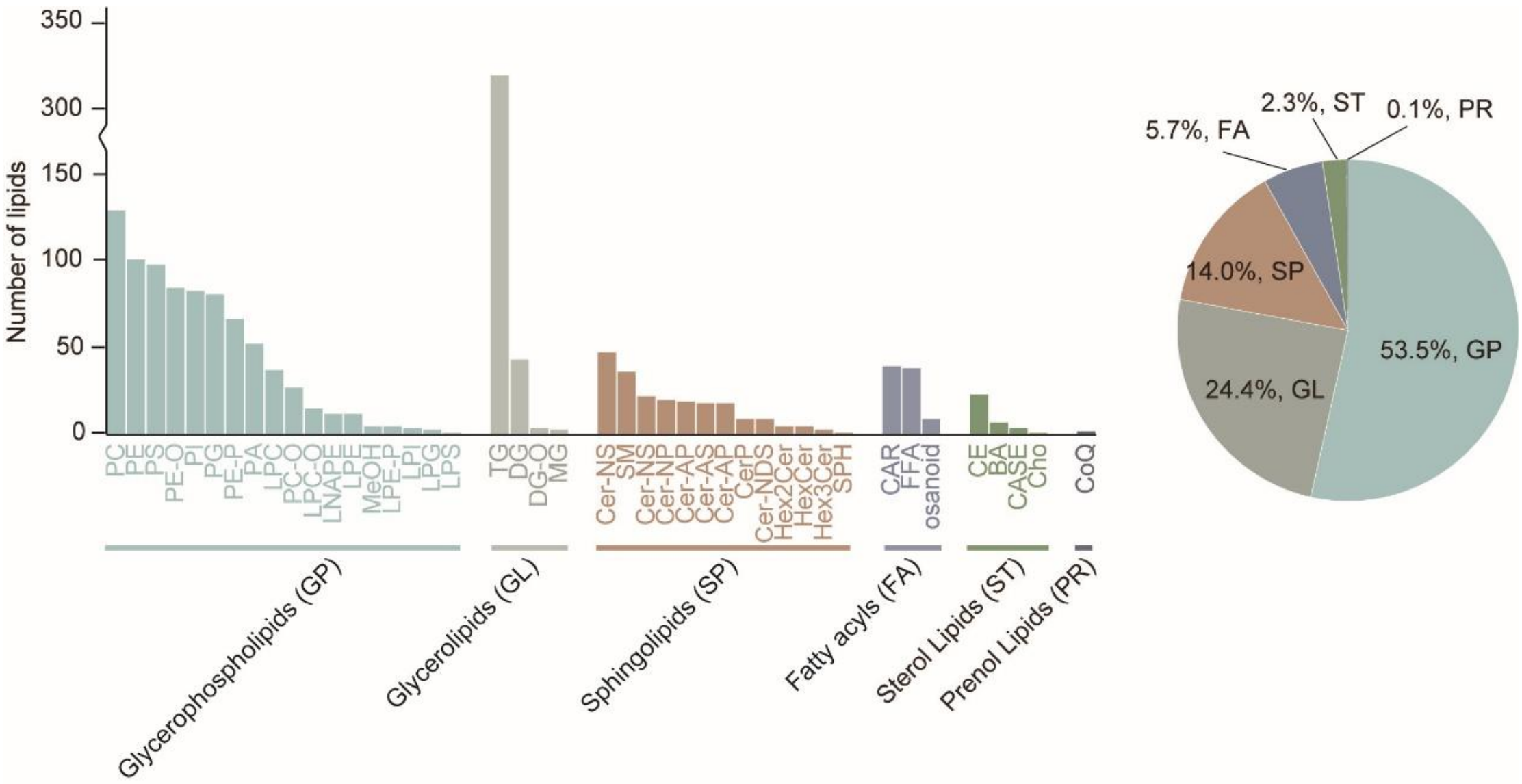

Figure S13. Distribution of the 1,515 lipids analyzed in this study, related to Figure 2. Lipids were divided into six categories, including: Glycerophospholipids (GP, 53.5%), Glycerolipids (GL, 24.4%), Sphingolipids (SP, 14.0%), Fatty acyls (FA, 5.7%), Sterol Lipids (ST, 2.3%), and; Prenol Lipids (PR, 0.1%). These six categories are shown in different colors, and include a total of 43 biochemical sub-classes.

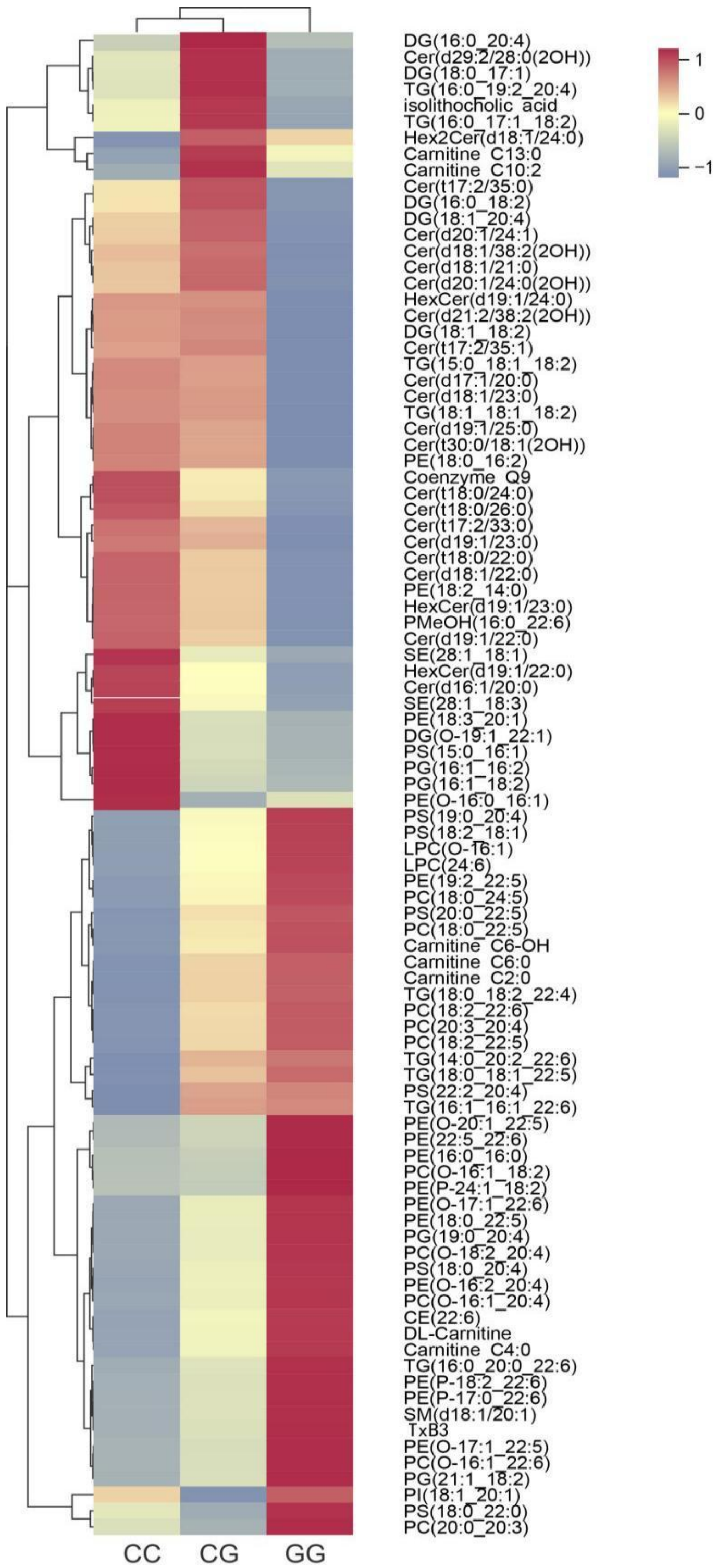

Figure S14. Hierarchical clustering heatmap of blood relative concentrations for 93 lipids showing strong statistical association with BCHE genotypes, related to Figure 2. The set of lipids was identified following orthogonal partial least square-discriminant analysis (OPLS-DA), based on variable importance in projection (VIP) values >1, |fold change, FC|≥1.5, and *P*-values <0.05.

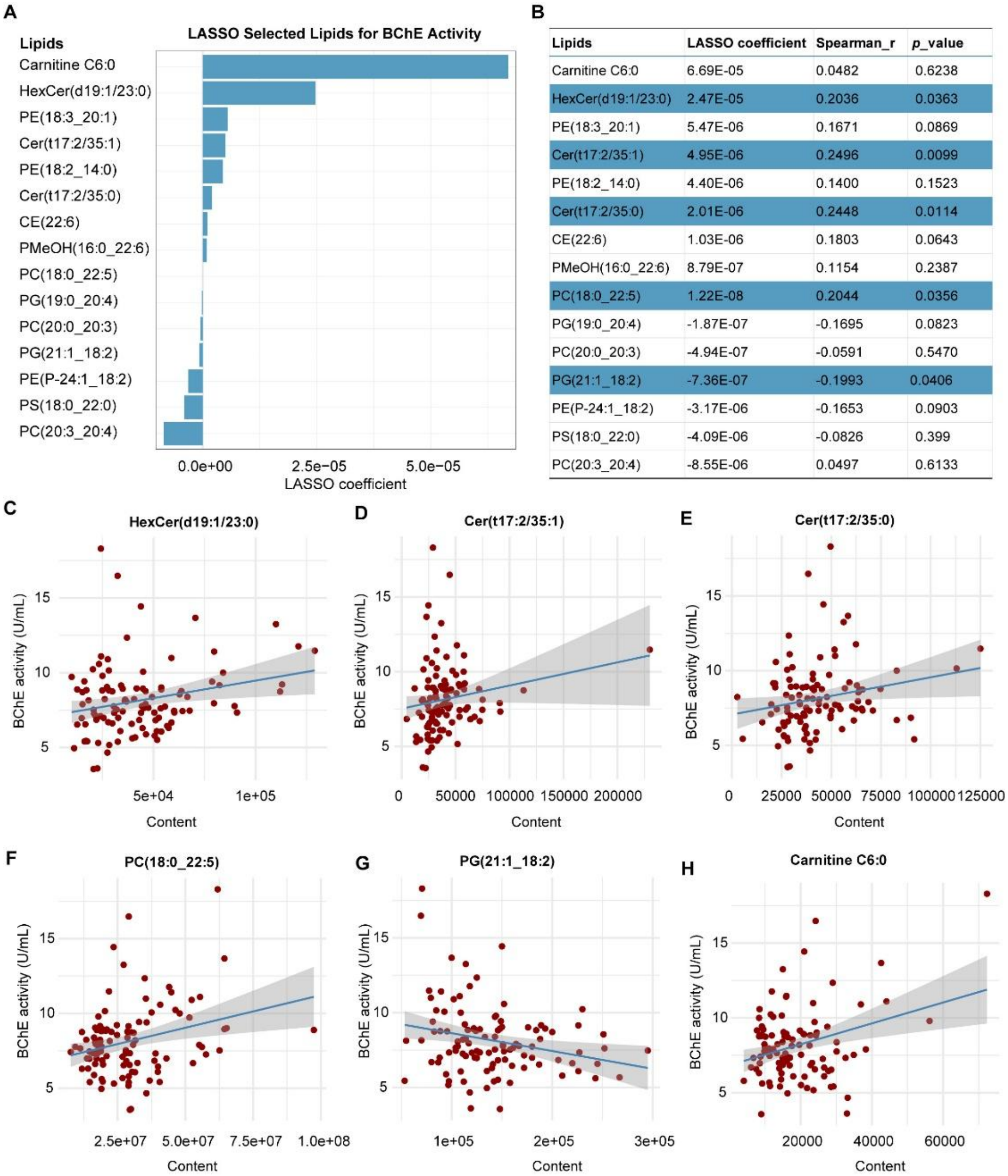

Lipids	LASSO coefficient	Spearman_r	*p*_value
Carnitine C6:0	6.69E-05	0.0482	0.6238
HexCer(d19:1/23:0)	2.47E-05	0.2036	0.0363
PE(18:3_20:1)	5.47E-06	0.1671	0.0869
Cer(t17:2/35:1)	4.95E-06	0.2496	0.0099
PE(18:2_14:0)	4.40E-06	0.1400	0.1523
Cer(t17:2/35:0)	2.01E-06	0.2448	0.0114
CE(22:6)	1.03E-06	0.1803	0.0643
PMeOH(16:0_22:6)	8.79E-07	0.1154	0.2387
PC(18:0_22:5)	1.22E-08	0.2044	0.0356
PG(19:0_20:4)	-1.87E-07	-0.1695	0.0823
PC(20:0_20:3)	-4.94E-07	-0.0591	0.5470
PG(21:1_18:2)	-7.36E-07	-0.1993	0.0406
PE(P-24:1_18:2)	-3.17E-06	-0.1653	0.0903
PS(18:0_22:0)	-4.09E-06	-0.0826	0.399
PC(20:3_20:4)	-8.55E-06	0.0497	0.6133

Figure S15. Least absolute shrinkage and selection operator (LASSO) regression analysis for the identification of lipid features associated with BChE activity, related to Figure 2.

(A) LASSO coefficients of the selected lipids contributing to BChE activity. LASSO regression analysis of 93 differential lipids identified from pairwise comparisons resulted in 15 lipids with non-zero coefficients.

(B) Table summarizing LASSO coefficients together with Spearman correlation coefficients (r) and corresponding *p*_values between lipid abundances and BChE activity.

(C–H) Scatter plots showing the correlations between BChE activity and six lipids (HexCer(d19:1/23:0), Cer(t17:2/35:1), Cer(t17:2/35:0), PC(18:0_22:5), PG(21:1_18:2), and Carnitine C6:0) displaying strong associations supported by both LASSO selection and Spearman correlation analysis. Spearman's r and *p*_values are indicated in each panel.

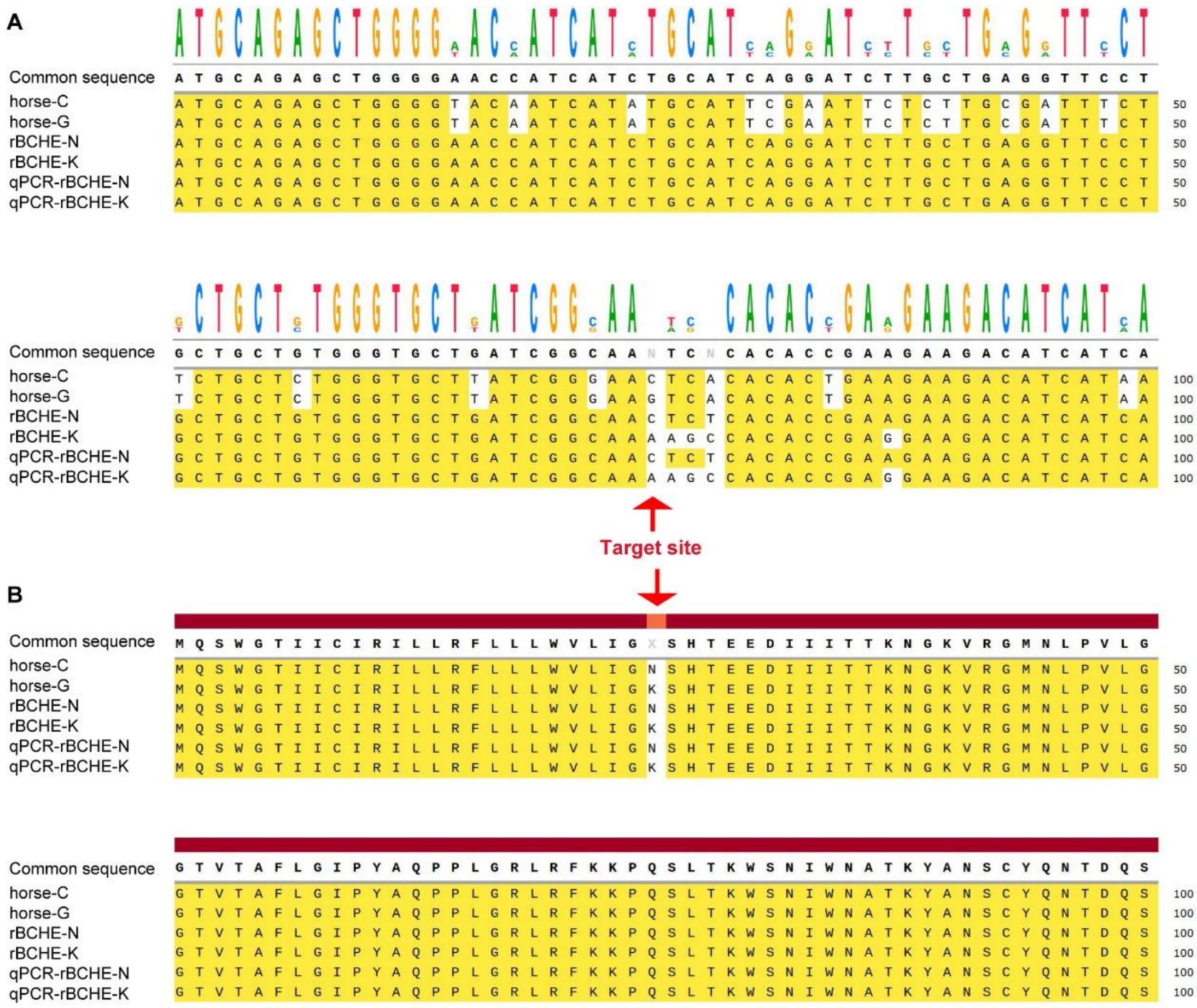

Figure S16. Sequence alignment of endogenous and optimized BCHE sequences, related to Figure 3.

(A) Nucleotide sequence alignment.

(B) Amino acid sequence alignment. Sequences include the endogenous equine genes (horse-C and horse-G), the constructed overexpression vectors (rBCHE-N and rBCHE-K), and the sequences amplified from RT-PCR products (qPCR-rBCHE-N and qPCR-rBCHE-K).

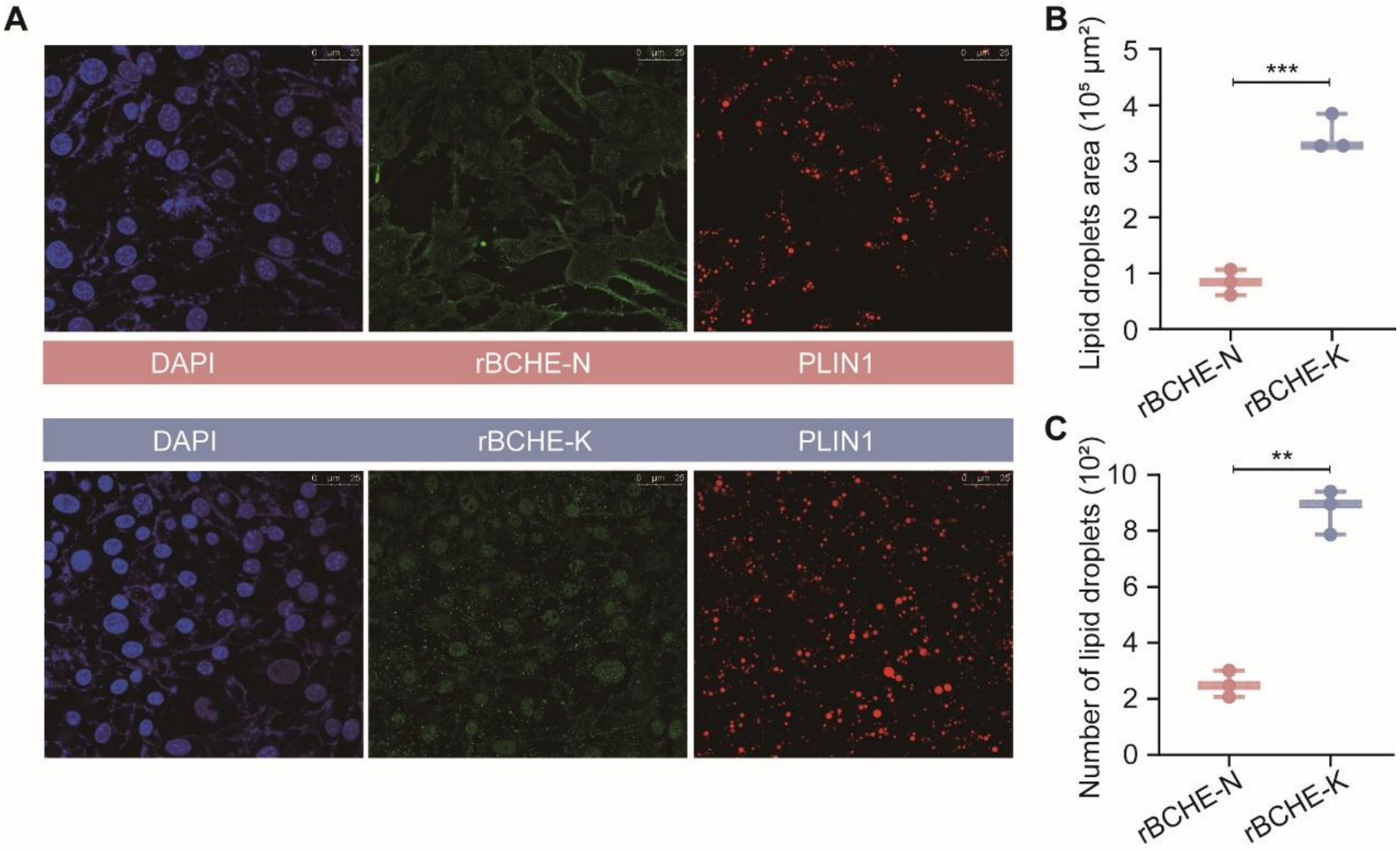

Figure S17. Immunofluorescence (IF) staining and quantification of flag-tagged BCHE and PLIN1 in HepG2 cells, related to Figure 3.

(A) IF staining. DAPI (blue) was used to identify nuclei, while AF488 (green) and CY3 (red) were used for visualizing the BCHE and PLIN1 proteins. The 'Merge' panels represent the cumulated signal of all three markers. *Top*: experiments carried out on cells expressing the "C" allele of the BCHE gene. Bottom: experiments carried out on cells expressing the "G" allele of the BCHE gene. Scale bar=25μm.

(B) Total lipid area (10^5 um^2). The total area encompassed by PLIN1 staining, as measured in ImageJ, provides a proxy for the total lipid area present in the picture analyzed.

(C) Number of lipid droplets, as measured in ImageJ on the basis of PLIN1 staining. In these experiments, adipogenesis was induced in transfected HepG2 cells by supplementing the culture medium with a mixture of sodium oleate and sodium palmitate (v:v=2:1). Because the C allele encodes the BChE-N form, recombinant BChE groups are displayed as rBChE-N, and rBChE-K.

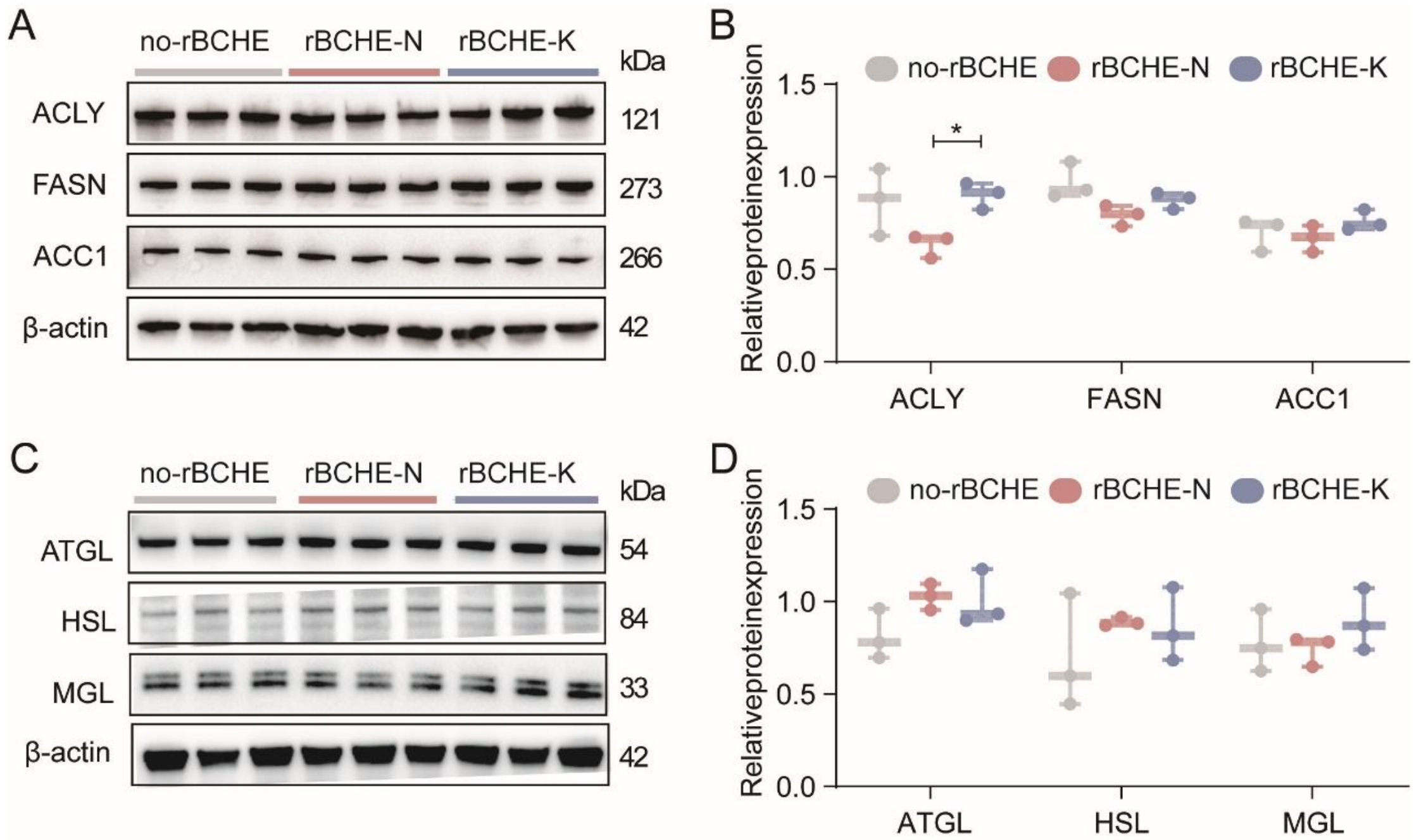

Figure S18. Lipogenesis (a) and lipolysis (b) control experiments in HepG2 cells, related to Figure 3.

(A) Western blot analysis of the levels of three proteins involved in lipogenesis. ACLY=ATP Citrate Lyase. FASN=Fatty Acid Synthase. ACC1=Acetyl-CoA Carboxylase Alpha. β-actin was used as a normalization control.

(B) Normalized protein levels of ACLY, FASN and ACC1, as measured with ImageJ.

(C) same as **(A)**, except that three proteins involved in lipolysis were considered. ATGL=Adipose Triglyceride Lipase. HSL=Hormone-Sensitive Lipase. MGL=Monoacylglycerol Lipase.

(D) Normalized protein levels of ATGL, HSL and MGL, as measured with ImageJ.

HepG2 cells were transfected with vectors carrying the “C” and “G” alleles of the BCHE, or an empty vector. Statistical significance was assessed using Kruskal-Wallis tests (*, *P*-value<0.05). Because the C allele encodes the BChE-N form, recombinant BChE groups are displayed as no-rBChE, rBChE-N, and rBChE-K.

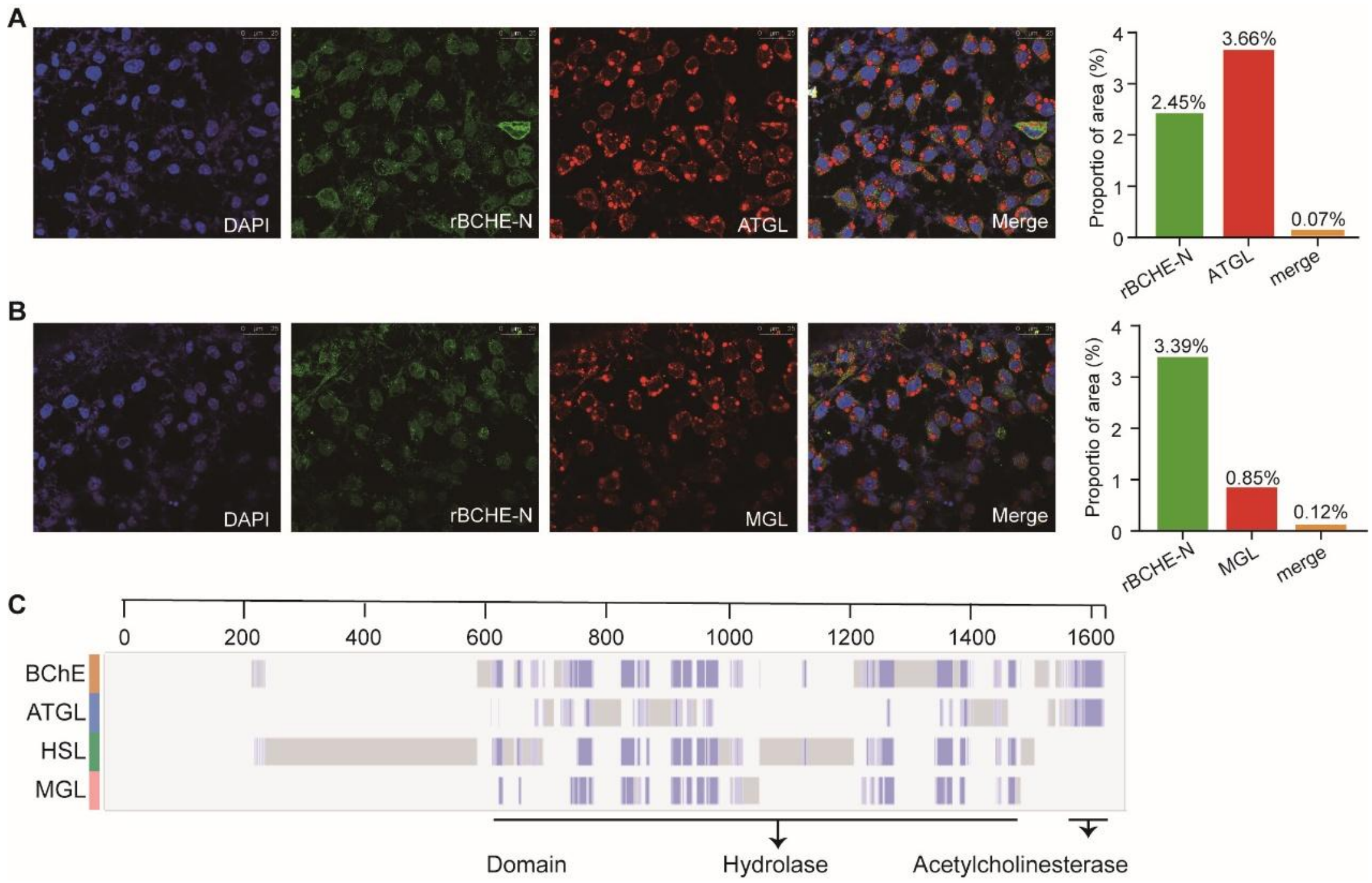

Figure S19. Immunofluorescence (IF) and structural comparison between four lipolytic enzymes, related to Figure 3. IF staining of nuclei, flag-tagged BChE, ATGL **(A)** (Adipose triglyceride Lipase), and MGL (Monoacylglycerol Lipase) **(B)** in HepG2 cells. DAPI (blue) was used to identify nuclei, while AF488 (green) and CY3 (red) were used for visualizing the BCHE and ATGL (top) or MGL (bottom) proteins. The 'merge' panels represent the cumulated signal of all three markers. Experiments were carried out on cells expressing the "C" allele of the *BCHE* gene. Scale bar=25μm. **(C)** Conservation analysis of the secondary structure of BCHE and classical lipolytic enzymes. Purple indicates similar protein secondary structures, whereas darkgrey indicates dissimilar secondary structures.

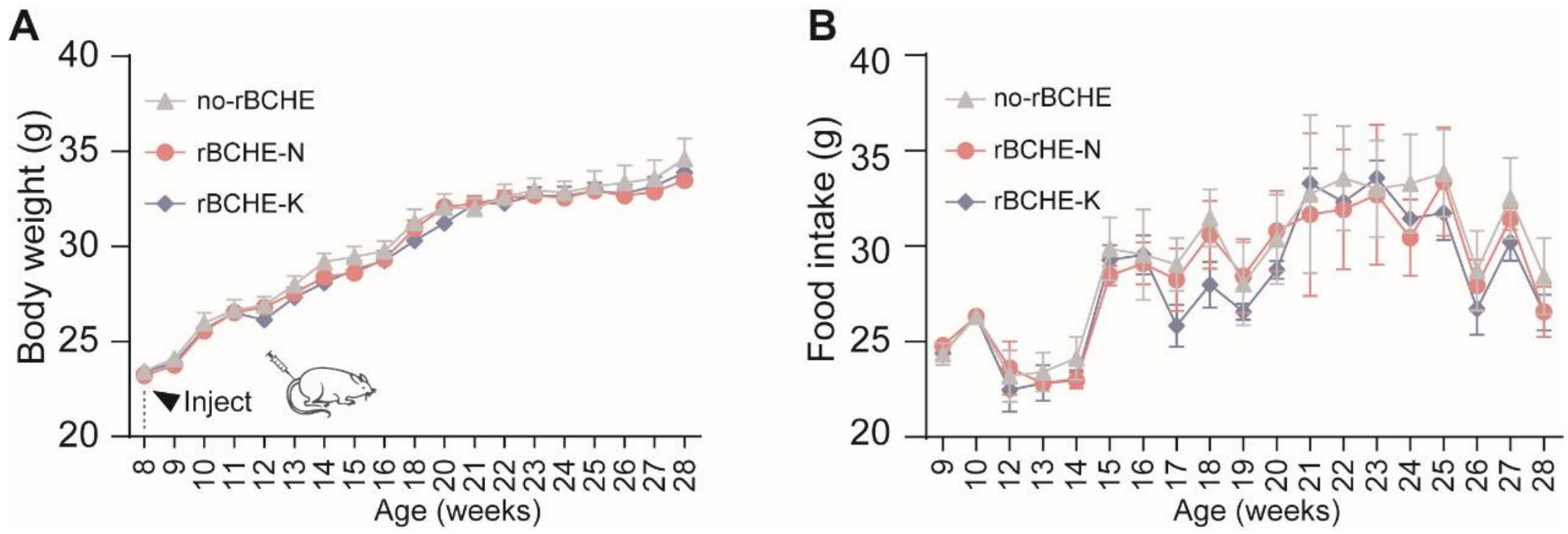

Figure S20. Body weight (A) and Food intake (B) monitoring of C57BL/6J mice, related to Figure 4.

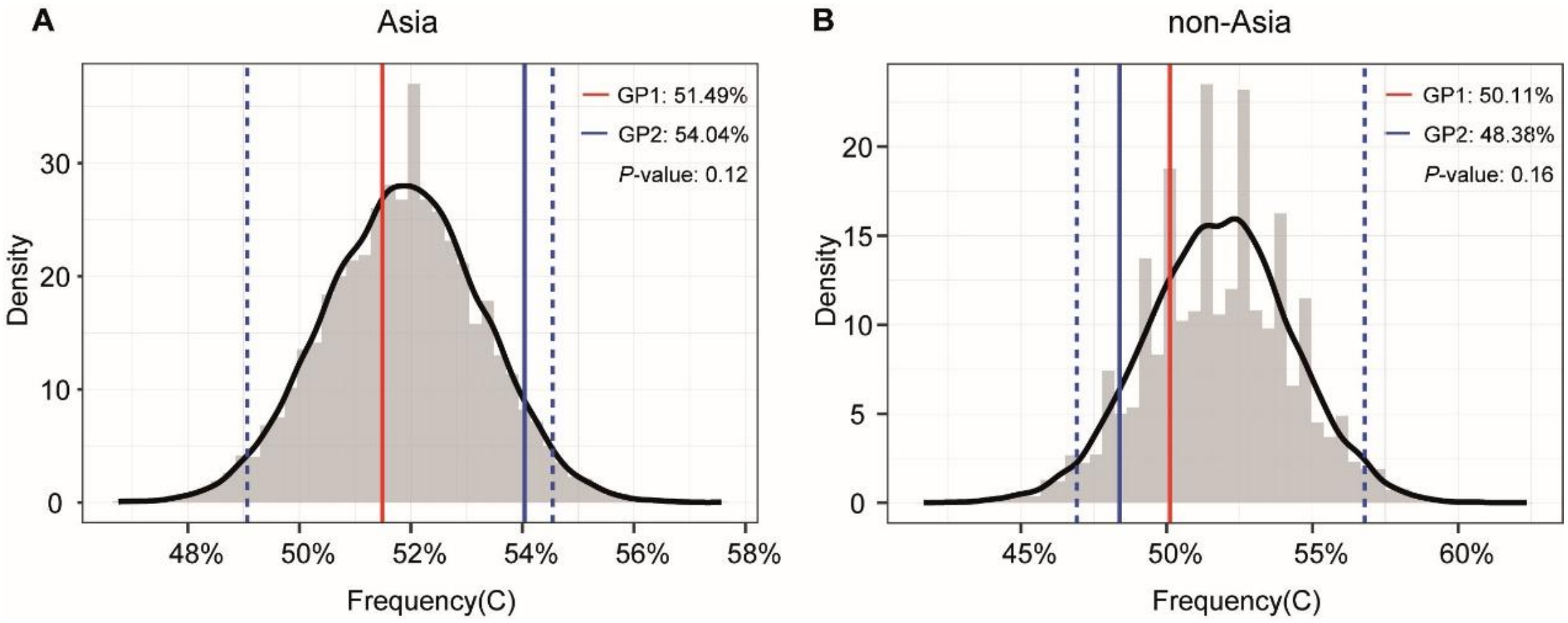

Figure S21. Resampling distribution of allele "C" frequency in Asia (A) and non-Asia (B) population of GP2 dataset, related to Figure 5. GP1 is a previously published global panel of horse genomes dataset. GP2 is this study dataset. The red solid line represents the actual frequency of allele "C" in the GP1 population, while the blue solid line represents the actual frequency of allele "C" in the GP2 population The blue dashed line represents the confidence interval for the GP2 population's weight sampling of 10,000 times.

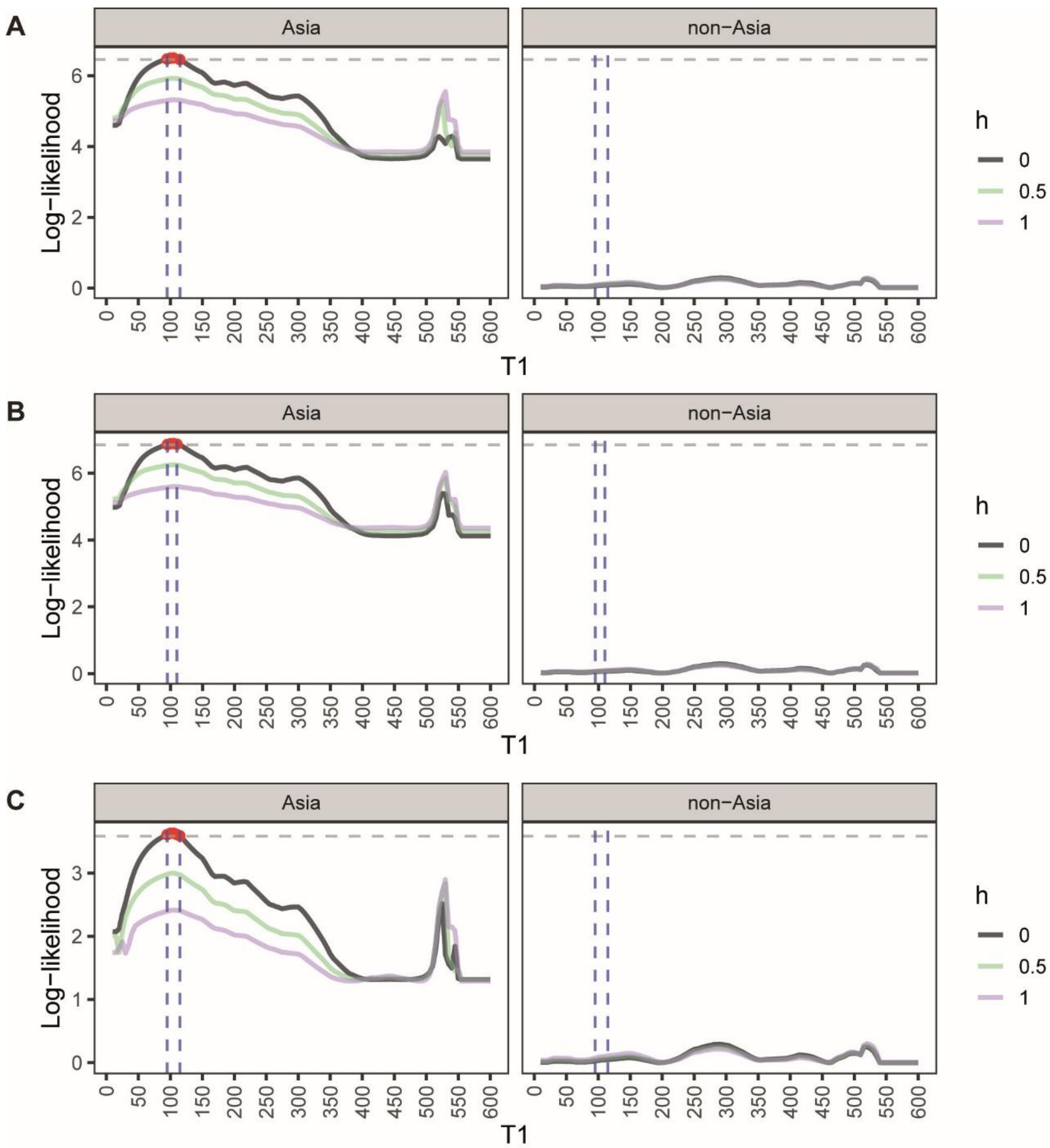

Figure S22. CLUES2 Log-Likelihood with sub-continental demographic models from Liu et al. (2025) (Asia, (A) vs non-Asia, (B)), and the global DOM2 demographic model from Der Sarkissian et al. (2015) (C) , related to Figure 5. Analyses were performed limiting the analyses to those specimens excavated in each individual region, before applying the subcontinental or global demographic model. We use a grid for inferring the time of selection shift, considering every 5 generations from 5 to 600 generations ago. The two vertical lines indicate the generations corresponding to the 99% Confidence Interval (CI).

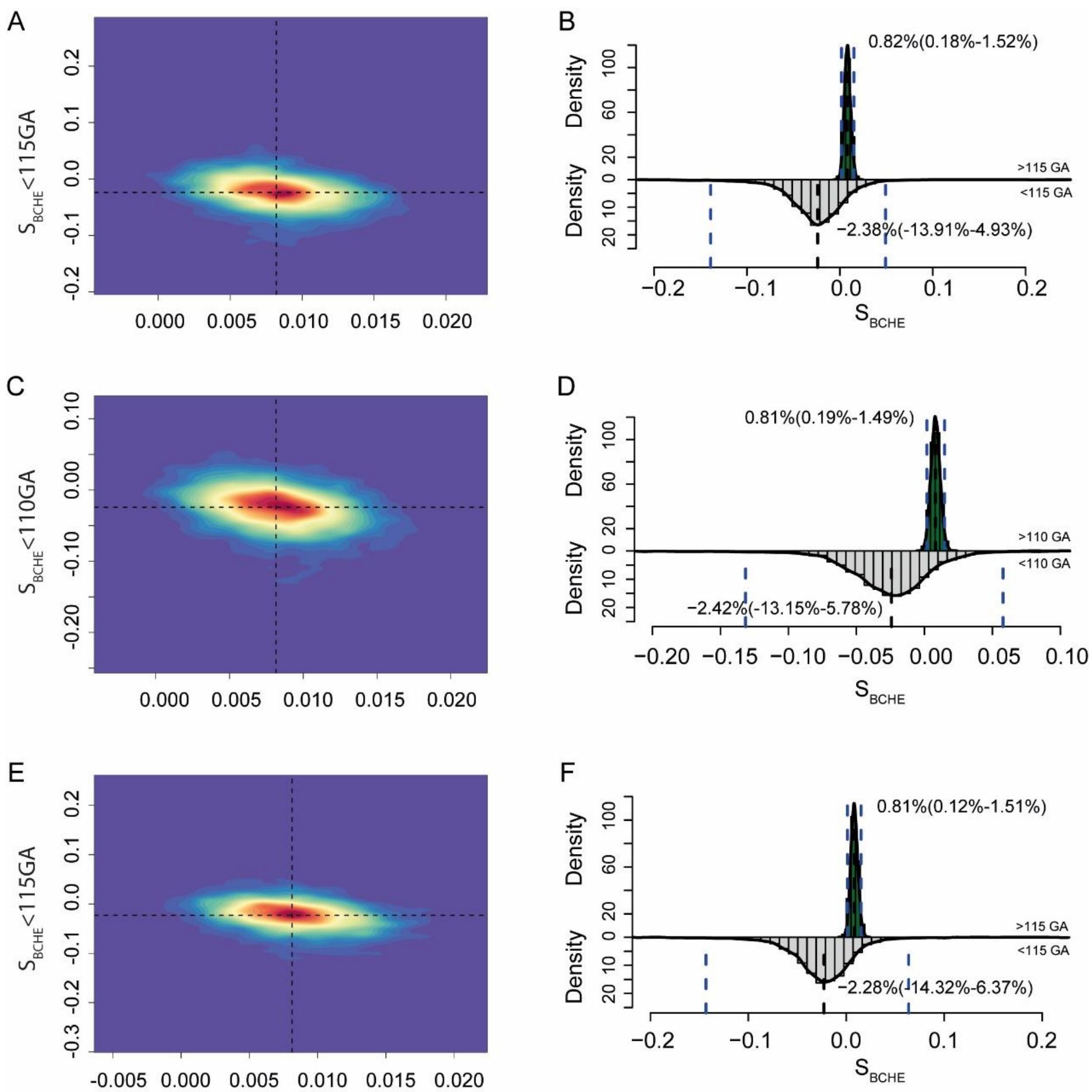

Figure S23. Selection analyses at the BCHE locus (ECA19:9,198,989, G>C) , related to Figure 5.

(A) PMMH joint kernel density posterior distributions for the selection coefficients prior to and after the time when the selection regime changed, using the subcontinental demographic model of Asia.

(B) Same as **(A)**, except that the individual posterior distributions of each selection coefficient are shown.

(C) PMMH joint kernel density posterior distributions for the selection coefficients prior to and after the time when the selection regime changed, using the non-Asian subcontinental demographic model .

(D) Same as **(C)**, except that the individual posterior distributions of each selection coefficient are shown.

(E) PMMH joint kernel density posterior distributions for the selection coefficients prior to and after the time when the selection regime changed, applying the DOM2 demographic model from Der Sarkissian et al. (2015) to ancient horse populations from Asia and outside Asia, separately.

(F) Same as **(E)**, except that the individual posterior distributions of each selection coefficient are shown.

The percentages reported indicate the posterior point estimates for the selection coefficients during the two temporal intervals considered, with 95% confidence intervals shown in parentheses. The blue dashed lines indicate the 95% confidence intervals of the posterior distributions.

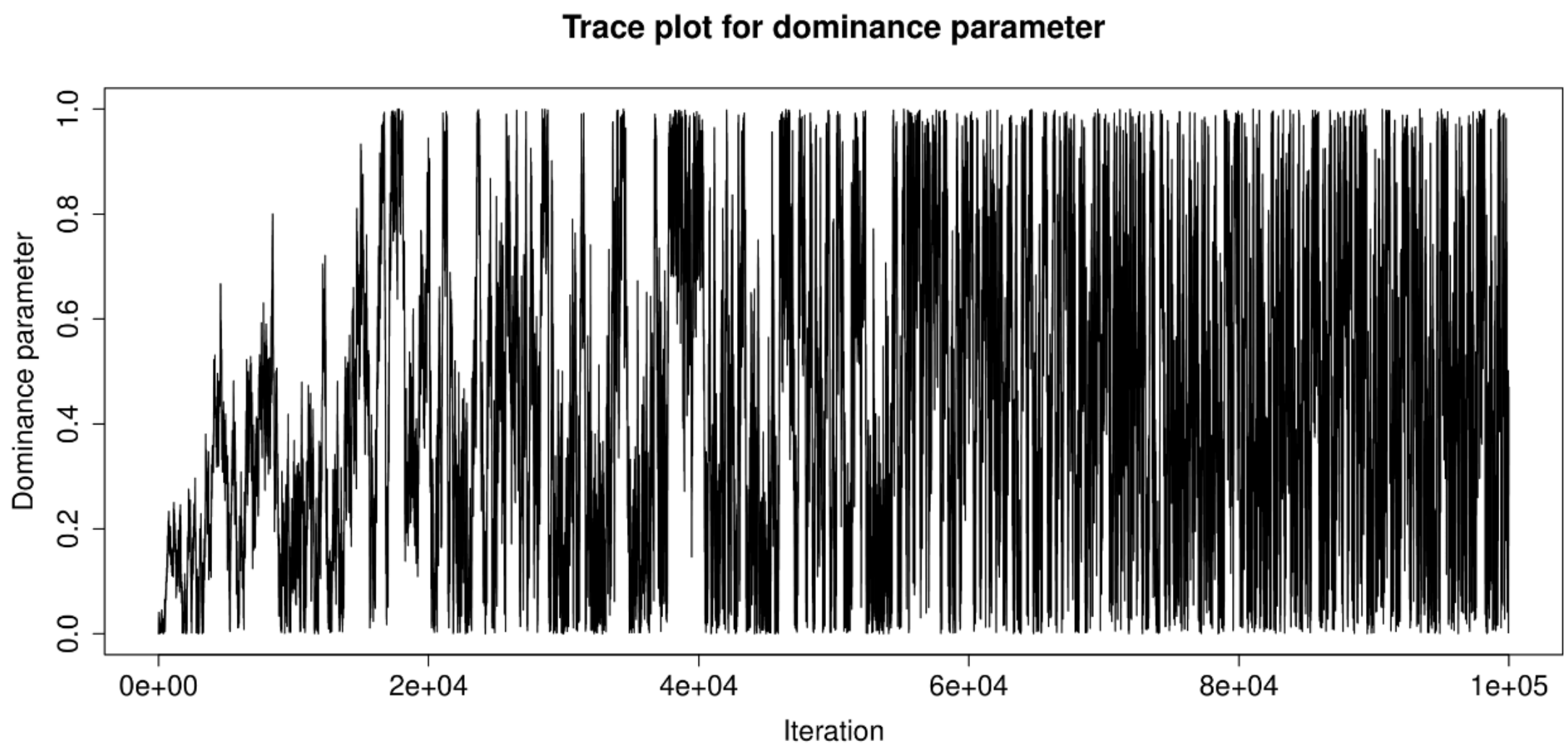

Figure S24. Trace plot of the dominance parameter (h) under the relaxed inheritance model, related to Figure 5.

Supplemental References

[S1] Letunic, I., Khedkar, S., and Bork, P. (2021). SMART: recent updates, new developments and status in 2020. Nucleic Acids Res. *49*, D458-D460. https://doi.org/10.1093/nar/gkaa937

[S2] Kumar, S., Stecher, G., Li, M., Knyaz, C., and Tamura, K. (2018). MEGA X: Molecular Evolutionary Genetics Analysis across Computing Platforms. Mol. Biol. Evol. *35*, 1547-1549. https://doi.org/10.1093/molbev/msy096

[S3] Su, H., Wang, W., Du, Z., Peng, Z., Gao, S.H., Cheng, M.M., and Yang, J. (2021). Improved Protein Structure Prediction Using a New Multi-Scale Network and Homologous Templates. Adv. Sci. *8*, e2102592. https://doi.org/10.1002/advs.202102592

[S4] Du, Z., Su, H., Wang, W., Ye, L., Wei, H., Peng, Z., Anishchenko, I., Baker, D., and Yang, J. (2021). The trRosetta server for fast and accurate protein structure prediction. Nat. Protoc. *16*, 5634-5651. https://doi.org/10.1038/s41596-021-00628-9

[S5] Wang, W., Peng, Z., and Yang, J. (2022). Single-sequence protein structure prediction using supervised transformer protein language models. Nat. Comput. Sci. *2*, 804-814. https://doi.org/10.1038/s43588-022-00373-3

[S6] Pires, D.E.V., Ascher, D.B., and Blundell, T.L. (2014). DUET: a server for predicting effects of mutations on protein stability using an integrated computational approach. Nucleic Acids Res. *42*, W314-W319. https://doi.org/10.1093/nar/gku411

[S7] Pires, D.E.V., Rodrigues, C.H.M., and Ascher, D.B. (2020). mCSM-membrane: predicting the effects of mutations on transmembrane proteins. Nucleic Acids Res. *48*, W147-W153. https://doi.org/10.1093/nar/gkaa416

[S8] Pandurangan, A.P., Ochoa-Montaño, B., Ascher, D.B., and Blundell, T.L. (2017). SDM: a server for predicting effects of mutations on protein stability. Nucleic Acids Res. *45*, W229-W235. https://doi.org/10.1093/nar/gkx439

[S9] Liu, X., Zhang, Y., Li, Y., Pan, J., Wang, D., Chen, W., Zheng, Z., He, X., Zhao, Q., Pu, Y.*, et al.* (2019). EPAS1 Gain-of-Function Mutation Contributes to High-Altitude Adaptation in Tibetan Horses. Mol. Biol. Evol. *36*, 2591-2603. https://doi.org/10.1093/molbev/msz158

[S10] McGarry, K.G., Schill, K.E., Winters, T.P., Lemmon, E.E., Sabourin, C.L., Harvilchuck, J.A., and Moyer, R.A. (2020). Characterization of Cholinesterases From Multiple Large Animal Species for Medical Countermeasure Development Against Chemical Warfare Nerve Agents. Toxicol. Sci. *174*, 124-132. https://doi.org/10.1093/toxsci/kfz250

[S11] McGarry, K.G., Lalisse, R.F., Moyer, R.A., Johnson, K.M., Tallan, A.M., Winters, T.P., Taris, J.E., McElroy, C.A., Lemmon, E.E., Shafaat, H.S.*, et al.* (2020). A Novel, Modified Human Butyrylcholinesterase Catalytically Degrades the Chemical Warfare Nerve Agent, Sarin. Toxicol. Sci. *174*, 133-146. https://doi.org/10.1093/toxsci/kfz251

[S12] Liu, X., Jia, Y., Pan, J., Zhang, Y., Gong, Y., Wang, X., Ma, Y., Alvarez, N., Jiang, L., and Orlando, L. (2025). Selection at the GSDMC locus in horses and its implications for human mobility. Science *389*, 925-930. https://doi.org/10.1126/science.adp4581
