# Supplementary material for: Adaptive variation in butyrylcholinesterase-influenced lipid metabolism during horse domestication
Source: Cell Rep. 2026 Aug 20;45(9):117879. doi: 10.1016/j.celrep.2026.117879 (PMC13597480; doi:10.1016/j.celrep.2026.117879)
Supplement: Supplementary file 2 [file mmc16.pdf]

Cell Reports

Article

Adaptive variation in butyrylcholinesterase-influenced lipid metabolism during horse domestication

Graphical abstract

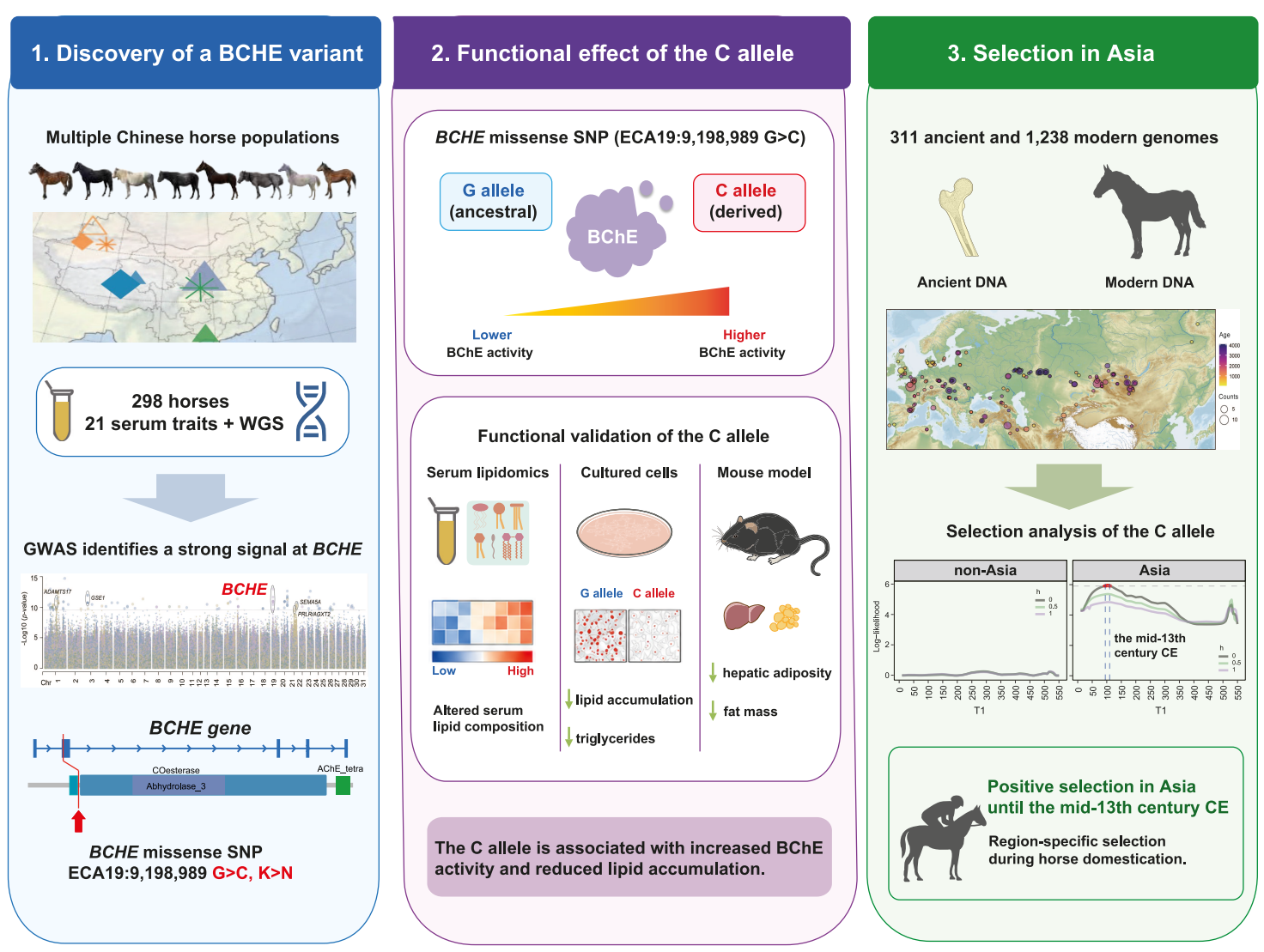

Highlights

- One missense SNP in *BCHE* affects lipid metabolism in horses
- The C variant increases BChE activity and reduces lipid accumulation
- Cellular and mouse models support a role for *BCHE* in fat storage
- The C variant was positively selected in Asian horses until the mid-13th century CE

Authors

Yanli Zhang, Xuexue Liu, Xintong Wang, ..., Yuehui Ma, Ludovic Orlando, Lin Jiang

Correspondence

jianglin@caas.cn

In brief

Zhang et al. identify a missense mutation in *BCHE* that affects lipid metabolism in horses. Cellular and mouse models show that the C variant increases BChE activity and reduces lipid accumulation. Ancient DNA analyses reveal positive selection in Asian horses until the mid-13th century CE.

Zhang et al., 2026, Cell Reports *45*, 117879
September 22, 2026 © 2026 The Authors. Published by Elsevier Inc.
https://doi.org/10.1016/j.celrep.2026.117879

Cell Reports

CellPress
OPEN ACCESS

Article

Adaptive variation in butyrylcholinesterase-influenced lipid metabolism during horse domestication

Yanli Zhang,[1,2,3,6] Xuexue Liu,[1,3,6] Xintong Wang,[4,5] Yanhui Yu,[1,2] Yaozhen Jia,[1,2] Yabin Pu,[1,2] Yuehui Ma,[1,2] Ludovic Orlando,[3] and Lin Jiang[1,2,7,*]

[1]State Key Laboratory of Animal Biotech Breeding, Institute of Animal Sciences, Chinese Academy of Agricultural Sciences (CAAS), Beijing 100081, China
[2]National Germplasm Center of Domestic Animal Resources, Ministry of Technology, Institute of Animal Sciences, Chinese Academy of Agricultural Sciences (CAAS), Beijing 100081, China
[3]Centre for Anthropobiology and Genomics of Toulouse (CNRS/Université de Toulouse), Faculté de Santé, Bâtiment A, 37 Allées Jules Guesde, 31000 Toulouse, France
[4]College of Animal Science and Technology, Qingdao Agricultural University, Qingdao, Shandong 266109, China
[5]Gongzhuling City Animal Disease Prevention and Control Center, Gongzhuling, Jilin 136100, China
[6]These authors contributed equally
[7]Lead contact
*Correspondence: jianglin@caas.cn
https://doi.org/10.1016/j.celrep.2026.117879

SUMMARY

Lipid metabolism influences horse athletic performance, yet its genetic basis remains unclear. Here, we investigate the genetic basis of lipid metabolic traits by quantifying key metabolic parameters and sequencing the genomes of 298 horses. We identify a missense mutation (ECA19:9,198,989, G>C) in the *BCHE* gene that is strongly associated with lipid metabolism, especially butyrylcholinesterase (BChE) activity. The "C" allele is associated with increased BChE activity and reduced intracellular triglyceride levels, hepatic adiposity, and overall fat mass, highlighting a central role for *BCHE* in lipid hydrolysis and fat storage in horses. This variant evolved neutrally across most of the horse domestication history outside Asia, but underwent strong positive selection in Asia until the 13th century CE. This spatiotemporal divergence suggests that Asian and non-Asian horses experienced distinct selective pressures on lipid metabolism, possibly reflecting differences in management practices, environmental conditions, or energetic demands before the rise of the Great Mongolian Empire.

INTRODUCTION

Serum biochemistry parameters are tightly linked to metabolic processes as well as to the physiological and health status in both humans and animals. In humans, they not only provide key indicators for routine disease diagnosis[1,2] and treatment monitoring but also for risk[3,4] and prognosis assessment.[5,6] Furthermore, serum biochemistry is key for monitoring energy metabolism in animals, and provides important predictors of performance capacity,[7] production efficiency,[8] and immune function.[9]

Previous work, including genome-wide association studies (GWASs) targeting blood biochemical traits, has identified numerous genetic loci significantly associated with human diseases.[6,10–12] In comparison, similar research in non-human animals remains limited, although recent work has started to reveal important serum-related traits and their genetic basis. For example, multi-omic studies in racing horses such as Throughbreds[13,14] and Yili horses[15,16] identified plasma metabolites strongly associated with performance traits, with training and prolonged endurance exercise profoundly reshaping lipid composition, energy, and protein metabolic pathways.[17–19] Shifts in metabolic profiles have also been associated with adiposity and metabolic syndromes, including bile acids segregating with horses showing higher metabolic risk,[20] and genome-wide analyses incorporating metabolite phenotypes from equine metabolic syndrome cohorts have provided evidence for a heritable component of metabolic traits.[21] Despite such advances, and the growing list of loci associated with serum biochemistry traits in species such as duck[22–24] and chicken,[25] precise characterization of the biological mechanisms linking genetic variation and metabolic traits is largely lacking, leaving their broader relevance and evolutionary history unclear.

In this study, we show that butyrylcholinesterase (BChE) plays a pivotal role in horse lipid metabolism, and that a high-activity variant of the *BCHE* gene has experienced shifting selection regimes in Asia over the last ~4,000 years. We measured 21 serum

© 2026 The Authors. Published by Elsevier Inc.
This is an open access article under the CC BY license (http://creativecommons.org/licenses/by/4.0/).

biochemistry parameters and sequenced the genomes of 298 Chinese horses to assess genotype-phenotype association. GWAS identified *BCHE*, which encodes the BChE enzyme, as the most significant locus associated with BChE activity, as one of the 21 serum parameters measured. We also identified a strong correlation between BChE activity and body conformation traits. Lipidomic profiling and cellular assays confirmed that higher BChE activity improves the efficiency of lipid catabolism, thereby establishing a mechanistic link between the *BCHE* gene, BChE enzymatic function, and lipid metabolism.

Beyond advancing understanding of the central role that *BCHE* plays in lipid hydrolysis and fat storage in horses, our findings add important insights to the history of horse management and breeding, following their initial domestication in the Pontic-Caspian steppes in the second half of the third millennium BCE,[26,27] and their further differentiation into local breeds or landraces adapted to specific activities and environments.[28] In particular, the contrasted evolutionary trajectories of the low- and high-activity *BCHE* variants within and outside Asia suggest different responses to distinct environmental pressures. The signature of positive selection detected in Asia for the "C" allele, which limits hepatic adiposity and overall fat storage, may reflect local adaptation to the energetic constraints of steppe ecosystems, where water limitation and strong seasonality not only impose relatively low and fluctuating energy availability but also favor metabolic efficiency under highly mobile pastoral lifeways.[29]

RESULTS

Genome sequencing and population structure

We collected blood samples from 298 horses representing eight Chinese domestic horse breeds, including Debao (DeBa), Ningqiang (NiQi), Hequ (HeQu), Yushu (YuSh), Guanzhong (GuZh), Kazakh (HSK), Yili (YiLi), and Yanqi (YaQi) horses (Figure 1A; Table S1; STAR Methods). Whole-genome sequencing was performed on the BGISEQ platform, yielding an average depth-of-coverage of ~10.62× per individual (Median = 10.13×, range = 4.55–18.67×; Table S2). Our dataset presents, for the first time, genome-wide data from the YuSh horse, a remote breed, native to extreme high altitude (~5,000 m above sea level).

After applying stringent quality control to the genome-wide dataset of 298 Chinese horses, we retained 19,878,270 high-quality SNPs for downstream analysis. Principal component analysis (PCA) and population structure analysis revealed four major genetic clusters, including Southern China ponies (SC; DeBa and NiQi), as well as horses from Northern China (NC; HSK, YaQi, and YiLi), the Qinghai-Tibetan (QT) plateau (HeQu and YuSh), and GuZh (Central China [CC]) horses (Figures 1B and S1A–S1C). These clusters are consistent with previous reports and highlight the genetic differentiation of these horse populations and regional adaptation.[30,31]

YuSh horses clustered closely with HeQu horses, another breed originating from the QT plateau, suggesting a shared evolutionary history shaped by adaptation to high-altitude environments. In contrast, the GuZh horse did not group with the NC cluster, despite its geographic location in NC. It formed instead a separate and genetically distinct lineage (Figure 1B). This divergence aligns with GuZh breeding history, which was derived as mixed ancestries from Budyonny (Russia) and Cleveland Bay (UK) horses (China National Commission of Animal Genetic Resources, 2011). GuZh horses also generally exhibited longer runs of homozygosity (ROHs) compared with other Chinese native breeds (Figures S1D–S1F), indicating lower genetic diversity and greater recent inbreeding levels.

Combined, our results suggest that the genetic structure of modern Chinese horse breeds has been shaped by both natural geographical isolation and human-mediated admixture.

Serum biochemistry traits

Serum biochemistry indicators are blood-derived parameters closely linked to physiological processes and are often considered direct reflections of metabolic activity. The 21 serum biochemistry parameters measured in 298 Chinese horses (Table S3; STAR Methods) showed substantial variation, with coefficients of variation ranging from 14.93% for calcium concentration (Ca^{2+} to 117.92% for myoglobin concentration [MYO]) (Figures S2 and S3).

PCA revealed distinct patterns of variation among the measured parameters (Figure S4). The first two principal components explained 38.3% of the total variance (Figures 1C and 1D), indicating substantial dimensionality reduction. Pairwise correlation analysis based on variable contributions identified four distinct clusters, suggesting the presence of functional interdependencies amongst subsets of parameters, independent of sex (Figures 1E and S5).

Among these subsets, the two transaminases measured (aspartate aminotransferase [AST] and alanine amino transferase [ALT]) showed a strong positive correlation (p value = 2.20e−16), consistent with their shared role in hepatic metabolism (Figure 1E). Similarly, the myocardial enzymes creatine kinase (CK), lactate dehydrogenase (LDH), and α-hydroxybutyrate dehydrogenase (HBDH) were highly correlated (p values < 6.44e−16), and showed a negative relationship with blood glucose levels (Figure 1E), reflecting a metabolic trade-off between energy demand and glucose utilization.

Our analyses also revealed a distinct lipid-associated cluster, comprising triglycerides (TG), cholesterol (CHOL), high-density lipoprotein cholesterol (HDL-c), and low-density lipoprotein cholesterol (LDL-c), as well as albumin (ALB) and the albumin/globulin ratio (A/G) (p values < 2.56e−7) (Figure 1E). Interestingly, BChE showed a significant positive correlation with HDL-c (p value = 6.99e−5), and a negative correlation with LDL-c (p value = 0.02). This pattern may reflect the complex influence of BChE on lipid metabolism, potentially through interactions with cholesterol esterase.[32] Similar associations have been reported in other mammalian species,[23,25,33,34] suggesting a conserved biochemical mechanism across taxa.

GWAS top-candidate locus for serum biochemistry traits

To investigate the genetic basis of serum biochemical variation, we conducted a *meta*-GWAS on 21 serum biochemistry traits in 298 horses. Ten traits exhibited well-calibrated QQ plots, with no significant inflation, indicating robust association signatures (Table S4). These traits included BChE, AST, alkaline

CellPress
OPEN ACCESS

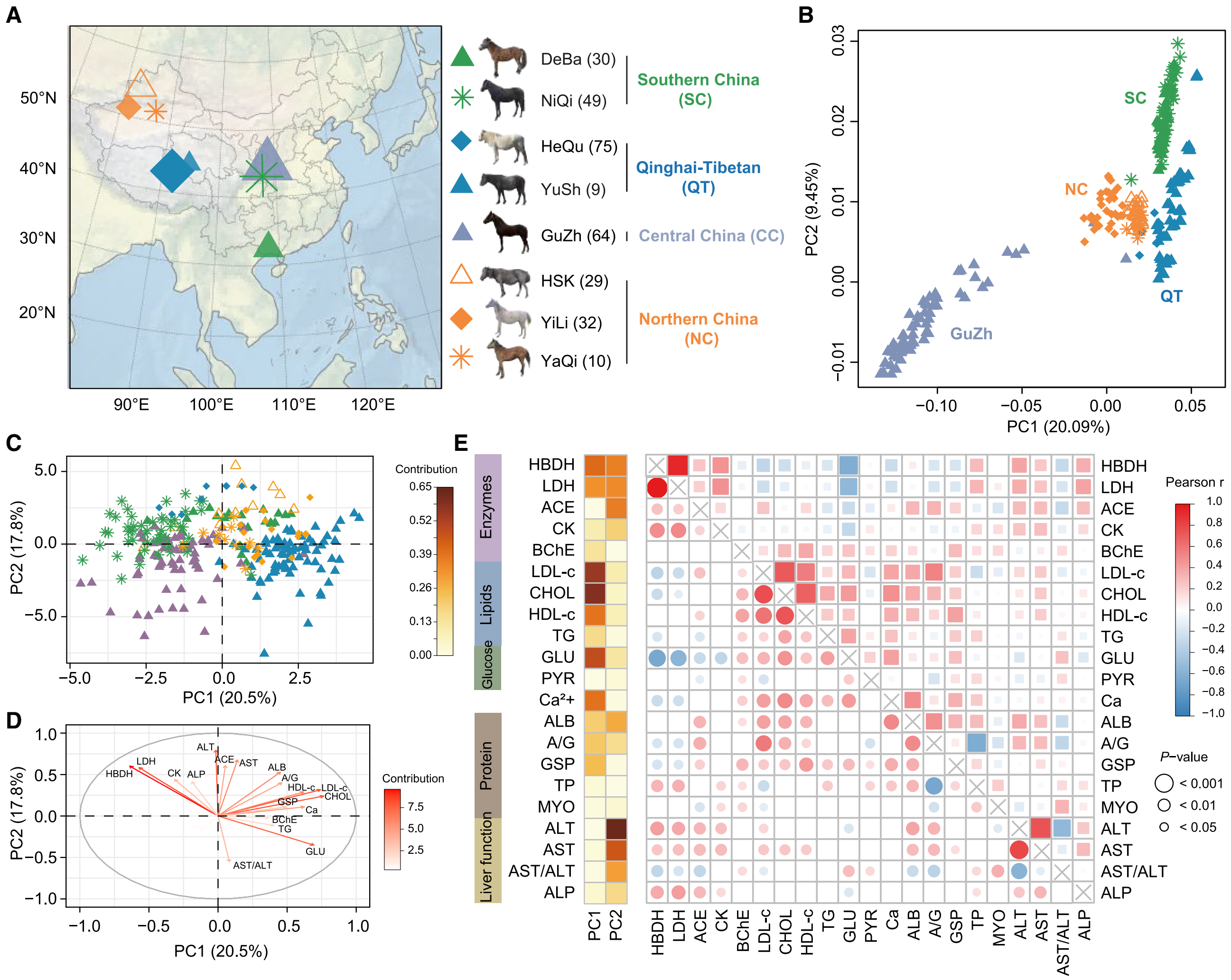

Figure 1. Integrated overview of the genetic background and molecular phenotype associations in Chinese horse breeds

(A) Sample map, with symbols and colors reflecting horse breeds from across China. SC, Southern China; QT, Qinghai-Tibetan plateau; CC, Central China; NC, Northern China; DaTo, DaTong; DeBa, Debao; GuZh, Guanzhong; HeQu, Hequ; HSK, Kazakh; NiQi, NiQiang; YaQi, YanQi; YiLi, YiLi; YuSh, YuShu.

(B) Principal component analysis (PCA) of autosomal SNP variation. The fraction of the total variance explained is reported between parentheses on each principal component.

(C) PCA of 21 molecular phenotypes, with vectors indicating the contribution of each phenotype to the first two principal components. A/G, globulin ratio; ACE, angiotensin-converting enzyme activity (U/L); ALB, albumin concentration (g/L); ALP, alkaline phosphatase activity (U/L); ALT, alanine amino transferase activity (U/L); AST, aspartate amino transferase activity (U/L); AST/ALT, ratio of AST and ALT; BChE, butyrylcholinesterase activity (U/mL); Ca, calcium concentration (mmol/L); CHOL, cholesterol concentration (mmol/L); CK, creatine kinase concentration (U/L); GLU, glucose concentration (mmol/L); GSP, glycosylated serum protein concentration (μg/L); HBDH, α-hydroxy-butyrate dehydrogenase activity (U/L); HDL-c, high-density lipoprotein cholesterol concentration (mmol/L); LDH, lactate dehydrogenase activity (U/L); LDL-c, low-density lipoprotein cholesterol concentration (mmol/L); MYO, myoglobin concentration (μg/L); PYR, pyruvic acid concentration (mmol/L); TG, triglyceride concentration (mmol/L); TP, total protein concentration (g/L).

(D) Individual contribution of each phenotype to the first two principal components.

(E) Matrix of pairwise Pearson correlations between molecular phenotypes. The colors above the main diagonal are proportional to the correlation coefficients, while the size of the circles below the main diagonal indicate statistical significance. See also Figures S1–S5 and Tables S1–S3.

phosphatase (ALP), glucose (GLU), as well as pyruvic acid (PYR), HBDH, angiotensin-converting enzyme (ACE), TG, CHOL, and LDL-c concentrations (Figure 2A).

Overall, a total of 50 genomic loci encompassing 110 SNPs exceeded the genome-wide significance threshold (2.82e−9; Table S5). The genomic windows supported by at least three consecutive significant SNPs included nine annotated genes (Figure 2A). Among these, *PRLR*, *AGXT2*, and *SEMA5A* were associated with AST, *KLHL7* with LDL-c, *ADAMTS17*, *THSD7B*, and GSE1 with TG, while *LHFPL4* and *BCHE* were associated with BChE. The most significant association was identified on chromosome 19, within the *BCHE* gene, for BChE activity (*p* value = 1.02e−13) (Figure 2A). *BCHE* encodes the BChE, a multifunctional enzyme with key protein domains including an

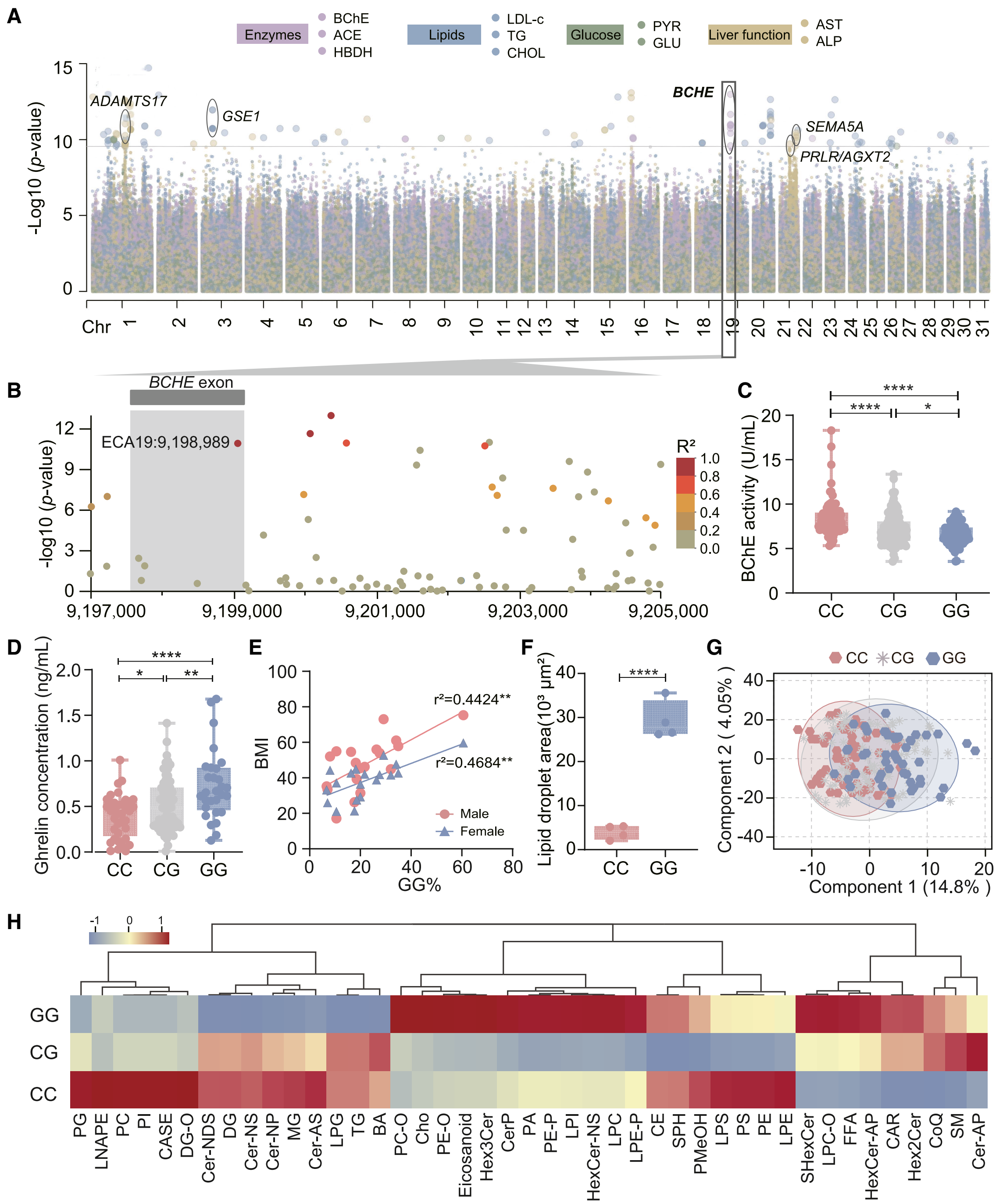

Figure 2. Statistical association between genotypes and phenotypes

(A) Manhattan plot of $-\log_{10}$(p values) reflecting the statistical association between genome-wide genetic variation and seven serum molecular phenotypes. The gray dashed line indicates the genome-wide significance level, with Bonferroni correction for multiple testing (p value = 2.82e−9). The names of the genes annotated within the 1 kb region flanking individual significant SNPs are reported. Colors indicate the main phenotype category (liver function, enzymatic activity, and lipids concentration).

(B) Zoom-in the region showing the top-significant associated SNPs, with linkage disequilibrium (LD) calculated within 500 kb flanking regions. The first two exons of *BCHE* are represented by gray boxes. The significant SNP located in the second exon (ECA19:9,198,989, G > C) is annotated.

(C) BChE activity (U/mL) in horse serum for the three genotypes at ECA19:9,198,989. The p values of Kruskal-Wallis tests indicate the level of statistical significance.

(D) Same as (C), for ghrelin concentration (mg/mL).

(E) Pearson linear regression analysis between horse body mass index (BMI) and the frequency of the GG genotype at ECA19:9,198,989.

(legend continued on next page)

acetylcholinesterase tetramerization domain (AChE_tetra), a carboxylesterase domain (COesterase), and an α/β hydrolase fold (Abhydrolase_3) (Figure S6A). This enzyme displays diverse biological functions, such as detoxifying nerve agents,[35,36] hydrolyzing cocaine,[37,38] and is involved in research on Alzheimer's disease,[39,40] emotional disorders,[41] and lipid metabolism.[40] Given its strong association and functional relevance, *BCHE* was prioritized as the most promising candidate gene for further validation.

Fine-mapping of a functional missense variant in *BCHE*

To pinpoint the causal variant underlying the *BCHE* association signal, we conducted fine-mapping of the candidate region. A strong linkage disequilibrium (LD) block was observed within the *BCHE* gene on chromosome 19 (chr19:9,197,000–9,205,000), with three SNPs (ECA19:9,198,989, ECA19:9,199,96, and ECA19:9,200,240) exhibiting high pairwise LD ($R^2 > 0.8$; Figure 2B). Among these, ECA19:9,198,989 (G/C) consists of a missense mutation resulting in an amino acid substitution from asparagine (N) to lysine (K). The "G" allele at this position is associated with a ~20% reduction in circulating BChE activity (*p* value = 1.55e−11) (Figure 2C). Linear modeling indicated that the effect of the *BCHE* missense variant on BChE activity remained highly significant after adjustment for sex, breed, and population structure (as represented by the first three principal components) ($\beta = 1.04$, p value $= 6.54 \times 10^{-8}$) (Figure S7). No significant variant × sex interaction was detected (*p* value = 0.377) (Figure S7). Overall, the *BCHE* missense variant could explain ~9.5% of the phenotypic variance in BChE activity. Comparative sequence alignment revealed that the region surrounding the asparagine (N) or lysine (K) residue is highly conserved, with approximately 90% sequence similarity across 60 vertebrate species (Figure S6B; Table S6), underscoring its functional importance.

Interestingly, while the lysine (K) residue is ancestral and widely conserved across mammals, the horse reference genome, which derives from a Thoroughbred individual,[42] carry the derived "G" allele, encoding an asparagine (N) residue. Structural modeling using TrRosetta[43–45] predicted significant conformational differences, with the *BCHE* protein containing the K residue exhibiting greater predicted structural stability (Figures S8 and S9).

Previous studies have shown that the BChE enzyme modulates ghrelin concentration by cleaving its octanoyl group, leading to change in appetite and impacting fat metabolism and deposition.[46,47] To explore this functional role in horses, we contrasted circulating ghrelin levels in blood across *BCHE* genotypes. Horses with the GG genotype (low BChE activity) showed significantly elevated ghrelin levels compared with other genotypes (*p* value = 9.67e−9), a trend consistent across multiple breeds (Figures 2D and S10). Combined, these results pointed to ECA19:9,198,989 as a strong candidate causal variant influencing both lipid metabolism and energy regulation in horses.

BChE activity and lipid-associated traits

Previous studies in humans have shown that BChE activity is associated with lower weight and body mass index (BMI).[48] To explore the physiological effects of the G > C *BCHE* K > N missense variant (ECA19:9,198,989) in horses, we explored the presence of possible correlations between its frequency and body conformation traits (Table S7). Regardless of sex, the allele "G" was significantly and positively corrected with BMI, body weight, height, length, and chest circumference ($r^2 = 0.2867$–0.4824, p values < 0.05) (Figures 2E and S11). These findings are consistent with previous observations in mice[49,50] and humans,[48] suggesting a conserved functional role for *BCHE* in regulating body size and energy storage.

Oil red O staining of liver sections further revealed that horses homozygous for the low-activity BChE allele ("GG"-genotype, encoding for the N *BChE* residue) accumulated more lipid droplets than those homozygous for the high-activity allele ("CC"-genotype, encoding for the K *BChE* residue) (Figures 2F and S12). This suggested a role for BChE in hepatic lipid metabolism in horses.

To investigate the impact of BChE on systemic lipid metabolism, we performed lipidomic profiling on serum samples from 214 horses, including 64 CC-genotype, 102 CG-genotype, and 48 GG-genotype individuals. A total of 1,515 lipid species were identified and quantified (Table S8). These lipids were classified into six major categories: glycerophospholipids (GP, 53.5%), glycerolipids (GL, 24.4%), sphingolipids (SP, 14.0%), fatty acyls (FA, 5.7%), sterol lipids (ST, 2.3%), and prenol lipids (PR, 0.1%), and further subdivided into 21 subclasses (Figure S12). We next calculated the coefficients of variation (CVs) and narrow-sense heritability (h^2) for all 1,515 lipids, leveraging associated whole-genome sequence data (Table S9). More than 65.5% lipids had CVs greater than 50%, while 77.0% showed h^2 above 0.5, indicating substantial individual variability and a strong genetic component in lipid regulation.

Orthogonal partial least square-discriminant analysis (OPLS-DA) revealed a clear separation between the lipidomic profiles of GG- and CC-genotype carriers (Figure 2G), while hierarchical clustering showed that the lipidomic profiles of CG heterozygous carriers were more similar to those of the CC-homozygous carriers than to GG-homozygous carriers (Figure 2H). These patterns suggest that the *BCHE* missense variant exerts a significant influence on lipid composition in horse serum.

Univariate analysis identified 93 lipid species that differed significantly between BCHE genotypes (p value < 0.05, VIP > 1 and $|\text{fold change}| \geq 1.2$) (Table S10). In GG-genotype horses, triacylglycerol (TG), phosphatidylcholine (PC), phosphatidylethanolamine (PE), phosphatidylserine (PS), and acylcarnitine (Cx:y) levels were significantly elevated, while ceramides (CER) and hexosylceramides (HexCer) were significantly reduced (p values < 0.05) (Figure S14). Specific lipid species, such as PC

(F) Lipid droplet area (10^2 μm^2) in seven homozygous horses at ECA19:9,198,989. Lipid droplet area is measured following Oil Red O staining of horse liver.
(G) PCA of blood relative concentrations for 1,515 lipids. Ellipses indicate the 95% confidence interval of each individual genotype at ECA19:9,198,989.
(H) Hierarchical clustering heatmap of blood relative concentrations for 1,515 lipids, grouped within 43 subclasses. Relative concentrations are z scored transformed, with zero values representing average values. Scores equal to (minus) one, thus, indicate values within one standard deviation above (below) the average. Data are represented as mean ± SD unless otherwise indicated. See also Figures S6 and S15; Tables S4–S10. Because the C allele is the main allele of interest in this study, genotypes are displayed as CC, CG, and GG in genotype-comparison panels.

(18:0_24:5), PC (O-16:1_22:6), PE (19:2_22:5), TG (16:0_20:0_22:6), and PE (P-17:0_22: 6), were significantly less abundant in CC-homozygous horses than in the other two genotype groups (Figure S14).

Least absolute shrinkage and selection operator (LASSO) regression analysis of the 93 differential lipids selected 15 candidate lipids with non-zero coefficients (Figure S15A). Subsequent Spearman correlation analysis highlighted six lipids (HexCer (d19:1/23:0), Cer (t17:2/35:1), Cer (t17:2/35:0), PC (18:0_22:5), PG (21:1_18:2), and Carnitine C6:0) that were strongly associated with BChE activity (Figures S15B–S15H), representing robust candidate biomarkers with potential biological relevance.

Collectively, our results suggest that *BCHE* genetic variation is associated with changes in BChE activity, leading to alterations in lipid composition and accumulation, especially marked for the six candidate biomarkers. This highlights the importance of BChE in lipid metabolism in horses.

Functional validation of the *BCHE* causative variant

To further elucidate the functional role of BChE in lipid metabolism, we carried out a series of experiments using both cell lines and a mouse model. We first constructed Flag-tagged expression plasmids carrying either the "C" (encoding for the N residue) or "G" (encoding for the K residue) allele at the equine BCHE missense variant (ECA19:9,198,989) (Figure S16; STAR Methods), and transfected them individually into HEK293T and HepG2 cells.

Western blot analyses revealed that the "G" allele resulted in significantly reduced BChE protein levels (66.59% in HEK293T, and 36.70% in HepG2), compared with the "C" allele (p values < 0.05) (Figures 3A, 3B, 3D, and 3E). BChE enzymatic activity was also markedly reduced: cells transfected with the Flag-tagged expression plasmids carrying the "G" allele showed an 11.05% (HEK293T) and 78.60% (HepG2) reduction in BChE activity relative to those transfected with plasmids carrying the "C" allele only (p values < 0.01, Figures 3C–3F). These results confirm that the "G" allele leads to both decreased protein levels and impaired enzymatic function.

Given the established association between BChE and lipid metabolism, we next induced lipid accumulation in HepG2 cells and performed Oil Red O staining to quantify lipid droplets. Cells expressing the "G" allele (K residue) exhibited a denser accumulation of lipid droplets compared with those expressing the "C" allele (N residue), although still less than the empty vector control (Figure 3G). Consistently, intracellular TG levels, a direct indicator of the cellular lipid content, were significantly higher in HepG2 cells expressing the "G" allele than in those expressing the "C" allele (p values = 6.50e−3), but lower than in the control (p values = 0.01) Figure 3H). Increased levels of lipid droplet-associated protein *PLIN1* in cells expressing the "G" allele further supported lipid accumulation (Figure S17).

We then assessed the expression of genes involved in lipid anabolism and catabolism. Cells expressing the "C" allele displayed significantly lower expression of the *de novo* lipidogenesis genes *ACLY* and *FASN* (p value < 0.05) (Figures S18A and S18B), suggesting that higher BChE activity reduces, or suppresses, lipogenesis. However, expression of key lipolytic enzymes, such as adipose triglyceride lipase (*ATGL*), hormone-sensitive lipase (*HSL*), and monoacylglycerol lipase *(MGL*), was not significantly altered in any group (Figures S18C and S18D). Despite this, co-localization of BChE with these enzymes was observed (Figure S19A), and structural similarity analyses indicated that BChE shares conserved domains with classical lipases (Figure S19B). This suggests that BChE may act in concert with canonical lipolytic enzymes to promote lipid mobilization.

The transfection assays above demonstrate that the *BCHE* missense variant compromises BChE protein levels and enzymatic activity, thereby impairing its lipid-hydrolyzing capacity and promoting lipid accumulation. To validate these findings *in vivo*, we generated recombinant AAV9 vectors designed to express the "C" or "G" allele in the liver, and administered them via tail vein injection to 8-week C57BL/6J mice (1.0×10^{13} vg/mouse). Empty vectors were injected as controls. Mice were then monitored weekly for food intake and body weight until 28 weeks of age, and showed no significant differences among the groups (Figure S20).

Mice injected with vectors carrying the "C" allele showed significantly higher *BCHE* transcription in the liver than those injected with vectors carrying the "G" allele (p values < 0.05) (Figures 4A–4E; STAR Methods). Concurrently, BChE activity was significantly enhanced in both the liver (p value = 0.04) and serum (p value = 0.02) (Figures 4A–4F). Oil Red O staining of liver sections revealed the greatest lipid accumulation in the liver of control mice, followed by those injected with vectors carrying the "G" allele, and least in those injected with vectors carrying the "C" allele. These differences were also mirrored in the morphology and color of the liver (Figures 4G and 4H). Moreover, mice injected with vectors carrying the "C" allele had significantly lower fat mass (Figure 4I), and smaller adipocytes (Figures 4J and 4K) compared with those injected with vectors carrying the "G" allele or controls. These *in vivo* results corroborate our *in vitro* findings, confirming that BChE plays a critical role in lipid hydrolysis and fat mobilization. The *BCHE* missense variant (G > C, leading to a K > N amino acid substitution) impairs these functions by reducing both the enzyme levels and activity, leading to increased lipid accumulation and adiposity.

Selection at the *BCHE* locus

Given the strong association between *BCHE* variation and lipid metabolism, we next examined the selection history of this locus during horse domestication. We first estimated the frequency of the "C" allele at position ECA19:9,198,989 in a global panel of 1,238 modern horses (Table S11). While the "C" allele appears fixed in the Przewalski's horse lineage, it reaches a worldwide frequency of 52.79% in modern domesticated horses (DOM2), with continental estimates of 54.04% in Asia, and 48.38% outside Asia. These values, obtained from both KASP genotyping (N = 541) and genotyping-by-sequencing (N = 736), were consistent with frequencies deduced from a previously published panel of horse genomes[28] (Figure S21; Table S12).

To gain understanding of frequency shifts over time, we analyzed an extensive genome time-series including 311 ancient horse specimens for this locus (Figures 5A–5C), and modeled temporal trajectories of the "C" allele within and outside Asia using CLUES2.[51] Outside Asia, the observed temporal frequency changes were compatible with neutral drift ($-\log_{10}p$

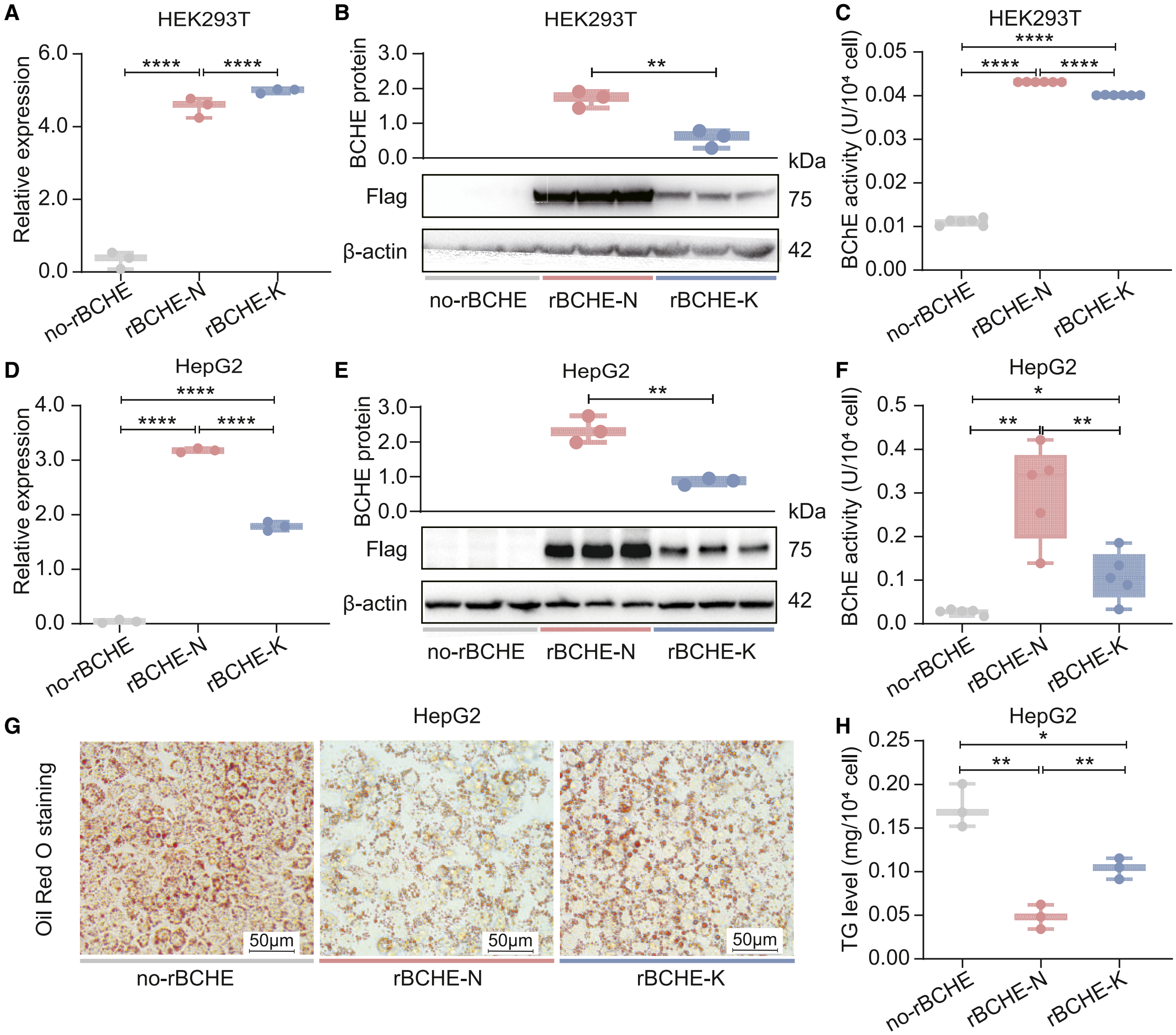

Figure 3. Overexpression of BCHE gene promotes lipolysis in HEK293T and HepG2 cells

(A) Over-expression of BCHE mRNA in HEK293T cells. Vector indicates the control expression level relative to GAPDH expression, while C and G reflect the fold expression increase in the presence of vectors carrying the two BCHE gene variants identified at ECA19:9,198,989.

(B) Western blot analysis and ImageJ quantification of BCHE protein levels. The levels of recombinant proteins were measured using anti-Flag antibodies, and normalized relative to β-actin levels and BCHE transcription levels.

(C) Normalized enzymatic activity of recombinant BChE in HEK293T cells. Activity was normalized by the amount of recombinant BChE.

(D) Same as (A) in HepG2 cells.

(E) Quantification of recombinant BCHE protein levels in HepG2 cells.

(F) Same as (C) in HepG2 cells.

(G) Oil red O staining after adipogenic induction of HepG2 cells transfected with recombinant vectors. Scale bars, 50 μm.

(H) Triglyceride (TG) levels in HepG2 cells transfected with recombinant vectors. Data are represented as mean ± SD unless otherwise indicated. Statistical significance was assessed using Kruskal-Wallis tests (*p value < 0.05; **p value < 0.01; ***p value < 0.001; ****p value < 0.0001). See also Figures S16–S19. Because the C allele encodes the BChE-N form, recombinant BChE groups are displayed as no-rBChE, rBChE-N, and rBChE-K.

value = 0.12), based on the global demographic model presented by Liu and colleagues. In contrast, neutral evolution could be rejected within Asia ($-\log_{10}p$ value = 2.58) (Table S13). Here, the "C" allele appears to have undergone positive selection from the earliest stages of DOM2 expansion outside their domestication center and until ~95–105 generations ago (i.e., ~777–703 years ago or ~1248–1322 Common Era, assuming an average generation time of 7.4 years[27]), when it shifted to neutrality.

We further validated these findings by repeating our analyses to additional demographic models: the subcontinental models

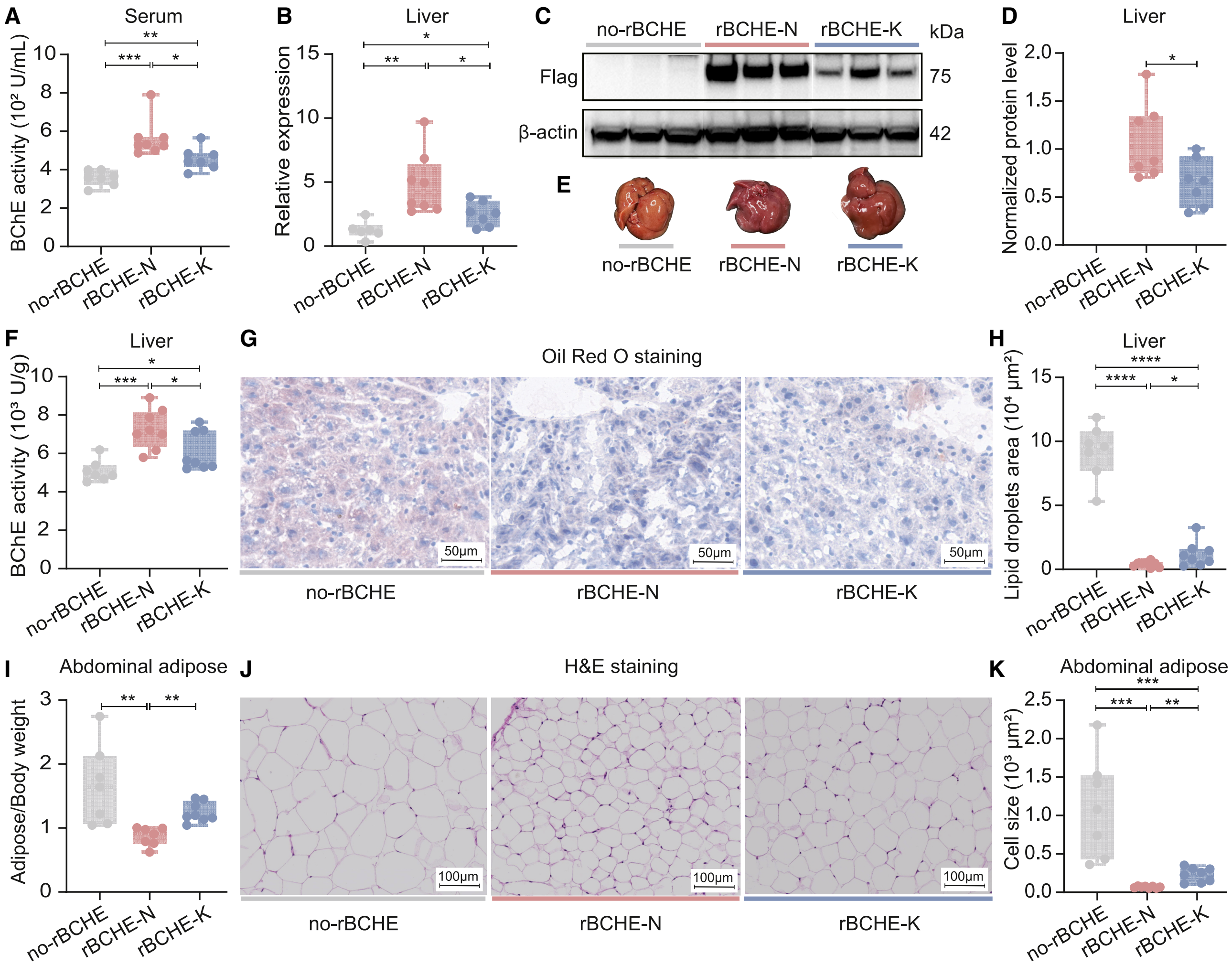

Figure 4. Overexpression of BCHE gene promotes lipolysis in mice

(A) Serum BChE activity (10^2 U/mL) in mice injected by three different recombinant vectors (G and C vectors carry the two BCHE gene variants identified at ECA19:9,198,989, while 'vector' indicates empty, control vectors.

(B) Over-expression of *BCHE* mRNA in liver.

(C) Western blot analysis of BCHE protein levels in three individuals per recombinant vector.

(D) Entire livers of three individuals injected by different recombinant vectors.

(E) ImageJ quantification of BCHE protein levels, normalized relative to β-actin levels and BCHE transcription levels.

(F) Liver BChE activity (10^2 U/mL).

(G) Oil red O staining of liver sections following injections by three different recombinant vectors. Scale bars, 50 μm.

(H) Lipid droplet area (10^4 μm^2) measured following Oil Red O staining of mice livers.

(I) Abdominal adipose content, relative to body mass.

(J) H&E staining of abdominal adipose tissue. Scale bars, 100 μm.

(K) Cell size (10^4 μm^2) of adipose tissue. Data are represented as mean ± SD unless otherwise indicated. Statistical significance was assessed using Kruskal-Wallis tests (**p* value < 0.05; ***p* value < 0.01; ****p* value < 0.001; *****p* value < 0.0001). See also Figure S20. Because the C allele encodes the BChE-N form, recombinant BChE groups are displayed as no-rBChE, rBChE-N, and rBChE-K.

presented by Liu et al.[28] for Asian and non-Asian horses, and the global model presented by Der Sarkissian et al.[52] for the DOM2 lineage. The results remained consistent across all models (Figures 5D–5G, S22, and S23; Table S13), providing robust evidence that positive selection was specific to Asia.

We next leveraged a complementary statistical framework based on particle marginal Metropolis-Hastings (PMMH),[53] to estimate selection coefficients before and after 105 generations ago. We first assumed recessivity for the "C" allele, since CLUES2[51] Log likelihood was maximized under a recessive model ($h = 0$). This analysis confirmed a shift from positive selection ($S^- = 0.83\%$, 95% confidence interval [CI] = 0.17%–1.50%) to neutrality in Asia, with a selection coefficient intercepting zero (95% CI = −11.76%–3.41%) from 105 generations ago to the present day (Figures 5D–5G, S22, and S23). The same pattern held when relaxing the recessivity assumption and allowing for

CellPress
OPEN ACCESS

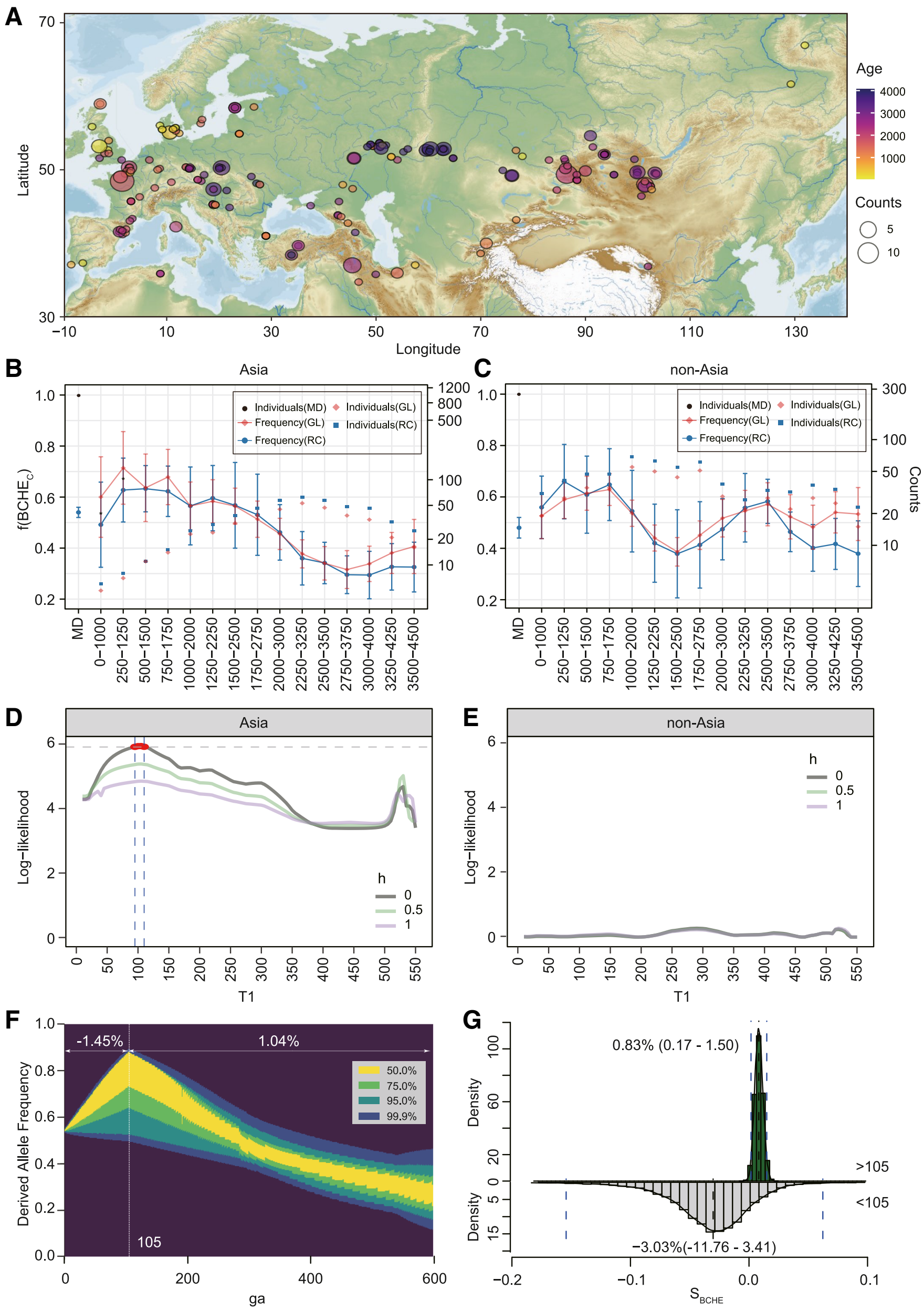

Figure 5. Selection history of the BCHE “C” allele

(A) Map of ancient horses analyzed in this study. The color gradient reflects time (radiocarbon dates or archaeological contexts).

(B) Temporal allele trajectory within Asia.

(C) Temporal allele trajectory outside Asia. Allele frequencies are estimated using read counts (RC), or genotype likelihoods (GL), from the corresponding numbers of individuals showing sufficient sequence coverage. The left vertical axis represents allele frequencies, while the right vertical axis indicates the number of individuals with non-null sequence coverage.

(D) CLUES2 Log Likelihood support for the selection model relative to neutrality in Asia. Analyses were performed using a grid for the time of selection shift, considering every 5 generations from 5 to 600 generations ago.

(E) Same as (D), conditioning on the horses from outside Asia.

(F) CLUES2 best-model fit for the temporal allele trajectory within Asia.

(G) PMMH-based posterior distribution for the selection coefficients before and after 105 generations ago. See also Figures S21–S24 and Tables S11–S14.

any possible *h* value (S^- = 0.89%, 95% CI = 0.16%–1.61% vs. S^+ 95% CI = −38.87%–2.90%), although these analyses indicated limited power to infer inheritance mode (Figure S24; Table S14).

Combined, our analyses indicate contrasting selection regimes for the *BCHE* “C” allele within and outside Asia, reflecting distinct breeding practices and goals across continents.

Moreover, they support a shift in selection pressure within Asia around the mid-13th century CE, broadly coincident with the rise and expansion of the Great Mongol Empire and the Yuan dynasty, the first of China's Late Imperial dynasties.[54]

DISCUSSION

Morphological development and athletic performance are closely influenced by metabolic processes, with genetic factors playing a pivotal role. Certain metabolic biomarkers have long been used to predict athletic potential in horses,[55] and exercise-related traits, including aerobic capacity, are known to show close association with circulating metabolic and lipid profiles.[13,15,56] Mutations in genes such as *MSTN* are already commercially applied to forecast racehorse performance.[57] Despite this, the genetic underpinnings of metabolic regulation and the influence of lipids on organismal development and phenotypic traits remain fragmentary.

To address this knowledge gap, we collected and analyzed serum biochemistry data from representative domestic horse breeds across China. By integrating whole-genome sequencing with meta-analysis of serum biochemical parameters, we identified a G > C missense mutation (ECA19:9,198,989) in the *BCHE* gene that is significantly associated with BChE activity and body conformation traits in horses. Because body conformation is an important determinant of locomotor and performance-related traits in horses, the observed association between the *BCHE* variant and body conformation provides a basis for future studies integrating *BCHE*-associated metabolic variation with standardized performance or exercise-related phenotyping.

GWAS revealed that the top association with BChE activity was at the *BCHE* locus, with the missense variant emerging as a likely causal mutation. BChE has traditionally been recognized for hydrolyzing substrates such as acetylcholine, organophosphates, and cocaine, and is involved in the treatment of organophosphate poisoning and Alzheimer's disease.[40,58] More recent research in mice has suggested that BChE may regulate body weight as well as insulin secretion and sensitivity by hydrolyzing ghrelin.[41,49] Our study expands on these findings by demonstrating the role of BChE in lipid deposition and hydrolysis in horses (Figure 3), in line with previous work in humans.[48]

The "C" allele is significantly associated with increased BChE enzymatic activity and decreased circulating levels of ghrelin and lipid content (Figure 4). Functional assays in cell lines and lipidomic analyses of serum confirmed the role of BChE in promoting lipid hydrolysis (Figure 5), with higher enzymatic activity correlating with more efficient lipid breakdown. While previous research proposed a BChE-ghrelin regulatory pathway, our findings provide evidence that BChE contributes to lipid metabolism, underscoring its broader physiological significance beyond ghrelin modulation. Remarkably, the specific activity and expression yield of equine BChE exceeds those reported in primates, pigs, birds, dogs, and mice,[59] highlighting the potential of horse-derived BChE for translational research and therapeutic applications. These findings open new avenues for exploring equine BChE in regulation and drug development.

Interestingly, while the "C" allele evolved under neutrality in Europe following the expansion of DOM2 horses beyond their domestication center, it was subject to positive selection in Asia until the mid-13th century CE. When considered alongside its functional consequences, this pattern points to divergent breeding goals and environment contexts in the two continents. In Asia, selection favored allele carriers with increased BChE enzymatic activity and decreased circulating levels of ghrelin and lipids, potentially reflecting distinct management practices and/or dietary regimes, possibly shaped by the predominance of steppe ecosystems. These contrasting selection regimes disappeared during the mid-13th century, after which Asian horse populations evolved under neutrality. Future work, including *BCHE* genotyping from high-resolution time series, will be required to determine whether this shift was triggered by the expansion of the Great Mongolian Empire, which formed a transcontinental horse-based nomad empire across Eurasia,[54] or by other major historical events, such as the rise of Late Imperial dynasties in China.

Limitations of the study

This study integrates genomic, biochemical, lipidomic, cellular, mouse-model to demonstrate the regulatory role of *BCHE* in equine lipid metabolism. Several limitations, however, remain to be addressed. First, larger cohorts with standardized metadata regarding athletic performance, feeding management, diet, and living environments are required to validate the associations beyond those Chinese horse breeds investigated. Second, although our functional assays confirm that the *BCHE* missense variant modulates BChE enzymatic activity and cellular lipid accumulation, the downstream mechanisms linking BChE function and lipid metabolism regulation remain further unexplored. Third, while the BCHE locus exhibits signatures of region-specific selection in Asian horse populations, the exact adaptive phenotype under selection remains uncharacterized. Accordingly, the phenotypic divergence between Asian and non-Asian horses cannot be directly attributed to variations in lipid metabolism. Further studies incorporating dense temporal sampling and multi-dimensional data covering phenotypic traits, archaeological records, environmental conditions, and breeding management contexts are needed to disentangle the distinct selective pressures acting on the *BCHE* locus and the exact context driving the shifting selection regimes detected.

RESOURCE AVAILABILITY

Lead contact

Further information and requests for resources and reagents should be directed to and will be fulfilled by the lead contact, Lin Jiang (jianglin@caas.cn).

Materials availability

No new, unique reagents were generated in this study.

Data and code availability

All whole-genome sequencing reads generated in this study have been deposited in the Sequence Read Archive (BioProject: PRJNA1074421). All other data supporting the findings of this study are available throughout the paper and its supplemental information. This paper does not report original code.

ACKNOWLEDGMENTS

This work was supported by the Fundamental Research Fund of CAAS (grant nos. CAAS-CSAB-202402 and Y2025YC50) and National Natural Science

Foundation of China (grant no. 32222079). L.O. was supported by the European Research Council (HorsePower-101071707).

AUTHOR CONTRIBUTIONS

L.J. and Y.Z. conceived and designed the study. Y.Z., Y.P., Y.M., and L.J. contributed horse samples and genomic data. Y.Z. measured and analyzed the serum biochemistry parameters data. Y.Z. and X.L. analyzed modern genome data. X.L. and L.O. analyzed ancient genome data. Y.Z. and X.W. performed cell expression analyses. Y.Z. and X.W. conducted metabolome data analysis. Y.Z., Y.J., and Y.Y. performed mouse feeding and validation experiments. Y.Z., X.L., L.O., and L.J. wrote the manuscript. All authors discussed the results and the manuscript.

DECLARATION OF INTERESTS

The authors declare no competing interests.

STAR★METHODS

Detailed methods are provided in the online version of this paper and include the following:

- KEY RESOURCES TABLE
- EXPERIMENTAL MODEL AND STUDY PARTICIPANT DETAILS
  - Horse samples
  - Mice
  - Cell lines
- METHODS
  - Whole-genome sequencing
  - Population structure analysis
  - Runs of homozygosity and LD-decay
  - Serum biochemistry parameters
  - Principal component analysis (PCA) and correlation analysis of serum biochemical parameters
  - Genome-wide association study
  - Linear modeling of BCHE variant effects
  - Serum ghrelin
  - Serum lipid profiling
  - Coefficients of variation and heritability
  - Least absolute shrinkage and selection operator (LASSO) regression analysis
  - Plasmid construction
  - Cell transfection
  - Western blot analysis
  - Adipogenic induction and Oil Red O staining
  - Intracellular triglyceride (TG) content
  - Immunofluorescence (IF) staining
  - Construction of *BCHE* overexpression mouse model
  - RT-qPCR experiments
  - Histological staining
  - Validation in the modern horse populations
  - Selection analyses
- QUANTIFICATION AND STATISTICAL ANALYSIS

SUPPLEMENTAL INFORMATION

Supplemental information can be found online at https://doi.org/10.1016/j.celrep.2026.117879.

Received: February 11, 2026
Revised: June 15, 2026
Accepted: August 3, 2026
Published: August 20, 2026

REFERENCES

1. Pervaiz, S., Anderson, F.P., Lohmann, T.P., Lawson, C.J., Feng, Y.J., Waskiewicz, D., Contois, J.H., and Wu, A.H. (1997). Comparative analysis of cardiac troponin I and creatine kinase-MB as markers of acute myocardial infarction. Clin. Cardiol. *20*, 269–271. https://doi.org/10.1002/clc.4960200316.

2. Halbgebauer, S., Steinacker, P., Verde, F., Weishaupt, J., Oeckl, P., von Arnim, C., Dorst, J., Feneberg, E., Mayer, B., Rosenbohm, A., et al. (2022). Comparison of CSF and serum neurofilament light and heavy chain as differential diagnostic biomarkers for ALS. J. Neurol. Neurosurg. Psychiatry *93*, 68–74. https://doi.org/10.1136/jnnp-2021-327129.

3. Long, M.T., Pedley, A., Colantonio, L.D., Massaro, J.M., Hoffmann, U., Muntner, P., and Fox, C.S. (2016). Development and Validation of the Framingham Steatosis Index to Identify Persons With Hepatic Steatosis. Clin. Gastroenterol. Hepatol. *14*, 1172–1180.e1172. https://doi.org/10.1016/j.cgh.2016.03.034.

4. Kim, J.W., Lee, K.J., Yang, H.R., Chang, J.Y., Moon, J.S., Khang, Y.H., and Ko, J.S. (2018). Prevalence and risk factors of elevated alanine aminotransferase among Korean adolescents: 2001-2014. BMC Public Health *18*, 617. https://doi.org/10.1186/s12889-018-5548-9.

5. Zhang, Y., Liu, M., Zhang, C., Zou, Y., Kang, L., and Song, L. (2024). Role of Biomarkers of Myocardial Injury to Predict Adverse Outcomes in Hypertrophic Cardiomyopathy. Circ. Cardiovasc. Qual. Outcomes *17*, e010243. https://doi.org/10.1161/circoutcomes.123.010243.

6. Mathey, C.M., Maj, C., Eriksson, N., Krebs, K., Westmeier, J., David, F.S., Koromina, M., Scheer, A.B., Szabo, N., Wedi, B., et al. (2024). Meta-analysis of ACE inhibitor-induced angioedema identifies novel risk locus. J. Allergy Clin. Immunol. *153*, 1073–1082. https://doi.org/10.1016/j.jaci.2023.11.921.

7. Monteverde, V., Congiu, F., Vazzana, I., Dara, S., Di Pietro, S., and Piccione, G. (2017). Serum lipid profile modification related to polyunsaturated fatty acid supplementation in thoroughbred horses. J. Appl. Anim. Res. *45*, 615–618. https://doi.org/10.1080/09712119.2016.1251439.

8. Sun, J., Xie, F., Wang, J., Luo, J., Chen, T., Jiang, Q., Xi, Q., Liu, G.E., and Zhang, Y. (2024). Integrated meta-omics reveals the regulatory landscape involved in lipid metabolism between pig breeds. Microbiome *12*, 33. https://doi.org/10.1186/s40168-023-01743-3.

9. Dang, E.V., and Reboldi, A. (2024). Cholesterol sensing and metabolic adaptation in tissue immunity. Trends Immunol. *45*, 861–870. https://doi.org/10.1016/j.it.2024.09.013.

10. Harrer, P., Inderhees, J., Zhao, C., Schormair, B., Tilch, E., Gieger, C., Peters, A., Jöhren, O., Fleming, T., Nawroth, P.P., et al. (2024). Phenotypic and genome-wide studies on dicarbonyls: major associations to glomerular filtration rate and gamma-glutamyltransferase activity. EBioMedicine *101*, 105007. https://doi.org/10.1016/j.ebiom.2024.105007.

11. Ghouse, J., Ahlberg, G., Andreasen, L., Banasik, K., Brunak, S., Schwinn, M., Larsen, I.H., Petersen, O., Sørensen, E., Ullum, H., et al. (2021). Association of Variants Near the Bradykinin Receptor B(2) Gene With Angioedema in Patients Taking ACE Inhibitors. J. Am. Coll. Cardiol. *78*, 696–709. https://doi.org/10.1016/j.jacc.2021.05.054.

12. Adams, C., Manouchehrinia, A., Quach, H.L., Quach, D.L., Olsson, T., Kockum, I., Schaefer, C., Ponting, C.P., Alfredsson, L., and Barcellos, L.F. (2024). Evidence supports a causal association between allele-specific vitamin D receptor binding and multiple sclerosis among Europeans. Proc. Natl. Acad. Sci. USA *121*, e2302259121. https://doi.org/10.1073/pnas.2302259121.

13. Bonhomme, M.M., Patarin, F., Kruse, C.J., François, A.C., Renaud, B., Couroucé, A., Leleu, C., Boemer, F., Toquet, M.P., Richard, E.A., et al. (2023). Untargeted Metabolomics Profiling Reveals Exercise Intensity-Dependent Alterations in Thoroughbred Racehorses' Plasma after Routine Conditioning Sessions. ACS Omega *8*, 48557–48571. https://doi.org/10.1021/acsomega.3c08583.

14. Li, C., Li, X., Liu, K., Xu, J., Yu, J., Liu, Z., Mach, N., Ni, W., Liu, C., Zhou, P., et al. (2025). Multiomic analysis of different horse breeds reveals that gut microbial butyrate enhances racehorse athletic performance. NPJ Biofilms Microbiomes *11*, 87. https://doi.org/10.1038/s41522-025-00730-w.

15. Wang, T., Meng, J., Wang, J., Ren, W., Yang, X., Adina, W., Bao, Y., Zeng, Y., and Yao, X. (2025). Absolute Quantitative Lipidomics Reveals Differences in Lipid Compounds in the Blood of Trained and Untrained Yili Horses. Vet. Sci. *12*, 255. https://doi.org/10.3390/vetsci12030255.

16. Chang, X., Zhang, Z., Yao, X., Meng, J., Ren, W., and Zeng, Y. (2025). Lipidomics and biochemical profiling of adult Yili horses in a 26 km endurance race: exploring metabolic adaptations. Front. Vet. Sci. *12*, 1597739. https://doi.org/10.3389/fvets.2025.1597739.

17. Le Moyec, L., Robert, C., Triba, M.N., Billat, V.L., Mata, X., Schibler, L., and Barrey, E. (2014). Protein catabolism and high lipid metabolism associated with long-distance exercise are revealed by plasma NMR metabolomics in endurance horses. PLoS One *9*, e90730. https://doi.org/10.1371/journal.pone.0090730.

18. Luck, M.M., Le Moyec, L., Barrey, E., Triba, M.N., Bouchemal, N., Savarin, P., and Robert, C. (2015). Energetics of endurance exercise in young horses determined by nuclear magnetic resonance metabolomics. Front. Physiol. *6*, 198. https://doi.org/10.3389/fphys.2015.00198.

19. Halama, A., Oliveira, J.M., Filho, S.A., Qasim, M., Achkar, I.W., Johnson, S., Suhre, K., and Vinardell, T. (2021). Metabolic Predictors of Equine Performance in Endurance Racing. Metabolites *11*, 82. https://doi.org/10.3390/metabo11020082.

20. Donnelly, C.G., Peng, S., Pflieger, L., Manfredi, J., Coleman, M., Rappaport, N., Price, N.D., and Finno, C.J. (2025). Bile acids segregate metabolic syndrome in a cohort of 100 deeply phenotyped horses. Commun. Biol. *8*, 1711. https://doi.org/10.1038/s42003-025-09111-7.

21. Patterson Rosa, L., Mallicote, M.F., Long, M.T., and Brooks, S.A. (2020). Metabogenomics reveals four candidate regions involved in the pathophysiology of Equine Metabolic Syndrome. Mol. Cell. Probes *53*, 101620. https://doi.org/10.1016/j.mcp.2020.101620.

22. Qi, J., Liu, H., Zhou, Z., Jiang, Y., Fan, W., Hu, J., Li, J., Guo, Z., Xie, M., Huang, W., et al. (2024). Genome-wide association study identifies multiple loci influencing duck serum biochemical indicators in the laying period. Br. Poult. Sci. *65*, 8–18. https://doi.org/10.1080/00071668.2023.2272982.

23. Tang, H., Zhang, H., Liu, D., Wang, Z., Yu, D., Fan, W., Guo, Z., Huang, W., Hou, S., and Zhou, Z. (2022). Genome-wide association study reveals the genetic determinism of serum biochemical indicators in ducks. BMC Genom. *23*, 856. https://doi.org/10.1186/s12864-022-09080-9.

24. Xu, Y., Liu, H., Jiang, Y., Fan, W., Hu, J., Zhang, Y., Guo, Z., Xie, M., Huang, W., Liu, X., et al. (2019). Genome-wide association studies reveal genetic loci associated with plasma cholinesterase activity in ducks. Anim. Genet. *50*, 287–292. https://doi.org/10.1111/age.12791.

25. Song, H., Li, W., Li, Y., Zhai, B., Guo, Y., Chen, Y., Han, R., Sun, G., Jiang, R., Li, Z., et al. (2023). Genome-wide association study of 17 serum biochemical indicators in a chicken F(2) resource population. BMC Genom. *24*, 98. https://doi.org/10.1186/s12864-023-09206-7.

26. Librado, P., Khan, N., Fages, A., Kusliy, M.A., Suchan, T., Tonasso-Calvière, L., Schiavinato, S., Alioglu, D., Fromentier, A., Perdereau, A., et al. (2021). The origins and spread of domestic horses from the Western Eurasian steppes. Nature *598*, 634–640. https://doi.org/10.1038/s41586-021-04018-9.

27. Librado, P., Tressières, G., Chauvey, L., Fages, A., Khan, N., Schiavinato, S., Calvière-Tonasso, L., Kusliy, M.A., Gaunitz, C., Liu, X., et al. (2024). Widespread horse-based mobility arose around 2200 BCE in Eurasia. Nature *631*, 819–825. https://doi.org/10.1038/s41586-024-07597-5.

28. Liu, X., Jia, Y., Pan, J., Zhang, Y., Gong, Y., Wang, X., Ma, Y., Alvarez, N., Jiang, L., and Orlando, L. (2025). Selection at the GSDMC locus in horses and its implications for human mobility. Science *389*, 925–930. https://doi.org/10.1126/science.adp4581.

29. Taylor, W.T.T., Clark, J., Bayarsaikhan, J., Tuvshinjargal, T., Jobe, J.T., Fitzhugh, W., Kortum, R., Spengler, R.N., Shnaider, S., Seersholm, F.V., et al. (2020). Early Pastoral Economies and Herding Transitions in Eastern Eurasia. Sci. Rep. *10*, 1001. https://doi.org/10.1038/s41598-020-57735-y.

30. Liu, X., Zhang, Y., Li, Y., Pan, J., Wang, D., Chen, W., Zheng, Z., He, X., Zhao, Q., Pu, Y., et al. (2019). EPAS1 Gain-of-Function Mutation Contributes to High-Altitude Adaptation in Tibetan Horses. Mol. Biol. Evol. *36*, 2591–2603. https://doi.org/10.1093/molbev/msz158.

31. Liu, X., Zhang, Y., Liu, W., Li, Y., Pan, J., Pu, Y., Han, J., Orlando, L., Ma, Y., and Jiang, L. (2022). A single-nucleotide mutation within the TBX3 enhancer increased body size in Chinese horses. Curr. Biol. *32*, 480–487.e486. https://doi.org/10.1016/j.cub.2021.11.052.

32. Santarpia, L., Grandone, I., Contaldo, F., and Pasanisi, F. (2013). Butyrylcholinesterase as a prognostic marker: a review of the literature. J. Cachexia Sarcopenia Muscle *4*, 31–39. https://doi.org/10.1007/s13539-012-0083-5.

33. Bulik-Sullivan, B., Finucane, H.K., Anttila, V., Gusev, A., Day, F.R., Loh, P.R., Duncan, L., Perry, J.R., Patterson, N., Robinson, E.B., et al. (2015). An atlas of genetic correlations across human diseases and traits. Nat. Genet. *47*, 1236–1241. https://doi.org/10.1038/ng.3406.

34. Tvarijonaviciute, A., Barić-Rafaj, R., Horvatic, A., Muñoz-Prieto, A., Guillemin, N., Lamy, E., Tumpa, A., Ceron, J.J., Martinez-Subiela, S., and Mrljak, V. (2019). Identification of changes in serum analytes and possible metabolic pathways associated with canine obesity-related metabolic dysfunction. Vet. J. *244*, 51–59. https://doi.org/10.1016/j.tvjl.2018.12.006.

35. Shenouda, J., Green, P., and Sultatos, L. (2009). An evaluation of the inhibition of human butyrylcholinesterase and acetylcholinesterase by the organophosphate chlorpyrifos oxon. Toxicol. Appl. Pharmacol. *241*, 135–142. https://doi.org/10.1016/j.taap.2009.08.014.

36. Allard, J.L., Shields, K.A., Munro, T.P., and Lua, L.H.L. (2022). Strategies for developing a recombinant butyrylcholinesterase medical countermeasure for Organophosphorus poisoning. Chem. Biol. Interact. *363*, 109996. https://doi.org/10.1016/j.cbi.2022.109996.

37. Gorelick, D.A. (1997). Enhancing cocaine metabolism with butyrylcholinesterase as a treatment strategy. Drug Alcohol Depend. *48*, 159–165. https://doi.org/10.1016/s0376-8716(97)00119-1.

38. Brimijoin, S., Gao, Y., Anker, J.J., Gliddon, L.A., Lafleur, D., Shah, R., Zhao, Q., Singh, M., and Carroll, M.E. (2008). A cocaine hydrolase engineered from human butyrylcholinesterase selectively blocks cocaine toxicity and reinstatement of drug seeking in rats. Neuropsychopharmacology *33*, 2715–2725. https://doi.org/10.1038/sj.npp.1301666.

39. Li, Q., Yang, H., Chen, Y., and Sun, H. (2017). Recent progress in the identification of selective butyrylcholinesterase inhibitors for Alzheimer's disease. Eur. J. Med. Chem. *132*, 294–309. https://doi.org/10.1016/j.ejmech.2017.03.062.

40. Xing, S., Li, Q., Xiong, B., Chen, Y., Feng, F., Liu, W., and Sun, H. (2021). Structure and therapeutic uses of butyrylcholinesterase: Application in detoxification, Alzheimer's disease, and fat metabolism. Med. Res. Rev. *41*, 858–901. https://doi.org/10.1002/med.21745.

41. Chen, V.P., Gao, Y., Geng, L., Parks, R.J., Pang, Y.P., and Brimijoin, S. (2015). Plasma butyrylcholinesterase regulates ghrelin to control aggression. Proc. Natl. Acad. Sci. USA *112*, 2251–2256. https://doi.org/10.1073/pnas.1421536112.

42. Kalbfleisch, T.S., Rice, E.S., DePriest, M.S., Jr., Walenz, B.P., Hestand, M.S., Vermeesch, J.R., BL, O.C., Fiddes, I.T., Vershinina, A.O., Saremi, N.F., et al. (2018). Improved reference genome for the domestic horse increases assembly contiguity and composition. Commun. Biol. *1*, 197. https://doi.org/10.1038/s42003-018-0199-z.

43. Du, Z., Su, H., Wang, W., Ye, L., Wei, H., Peng, Z., Anishchenko, I., Baker, D., and Yang, J. (2021). The trRosetta server for fast and accurate protein structure prediction. Nat. Protoc. *16*, 5634–5651. https://doi.org/10.1038/s41596-021-00628-9.

44. Su, H., Wang, W., Du, Z., Peng, Z., Gao, S.H., Cheng, M.M., and Yang, J. (2021). Improved Protein Structure Prediction Using a New Multi-Scale Network and Homologous Templates. Adv. Sci. *8*, e2102592. https://doi.org/10.1002/advs.202102592.

45. Wang, W., Peng, Z., and Yang, J. (2022). Single-sequence protein structure prediction using supervised transformer protein language models. Nat. Comput. Sci. *2*, 804–814. https://doi.org/10.1038/s43588-022-00373-3.

46. Brimijoin, S., Chen, V.P., Pang, Y.P., Geng, L., and Gao, Y. (2016). Physiological roles for butyrylcholinesterase: A BChE-ghrelin axis. Chem. Biol. Interact. *259*, 271–275. https://doi.org/10.1016/j.cbi.2016.02.013.

47. Schopfer, L.M., Lockridge, O., and Brimijoin, S. (2015). Pure human butyrylcholinesterase hydrolyzes octanoyl ghrelin to desacyl ghrelin. Gen. Comp. Endocrinol. *224*, 61–68. https://doi.org/10.1016/j.ygcen.2015.05.017.

48. Dorling, J.L., Clayton, D.J., Jones, J., Carter, W.G., Thackray, A.E., King, J.A., Pucci, A., Batterham, R.L., and Stensel, D.J. (2019). A randomized crossover trial assessing the effects of acute exercise on appetite, circulating ghrelin concentrations, and butyrylcholinesterase activity in normal-weight males with variants of the obesity-linked FTO rs9939609 polymorphism. Am. J. Clin. Nutr. *110*, 1055–1066. https://doi.org/10.1093/ajcn/nqz188.

49. Chen, V.P., Gao, Y., Geng, L., Stout, M.B., Jensen, M.D., and Brimijoin, S. (2016). Butyrylcholinesterase Deficiency Promotes Adipose Tissue Growth and Hepatic Lipid Accumulation in Male Mice on High-Fat Diet. Endocrinology *157*, 3086–3095. https://doi.org/10.1210/en.2016-1166.

50. Chen, V.P., Gao, Y., Geng, L., and Brimijoin, S. (2017). Butyrylcholinesterase gene transfer in obese mice prevents postdieting body weight rebound by suppressing ghrelin signaling. Proc. Natl. Acad. Sci. USA *114*, 10960–10965. https://doi.org/10.1073/pnas.1706517114.

51. Vaughn, A.H., and Nielsen, R. (2024). Fast and Accurate Estimation of Selection Coefficients and Allele Histories from Ancient and Modern DNA. Mol. Biol. Evol. *41*, msae156. https://doi.org/10.1093/molbev/msae156.

52. Der Sarkissian, C., Ermini, L., Schubert, M., Yang, M.A., Librado, P., Fumagalli, M., Jónsson, H., Bar-Gal, G.K., Albrechtsen, A., Vieira, F.G., et al. (2015). Evolutionary Genomics and Conservation of the Endangered Przewalski's Horse. Curr. Biol. *25*, 2577–2583. https://doi.org/10.1016/j.cub.2015.08.032.

53. He, Z., Dai, X., Lyu, W., Beaumont, M., and Yu, F. (2023). Estimating Temporally Variable Selection Intensity from Ancient DNA Data. Mol. Biol. Evol. *40*, msad008. https://doi.org/10.1093/molbev/msad008.

54. Weatherford, J. (2004). Genghis Khan and the Making of the Modern World (Crown).

55. Meng, S., Zhang, Y., Lv, S., Zhang, Z., Liu, X., and Jiang, L. (2023). Comparison of muscle metabolomics between two Chinese horse breeds. Front. Vet. Sci. *10*, 1162953. https://doi.org/10.3389/fvets.2023.1162953.

56. Wang, T., Huang, J., Ren, W., Meng, J., Yao, X., Chu, H., Yao, R., Zhai, M., and Zeng, Y. (2025). Cardiac-Metabolic Coupling Revealed by Lipid and Energy Metabolomics Determines 80 km Endurance Performance in Yili Horses. Biology *14*, 1581. https://doi.org/10.3390/biology14111581.

57. O'Hara, V., Cowan, A., Riddell, D., Massey, C., Martin, J., and Piercy, R.J. (2021). A highly prevalent SINE mutation in the myostatin (MSTN) gene promoter is associated with low circulating myostatin concentration in Thoroughbred racehorses. Sci. Rep. *11*, 7916. https://doi.org/10.1038/s41598-021-86783-1.

58. Lockridge, O. (2015). Review of human butyrylcholinesterase structure, function, genetic variants, history of use in the clinic, and potential therapeutic uses. Pharmacol. Ther. *148*, 34–46. https://doi.org/10.1016/j.pharmthera.2014.11.011.

59. McGarry, K.G., Schill, K.E., Winters, T.P., Lemmon, E.E., Sabourin, C.L., Harvilchuck, J.A., and Moyer, R.A. (2020). Characterization of Cholinesterases From Multiple Large Animal Species for Medical Countermeasure Development Against Chemical Warfare Nerve Agents. Toxicol. Sci. *174*, 124–132. https://doi.org/10.1093/toxsci/kfz250.

60. Li, H., Handsaker, B., Wysoker, A., Fennell, T., Ruan, J., Homer, N., Marth, G., Abecasis, G., and Durbin, R. (2009). The Sequence Alignment/Map format and SAMtools. Bioinformatics *25*, 2078–2079. https://doi.org/10.1093/bioinformatics/btp352.

61. McCormick, R.F., Truong, S.K., and Mullet, J.E. (2015). RIG: Recalibration and Interrelation of Genomic Sequence Data with the GATK. G3 *5*, 655–665. https://doi.org/10.1534/g3.115.017012.

62. Danecek, P., Auton, A., Abecasis, G., Albers, C.A., Banks, E., DePristo, M.A., Handsaker, R.E., Lunter, G., Marth, G.T., Sherry, S.T., et al. (2011). The variant call format and VCFtools. Bioinformatics *27*, 2156–2158. https://doi.org/10.1093/bioinformatics/btr330.

63. Purcell, S., Neale, B., Todd-Brown, K., Thomas, L., Ferreira, M.A., Bender, D., Maller, J., Sklar, P., de Bakker, P.I., Daly, M.J., et al. (2007). PLINK: a tool set for whole-genome association and population-based linkage analyses. Am. J. Hum. Genet. *81*, 559–575. https://doi.org/10.1086/519795.

64. Yang, J., Lee, S.H., Goddard, M.E., and Visscher, P.M. (2011). GCTA: a tool for genome-wide complex trait analysis. Am. J. Hum. Genet. *88*, 76–82. https://doi.org/10.1016/j.ajhg.2010.11.011.

65. Alexander, D.H., Novembre, J., and Lange, K. (2009). Fast model-based estimation of ancestry in unrelated individuals. Genome Res. *19*, 1655–1664. https://doi.org/10.1101/gr.094052.109.

66. Kang, H.M., Sul, J.H., Service, S.K., Zaitlen, N.A., Kong, S.Y., Freimer, N.B., Sabatti, C., and Eskin, E. (2010). Variance component model to account for sample structure in genome-wide association studies. Nat. Genet. *42*, 348–354. https://doi.org/10.1038/ng.548.

67. Dong, S.S., He, W.M., Ji, J.J., Zhang, C., Guo, Y., and Yang, T.L. (2021). LDBlockShow: a fast and convenient tool for visualizing linkage disequilibrium and haplotype blocks based on variant call format files. Brief. Bioinform. *22*, bbaa227. https://doi.org/10.1093/bib/bbaa227.

68. Friedman, J., Hastie, T., and Tibshirani, R. (2010). Regularization Paths for Generalized Linear Models via Coordinate Descent. J. Stat. Softw. *33*, 1–22. https://doi.org/10.18637/jss.v033.i01.

69. Tibshirani, R. (1996). Regression Shrinkage and Selection Via the Lasso. J. R. Stat. Soc. Series B Stat. Methodol. *58*, 267–288. https://doi.org/10.1111/j.2517-6161.1996.tb02080.x.

70. Wang, Q., Li, Q., Liu, R., Zheng, M., Wen, J., and Zhao, G. (2016). Host cell interactome of PA protein of H5N1 influenza A virus in chicken cells. J. Proteomics *136*, 48–54. https://doi.org/10.1016/j.jprot.2016.01.018.

71. Dipta, B., Sood, S., Mangal, V., Bhardwaj, V., Thakur, A.K., Kumar, V., and Singh, B. (2024). KASP: a high-throughput genotyping system and its applications in major crop plants for biotic and abiotic stress tolerance. Mol. Biol. Rep. *51*, 508. https://doi.org/10.1007/s11033-024-09455-z.

STAR★METHODS

KEY RESOURCES TABLE

REAGENT or RESOURCE	SOURCE	IDENTIFIER
**Biological samples**		
Debao pony (CSTR:12156.05.1311C0001000001081)	this study	PRJNA1074421
Ningqiang pony (CSTR:12156.05.1311C0001000001050)	this study	PRJNA1074421
Hequ horse (CSTR:12156.05.1311C0001000001033)	this study	PRJNA1074421
Yushu horse (CSTR:12156.05.1311C0001000001051)	this study	PRJNA1074421
Guanzhong horse (CSTR:12156.05.1311C0001000001059)	this study	PRJNA1074421
Kazakh horse (CSTR:12156.05.1311C0001000001037)	this study	PRJNA1074421
Yili horse (CSTR:12156.05.1311C0001000001053)	this study	PRJNA1074421
Yanqi horse (CSTR:12156.05.1311C0001000001036)	this study	PRJNA1074421
**Chemicals, peptides, and recombinant proteins**		
DME/F-12 medium	HyClone	Cat# SH30023.01
Fetal bovine serum (FBS)	Gibco	Cat# A5669701
Triton X-100 solution	Solarbio	Cat# T8200
TRIzol	Invitrogen	Cat# 15596018
Sodium oleate	Fila	Cat# T36390
Sodium palmitate	Fila	Cat# S161420
**Commercial assays**		
Wizard® Genomic DNA Purification Kit	Promega	Cat# A1125
Alanine Aminotransferase (ALT) Assay Kit	Maccura	Cat# CH0101201
Aspartate Aminotransferase (AST) Assay Kit	Maccura	Cat# CH0101202
Alkaline Phosphatase (ALP) Assay Kit	Maccura	Cat# CH0101203
Total Protein (TP) Assay Kit	Maccura	Cat# CH0101001
Albumin (ALB) Assay Kit	Maccura	Cat# CH0101002
Cholinesterase (ChE) Assay Kit	Maccura	Cat# CH0101217
High-Density Lipoprotein Cholesterol (HDL-C) Assay Kit	Maccura	Cat# CH0101161
Low-Density Lipoprotein Cholesterol (LDL-C) Assay Kit	Maccura	Cat# CH0101162
Triglycerides (TG) Assay Kit	Maccura	Cat# CH0101151
Total Cholesterol (Chol) Assay Kit	Maccura	Cat# CH0101152
Glucose (Glu) Assay Kit	Maccura	Cat# CH0101102
Glycosylatedserumprotein (GSP) Assay Kit	SJodax	Cat# RB40411
Creatine Kinase (CK) Assay Kit	Maccura	Cat# CH0101210
Lactate Dehydrogenase (LDH) Assay Kit	Maccura	Cat# CH0101208
α-Hydroxybutyrate Dehydrogenase (HBDH) Assay Kit	Maccura	Cat# CH0101209
Angiotensin-converting enzyme (ACE) Assay Kit	SJodax	Cat# RB50652

(*Continued on next page*)

Continued

REAGENT or RESOURCE	SOURCE	IDENTIFIER
Myoglobin (MYO) Assay Kit	SJodax	Cat# RB50711
Pyruvicacid (PYR) Assay Kit	SJodax	Cat# RC40901
Calcium (Ca^{2+} Assay Kit	Maccura	Cat# CH0101251
Horse ghrelin ELISA assay Kit	Enzyme-linked	N/A
Lipofectamine 3000 Reagent Kit	Invitrogen	Cat# L3000015
Triglycerides (TG) ELISA Assay Kit	Solarbio	Cat# BC0625
Taq Pro Universal SYBR qPCR Master Mix	Vazyme	Cat# Q712-03
HiScript III All-in-one RT SuperMix Perfect for qPCR	Vazyme	Cat# R333-01
Recombinant DNA		
pcDNA3.1-3*flag (no-rBCHE)	This study	N/A
pcDNA3.1-3*flag-BCHE-N (rBCHE-N)	This study	N/A
pcDNA3.1-3*flag-BCHE-K (rBCHE-K)	This study	N/A
rAAV9-3*flag (no-rBCHE)	This study	N/A
rAAV9-3*flag-BCHE-N (rBCHE-N)	This study	N/A
rAAV9-3*flag-BCHE-N (rBCHE-K)	This study	N/A
Antibodies		
Anti-DDDDK tag (Binds to FLAG® tag sequence) antibody [EPR20018-251]	Abcam	Cat# ab205606; RRID:AB_2916341
anti-β-actin antibody [AC-15]	Abcam	Cat# Ab6276; RRID:AB_2223210
Alexa Fluor 488 Goat anti-Mouse IgG (H + L)	Invitrogen	Cat# A28175; RRID: AB_2536161
Cy3-conjugated antibodies	Proteintech	Cat# SA00009-1; RRID: AB_2814746
Deposited data		
Raw and analyzed data	This study	NCBI BioProject: PRJNA1074421
Experimental models: Cell lines		
Human: human hepatocellular carcinoma: HepG2	Peking Union Medical College Hospital	RRID: 1101HUM-PUMC000035
Human: human embryonic kidney 293T cell: HEK293T	Peking Union Medical College Hospital	RRID: 3101HUMSCSP5293
Experimental models: Organisms/strains		
C57BL/6J mice	Sibeifu	N/A
Software and algorithms		
Burrows-Wheeler Algorithm (BWA)	[44]	http://bio-bwa.sourceforge.net/
Genome Analysis Toolkit (GATK)	[45]	https://gatk.broadinstitute.org/hc/en-us
VCFtools	[46]	http://vcftools.sourceforge.net
PLINK	[47]	https://www.cog-genomics.org/plink2
GCTA	[48]	http://cnsgenomics.com/software/gcta/mlmassoc.html
ADMIXTURE	[49]	http://dalexander.github.io/admixture/index.html
Efficient Mixed Model Association eXpedited method (EMMAX)	[51]	https://genome.sph.umich.edu/wiki/EMMAX
CLUES2	[36]	https://github.com/avaughn271/CLUES2
PMMH	[38]	https://github.com/zhangyi-he/WFM-1L-DiffusApprox-PMMH

EXPERIMENTAL MODEL AND STUDY PARTICIPANT DETAILS

Horse samples

We collected peripheral blood samples from 298 horses representing eight populations, including Debao pony (DeBa, $N = 30$), Ningqiang pony (NiQi, $N = 49$), Hequ horse (HeQu, $N = 75$), Yushu horse (YuSh, $N = 9$), Guanzhong horse (GuZh, $N = 64$), Kazakh horse

(HSK, N = 29), Yili horse (YiLi, N = 32), and Yanqi horse (YaQi, N = 10) (Table S1). Samples were collected from eight geographically distinct farms or pastures. Adult horses were preferentially sampled, although exact age records were not consistently available. Sex was considered during sampling, but ratios were not strictly balanced across populations (Tables S2; S3). Under veterinary guidance, only clinically healthy individuals without obvious signs of disease were included, although fitness or training status was not directly quantified. The populations were grouped into four regional categories: southern China (SC; DeBa and NiQi), Qinghai–Tibetan (QT; HeQu and YuSh), central China (CC; GuZh), and northern China (NC; YiLi, YaQi, and HSK). These populations differ in breeding background, body type, adaptive characteristics, management system, and environmental conditions, including altitude and climate (Table S1). Such factors may contribute to inter-population variation, particularly in metabolic analyses, and were therefore considered in the interpretation of the results.

Blood samples were collected from horses under the supervision of a licensed equine veterinarian. The jugular vein was punctured using a sterile blood collection needle, and approximately 10 mL of blood was drawn into EDTA and heparinized tubes. Samples were immediately placed on ice and transported to the laboratory for processing. Serum was separated by centrifugation at 1200× g for 5 min and stored at −80°C until further analysis. All procedures involving horse sampling were conducted in strict accordance with institutional and national ethical guidelines. Ethical approval for this study was granted by the Animal Welfare and Ethics Committee of the Institute of Animal Science, Chinese Academy of Agricultural Sciences, Beijing, China, under protocol number IAS2023-92.

Mice

C57BL/6J mice (8 weeks old) were purchased from Sibeifu (Beijing) Biotechnology Co., Ltd., and housed at the Laboratory Animal Center of the Institute of Animal Sciences, Chinese Academy of Agricultural Sciences, Beijing, China. All mice were male and were housed under a 12-h light/dark cycle with free access to food and water. All mouse experiments were conducted in strict accordance with institutional and national ethical guidelines. Ethical approval for the mouse experiments was granted by the Ethics Committee for Animal Experimentation of the Institute of Animal Science, Chinese Academy of Agricultural Sciences, Beijing, China, under protocol number IAS2024-171.

Cell lines

Human embryonic kidney 293T (HEK293T) cells and human hepatocellular carcinoma (HepG2) cells were purchased from the Peking Union Medical College Hospital. Cell-line authentication and mycoplasma testing were performed by the provider. The cell lines were not independently authenticated or tested for mycoplasma contamination in this study. Both cell lines were cultured in Dulbecco's Modified Eagle Medium Nutrient Mixture F-12 (DME/F-12; HyClone, USA) supplemented with 10% certified fetal bovine serum (Gibco, USA) and penicillin (100 U/mL)/streptomycin (100 μg/mL) (Gibco, USA) at 37°C and 5% CO_2.

METHODS

Whole-genome sequencing

Genomic DNA was extracted from the blood using the standard Promega extraction protocol (Promega, A1125, America). DNA quality and integrity were assessed via A260/A280 ratio and agarose gel electrophoresis. Library preparation and whole-genome sequencing were conducted by BGI Genomics (Shenzhen, China). Briefly, qualified genomic DNA was fragmented, end-repaired, A-tailed, and ligated with indexed adapters to construct paired-end sequencing libraries. These libraries were then sequenced on the DNBSEQ-T7 platform to generate 150 bp paired-end reads. The raw sequencing data processing, including base calling and quality control, was performed using BGI's in-house bioinformatics pipelines. Clean sequence reads were aligned to the EquCab3 horse reference genome[42] using Burrows-Wheeler Algorithm (BWA-mem algorithm),[60] with default parameters. PCR duplicates were removed using the MarkDuplicates function in Picard (picard-tools-1.56, http://picard.sourceforge.net), and local realignment around indels was performed by Genome Analysis Toolkit (GATK).[61] Sequencing achieved an average depth-of-coverage of 10.62-fold per individual, yielding a total of 19,878,270 high-confidence SNPs (Table S2). SNP calling was first performed in each individual using GATK HaplotypeCaller (version 4.0), followed by joint genotyping with GenotypeGVCFs applying default settings. SNPs were filtered by retaining only those with a minor allele frequency (MAF) > 0.01, genotype call rate >90%, missing genotype rate <10%, biallelic status, and Hardy-Weinberg equilibrium (HWE) p-value > 1e−5 using VCFTools.[62]

Population structure analysis

Linkage disequilibrium (LD) pruning was performed in PLINK v1.9,[63] using sliding windows of 50 SNPs, a step size of 10, and a pairwise r^2 threshold of 0.2 (–indep-pairwise 1000 10 0.2), resulting in 3,513,517 independent SNPs for downstream analyses. Principal component analysis (PCA) was carried out using GCTA v1.91,[64] and visualization of the first three principal components, which combined, explained 24.6% of the total variance, was conducted in R. Individual ancestry was inferred using ADMIXTURE v1.3.0[65] with 10,000 bootstrap pseudo-replicates, exploring K values ranging from 2 to 5. A Neighbor-Joining (NJ) tree was constructed in MEGA-X v10.1.8 based on the pairwise genetic distance matrix computed by PLINKv1.9[63] (1-ibs).

Runs of homozygosity and LD-decay

Runs of homozygosity (ROH) were identified for each breed or population using the –homozyg function implemented in PLINK v1.9,[63] based on the unpruned matrix of high-quality SNPs (N = 19,878,270). The main analysis was conducted using the following parameters[30]: –homozyg-window-snp 50, –homozyg-window-het 1, –homozyg-snp 10, –homozyg-kb 100, –homozyg-density 10, and –homozyg-gap 100. These settings were selected for the high-density whole-genome SNP dataset to retain sensitivity for relatively short ROH while limiting artificial extension caused by sparse marker density or excessive merging of adjacent homozygous tracts. In general, lower minimum SNP-number or ROH-length thresholds increase the detection of short ROH, whereas larger inter-SNP gap allowances or more permissive heterozygosity settings may inflate ROH length estimates by merging neighboring homozygous segments. To assess parameter sensitivity, we further compared the main parameter set with two previously published ROH parameter settings. Although the absolute estimates of total ROH length, mean ROH length, and f_{ROH} varied among parameter sets, the major population-level patterns remained broadly consistent.

Serum biochemistry parameters

A total of 21 baseline serum biochemistry parameters were quantified, including alanine amino transferase (ALT), aspartate amino transferase (AST), AST/ALT, total protein (TP), globulin ratio (A/G), albumin (ALB), butyrylcholinesterase (BChE), alkaline phosphatase (ALP), glucose (GLU), pyruvicacid (PYR), glycosylated serum protein (GSP), creatine kinase (CK), lactate dehydrogenase (LDH), α-hydroxybutyrate dehydrogenase (HBDH), myoglobin (MYO), angiotensin-converting enzyme (ACE), calcium (Ca), triglyceride (TG), cholesterol (CHOL), high-density lipoprotein cholesterol (HDL-c), low-density lipoprotein cholesterol (LDL-c) (Table S3). All measurements were performed using an automatic analyser (Hitachi 7080, Japan) in accordance with the manufacturer's protocols. Assay kits were sourced from Maccura Biotech Co., Ltd.

Principal component analysis (PCA) and correlation analysis of serum biochemical parameters

To account for age-related effects on serum biochemical parameters, we applied a linear regression model defined as: $y = \beta_0 + \beta_1 x + \epsilon$, where β_0 is the intercept, β_1 represents the change in the indicator per unit increase in age (x), and ϵ is the residual error term. Principal component analysis (PCA) was carried out in R to identify major trends and sources of variation within the biochemical dataset. Contribution plots and PCA score distributions were generated to visualize the principal parameters contributing to principal components. Correlation analysis was performed using the cor() function in R on a complete dataset (i.e., no missing values), with Pearson's correlation coefficient (r^2) quantifying pairwise associations between traits. The ggplot2 package was used for data visualization.

Genome-wide association study

We carried out genome-wide association studies (GWASs) for 21 serum biochemistry traits across 298 Chinese horse samples using the Efficient Mixed Model Association eXpedited method (EMMAX).[66] To control for sex and population structure, sex and the first three principal components derived from whole genome SNP data were included as fixed covariates. To account for cryptic relatedness and polygenic background, a kinship matrix was incorporated as a random effect. This matrix was specifically generated as an Identity-by-State (IBS) matrix using the emmax-kin software. The calculation employed the recommended HEreg method (-h flag) to standardize the IBS values based on expectations under Hardy-Weinberg Equilibrium, followed by scaling to achieve a mean of zero (-s flag). This optimized approach yields a robust IBS-based genetic relationship matrix that most effectively captures the realized genetic similarities among all pairs of individuals for inclusion in the mixed model. Genome-wide significance thresholds were determined using Bonferroni, setting a cutoff at 2.82e−9 (0.05/total SNPs). Linkage disequilibrium (LD) analysis was conducted using PLINK v1.9,[63] and LD blocks within 6 kb were visualized using LDBlockShow v1.35.[67]

Linear modeling of BCHE variant effects

To further evaluate the effect of the *BCHE* variant on BChE activity, we performed additional linear-model analyses in R. Individuals with missing values for BChE activity, genotype, or sex were excluded. The three *BCHE* genotypes at ECA19:9,198,989 (G/C) were coded additively as 0, 1, and 2, according to the number of "C" alleles. Models included sex, breed, and the first three principal components as covariates. The main-effect model was specified as BChE $\sim$ Genotype + Sex + Breed + PC1 + PC2 + PC3, and the interaction model as BChE $\sim$ Genotype $\times$ Sex + Breed + PC1 + PC2 + PC3. The variant $\times$ sex interaction was assessed by comparing models with and without the interaction term. The proportion of phenotypic variance explained by the *BCHE* locus was estimated as the increase in model R^2 after adding the SNP term to a baseline model including sex, breed, and the first three principal components.

Serum ghrelin

To assess basal ghrelin levels, serum was collected from 134 individuals selected from the original cohort of 298 horses across five horse breeds: Hequ horse (HeQu, N = 22), Yushu horse (YuSh, N = 21), Guanzhong horse (GuZh, N = 31), Kazakh horse (HSK, N = 23), and Yanqi horse (YaQi, N = 37). This subset was not designed to enforce exact proportional sampling across breeds or strict matching of sex composition, but to retain the main ecological and management backgrounds represented in the original cohort, including predominantly grazing populations, one stall-fed population, and populations from different altitude regions. Ghrelin concentration was quantified using a spectrophotometric ELISA assay (Enzyme-linked Biotechnology, China), following the standardized

manufacturer's protocol to ensure consistency and reliability. Absorbance was measured at 450 nm with a Tecan Infinite 200 Pro MultiMode Microplate Reader.

Serum lipid profiling

We profiled the lipid composition of horse serum samples using liquid chromatography-tandem mass spectrometry (LC/MS-MS), enabling comprehensive characterization of lipid metabolites. A total of 214 horses representing 10 breeds were selected and stratified by genotypes, with 64 CC homozygotes, 102 heterozygotes, and 48 GG homozygotes at ECA19:9,198,989 (G/C). The breed distribution included Debao (DeBa, $N = 22$), Ningqiang (NiQi, $N = 32$), Hequ horse (HeQu, $N = 23$), Yushu horse (YuSh, $N = 24$), Guanzhong horse (GuZh, $N = 25$), Kazakh horse (HSK, $N = 28$), and Yanqi horse (YaQi, $N = 30$). Lipidomics analysis was conducted by Metware Biotechnology Co., Ltd, using the procedures descripted as Supplementary information.

Coefficients of variation and heritability

Coefficients of variation (CV) for serum biochemistry parameters and metabolites were calculated as follows: CV (%) = (sd/mean)*100%, where "sd" and "mean" represent the standard deviation and mean of individual serum biochemistry parameters and metabolites, respectively.

The narrow-sense heritability (h^2) of each serum biochemical trait was estimated using a genomic-relatedness-based restricted maximum likelihood (GREML) approach in GCTA v1.90.[64] The model incorporated a genetic relationship matrix (GRM) derived from genome-wide SNPs to estimate the additive genetic variance (σ^2_a). Sex and the top 100 genetic principal components were included as fixed covariates. The heritability was calculated as $h^2 = \sigma^2\_a/(\sigma^2\_a + \sigma^2\_e)$, where σ^2_e is the residual variance.

Least absolute shrinkage and selection operator (LASSO) regression analysis

A total of 93 differential lipids identified from pairwise comparisons were used as predictor variables, with BChE activity treated as the response variable. Prior to analysis, lipid abundances were standardized to ensure comparability across variables. LASSO regression analysis was performed using the *glmnet* R package,[68] which implements L1-regularization to select a subset of predictive variables.[69] 10-fold cross-validation was applied to determine the optimal regularization parameter (λ), and lipids with non-zero coefficients at the optimal λ were retained for subsequent analysis.

To further assess associations between individual lipids and BChE activity, Spearman correlation analysis was conducted. Lipids supported by both LASSO selection and Spearman correlation analysis were further visualized using correlation scatterplots.

Plasmid construction

All expression constructs were synthesized by Tsingke Biotechnology Co., Ltd., except for the 3×Flag-tagged pcDNA3.1 vector provided by Dr. Li.[70] Site-specific mutations were introduced by PCR-based mutagenesis using high-fidelity DNA polymerase, followed by digestion with NheI and BamHI and subcloning into the pcDNA3.1 vector using the EasyGeno Rapid Recombinant Clone Kit (TIANGEN). To enhance expression efficiency in mammalian cells, the equine BCHE coding sequence was codon-optimized (CAI increased from 0.64 to 0.94) using GeneOptimizer, while maintaining the original amino acid sequence. Detailed procedures are provided in the Supplementary Information.

Cell transfection

Recombinant full-length equine BChE proteins, each tagged with a C-terminal 3×FLAG epitope, were transiently expressed in HEK293T and HepG2 cells. Cells were seeded and cultured to ~70% confluence before transfection with 5 mg of plasmid DNA using a Lipofectamine 3000 Reagent kit (Invitrogen, USA), following the manufacturer's instructions.

Western blot analysis

To confirm BChE overexpression 48-h post-transfection, HEK293T and HepG2 cell lysates were prepared using lysis buffer (Solarbio, China) with phenyl methyl sulfonyl fluoride (PMSF; Solarbio, China). Proteins were separated by SDS-PAGE on 10% sodium dodecyl sulfate-polyacrylamide gels, and transferred to polyvinylidene fluoride (PVDF) membranes (Millipore, USA). Membranes were blocked with Fast Blocking Western solution (Beyotime Biotechnology, China) for 10min, followed by overnight incubation at 4°C with rabbit anti-FLAG (1:5000; Abcam, UK) and mouse anti-β-actin (1:2000; Abcam, UK) primary antibodies. After three washes with TBST, membranes were incubated with appropriate HRP-conjugated secondary antibodies for 1h at room temperature and washed again three times. Detection was performed using enhanced chemiluminescent substrate, and images were captured using a Tanon 5200 Multi Imaging system (Tanon, China).

Adipogenic induction and Oil Red O staining

To evaluate the role of *BCHE* on lipid metabolism, adipogenesis was induced in HepG2 cells using a mixture of sodium oleate and sodium palmitate (v: v = 2:1) (Fila Biotechnology, China), following a standard protocol based on an adipogenic cocktail. Forty-eight hours post-transfection, sodium oleate and sodium palmitate were added to the HepG2 culture medium. After 24h of induction, cells

were washed three times with PBS, fixed with 4% paraformaldehyde for 30 min at room temperature, and stained with Oil red O staining solution (Beyotime Biotechnology, China) for 30 min. Lipid droplet accumulation was visualized as orange-red inclusions under a Zeiss LSM880 confocal microscope (Zeiss, Germany).

Intracellular triglyceride (TG) content

Following adipogenic induction, HepG2 cells were harvested, and intracellular lipids were extracted using a 1:1 (v/v) mixture of n-heptane and isopropanol, followed by ultrasonic disruption with a Bioruptor UCD-200 (Diagenode, Belgium). Intracellular triglyceride (TG) levels were quantified using an ELISA assay kit (Solarbio, China), following the manufacturer's instructions. Absorbance was measured at 420nm using a Tecan infinite 200 pro MultiMode Microplate Reader (Tecan, Switzerland). TG concentrations were calculated using the following formula:

TG content (mg/10^4 cell) = $C_{standard} \times (A_{test} - A_{blank})/(A_{standard} - A_{blank})$/Cell (mg/$10^4$ cell), where $C_{standard}$ = 1mg/mL.

Immunofluorescence (IF) staining

Given the apparent hydrolysis effect of BChE on lipid droplets, we sought to determine the subcellular localization of the enzyme in HepG2 cells, following adipogenic induction. Immunofluorescence staining was carried out to visualize BChE distribution. Treated cells were washed three times with PBS and fixed with 4% paraformaldehyde for ~15-30min at room temperature. After fixation, cells were permeabilized with 0.25% Triton X-100 (Solarbio, China), and washed again with PBS. Blocking was carried out using 10% goat serum for 1h at room temperature. Cells were then incubated overnight at 4°C with a rabbit anti-FLAG primary antibody (1:5000; Abcam, UK) diluted in blocking buffer, followed by three PBS washes. Secondary antibody incubation was conducted for 1h at room temperature using Alexa Fluor 488 (Invitrogen, USA) and Cy3-conjugated antibodies (Proteintech, USA), diluted in PBS. Nuclei were stained with DAPI (50–100 μg/mL) (Solarbio, China) for 15 min at room temperature. Fluorescence images were captured using a Zeiss LSM880 confocal scanning microscope (Zeiss, Germany).

Construction of *BCHE* overexpression mouse model

To investigate BChE effects *in vivo*, we used overexpression vectors carrying the C and "G" alleles, or flag controls, to generate a type 9 recombinant adeno-associated virus (rAAV), under the control of a CMV promoter. The constructs were synthesized and packaged by HanHeng Biotechnology (Shanghai) Co., Ltd., via helper virus-free transient transfection in HEK-293 cells, with viral titers of no less than 1×10^{12} vector genomes (V.g.)/mL.

A total of 24 male C57BL/6J mice (8-weeks old) were purchased from Sibeifu (Beijing) Biotechnology Co., Ltd., and randomly subdivided into three groups. The first group (no-rBCHE) was injected with Flag-rAAV, while the second (rBCHE-N) and third (rBCHE-K) groups were injected with C-rAAV and G-rAAV vectors. Each mouse received a 100μL tail vein injection. Body weight and food intake were monitored weekly until sacrifice at week 28.

RT-qPCR experiments

Liver and adipose tissues were collected from rAAV-injected C57BL/6J mice at 28 weeks. Mice were euthanized under anesthesia, and tissues were excised, weighed, and either snap-frozen for RNA extraction or fixed for histological analysis. Total RNA was isolated using TRIzol (Invitrogen) and reverse-transcribed with the HiScript III All-in-one RT SuperMix Kit (Vazyme). Quantitative real-time PCR was performed using SYBR Green chemistry on an ABI Q7 Flex Real-Time PCR System (Applied Biosystems). BCHE mRNA levels were normalized to ACTB and calculated using the $2^{\wedge}-\Delta\Delta CT$ method. Detailed information on primer sequences and thermocycling parameters is provided in the Supplementary Information. Detailed procedures are provided in the Supplementary Information.

Histological staining

Mice were euthanized by cervical dislocation under anesthesia. The abdominal cavity was immediately opened through a midline incision, and liver and fat pads surrounding the gonads or the abdominal wall were carefully dissected, weighed, and either snap-frozen in liquid nitrogen or fixed in 4% paraformaldehyde (Solarbio, China). Fixed samples were subjected to Hematoxylin and Eosin (H&E) staining, while frozen samples were subjected to Oil Red O staining. All embedding, sectioning, and staining processes were carried out by Servicebio Biotechnology Co., Ltd.

Validation in the modern horse populations

We genotyped 1,238 modern horses (GP2) from 64 breeds or populations to assess the frequency of the *BCHE* SNP variant (ECA19:9,198,989, G/C) in present-day horse populations worldwide. The validation dataset included 978 Asian and 260 non-Asian individuals, and detailed information on the GP2 dataset is provided in Table S11. Genotyping was performed using two approaches: Kompetitive Allele-Specific PCR (KASP)[71] for 525 horses and SNP calling from whole-genome sequencing data for 713 horses. KASP genotyping was carried out by China Golden Marker (Beijing) Biotech Co., Ltd. The sequencing data included those data newly generated in this study and publicly available data from previous studies (NCBI BioProject accession nos. PRJEB14779, PRJEB9267, PRJEB9269, PRJEB9799, PRJNA230019, PRJNA641243, PRJNA841639, PRJNA331853, PRJNA357976, PRJNA168142, PRJNA391840, PRJNA515160, and PRJNA780752).

Selection analyses

We estimated modern allele frequencies using two independent datasets. The first (GP1) included 990 modern DOM2 horses reported in,[28] and the second (GP2) included 1,238 modern DOM2 horses sequenced by KASP[71] and whole-genome sequencing. Horses were classified as Asia (AS) or non-Asia (non-AS) showed different genetic ancestry profiles (Figure S19). Allele frequencies and 95% HPD intervals were then estimated in R. Based on GP1, the "C" allele frequency was 0.51 (95% HPD = 0.48–0.54, $N = 571$) in AS horses and 0.48 (95% HPD = 0.44–0.51, $N = 419$) in non-AS horses. Similarly, GP2 yielded a frequency of 0.54 (95% HPD = 0.52–0.57, $N = 987$) in AS horses and 0.48 (95% HPD = 0.44–0.53, $N = 269$) in non-AS horses.

Demographic modeling was performed as described by Liu et al.[28] Ancient DOM2 horses were further grouped into AS and non-AS categories based on their geographic distribution. Genotype probabilities were estimated using ANGSD v0.930, following the procedures outlined by Liu et al.[28] Genotype likelihoods were log-transformed, and the age of each individual, determined from radiocarbon dating, was converted to generations assuming a generation time of 7.4 years.[27]

Both one-epoch and two-epochs selection models were applied to AS and non-AS horses. For the two-epochs model, the time point at which the selection regime shifted (T) was inferred through a grid search, testing values of T between 10 and 550 generations in steps of five, with Tend fixed at 600 generations. Three dominance parameters were considered: $h = 0$ (recessive), $h = 0.5$ (codominant), and $h = 1$ (dominant). The maximum selection coefficient (-sMax) was explored within the range of -0.75 to 0.75. The model yielding the highest log likelihood and corresponding log likelihood was taken as the most likely selection scenario for the dataset, including estimates of both T and h.

To ensure the robustness of our findings against potential demographic mis-specifications, we cross-validated the selection signals using multiple independent demographic scenarios: the subcontinental models from Liu et al.[28] for DOM2 populations within and outside Asia, as well as the global model from Der Sarkissian et al.[52] for the global DOM2 lineage. For each demographic scenario, we re-ran the CLUES2 grid search across the same range of selection shift times (T) and dominance parameters (h) (Table S13).

Selection coefficients were further estimated using the PMMH software.[53] Input files were derived from genotype likelihoods generated by ANGSD, following the same procedure as in CLUES2, with T and h fixed to the best estimates obtained from CLUES2. To improve convergence, we increased the number of iterations from 20,000 to 100,000. Both PMMH v1.5 and v1.4 were run to investigate the potential impact of the inheritance mode assumed for the "C" allele.

QUANTIFICATION AND STATISTICAL ANALYSIS

Statistical analyses and data visualization were performed using R v4.2.2, GraphPad Prism v8.0.2, and analysis-specific software described in the corresponding Method details subsections. Group comparisons were assessed using Kruskal-Wallis tests, and P-values <0.05 were considered statistically significant. Exact values of n are provided in the corresponding Results section, figure legends, tables, and Method details subsections. Depending on the analysis, n represents the number of horses, mice, serum samples, tissue samples, cell-culture replicates, lipid species, SNPs, or ancient genomes, as specified in the relevant section. Measures of center and dispersion or precision, including mean, SD, confidence intervals, and HPD intervals, are reported in the corresponding figure legends, figures, Results section, tables, and Method details subsections.

Cell Reports, Volume *45*

Supplemental information

Adaptive variation

in butyrylcholinesterase-influenced lipid

metabolism during horse domestication

Yanli Zhang, Xuexue Liu, Xintong Wang, Yanhui Yu, Yaozhen Jia, Yabin Pu, Yuehui Ma, Ludovic Orlando, and Lin Jiang

Supplementary Methods S1: Additional methodological details supporting the STAR Methods

Conservation analysis of BCHE protein

To investigate the structural features of the equine BCHE protein, we retrieved its protein sequence from the Uniprot database (Accession Nb. F7CC84). Domain annotations provided by Uniprot were supplemented using the Simple Modular Architecture Research Tool (SMART)[1], revealing four distinct regions, including the Acetylcholinesterase tetramerization domain (AChE_tetra), the Carboxylesterase family domain (COesterase), the Abhydrolase_3 domain, and a low-complexity region. To assess the evolutionary conservation of BCHE across taxa, we downloaded BCHE protein sequences from 60 vertebrate via NCBI, comprising 34 mammals, 15 birds, four lepidosaurs, four turtles, two amphibians, and one lungfish (accession numbers are listed in Table S6. Multiple sequence alignment was performed using the MUSCLE algorithm with default parameters, followed by manual curation. A Neighbor-Joining (NJ) tree was constructed using MEGA X[2]. The evolutionary distances were computed using the Poisson correction method. All positions containing gaps and missing data were eliminated (complete deletion option). The robustness of the inferred phylogeny was evaluated by bootstrap resampling with 1000 replicates.

Prediction of BCHE protein structure and stability

To evaluate structural changes in the BChE protein resulting from the amino acid substitution identified, we used the transform-restrained Rosetta (trRosetta) server for accurate *de novo* prediction of K ("G" allele) and N ("C" allele) protein structures[3-5]. The inclusion of homologous templates improved model accuracy, with TM-scores of 0.936 (K, "G" allele), and 0.948 (N, "C" allele), indicating structural reliability. The trRosetta server is freely accessible at https://yanglab.nankai.edu.cn/trRosetta/. The resulting structural models of the two variant BCHE proteins were further evaluated for stability using the DUET web server, which integrates three computational approaches, i.e., DUET[6], mCSM[7], and SDM[8]. All predictions consistently supported differential stability between the two variants (Figures S8 and S9). The DUET web server is freely accessible at http://structure.bioc.cam.ac.uk/duet.

Lipid profiling

We profiled the lipid composition of horse serum samples using liquid chromatography-tandem mass spectrometry (LC/MS-MS), enabling comprehensive characterization of lipid metabolites.

For lipid extraction, 50 μL of serum was mixed with 1mL of extraction solvent (MTBE: MeOH =3:1, v/v) containing internal standards. The mixture was vortexed for 15 min, followed by the addition of 200 μL of water, vortexing for 1 min, and centrifugation at 12,000 rpm for 10 min. The upper organic phase (200 μL) was collected, dried using a vacuum concentrator, and resuspended in 200 μL mobile phase B prior to LC-MS/MS analysis on the LC-ESI-MS/MS platform (UPLC, ExionLC AD; MS, QTRAP® System, https://sciex.com/). Chromatic separation was achieved on a Thermo Accucore™ C30 column (2.6 μm, 2.1 mm*100 mm i.d.). The solvent system consisted of mobile phase A: acetonitrile/water (60/40, V/V, 0.1% formic acid, 10 mmol/L ammonium formate), and; mobile phase B: acetonitrile/isopropanol (10/90 V/V, with the same additives. The gradient program was as follows: A/B（80:20, V/V）at 0 min, 70:30 V/V at 2.0 min, 40:60 V/V at 4 min, 15:85 V/V at 9 min, 10:90 V/V at 14 min, 5:95 V/V at 15.5 min, 5:95 V/V at 17.3 min, 80:20 V/V at 17.3 min, 80:20 V/V at 20 min. Flow rate was set at 0.35 ml/min, column temperature at 45°C, and injection volume at 2μl.

Mass spectrometry was performed in both positive and negative ion modes using a ESI-triple quadrupole-linear ion trap (QTRAP) equipped with an ESI Turbo Ion-Spray interface controlled by Analyst v1.6.3 software (Sciex). ESI parameters were included: source temperature 500°C, ion spray voltage (IS) 5500 (positive) / -4500 V (negative), ion source gas 1 (GS1=45 psi), ion source gas 2 (GS2=55 psi), curtain gas (CUR=35 psi), and collision gas (CAD) set to medium. Instrument tuning and mass calibration were performed using 10 and 100 μmol/L polypropylene glycol solutions in QQQ and LIT modes, respectively. Multiple reaction monitoring (MRM) was used for metabolite detection, with nitrogen as the collision gas (5 psi). Declustering potential (DP) and collision energy (CE) were optimized for each MRM transition, and metabolite-specific MRM transitions were assigned on the basis of retention times.

Metabolite identification was carried out using the targeted standard Metare Database (MWDB), which integrates compound retention time (RT), parent ion pair information, and MS/MS fragmentation spectra for qualitative analysis. After raw mass spectrometry data acquisition across all samples, peak areas were extracted for each metabolite, and signal intensities were normalized

across samples to correct for inter-run variability. Multivariate statistical analyses were conducted using the Metware Cloud (https://cloud.metware.cn/). Principal component analysis (PCA) and orthogonal partial least squares-discriminant analysis (OPLS-DA) were performed to capture global variation and discriminate between groups. Model performance was assessed using the R^2X, R^2Y, and Q^2 metrics to ensure robustness and predictive reliability. Differential metabolites were defined as those with a variable importance in projection (VIP) score >1.0, an absolute|fold change (FC)| ≥1.5, and a statistical significance threshold of $P<0.05$.

Plasmid construction

Vector construction followed previously described protocols[9]. Briefly, site-specific mutations were introduced by designing complementary oligonucleotide primers spanning the target locus. PCR amplification was then performed using a high-fidelity DNA polymerase (Vazyme, China) under the following thermocycling conditions: an initial denaturation at 98℃ for 30 s; followed by 18 cycles of 98℃ for 10 s, 60℃ for 30 s, and 72℃ for 6 min; with a final extension at 72℃ for 10 min. This process generated both the mutant (C) and wild-type (G) variant fragments, and the methylated parental DNA template was subsequently digested with *NheI* and *BamHI* restriction enzyme. The EasyGeno Rapid Recombinant Clone Kit (VI201, TIANGEN) was used to subclone the wild-type and the mutant sequences of the equine BCHE gene into the pcDNA3.1 vector for subsequent overexpression analysis. The native equine BCHE (horse-BCHE) sequence exhibited a suboptimal codon adaptation index (CAI) of 0.64 for expression in mammalian systems. To enhance its translational efficiency in human and murine host cells, we performed codon optimization using GeneOptimizer® software (Tsingke Biotechnology Co., Ltd.), while ensuring the encoded amino acid sequence remained unchanged (Figure S16). The optimization process incorporated preferred codon usage patterns from both Homo sapiens and Mus musculus. The optimized construct achieved a significantly improved CAI of 0.94, representing a 46.9% increase in codon adaptation relative to the wild-type sequence. This substantial enhancement in codon optimization is predicted to markedly improve BCHE protein expression levels in the target expression systems.

Detection of the recombinant BChEs activity

BChE enzymatic activity was measured using a spectrophotometric assay (Enzyme-linked

Biotechnology, China), as previously described[10; 11]. Culture media containing recombinant BChEs, collected post-transfection and adipogenic induction, were subjected to ultrasonic disruption and centrifuged at 1000 g for 10min after to remove cellular debris. For each reaction, 0.1mL of sample, standard, or ddH$_2$O (blank) was mixed with 0.5mL of substrate and 0.5mL of indicator, and incubated at 37°C for 6 min. Subsequently, 0.03mL inhibitor and 0.1mL clarifying agent were added to each mixture and incubated at room temperature for 15min. Absorbance was measured at 412 nm using a Tecan infinite 200 pro MultiMode Microplate Reader. Enzymatic activity (U/mL) was calculated using the formula:

Active enzyme units (U/mL) = $\frac{A_{test}-A_{control}}{A_{standard}-A_{blank}} \times C_{standard} \times N$

where $C_{standard}$ is the concentration of the standard, and N is the sample dilution factor.

RT-qPCR experiments

We sacrificed rAAV-injected C57BL/6J mice at 28 weeks and collected tissues including liver and adipose tissues. Mice were euthanized by cervical dislocation under anesthesia. The abdominal cavity was immediately opened through a midline incision, and abdominal adipose tissue was carefully dissected. Fat pads surrounding the gonads or the abdominal wall were excised, weighed, and either snap-frozen in liquid nitrogen for subsequent analysis or fixed in 4% paraformaldehyde (Solarbio, China) for histology. Total RNA was extracted using TRIzol (Invitrogen, USA) and the TIANGEN Reagent kit (TIANGEN, China). First-strand cDNA was synthesized by reverse-transcription using the HiScript III All-in-one RT SuperMix Perfect for qPCR Kit (Vazyme, China). Quantitative real-time PCR (qPCR) was performed on an ABI Q7 Flex Real-Time PCR System (Applied Biosystems, USA) using the Taq Pro Universal SYBR qPCR Master Mix kit (Vazyme, China). BCHE expression levels were measured through technical replicates and normalized to ACTB expression levels. qPCR conditions were as follows: Each 20 µL qPCR reaction contained 10 µL of 2X Taq Pro Universal SYBR qPCR Master Mix, 0.4 µL of each forward and reverse primer (10µM), 2 µL of cDNA template, and 7.2 µL of ddH2O. The thermal cycling conditions were as follows: initial denaturation at 95°C for 30s; followed by 40 cycles of 95°C for 10s (denaturation) and 60°C for 30s (annealing/extension). Forward and reverse PCR primers were 5'-TGCAGGTTTTGGACCCTGTTCTT and 5'-ACTACAAGGATGACGATGA, and 5'-TCTGGCACCACCTTCTACAATG and 5'-AGCACAGCCTGGATAGCAACG for BCHE

and ACTB, respectively). These PCR primers targeted PCR fragments of 188 bp and 260 bp, respectively. Gene expression fold-change was calculated via the $2^{(-\Delta\Delta CT)}$ method. For each sample, ΔCT was computed as $CT\_target - CT\_ACTB$. The $\Delta\Delta CT$ value was then determined using the formula $\Delta\Delta CT = \Delta CT\_sample - \Delta CT\_control$, where the control group average ΔCT served as the calibrator. Results are expressed as $2^{(-\Delta\Delta CT)}$.

Validation in the modern horse populations

We genotyped 1,277 modern horses from 69 breed or populations to assess the frequency of the BCHE SNP variant (ECA19:9,198,989, G/C) across the world (Table S11). This data set included: 21 Przewalski's horses; 1097 Chinese landrace horses from Southwest China (SC=243; DeBa=57, NiQi=57, YuNa=17, WeSh=27, LiCh=25, LuPi=25, and JiCh=35), Qinghai-Tibet (QT=352; YuSh=50, HeQu=83, JiZi=16, MZH=24, NiMu=37, LKZ=21, DaTo=57, and CDM=64), Central China (CC=104, GuZh=104), North China (NC=370; JiLi=24, HSK=38, HeHe=29, YiLi=44, MoGo=60, ELC=57, YaQi=69, WuSh=27 and SeSe=22); Mongolia horse (WMG=28); Pony mix (Pony mix=59); Quarter horse (Quarter=37); Thoroughbred (ThB=72); Middle East horse (MA=20); Europe horse (EU=78); and American horse (AM=12). Breed classification followed the China National Commission of Animal Genetic Resources 2011 and Horse and Ass Breeds in China (1986). SNP calling was carried out as described follows (section Whole-genome sequencing). Genotyping of the “C” allele was performed with Graphtyper v2.7.4 using default parameters, and variants were subsequently filtered with vcffilter(Garrison et al. 2022), using the following criteria: -f 'ABHet < 0.0 | ABHet > 0.33' -f 'ABHom < 0.0 | ABHom > 0.97' -f 'MaxAASR > 0.4' -f 'MQ > 30' -t 'pass' -F 'graphtyper_filter'. The frequencies of the BCHE “C” allele estimated within and outside Asia were compared with the frequencies present in a previously published global panel of horse genomes[12], applying the same genotyping filters as above. To assess the consistency of the genotype frequencies between this global panel (GP1) and this study data (GP2), we performed a bootstrap resampling analysis. We used the breed proportions from GP1 as sampling weights to generate 10,000 bootstrap samples from GP2. The empirical distribution of allele “C” frequencies from these resamples was then compared to the observed frequency in GP2, with significance determined using 95% confidence intervals and two-sided *P*-values.

Supplementary Figures

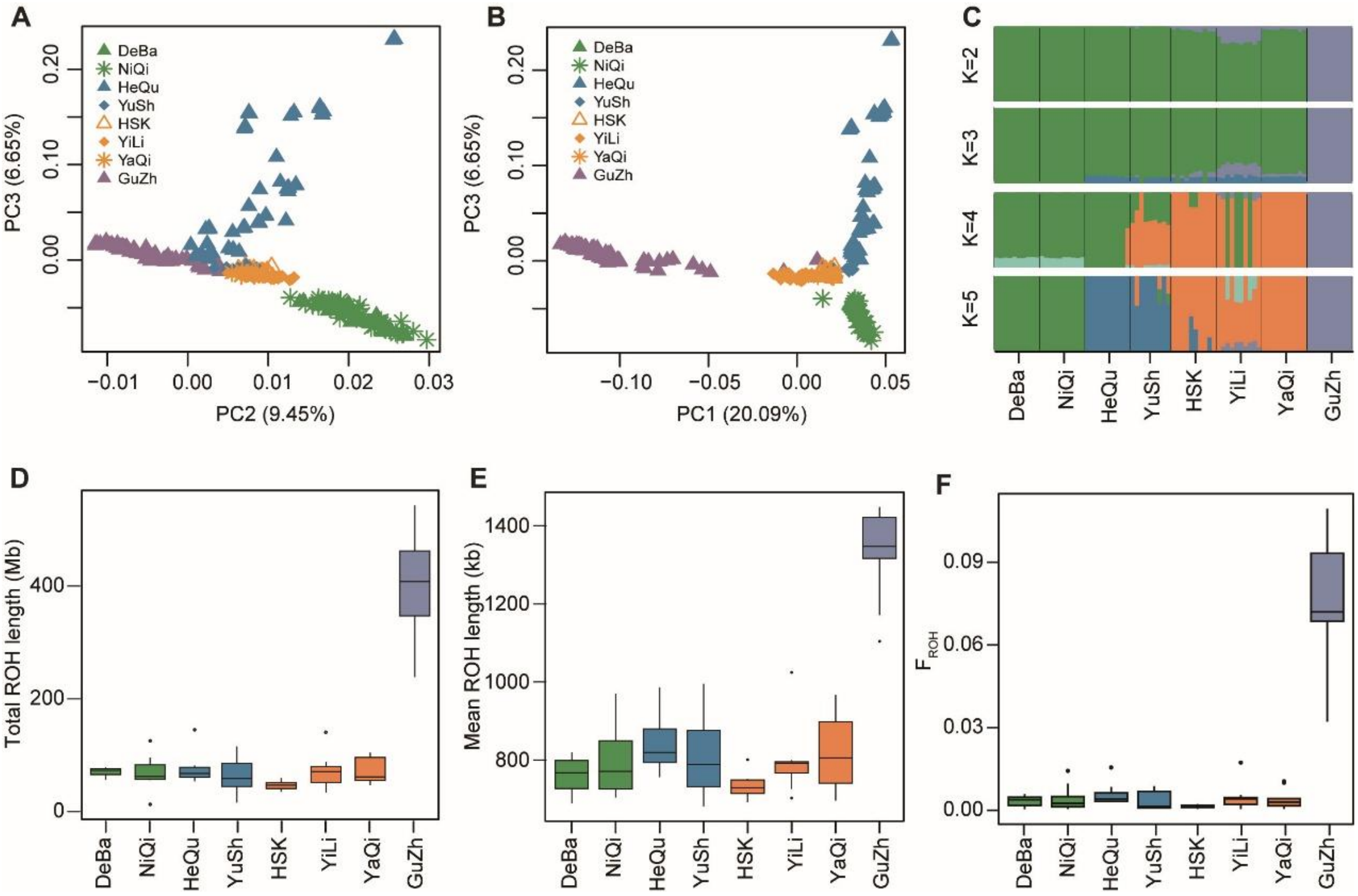

Figure S1. Population structure and length of Runs of Homozygosity (ROH), related to Figure 1.

(A) Principal Component Analysis (PCA) of autosomal SNP variation. The fractions of the total variance explained by PC2 and PC3 are reported between parentheses on each axis. DeBa=Debao, GuZh=Guanzhong, HeQu=Hequ, HSK=Kazakh, NiQi=NiQiang, YaQi=YanQi, YiLi=YiLi, YuSh=YuShu.

(B) same as **(A)**, for PC1 and PC3.

(C) ADMIXTURE profiles for 10 randomly selected individuals per breed. Analyses assumed K=2 to K=5 genetic ancestry components, with K=4 representing the optimal value according to cross-validation.

(D) Total ROH length (Mb).

(E) Mean ROH length (kb).

(F) Inbreeding Coefficient based on ROH (F_{ROH}).

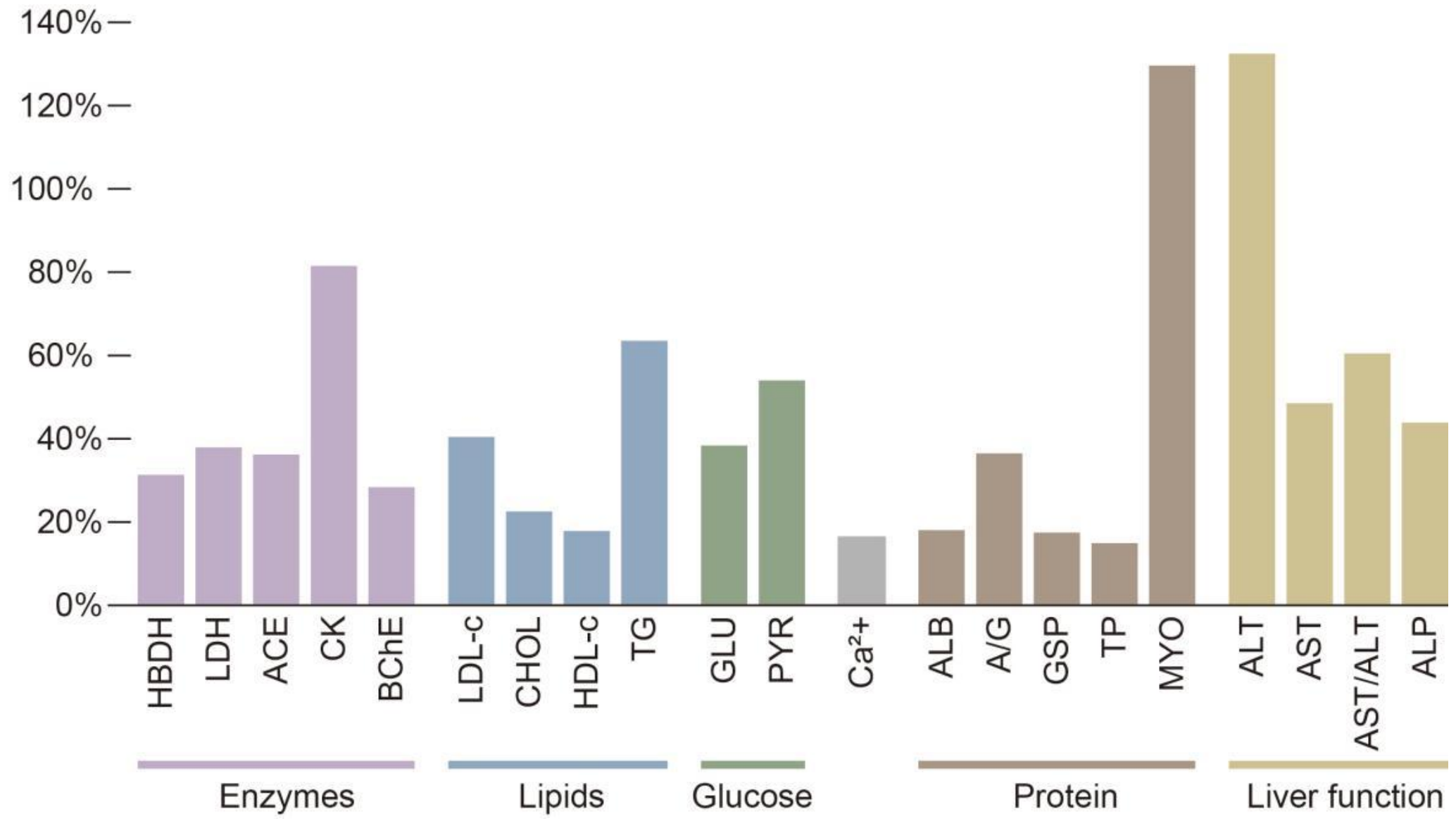

Figure S2. Coefficient of variation for 21 serum biochemical parameters, related to Figure 1. Serum parameters include: A/G=globulin ratio, ACE=angiotensin-converting enzyme activity (U/L), ALB=albumin concentration (g/L), ALP=alkaline phosphatase activity (U/L), ALT=alanine amino transferase activity (U/L), AST=aspartate amino transferase activity (U/L), AST/ALT=ratio of AST and ALT, BChE=butyrylcholinesterase activity (U/mL), Ca^{2+}=calcium concentration (mmol/L), CHOL=cholesterol concentration (mmol/L), CK=creatine kinase activity (U/L), GLU=glucose concentration (mmol/L), GSP=glycosylated serum protein concentration (μg/L), HBDH=α-hydroxy-butyrate dehydrogenase activity (U/L), HDL-c=high density lipoprotein cholesterol concentration (mmol/L), LDH=lactate dehydrogenase activity (U/L), LDL-c=low density lipoprotein cholesterol concentration (mmol/L), MYO=myoglobin concentration (μg/L), PYR=pyruvic acid concentration (mmol/L), TG=triglyceride concentration (mmol/L), and; TP=total protein concentration (g/L). Coefficients of variation were calculated by dividing the standard deviation by the average and are expressed in percentage. Colors indicate the main class to which each parameter belongs to.

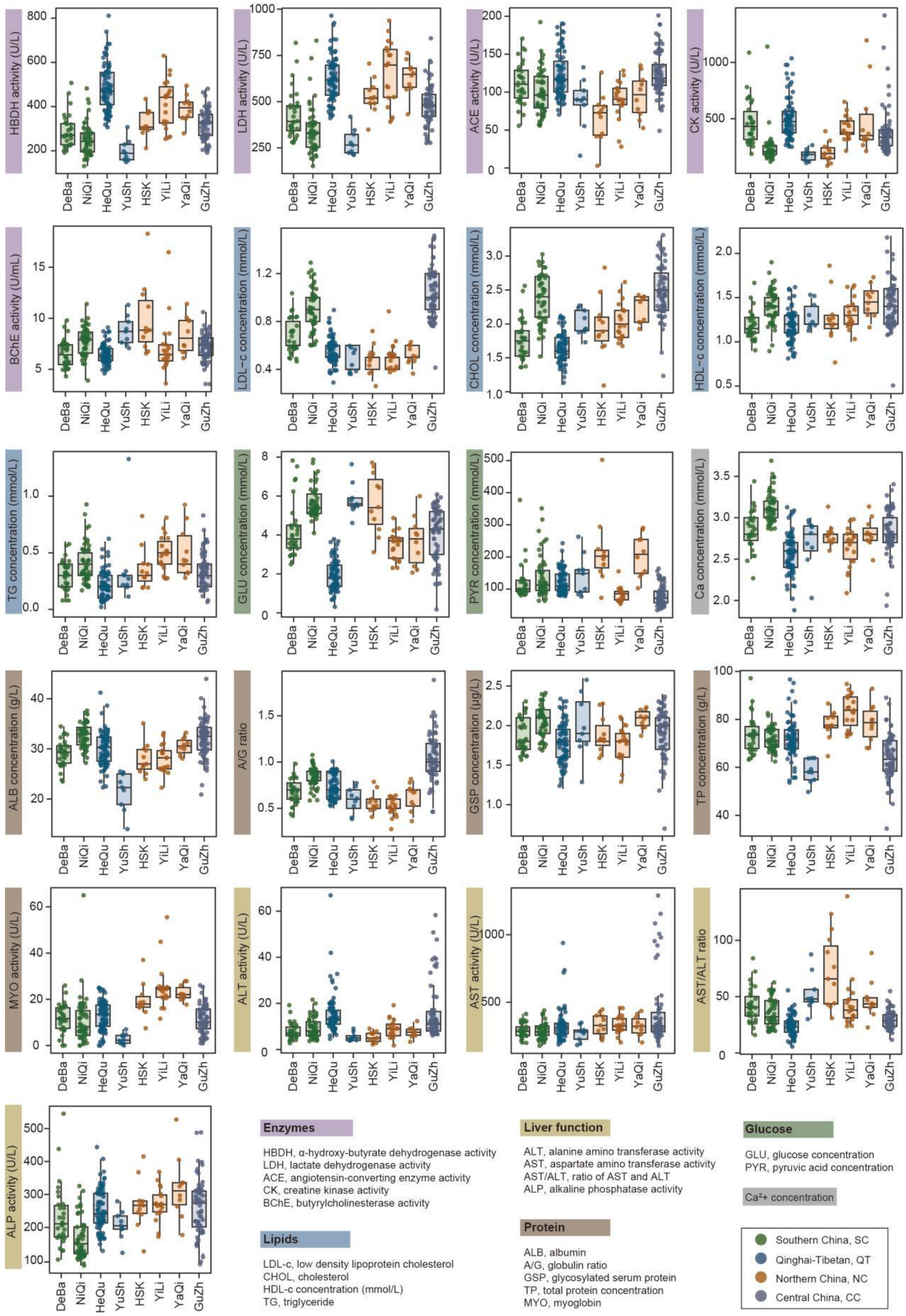

Figure S3. Individual measurements of 21 serum biochemical parameters in eight breeds of Chinese horses, related to Figure 1. DeBa=Debao, NiQi=Niqiang, HeQu=Hequ, YuSh=Yushu, GuZh=Guanzhong, HSK=Kazakh, YaQi=Yanqi, and YiLi=Yili.

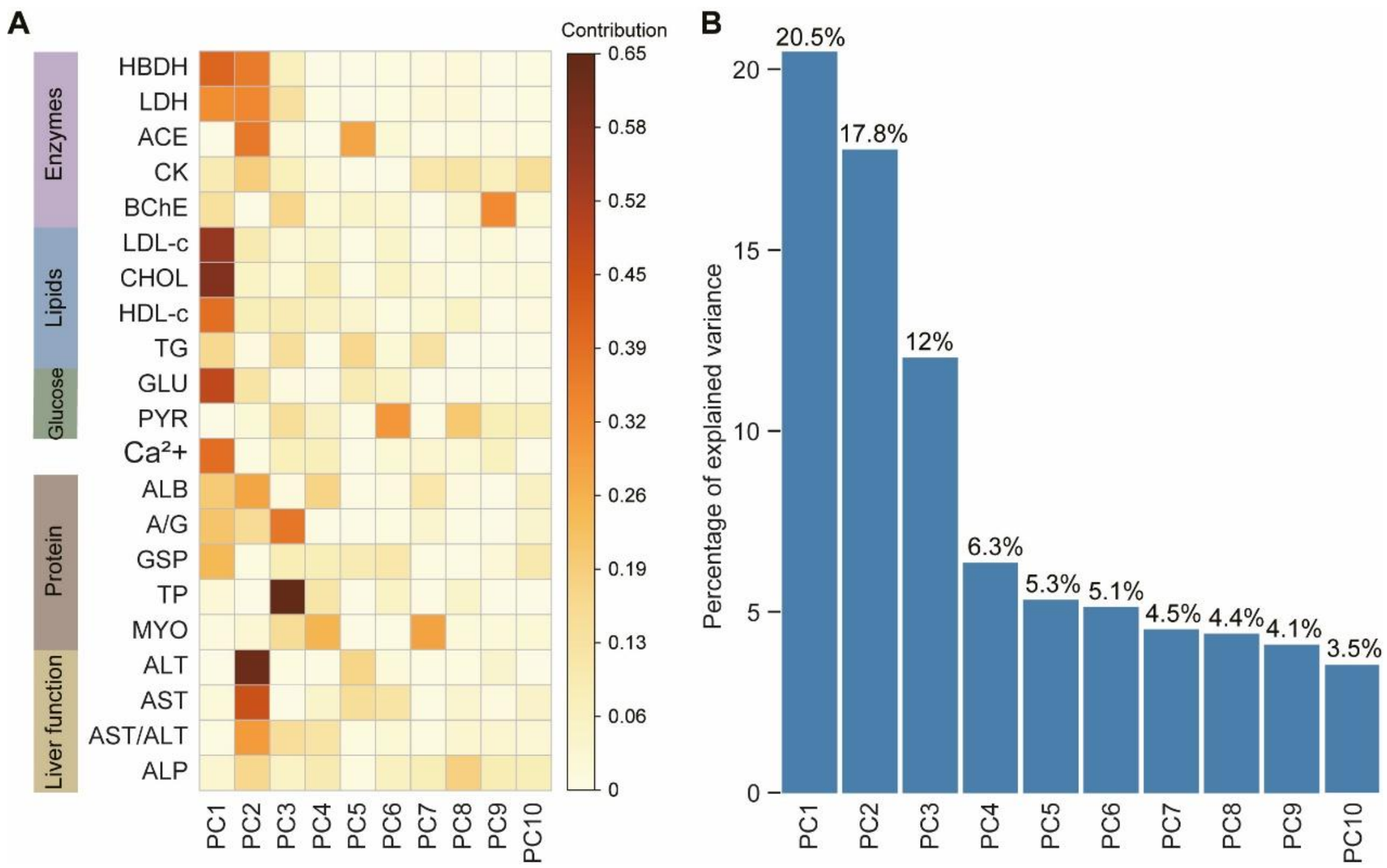

Figure S4. Principal Component Analysis of the variation measured in 21 serum biochemical parameters, related to Figure 1.

(A) Weight contribution of each serum biochemical parameter to the first 10 principal components.

(B) Fraction of the total phenotypic variance explained by the first 10 principal components. Combined, the first ten components explain 84.5% of the total variance. The location of each horse on the PCA space represented by the first two components is shown on Fig. 1c, and color-coded according to the breeds they belong to.

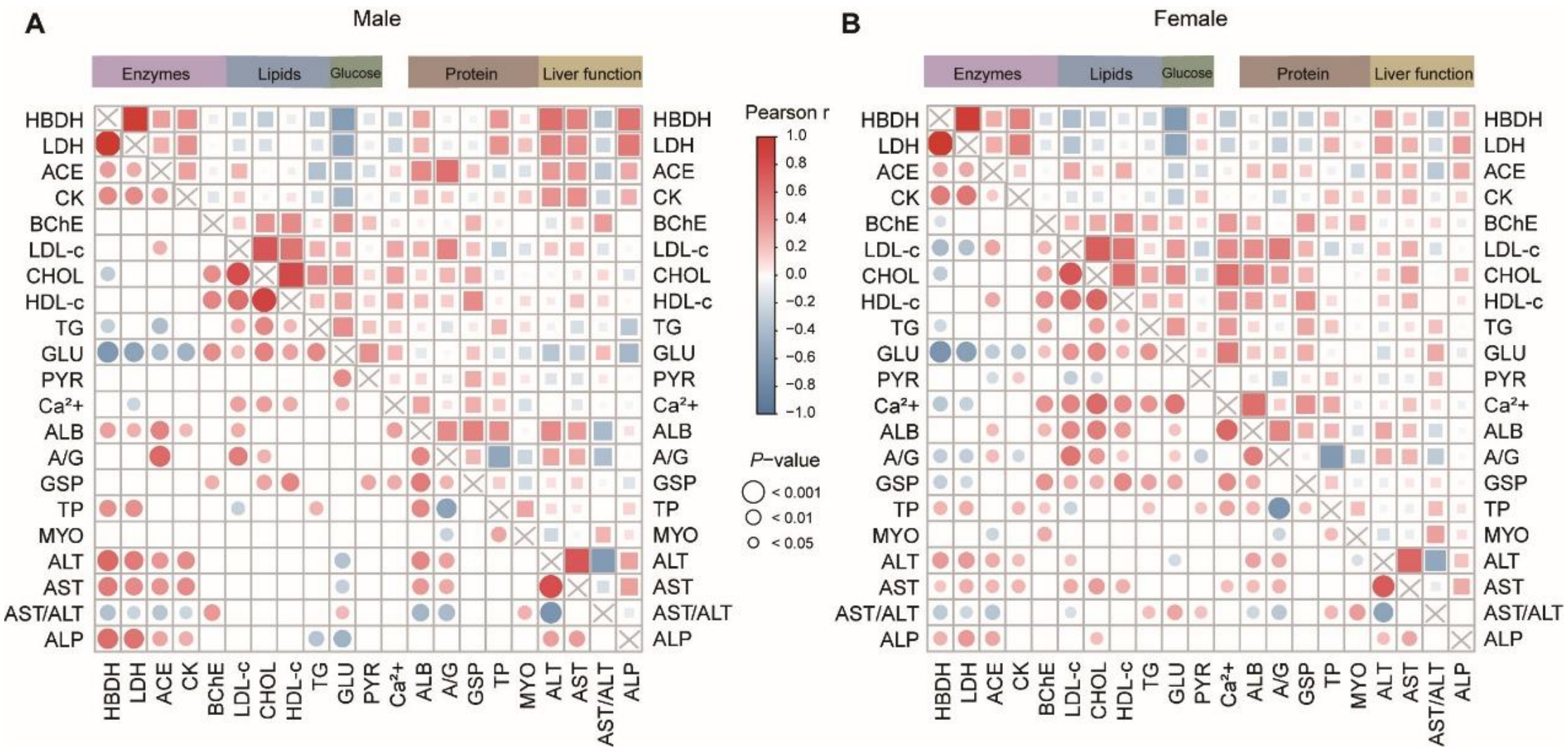

Figure S5. Matrices of pairwise Pearson correlations between biochemical parameters in males (A) and females (B) , related to Figure 1. The colors above the main diagonal are proportional to the correlation coefficients, while the size of the circles below the main diagonal indicate statistical significance.

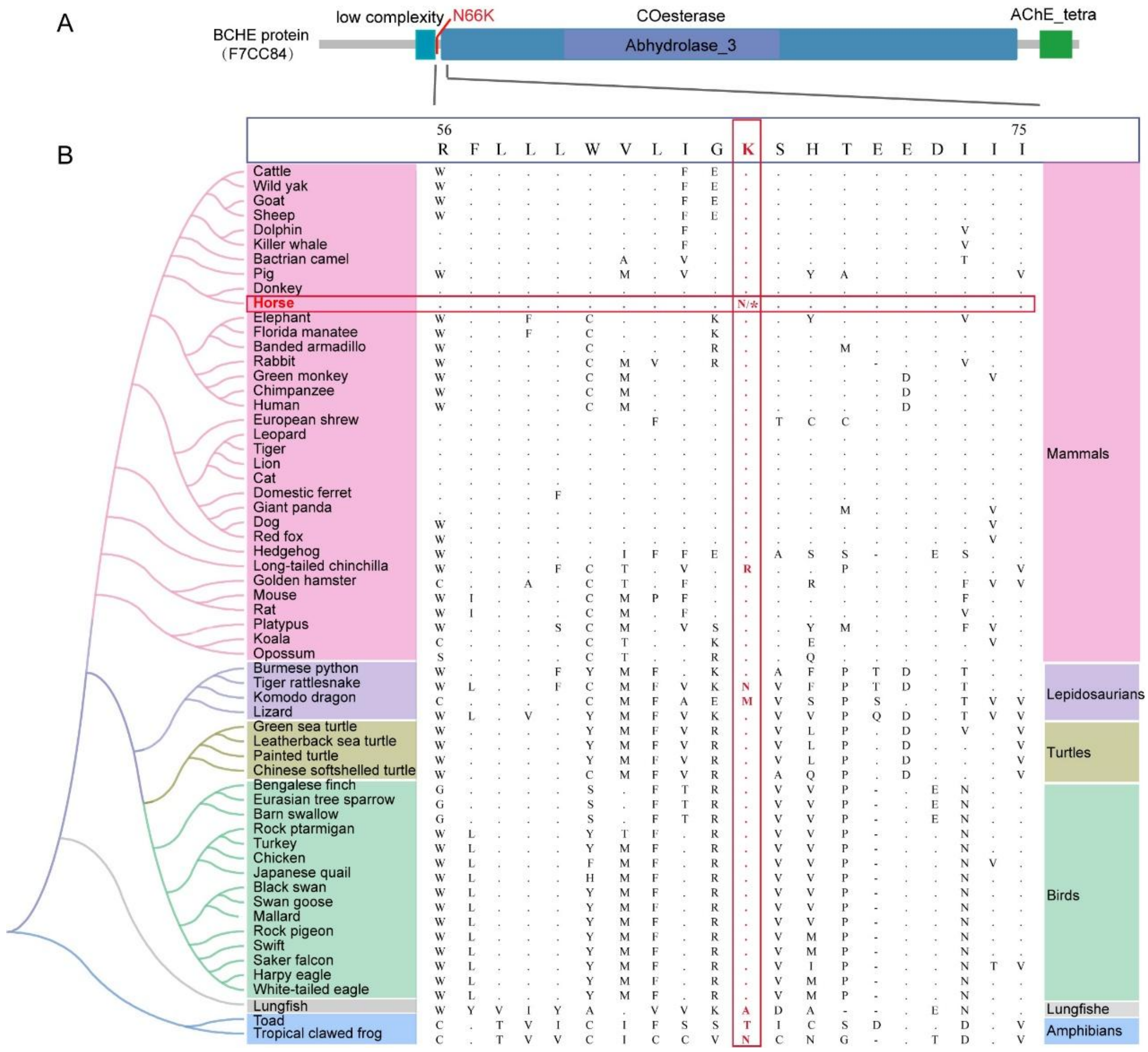

Figure S6. Conservation analysis of BCHE protein sequence, related to Figure 2.

(A) BCHE horse protein domains (AChE_tetra=Acetylcholinesterase tetramerization domain, COesterase= Carboxylesterase family, Abhydrolase_3, and; low-complexity domain).

(B) Sequence alignment between amino-acids 56 and 75 of 60 Vertebrate BCHE proteins (Table S6). The Neighbor-Joining tree represents the phylogenetic relationships between sequences along the entire BCHE protein, as obtained from MEGA X.

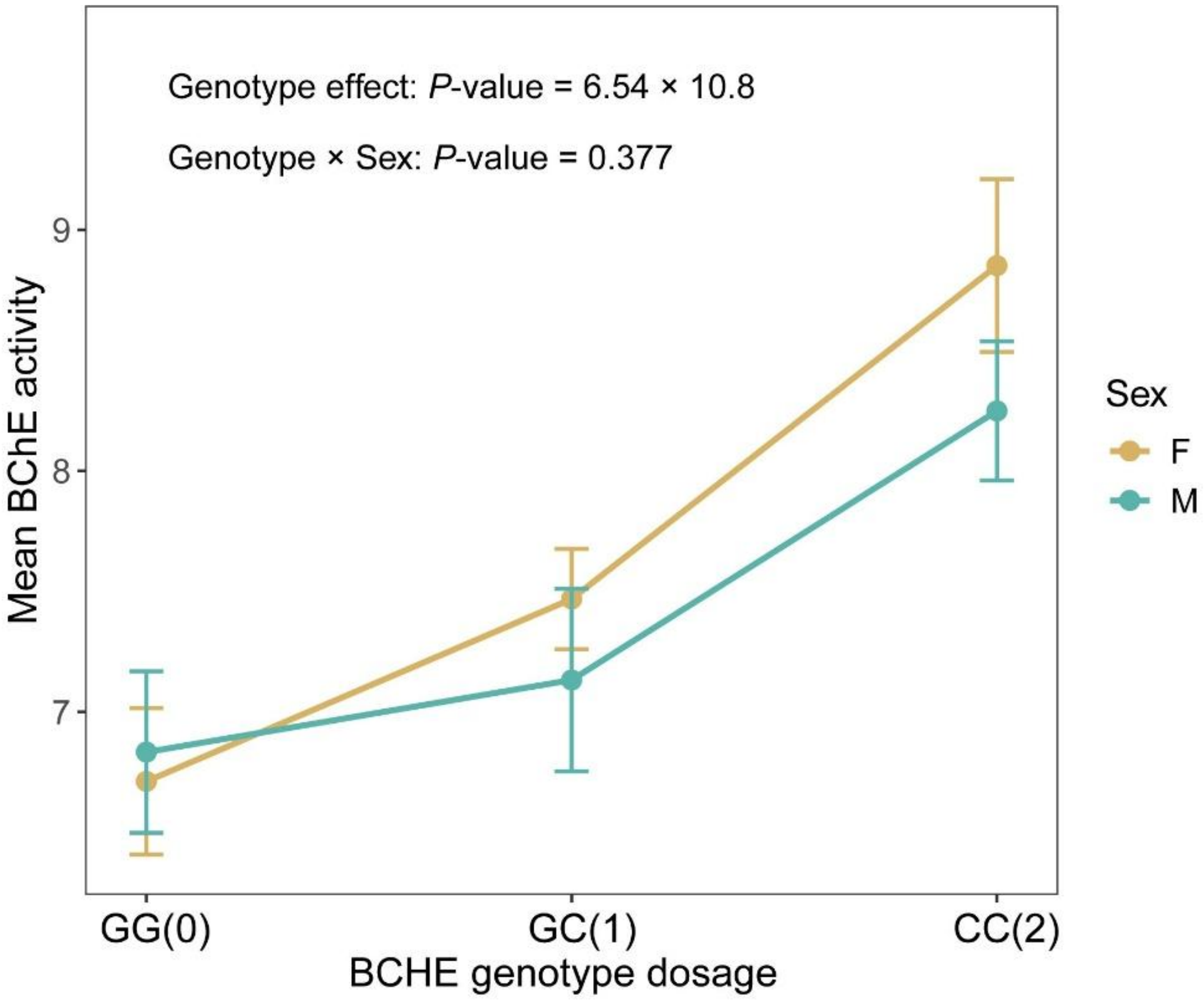

Figure S7. Sex-stratified association between the BCHE missense variant (ECA19:9,198,989, G/C) and BChE activity, related to Figure 2. Mean BChE activity is shown by genotype dosage of the BCHE missense variant (ECA19:9,198,989, G/C) separately for females and males, with error bars indicating standard errors. BChE activity increased with the "C"-allele dosage in both sexes. The variant effect remained significant after adjustment for sex and other covariates ($\beta = 1.04$, *P*-value = 6.54×10^{-8}), whereas the variant × sex interaction was not significant (*P*-value = 0.377).

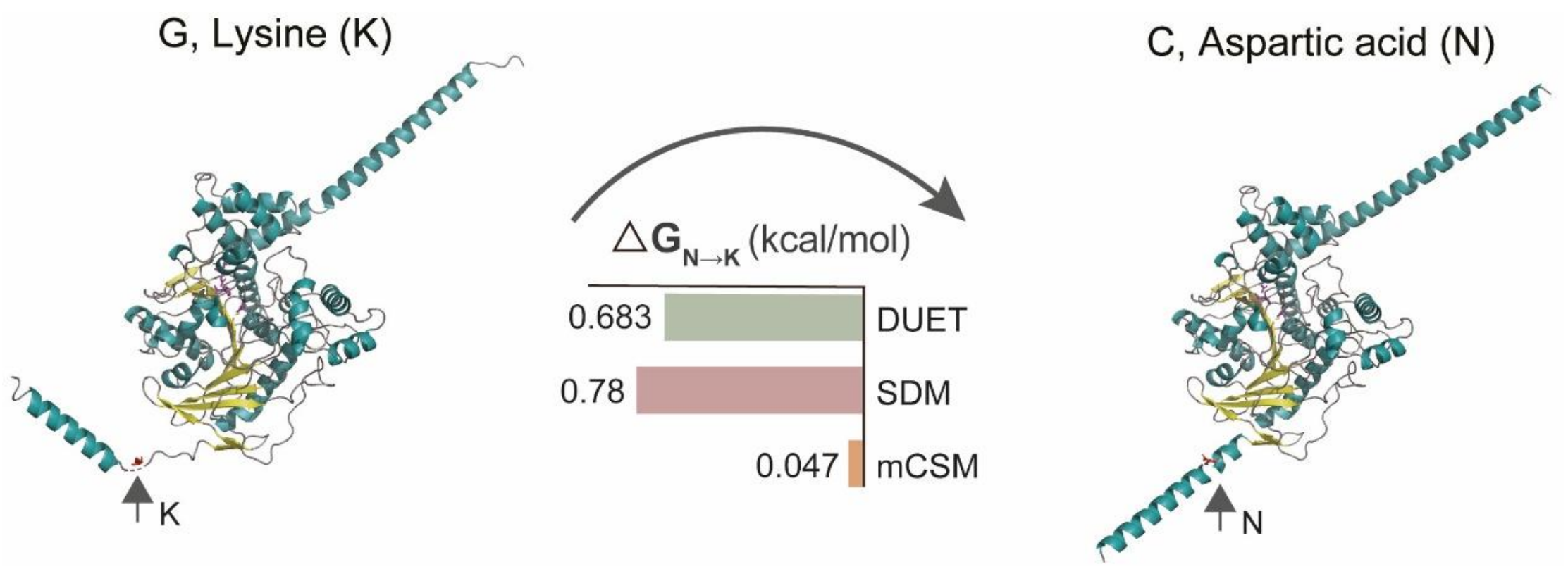

Figure S8. Predicted secondary structures for two genetic variants of the horse BCHE protein, related to Figure 2. The two variants modeled correspond to the "C" and "G" alleles identified at position ECA19:9198989. Colors represent various secondary structures, including alpha-helices (blue), beta-sheets (yellow), and random coils (grey). The arrows indicate the position of the mutated amino acids, with the "C" allele translating into Aspartic acid (N), and the "G" allele into Lysine (K). The middle panel displays the Gibbs free energy variation for the two protein models, as predicted using the trRosetta online server (http://struct.bioc.cam.ac.uk/duet), and assuming DUET, mCSM, and SDM methods.

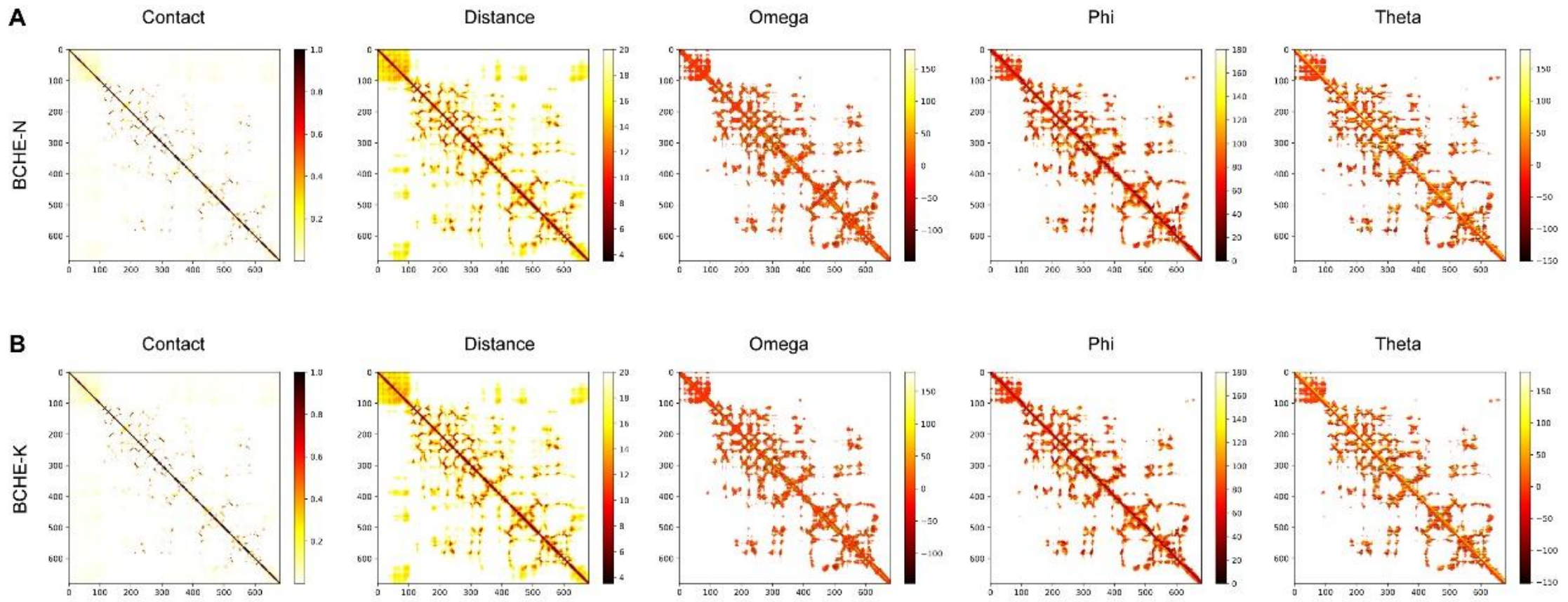

Figure S9. Predicted inter-residue distance and orientations distributions for BCHE variants, related to Figure 2. Panels display the output matrices from the trRosetta structure prediction network for the **(A)** horse-C and **(B)** horse-G protein sequences. For each variant, the five probabilistic distributions are shown: Contact (probability of residue pair contact), Distance (Å, between Cβ atoms), Omega (dihedral angle around the Cβ-Cβ virtual bond, °), Phi (rotation angle around the virtual Cβ-Cβ bond, °), and Theta (angle between Cα-Cβ vectors, °). These matrices represent the network's confidence and are used as constraints to generate the final 3D structural models.

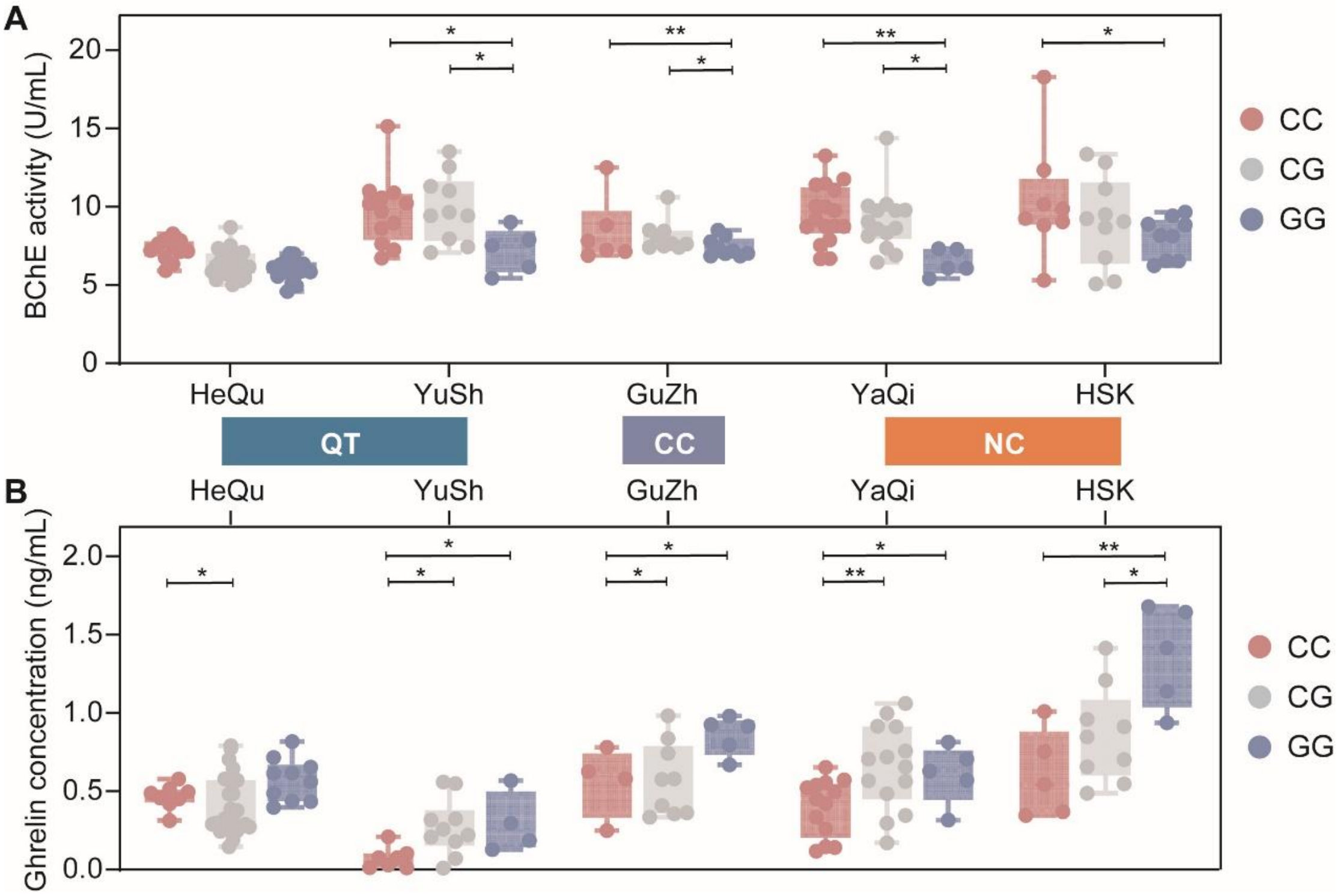

Figure S10. Serum BChE activity (U/mL) (A) and ghrelin concentration (ng/mL) (B) in five horse breeds for the CC, CG and GG genotypes at ECA19:9198989, related to Figure 2. Statistical significance was assessed using Kruskal-Wallis tests (*, *P*-value<0.05; *, *P*-value<0.01; **). Because the C allele is the main allele of interest in this study, genotypes are displayed as CC, CG, and GG in genotype-comparison panels.

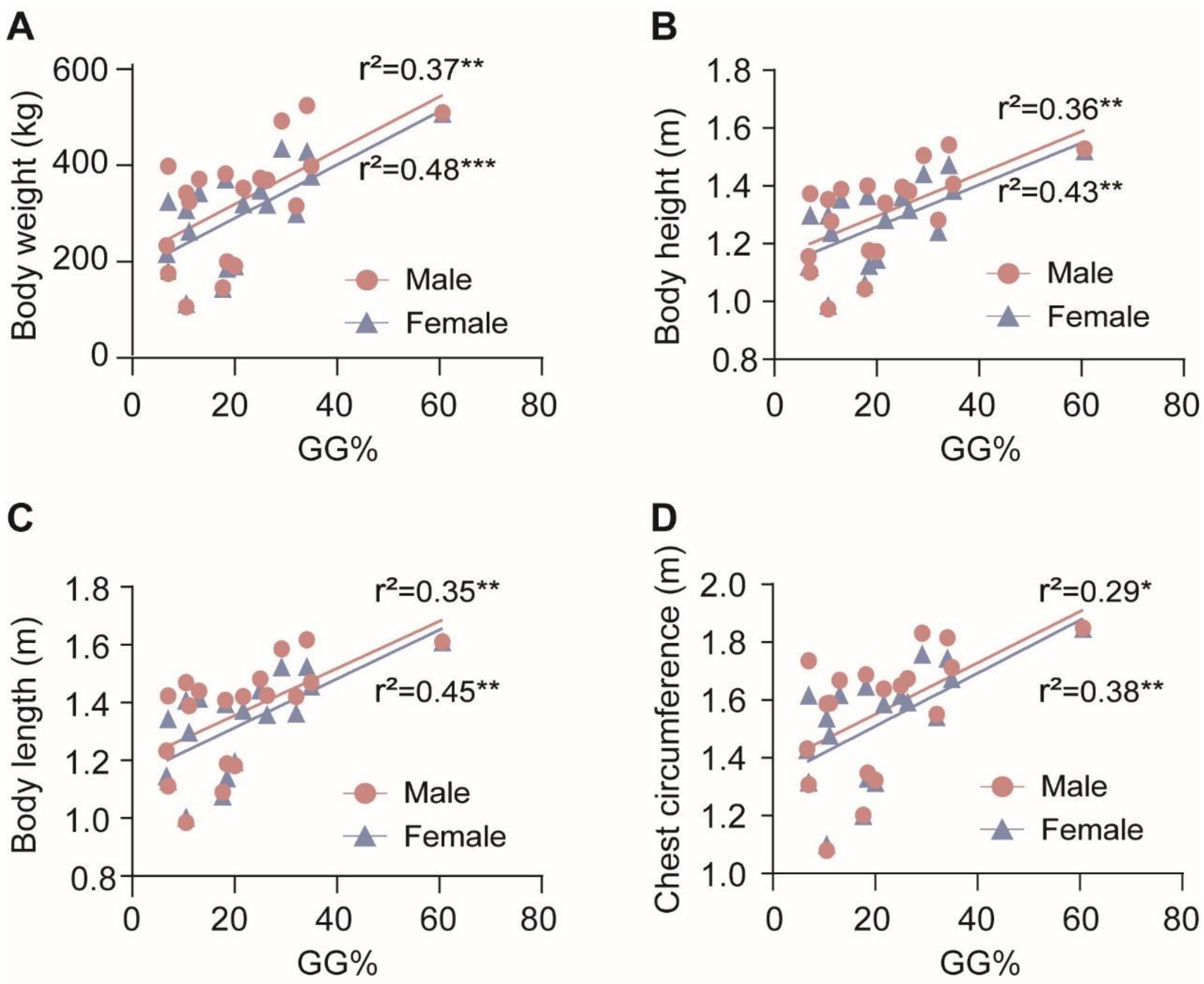

Figure S11. Sex-specific linear regression analysis between horse the frequency of the GG genotype at ECA19:9198989, and weight (A), body height (B), body length (C), chest circumference (D) , related to Figure 2. Red circles and blue triangles represent males and females, respectively. All body size data were from China National Commission of Animal Genetic Resources. R-squared Pearson correlation coefficients are indicated next to their respective models (*, *P*-value<0.05; **, *P*-value<0.01; ***, *P*-value<0.001).

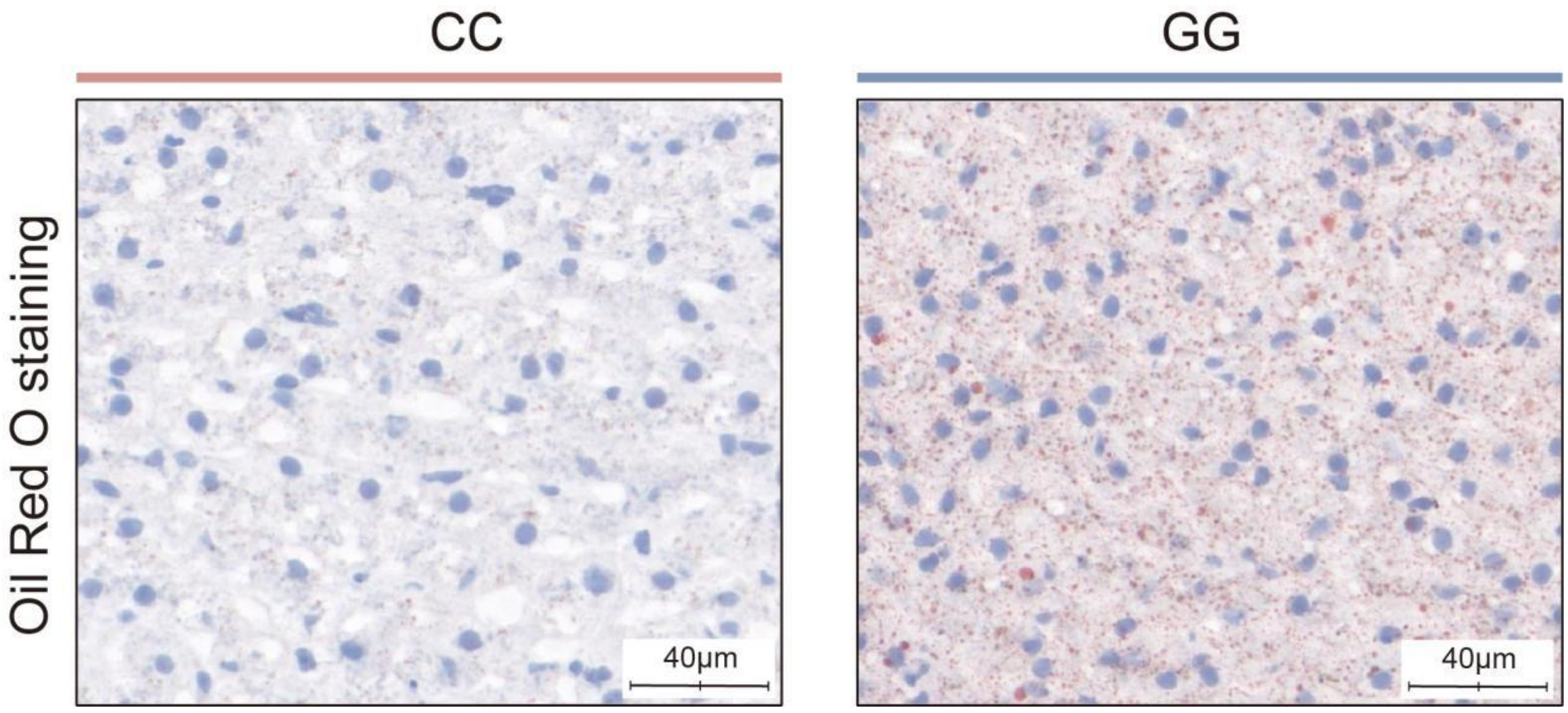

Figure S12. Oil red O staining of horse liver sections for two homozygous individuals at ECA19:9198989, related to Figure 2. The genotype of each individual is reported on the picture. Scale bar=40 μm.

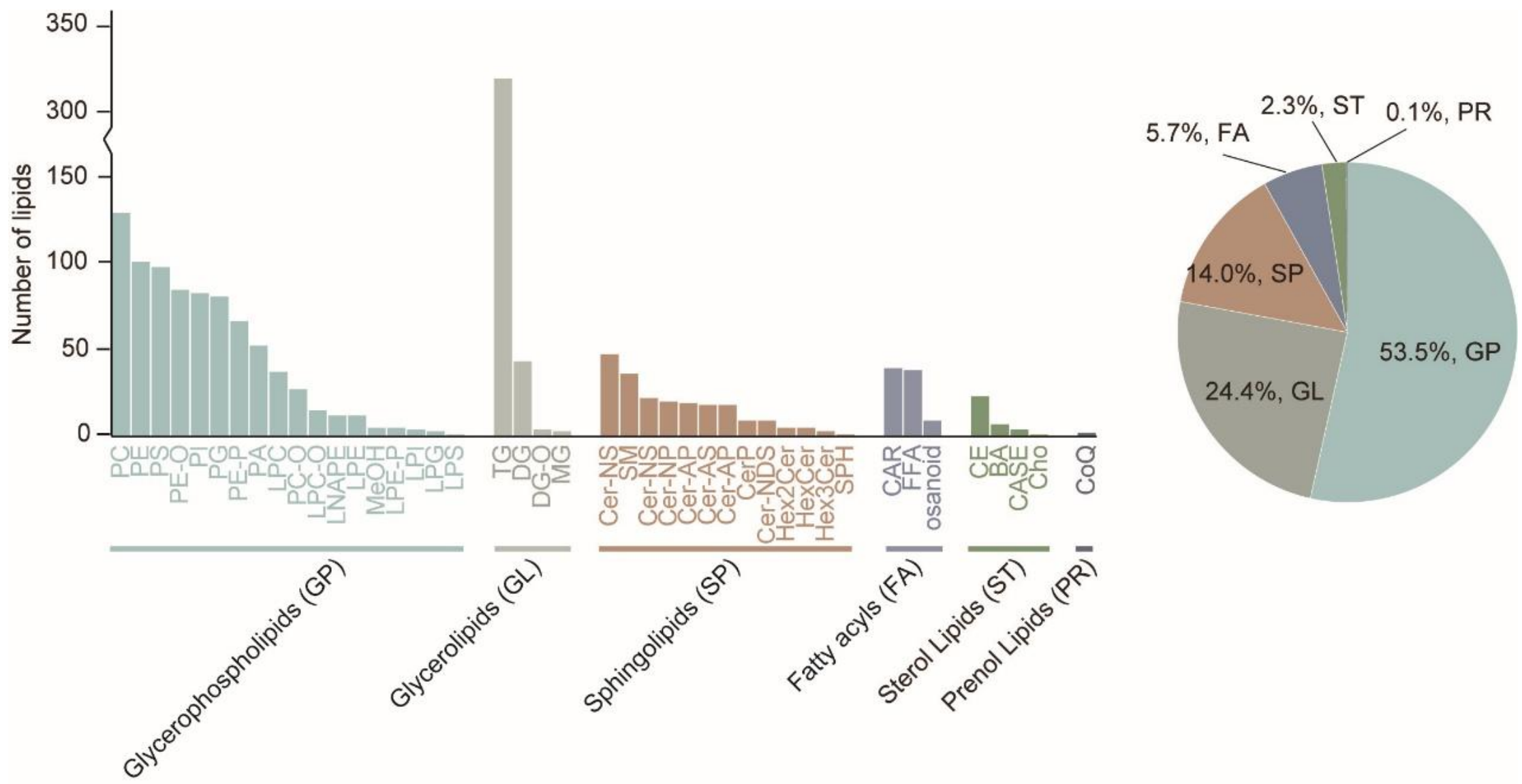

Figure S13. Distribution of the 1,515 lipids analyzed in this study, related to Figure 2. Lipids were divided into six categories, including: Glycerophospholipids (GP, 53.5%), Glycerolipids (GL, 24.4%), Sphingolipids (SP, 14.0%), Fatty acyls (FA, 5.7%), Sterol Lipids (ST, 2.3%), and; Prenol Lipids (PR, 0.1%). These six categories are shown in different colors, and include a total of 43 biochemical sub-classes.

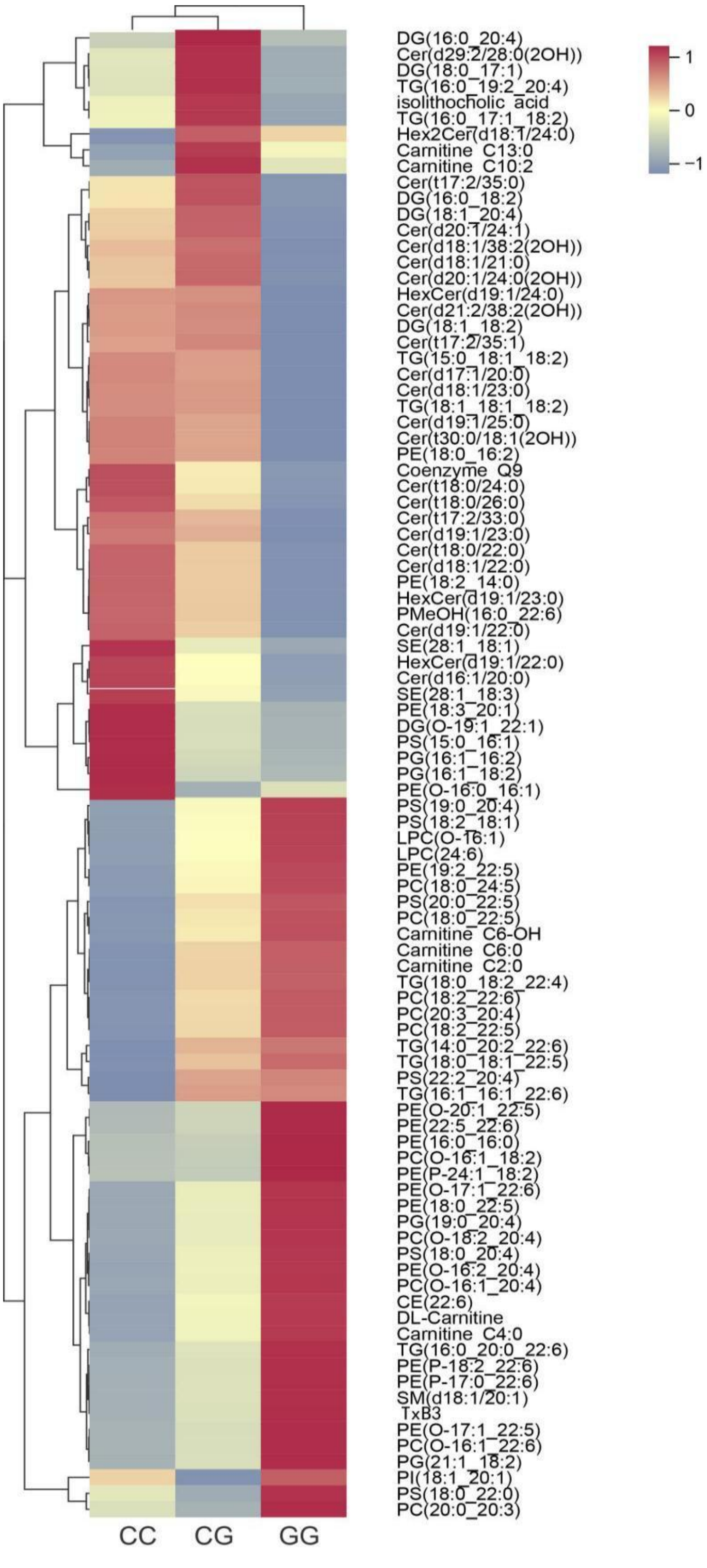

Figure S14. Hierarchical clustering heatmap of blood relative concentrations for 93 lipids showing strong statistical association with BCHE genotypes, related to Figure 2. The set of lipids was identified following orthogonal partial least square-discriminant analysis (OPLS-DA), based on variable importance in projection (VIP) values >1, |fold change, FC|≥1.5, and *P*-values <0.05.

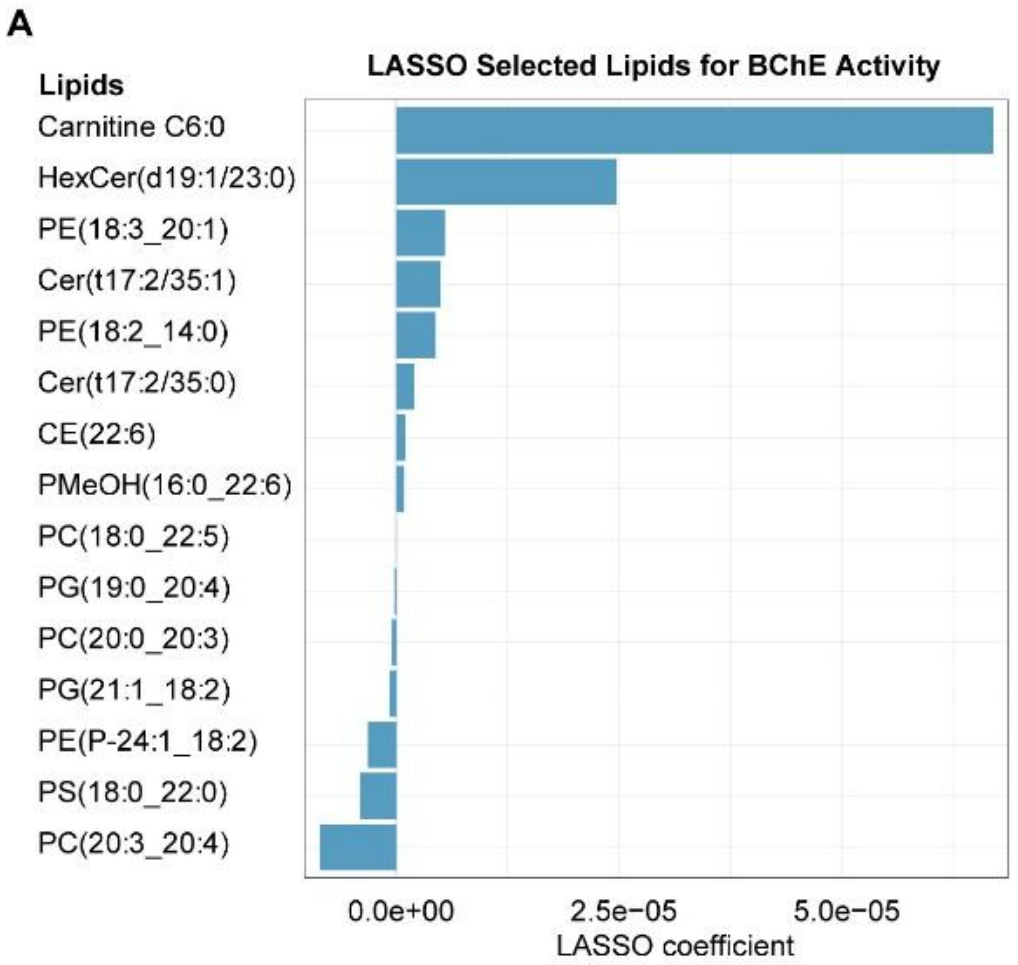

B

Lipids	LASSO coefficient	Spearman_r	*p*_value
Carnitine C6:0	6.69E-05	0.0482	0.6238
HexCer(d19:1/23:0)	2.47E-05	0.2036	0.0363
PE(18:3_20:1)	5.47E-06	0.1671	0.0869
Cer(t17:2/35:1)	4.95E-06	0.2496	0.0099
PE(18:2_14:0)	4.40E-06	0.1400	0.1523
Cer(t17:2/35:0)	2.01E-06	0.2448	0.0114
CE(22:6)	1.03E-06	0.1803	0.0643
PMeOH(16:0_22:6)	8.79E-07	0.1154	0.2387
PC(18:0_22:5)	1.22E-08	0.2044	0.0356
PG(19:0_20:4)	-1.87E-07	-0.1695	0.0823
PC(20:0_20:3)	-4.94E-07	-0.0591	0.5470
PG(21:1_18:2)	-7.36E-07	-0.1993	0.0406
PE(P-24:1_18:2)	-3.17E-06	-0.1653	0.0903
PS(18:0_22:0)	-4.09E-06	-0.0826	0.399
PC(20:3_20:4)	-8.55E-06	0.0497	0.6133

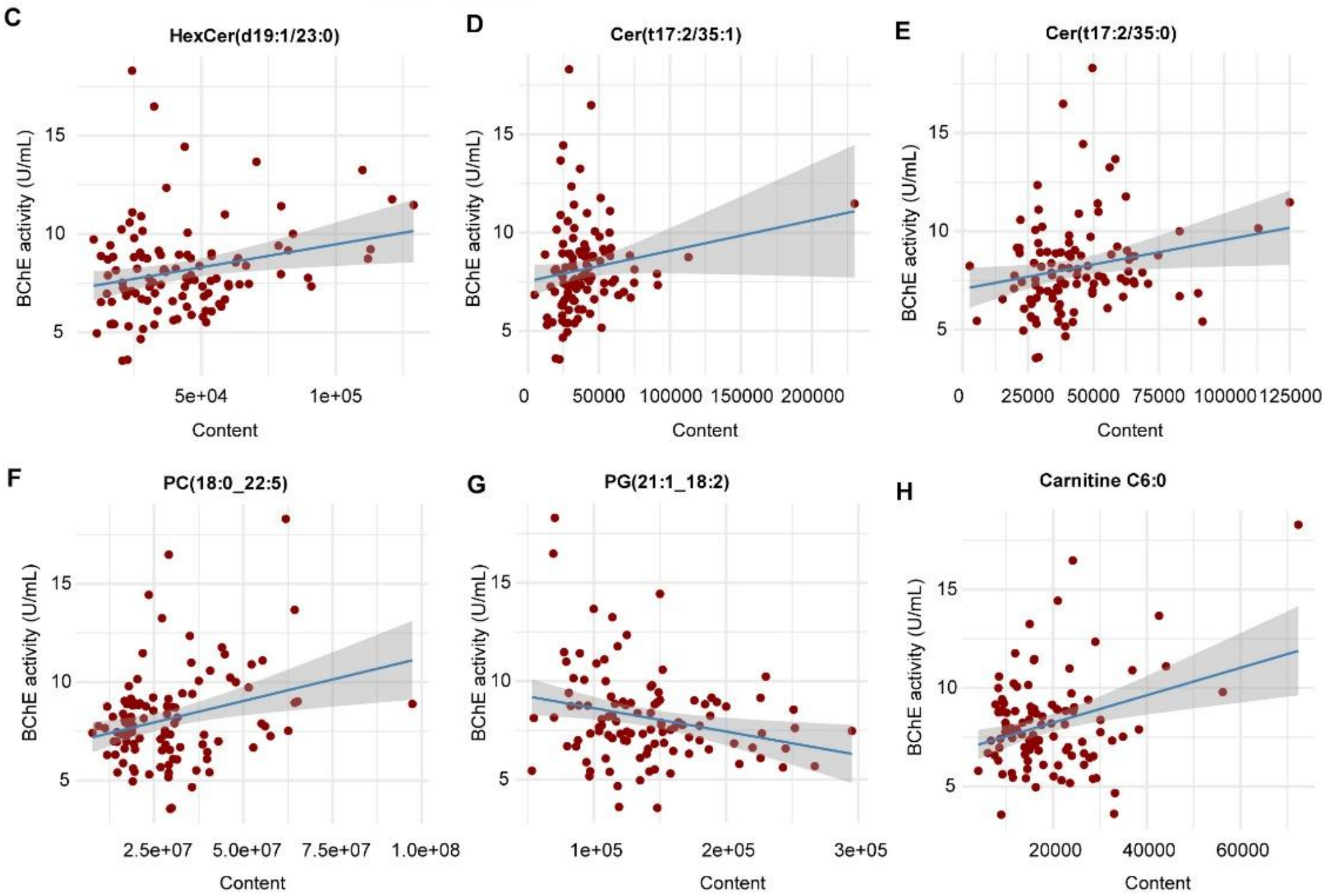

Figure S15. Least absolute shrinkage and selection operator (LASSO) regression analysis for the identification of lipid features associated with BChE activity, related to Figure 2.

(A) LASSO coefficients of the selected lipids contributing to BChE activity. LASSO regression analysis of 93 differential lipids identified from pairwise comparisons resulted in 15 lipids with non-zero coefficients.

(B) Table summarizing LASSO coefficients together with Spearman correlation coefficients (r) and corresponding *p*_values between lipid abundances and BChE activity.

(C–H) Scatter plots showing the correlations between BChE activity and six lipids (HexCer(d19:1/23:0), Cer(t17:2/35:1), Cer(t17:2/35:0), PC(18:0_22:5), PG(21:1_18:2), and Carnitine C6:0) displaying strong associations supported by both LASSO selection and Spearman correlation analysis. Spearman's r and *p*_values are indicated in each panel.

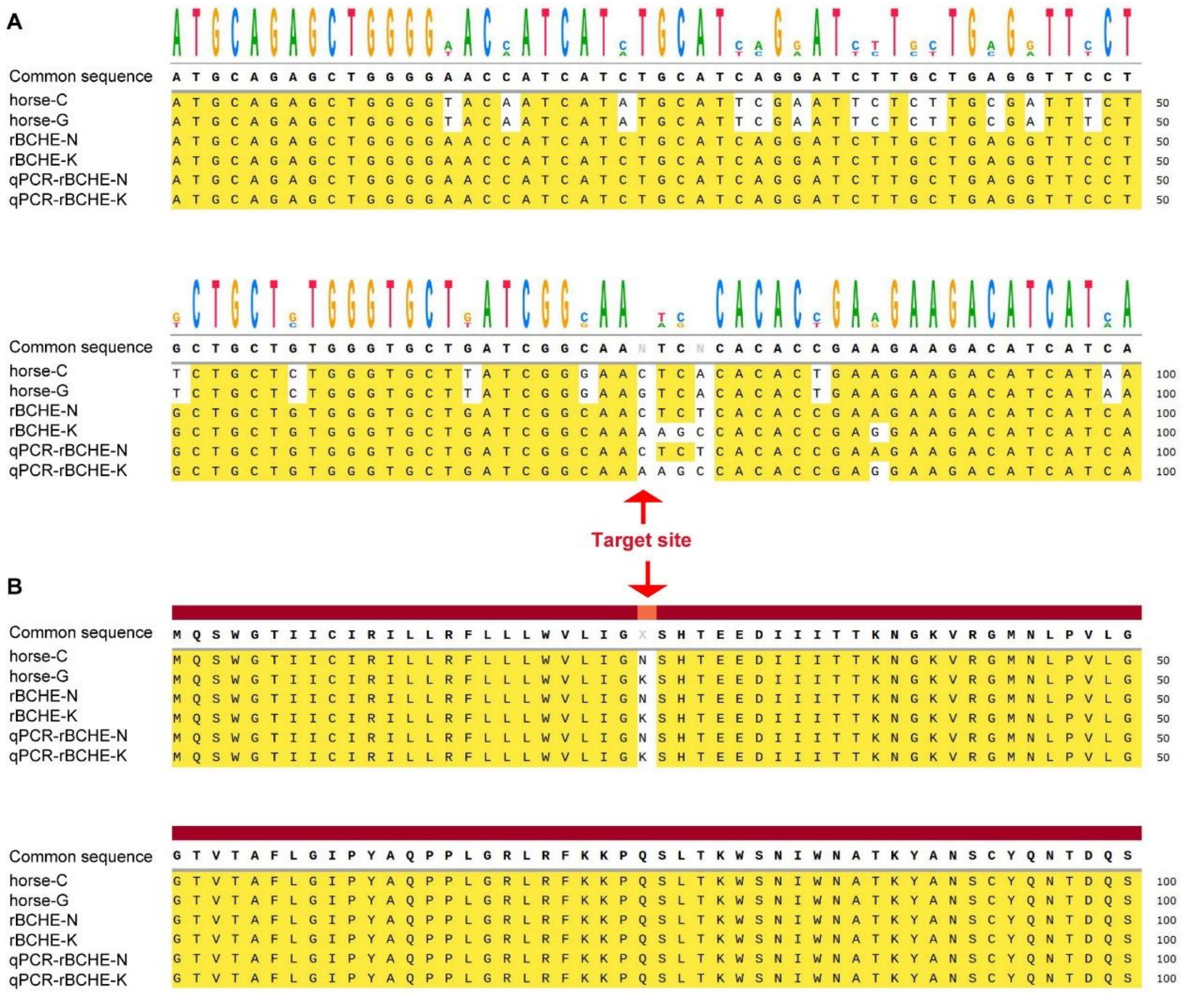

Figure S16. Sequence alignment of endogenous and optimized BCHE sequences, related to Figure 3.

(A) Nucleotide sequence alignment.

(B) Amino acid sequence alignment. Sequences include the endogenous equine genes (horse-C and horse-G), the constructed overexpression vectors (rBCHE-N and rBCHE-K), and the sequences amplified from RT-PCR products (qPCR-rBCHE-N and qPCR-rBCHE-K).

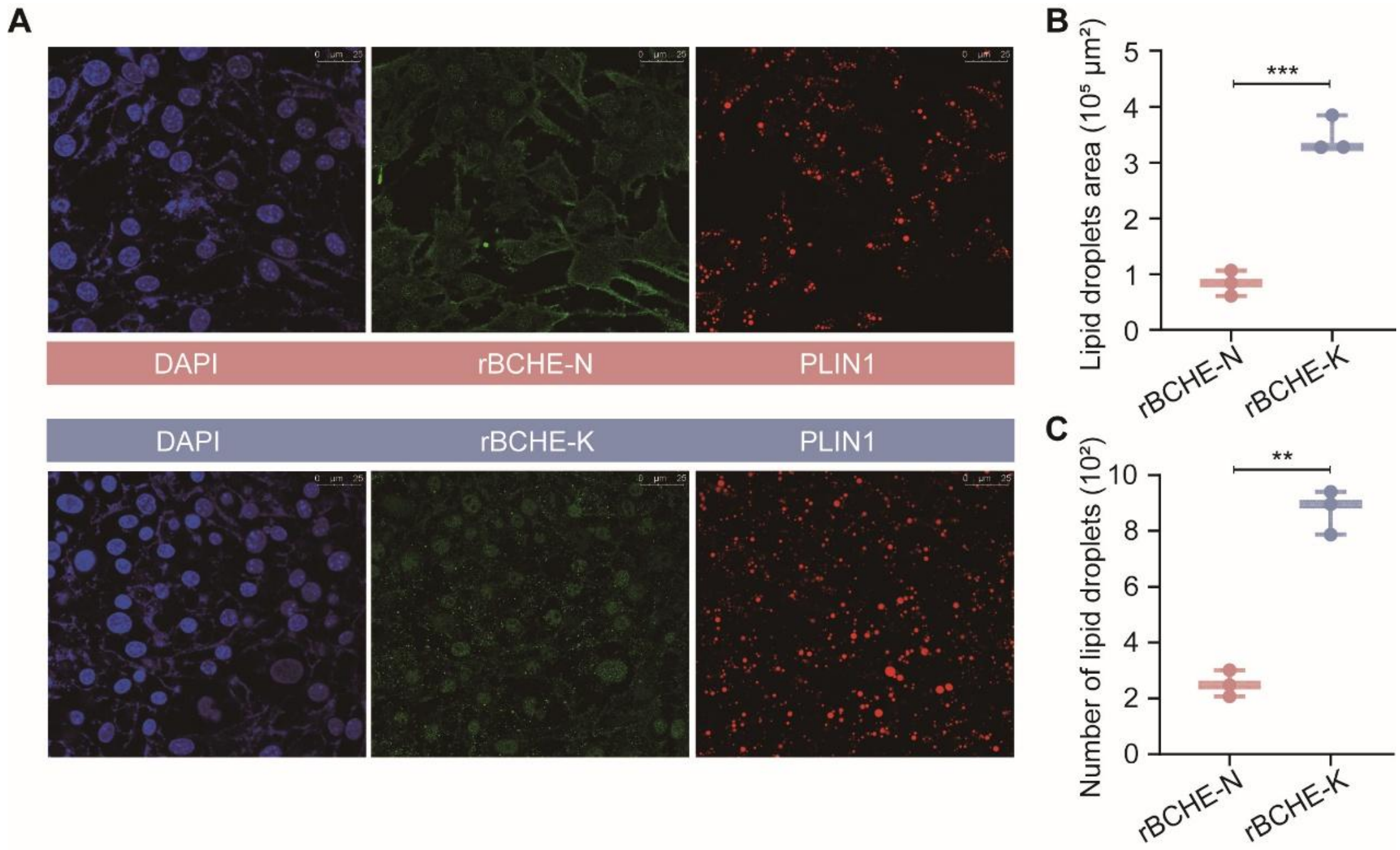

Figure S17. Immunofluorescence (IF) staining and quantification of flag-tagged BCHE and PLIN1 in HepG2 cells, related to Figure 3.

(A) IF staining. DAPI (blue) was used to identify nuclei, while AF488 (green) and CY3 (red) were used for visualizing the BCHE and PLIN1 proteins. The 'Merge' panels represent the cumulated signal of all three markers. *Top*: experiments carried out on cells expressing the "C" allele of the BCHE gene. Bottom: experiments carried out on cells expressing the "G" allele of the BCHE gene. Scale bar=25μm.

(B) Total lipid area (10^5 um^2). The total area encompassed by PLIN1 staining, as measured in ImageJ, provides a proxy for the total lipid area present in the picture analyzed.

(C) Number of lipid droplets, as measured in ImageJ on the basis of PLIN1 staining. In these experiments, adipogenesis was induced in transfected HepG2 cells by supplementing the culture medium with a mixture of sodium oleate and sodium palmitate (v:v=2:1). Because the C allele encodes the BChE-N form, recombinant BChE groups are displayed as rBChE-N, and rBChE-K.

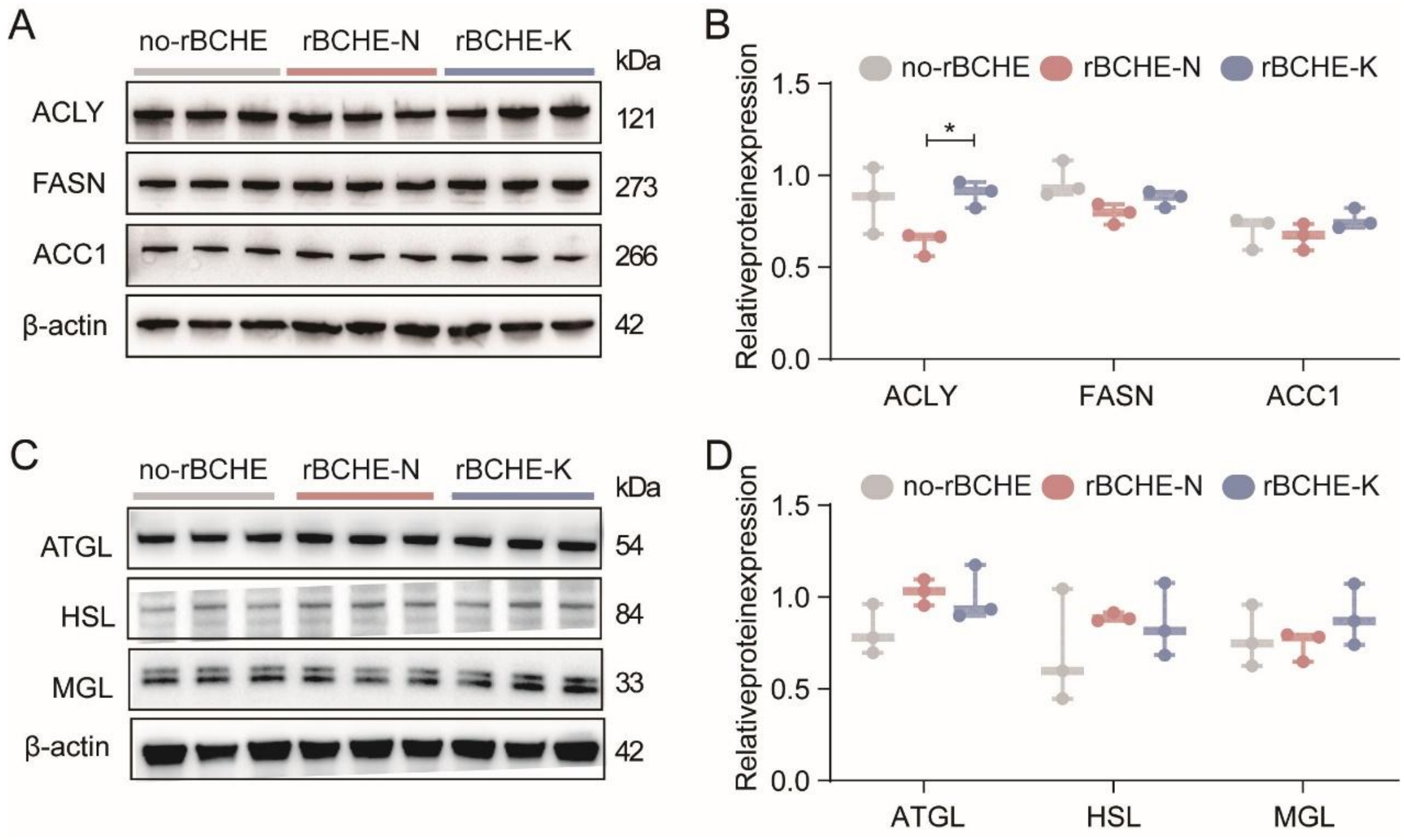

Figure S18. Lipogenesis (a) and lipolysis (b) control experiments in HepG2 cells, related to Figure 3.

(A) Western blot analysis of the levels of three proteins involved in lipogenesis. ACLY=ATP Citrate Lyase. FASN=Fatty Acid Synthase. ACC1=Acetyl-CoA Carboxylase Alpha. β-actin was used as a normalization control.

(B) Normalized protein levels of ACLY, FASN and ACC1, as measured with ImageJ.

(C) same as **(A)**, except that three proteins involved in lipolysis were considered. ATGL=Adipose Triglyceride Lipase. HSL=Hormone-Sensitive Lipase. MGL=Monoacylglycerol Lipase.

(D) Normalized protein levels of ATGL, HSL and MGL, as measured with ImageJ.

HepG2 cells were transfected with vectors carrying the “C” and “G” alleles of the BCHE, or an empty vector. Statistical significance was assessed using Kruskal-Wallis tests (*, P-value<0.05). Because the C allele encodes the BChE-N form, recombinant BChE groups are displayed as no-rBChE, rBChE-N, and rBChE-K.

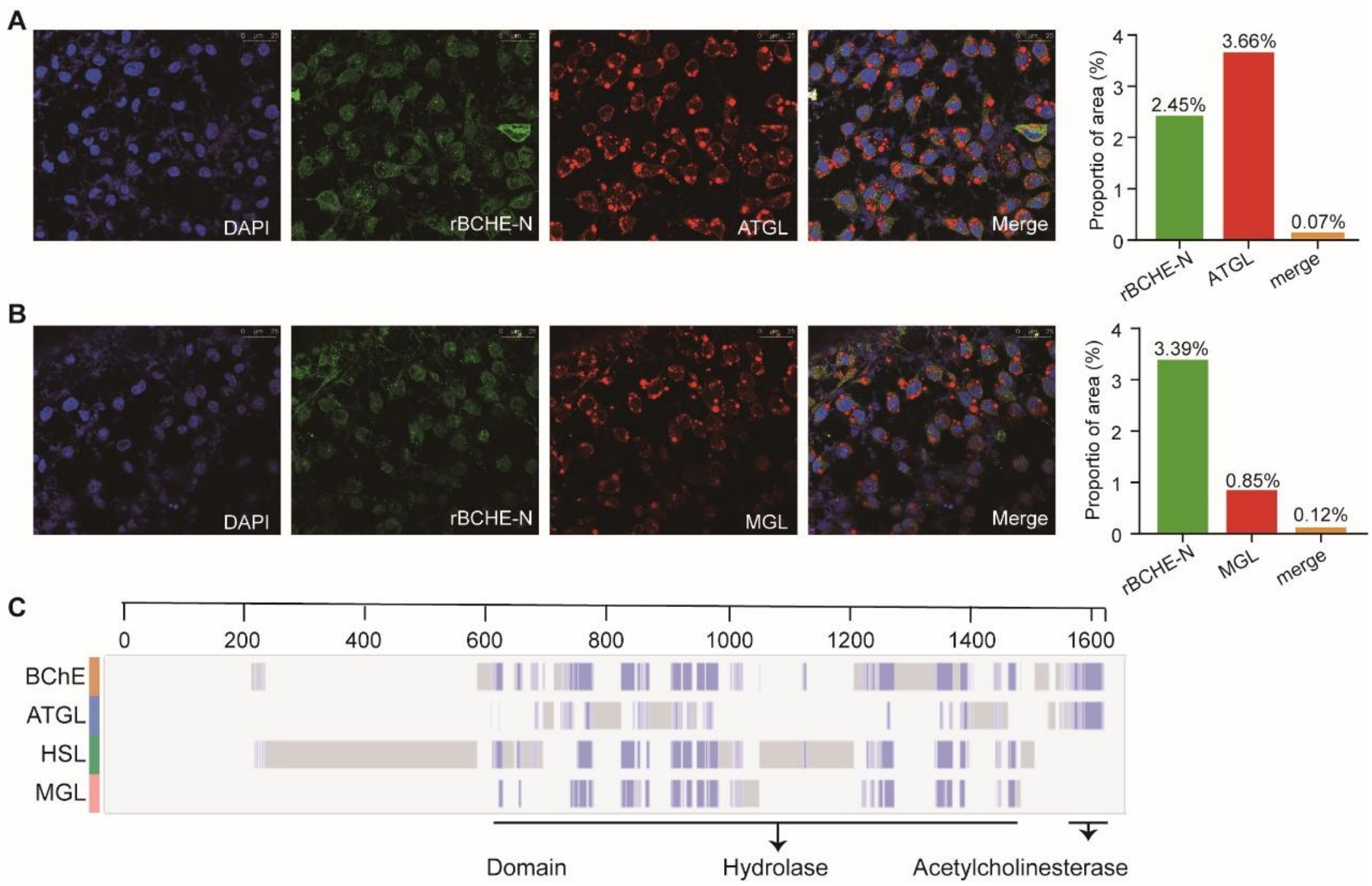

Figure S19. Immunofluorescence (IF) and structural comparison between four lipolytic enzymes, related to Figure 3. IF staining of nuclei, flag-tagged BChE, ATGL **(A)** (Adipose triglyceride Lipase), and MGL (Monoacylglycerol Lipase) **(B)** in HepG2 cells. DAPI (blue) was used to identify nuclei, while AF488 (green) and CY3 (red) were used for visualizing the BCHE and ATGL (top) or MGL (bottom) proteins. The 'merge' panels represent the cumulated signal of all three markers. Experiments were carried out on cells expressing the "C" allele of the *BCHE* gene. Scale bar=25μm. **(C)** Conservation analysis of the secondary structure of BCHE and classical lipolytic enzymes. Purple indicates similar protein secondary structures, whereas darkgrey indicates dissimilar secondary structures.

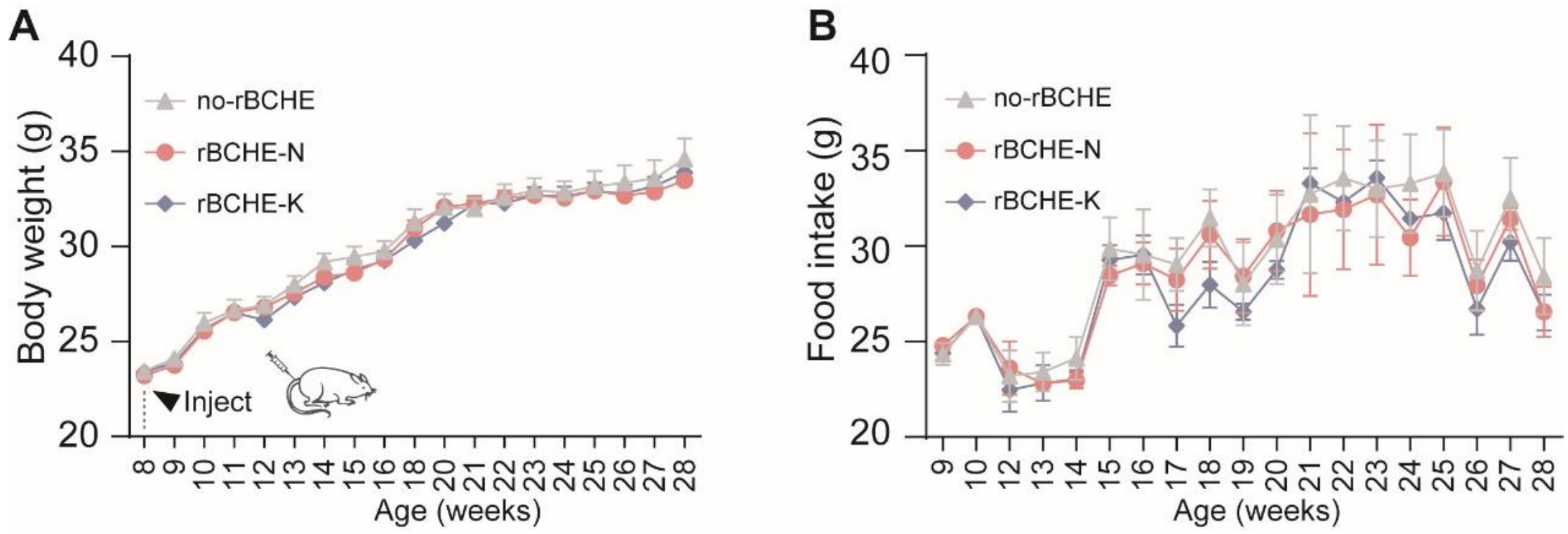

Figure S20. Body weight (A) and Food intake (B) monitoring of C57BL/6J mice, related to Figure 4.

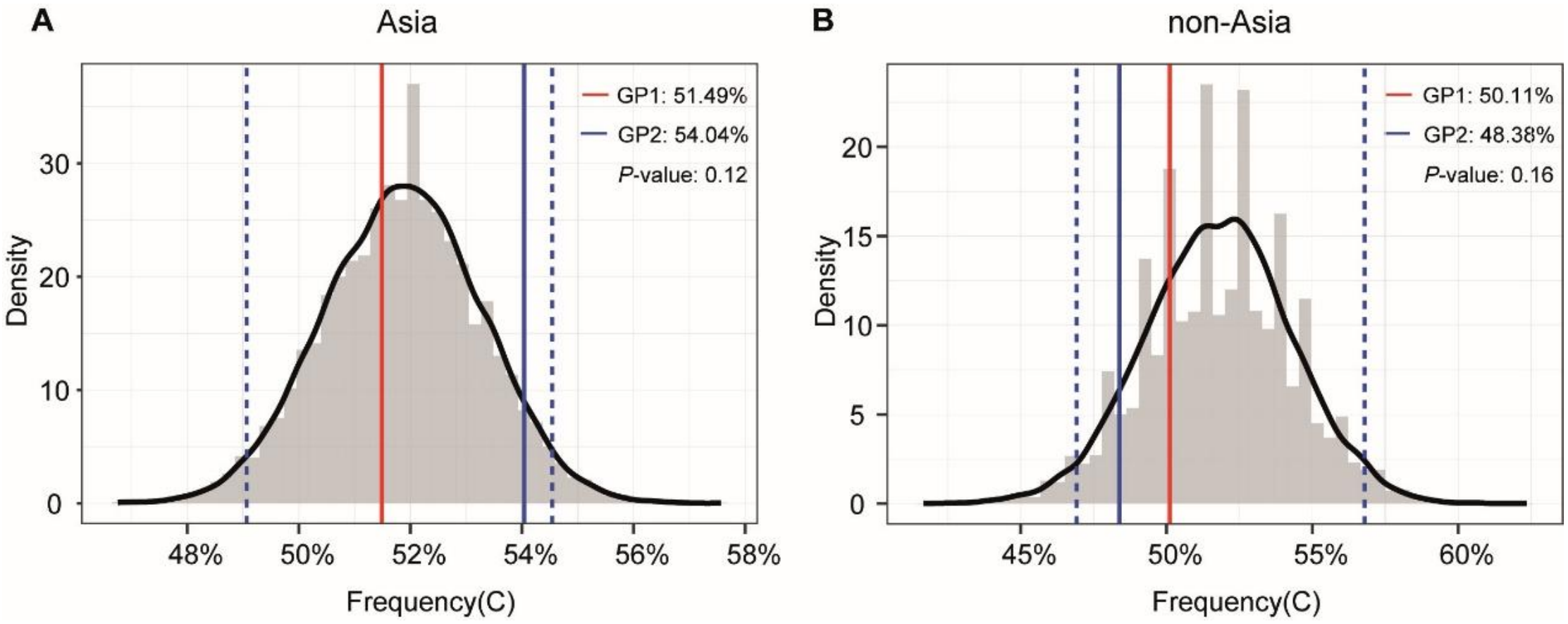

Figure S21. Resampling distribution of allele "C" frequency in Asia (A) and non-Asia (B) population of GP2 dataset, related to Figure 5. GP1 is a previously published global panel of horse genomes dataset. GP2 is this study dataset. The red solid line represents the actual frequency of allele "C" in the GP1 population, while the blue solid line represents the actual frequency of allele "C" in the GP2 population The blue dashed line represents the confidence interval for the GP2 population's weight sampling of 10,000 times.

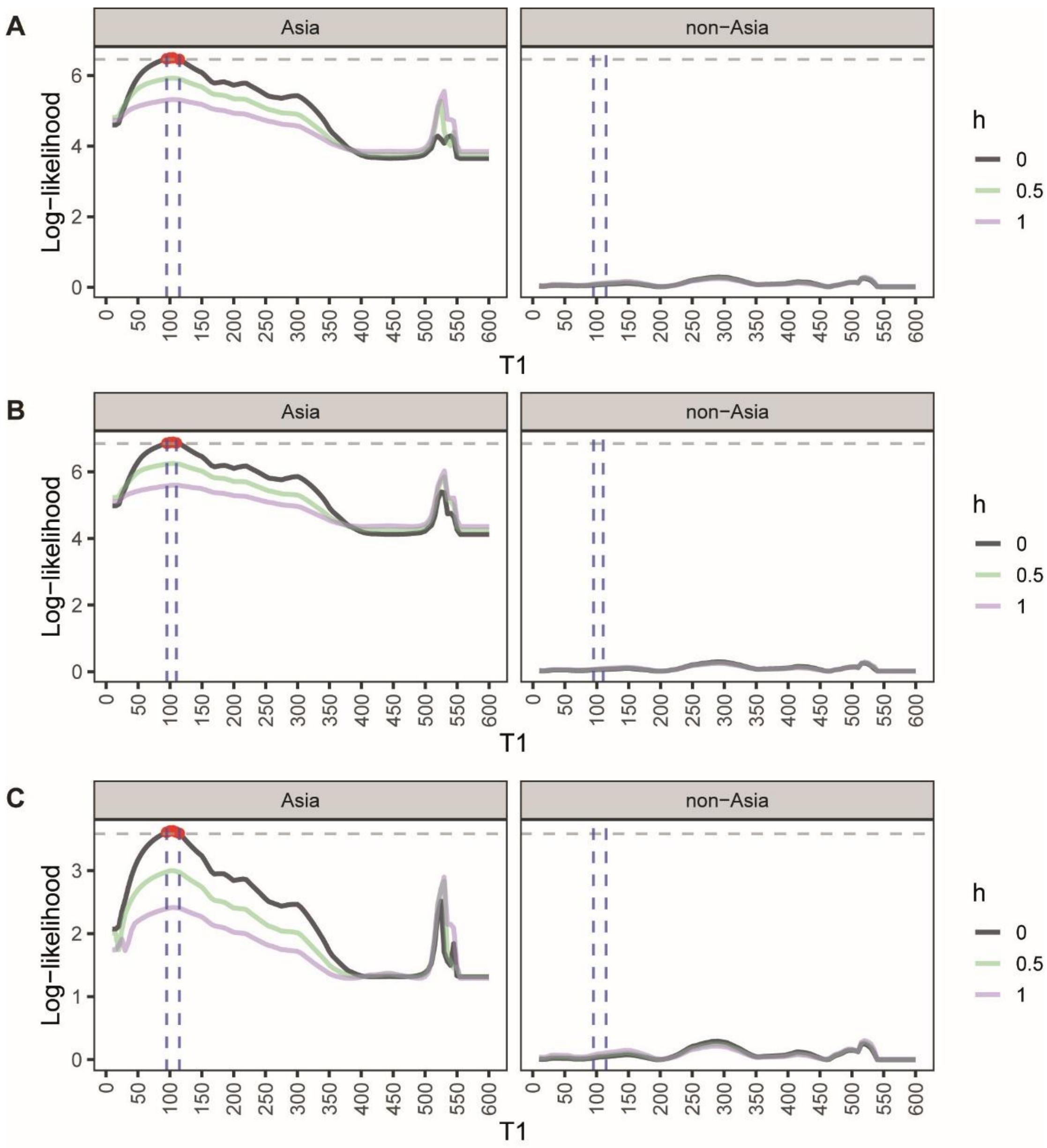

Figure S22. CLUES2 Log-Likelihood with sub-continental demographic models from Liu et al. (2025) (Asia, (A) vs non-Asia, (B)), and the global DOM2 demographic model from Der Sarkissian et al. (2015) (C) , related to Figure 5. Analyses were performed limiting the analyses to those specimens excavated in each individual region, before applying the subcontinental or global demographic model. We use a grid for inferring the time of selection shift, considering every 5 generations from 5 to 600 generations ago. The two vertical lines indicate the generations corresponding to the 99% Confidence Interval (CI).

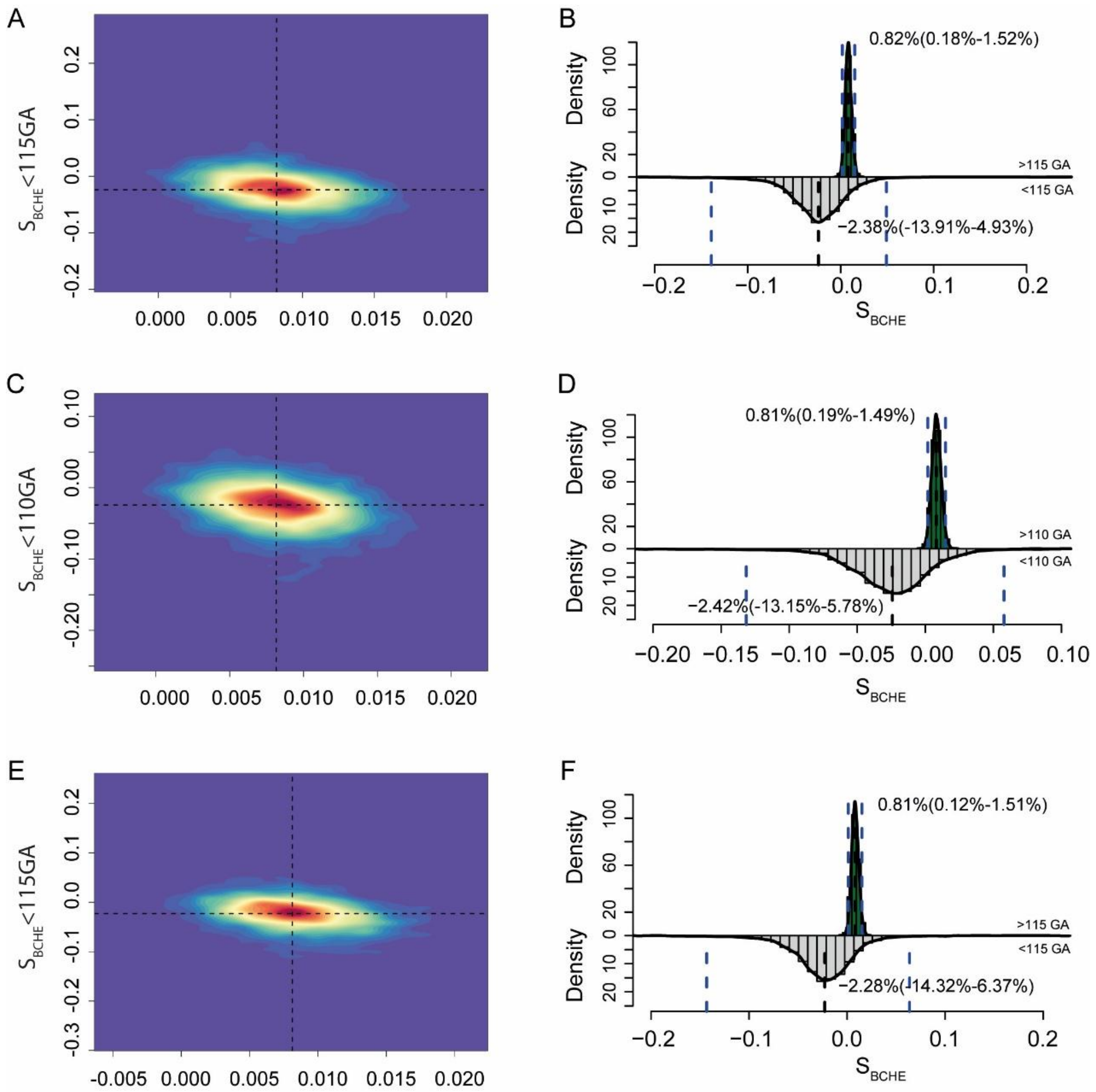

Figure S23. Selection analyses at the BCHE locus (ECA19:9,198,989, G>C) , related to Figure 5.

(A) PMMH joint kernel density posterior distributions for the selection coefficients prior to and after the time when the selection regime changed, using the subcontinental demographic model of Asia.

(B) Same as **(A)**, except that the individual posterior distributions of each selection coefficient are shown.

(C) PMMH joint kernel density posterior distributions for the selection coefficients prior to and after the time when the selection regime changed, using the non-Asian subcontinental demographic model .

(D) Same as **(C)**, except that the individual posterior distributions of each selection coefficient are shown.

(E) PMMH joint kernel density posterior distributions for the selection coefficients prior to and after the time when the selection regime changed, applying the DOM2 demographic model from Der Sarkissian et al. (2015) to ancient horse populations from Asia and outside Asia, separately.

(F) Same as **(E)**, except that the individual posterior distributions of each selection coefficient are shown.

The percentages reported indicate the posterior point estimates for the selection coefficients during the two temporal intervals considered, with 95% confidence intervals shown in parentheses. The blue dashed lines indicate the 95% confidence intervals of the posterior distributions.

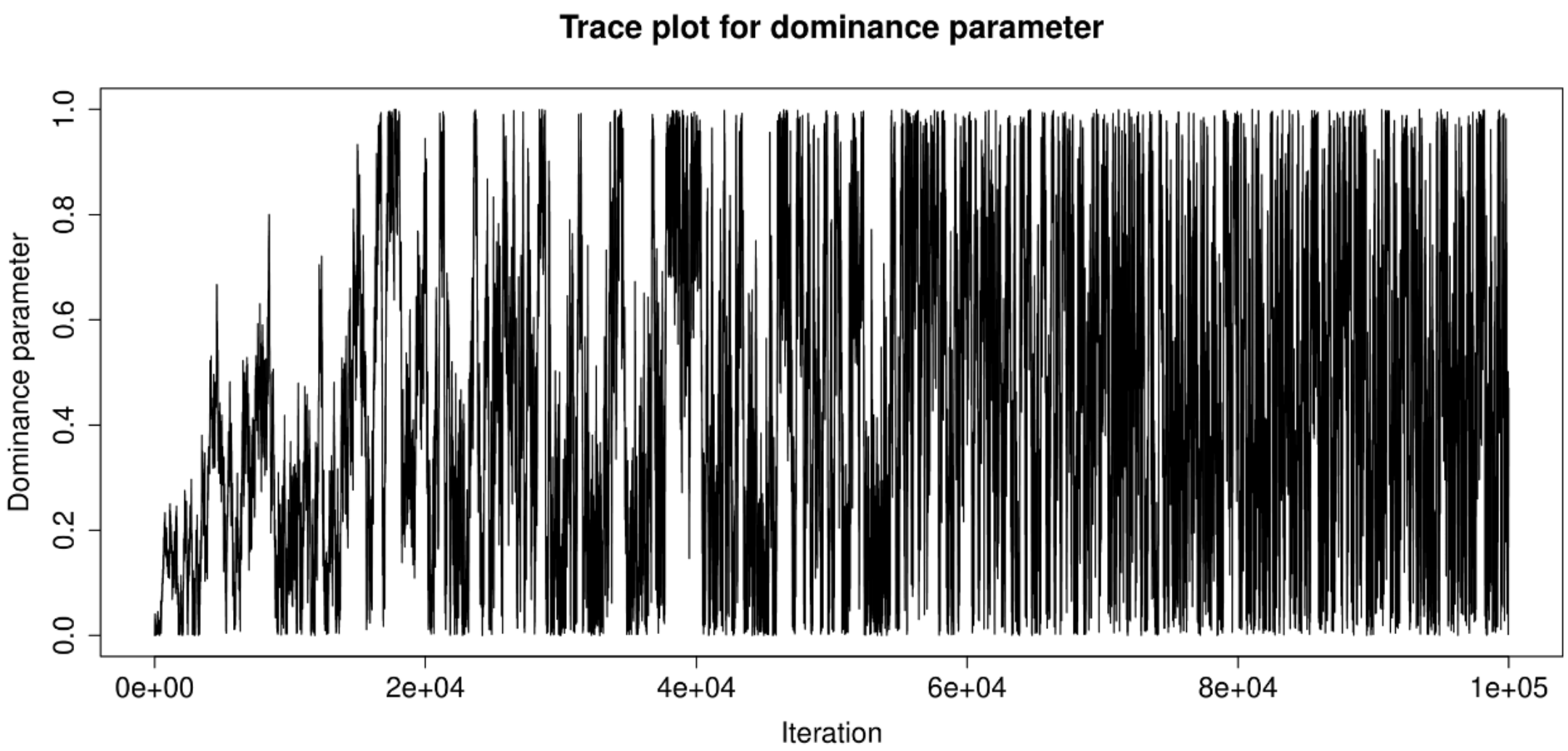

Figure S24. Trace plot of the dominance parameter (h) under the relaxed inheritance model, related to Figure 5.

Supplemental References

[S1] Letunic, I., Khedkar, S., and Bork, P. (2021). SMART: recent updates, new developments and status in 2020. Nucleic Acids Res. *49*, D458-D460. https://doi.org/10.1093/nar/gkaa937

[S2] Kumar, S., Stecher, G., Li, M., Knyaz, C., and Tamura, K. (2018). MEGA X: Molecular Evolutionary Genetics Analysis across Computing Platforms. Mol. Biol. Evol. *35*, 1547-1549. https://doi.org/10.1093/molbev/msy096

[S3] Su, H., Wang, W., Du, Z., Peng, Z., Gao, S.H., Cheng, M.M., and Yang, J. (2021). Improved Protein Structure Prediction Using a New Multi-Scale Network and Homologous Templates. Adv. Sci. *8*, e2102592. https://doi.org/10.1002/advs.202102592

[S4] Du, Z., Su, H., Wang, W., Ye, L., Wei, H., Peng, Z., Anishchenko, I., Baker, D., and Yang, J. (2021). The trRosetta server for fast and accurate protein structure prediction. Nat. Protoc. *16*, 5634-5651. https://doi.org/10.1038/s41596-021-00628-9

[S5] Wang, W., Peng, Z., and Yang, J. (2022). Single-sequence protein structure prediction using supervised transformer protein language models. Nat. Comput. Sci. *2*, 804-814. https://doi.org/10.1038/s43588-022-00373-3

[S6] Pires, D.E.V., Ascher, D.B., and Blundell, T.L. (2014). DUET: a server for predicting effects of mutations on protein stability using an integrated computational approach. Nucleic Acids Res. *42*, W314-W319. https://doi.org/10.1093/nar/gku411

[S7] Pires, D.E.V., Rodrigues, C.H.M., and Ascher, D.B. (2020). mCSM-membrane: predicting the effects of mutations on transmembrane proteins. Nucleic Acids Res. *48*, W147-W153. https://doi.org/10.1093/nar/gkaa416

[S8] Pandurangan, A.P., Ochoa-Montaño, B., Ascher, D.B., and Blundell, T.L. (2017). SDM: a server for predicting effects of mutations on protein stability. Nucleic Acids Res. *45*, W229-W235. https://doi.org/10.1093/nar/gkx439

[S9] Liu, X., Zhang, Y., Li, Y., Pan, J., Wang, D., Chen, W., Zheng, Z., He, X., Zhao, Q., Pu, Y., *et al.* (2019). EPAS1 Gain-of-Function Mutation Contributes to High-Altitude Adaptation in Tibetan Horses. Mol. Biol. Evol. *36*, 2591-2603. https://doi.org/10.1093/molbev/msz158

[S10] McGarry, K.G., Schill, K.E., Winters, T.P., Lemmon, E.E., Sabourin, C.L., Harvilchuck, J.A., and Moyer, R.A. (2020). Characterization of Cholinesterases From Multiple Large Animal Species for Medical Countermeasure Development Against Chemical Warfare Nerve Agents. Toxicol. Sci. *174*, 124-132. https://doi.org/10.1093/toxsci/kfz250

[S11] McGarry, K.G., Lalisse, R.F., Moyer, R.A., Johnson, K.M., Tallan, A.M., Winters, T.P., Taris, J.E., McElroy, C.A., Lemmon, E.E., Shafaat, H.S., *et al.* (2020). A Novel, Modified Human Butyrylcholinesterase Catalytically Degrades the Chemical Warfare Nerve Agent, Sarin. Toxicol. Sci. *174*, 133-146. https://doi.org/10.1093/toxsci/kfz251

[S12] Liu, X., Jia, Y., Pan, J., Zhang, Y., Gong, Y., Wang, X., Ma, Y., Alvarez, N., Jiang, L., and Orlando, L. (2025). Selection at the GSDMC locus in horses and its implications for human mobility. Science *389*, 925-930. https://doi.org/10.1126/science.adp4581
